# Room temperature olefination of methane with titanium–carbon multiple bonds†
†Electronic supplementary information (ESI) available. CCDC 1590107 and 1590108. For ESI and crystallographic data in CIF or other electronic format see DOI: 10.1039/c7sc05238c


**DOI:** 10.1039/c7sc05238c

**Published:** 2018-02-13

**Authors:** Takashi Kurogi, Joonghee Won, Bohyun Park, Oleksandra S. Trofymchuk, Patrick J. Carroll, Mu-Hyun Baik, Daniel J. Mindiola

**Affiliations:** a Department of Chemistry , University of Pennsylvania , Philadelphia , PA 19104 , USA . Email: mindiola@sas.upenn.edu; b Department of Chemistry , Korea Advanced Institute of Science and Technology (KAIST) , Daejeon 34141 , Republic of Korea . Email: mbaik2805@kaist.ac.kr; c Center for Catalytic Hydrocarbon Functionalizations , Institute for Basic Science (IBS) , Daejeon 34141 , Republic of Korea

## Abstract

C–H activation of methane followed by dehydrocoupling at room temperature led ultimately to the formation of the olefin H_2_C=CH^*t*^Bu *via* the addition of redox-active ligands (L) such as thioxanthone or 2,2′-bipyridine (bipy) to (PNP)Ti=CH^*t*^Bu(CH_3_) (**1**). Using both of these exogenous ligand systems, we could trap the titanium fragment *via* an insertion reaction with these two substrates to afford species of the type (PNP)Ti(L)(LH). A combination of computational and isotopic labeling studies reveals that the L ligand promotes the C–C bond forming step by migration of the methyl moiety in **1** to the α-alkylidene carbon by producing a Ti(iii) species (PNP)Ti{CH(CH_3_)^*t*^Bu}(L). In the case of L = thioxanthone, β-hydrogen abstraction gives an olefin, whereas with 2,2′-bipyridine β-hydride elimination and migratory insertion lead to (PNP)Ti(L)(LH). These redox-active ligands play two important roles: (i) they accept an electron from the Ti-alkylidene fragment to allow the methyl to approach the alkylidene and (ii) they serve as traps of a hydrogen atom resulting from olefin elimination. These systems represent the first homogeneous models that can activate methane and selectively dehydrocouple it with a carbene to produce an olefin at room temperature.

## Abstract 2

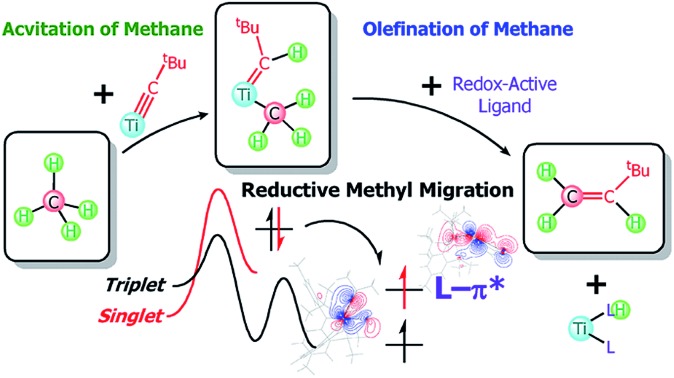
C–H activation of methane followed by dehydrocoupling at room temperature led ultimately to the formation of the olefin H_2_C=CH^*t*^Bu *via* the addition of redox-active ligands (L) such as thioxanthone or 2,2′-bipyridine (bipy) to (PNP)Ti=CH^*t*^Bu(CH_3_) (**1**).

## Introduction

The non-oxidative conversion of methane to an olefin is fundamentally an endergonic process, and hence only a handful examples exist, where this remarkable reaction was seen.^1^ The challenge lies in making weaker C–C and C=C bonds at the expense of breaking the stronger C–H bonds of methane or another alkane. Surface supported organometallic reagents and heterogeneous catalysts with a sulfur-based hydrogen acceptor were reported previously, but they require high temperatures with concurrent release of toxic H_2_S.^2^,^3^ The olefination of methane at room temperature using a well-defined organometallic reagent has not been possible thus far. We were inspired by a few well-defined organometallic complexes that can undergo methyl migration by forming C–C bonds, especially under mild conditions.^4^–^9^ Notably, in two examples the methyl moiety was derived from methane as in Cp*_2_Sc(CH_3_)^8^ and Cp*W(NO)(CH_3_)(η^3^-CH_2_CHCMe_2_).^9^ In the case of Sc, Tilley and Sadow reported the catalytic hydromethylation of propene with methane by Cp*_2_Sc(CH_3_) at room temperature.^8^ This process requires σ-bond metathesis, migratory insertion of an olefin such as propene, and finally σ-bond metathesis again to form a homologation product according to the reaction CH_4_ + H_2_C=CHCH_3_ → H_3_CCH(CH_3_)_2_. More recently, Legzdins and co-workers found that addition of CO under high pressure to Cp*W(NO)(CH_3_)(η^3^-CH_2_CHCMe_2_), a species derived from methane activation, could result in insertion into the methyl ligand ultimately leading to extrusion of a mixture of β,γ-unsaturated ketones.^9^

**Scheme 1:**
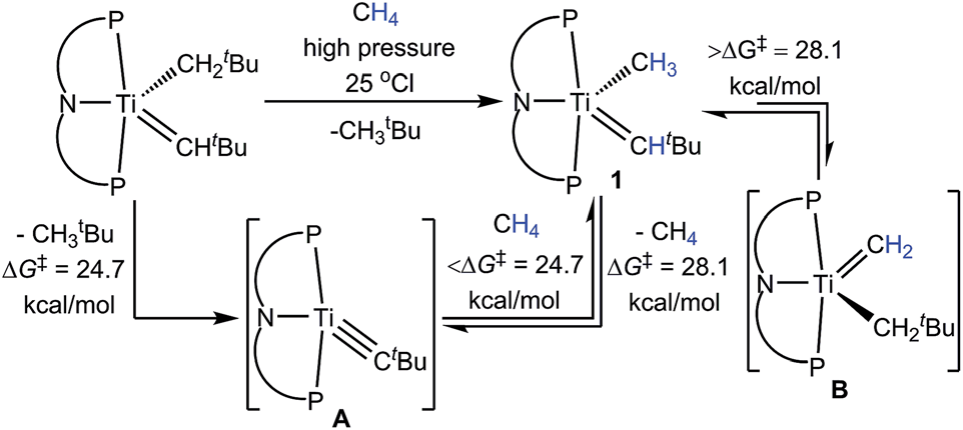
Methane activation *via* intermediate **A** as well as elimination and dehydrogenation.

We previously reported how complex (PNP)Ti=CH^*t*^Bu(CH_2_^*t*^Bu) can eliminate H_3_C^*t*^Bu to form transient (PNP)Ti≡C^*t*^Bu (**A**), which then activates CH_4_*via* 1,2-CH bond addition to form the neopentylidene–methyl (PNP)Ti=CH^*t*^Bu(CH_3_) (**1**) (Scheme 1).^10^,^11^ The formation of **A** was found to be rate-determining with a Δ*G*^‡^ of 24.7 kcal mol^–1^ based on kinetic studies (calculated Δ*G*^‡^: 27.8 kcal mol^–1^).^12^ Although complex **1** can also extrude CH_4_ to reform **A**, this process was found to be much slower with an associated Δ*G*^‡^ of 28.1 kcal mol^–1^ (calculated Δ*G*^‡^: 33.0 kcal mol^–1^).^10^ Notably, it was found that **1** tautomerizes to the methylidene (PNP)Ti=CH_2_(CH_2_^*t*^Bu) (**B**), but does so also very slowly with a Δ*G*^‡^ > 28.1 kcal mol^–1^ (calculated Δ*G*^‡^: 34.4 kcal mol^–1^).^10^ Therefore, both elimination and dehydrogenation of CH_4_ can take place competitively. More evidence for **B** being the more reactive tautomeric form, was derived from an independent synthesis involving “CH_2_” group transfer from a phosphorus ylide to the titanium olefin complexes of the type (PNP)Ti(CH_2_^*t*^Bu)(η^2^-olefin).^13^ Upon transfer of “CH_2_”, **B** is not observed, but instead tautomerizes quickly to **1** corroborating our claim that **B** lies 7.8 kcal mol^–1^ higher in energy to **1** (based on calculated free energies).^10^,^13^

## Results and discussion

**Scheme 2:**
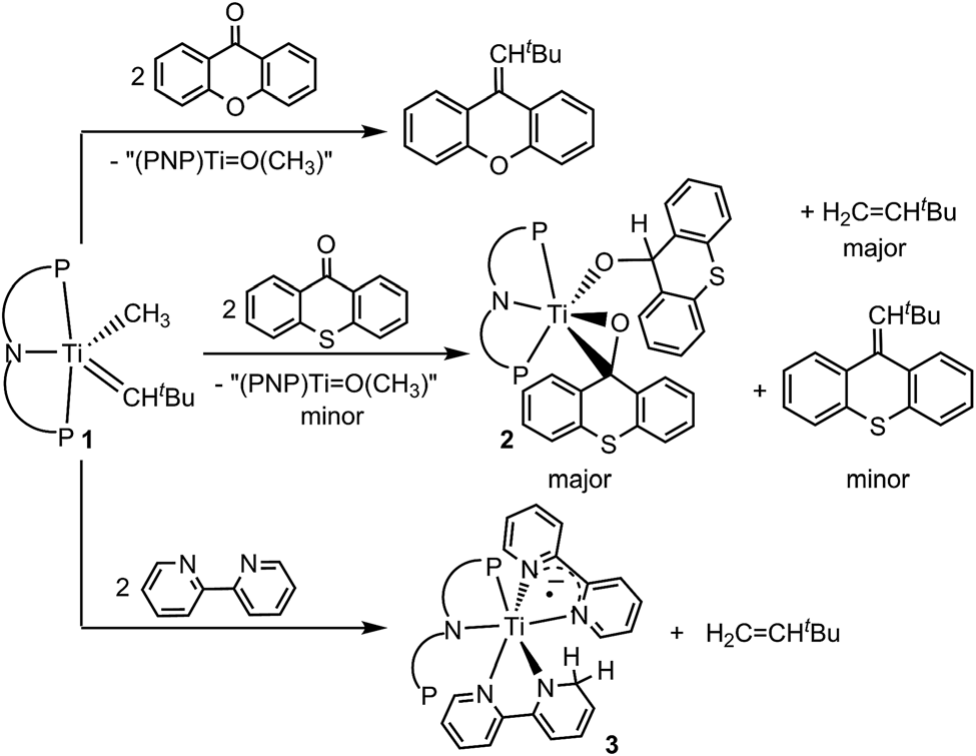
Reactivity of **1** with the ketones such as xanthone and thioxanthone as well as 2,2′-bipyridine.

Based on this premise, we studied tautomer **B** since it contains the dehydrocoupled form of methane and may allow for accessing olefins *via* reductive coupling.^14^,^15^ Particularly promising is the use of exogenous ligands that may bind to the Ti(iv) center without promoting α-hydrogen abstraction to form **A** by losing CH_4_. Unfortunately, complexes **1** and intermediates **A** and **B** pose some challenges since they contain reactive and nucleophilic alkylidyne and alkylidene moieties that may engage in Wittig-like chemistry.^10^ We first used the rigid ketone xanthone and found that it engages with **1**, albeit slowly to carry out the expected Wittig-like chemistry and produce ^*t*^BuHC=C_13_H_8_O as shown in Scheme 2. The putative titanium oxo complex [(PNP)Ti=O(CH_3_)] could not be detected and instead, a mixture of metal-based products was observed by ^31^P NMR spectroscopy.^16^ Our inability to isolate (PNP)Ti=O(CH_3_) was not unexpected since the close analogue, (PNP)Ti=O(CH_2_^*t*^Bu), is known to decompose rather quickly in solution.^17^ Surprisingly, when thioxanthone (O=CC_12_H_8_S) is used instead, we observed some Wittig-like reactivity along with an additional olefin, which was identified to be H_2_C=CH^*t*^Bu on the basis of ^1^H NMR spectroscopy and GC-MS (Scheme 2).^16^ Formation of H_2_C=CH^*t*^Bu was unambiguously confirmed when compared to an independently prepared sample. Since the reaction mixture contained some unreacted **1**, performing the same transformation using a 2 equiv. of thioxanthone produced higher yields of ^*t*^BuHC=C_13_H_8_S and H_2_C=CH^*t*^Bu in approximately 1 : 9 based on the ^1^H NMR spectrum. Under these conditions, we were also able to isolate the titanium complex (PNP)Ti(η^2^-O=CC_12_H_8_S)(OCHC_12_H_8_S) (**2**) in 52% yield (Scheme 2).

**Fig. 1:**
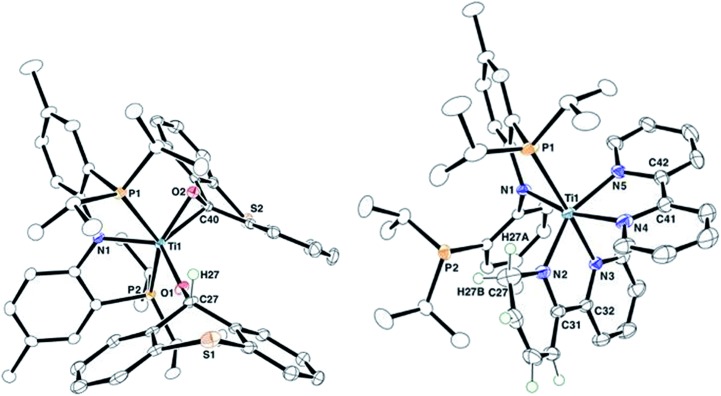
Solid-state structures of complex **2** (left) and **3** (right) with thermal ellipsoids at the 50% probability level. H-atoms with the exception of β-H (**2**) and bipyH (**3**) have been omitted for clarity.

Complex **2** has been characterized by ^1^H, ^13^C, and ^31^P NMR spectroscopy in addition to solid-state X-ray diffraction analysis. A combination of isotope labeling experiments and multidimensional NMR spectra clearly assigned the thioxanthoxide hydrogen Ti–OC***H*** at 6.88 (^1^H NMR) and carbon Ti–O***C***H at 83.8 (^13^C NMR) ppm. The most notable features in the solid-state structure of **2** are the presence of a η^2^-(*O*,*C*) bound thioxanthone in addition to a coordinated thioxanthoxide resulting from a hydrogen adding to the ketone carbon of the formal thioxanthone (Fig. 1, left). The coordinated nature of the thioxanthone (C–O, 1.375(2) Å) and presence of a thioxanthoxide imply the ketone ligand to serve as a π-acid but also hydrogen acceptor from the formal methyl group of **1**. The formation of H_2_C=CH^*t*^Bu indicated that both methyl and neopentylidene ligands have undergone dehydrocoupling in **1**. To circumvent the Wittig-like reaction observed between **1** and thioxanthone, we resorted to a ligand that lacked the ketone unit but which could be resistant to alkyl and alkylidene units. Treatment of **1** with two equiv. of 2,2′-bipyridine (bipy) in benzene over 24 hours resulted in the formation of H_2_C=CH^*t*^Bu along with the titanium complex (PNP)Ti(bipy)(bipyH) (**3**), that was isolated in 33% yield (Scheme 2). Akin to **2**, a combination of isotopic labeling experiments and multidimensional NMR spectra clearly assign the hydrogen for the reduced bipyH at 6.80 ppm. As a result of the aromaticity being perturbed due to hydrogen addition to the bipy ligand, six olefinic resonances are also observed in the 4.15–6.03 ppm region in the ^1^H NMR spectrum where three of these resonances at 4.15, 4.83 and 5.87 ppm could be assigned for the hydropyridyl unit of bipyH by multidimensional NMR spectroscopy. In addition, the ^31^P NMR spectrum shows two broad resonances at 53.0 and 55.0 ppm indicating the fluxional behavior of P arms in solution. A solid-state structure confirmed our proposed formula showing a κ^2^-bound PNP ligand as well as one coordinated bipy ligand and a second reduced form of the bipy, namely bipyH (Fig. 1, right). Fortunately, hydrogens atoms were located and refined isotropically allowing us to pinpoint the locus of reduction of the bipy ligand, that being at the 6 position. The C27–N2 position has been significantly elongated (1.464(3) Å) when compared to co-crystallized bipy (1.345(3), 1.342(2) Å), thus resulting in some puckering of the ring and rendering this scaffold overall monoanionic. Complex **3** has a deep indigo color, which is generally atypical among high-valent titanium complexes. A UV-Vis spectrum of **3** shows a strong absorption band at 565 nm (*ε* = 8370 M^–1^ cm^–1^), which we propose contributes to the violet color.^18^ The latter property is in accord with this species possessing a π-radical bipy ligand and we assign this feature as an MLCT, consistent with **3** having a Ti(iii) radical antiferromagnetically coupled to a bipy˙^–^.^18^,^19^

**Fig. 2:**
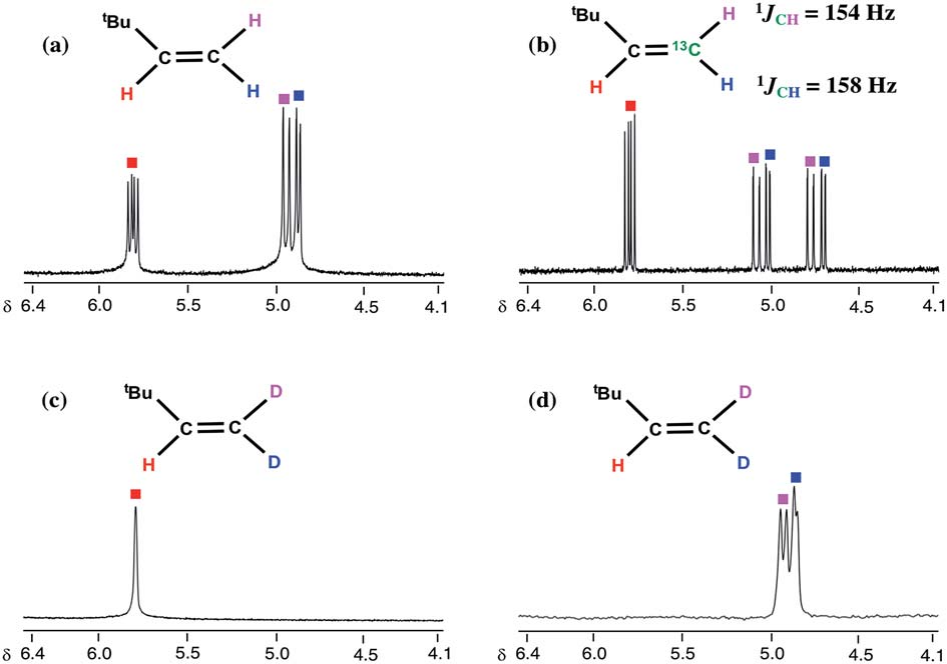
Expanded ^1^H and ^2^H NMR spectra of H_2_C=CH^*t*^Bu (a), H_2_^13^C=CH^*t*^Bu (b), and D_2_C=CH^*t*^Bu (^1^H: (c), ^2^H: (d)) obtained from **1**, **1**-^13^C, and **1**-D_3_ with thioxanthone and bipy.

**Scheme 3:**
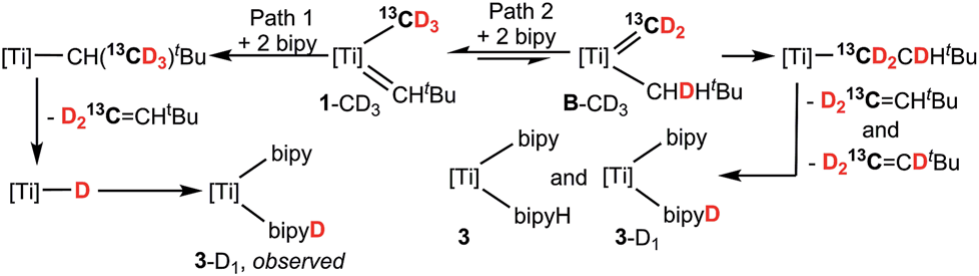
Isotopic labelling studies using **1**-^13^C and **1**-D_3_ (labelled **1**-^13^CD_3_ for simplicity) with two equiv. of bipy. The role of bipy and PNP are not shown for clarity ([Ti] = (PNP)Ti).

To establish the origin of the inserted hydrogen in both **2** and **3**, as well as propose the most likely pathway to C–C bond formation, we conducted isotopic labelling studies using the ^13^C and ^2^H isotopomers (PNP)Ti=CH^*t*^Bu(^13^CH_3_) (**1**-^13^C) and (PNP)Ti=CH^*t*^Bu(CD_3_) (**1**-D_3_), respectively, with 2 equiv. of bipy and thioxanthone.^16^ As expected, examination of the reaction mixture using **1**-^13^C irrefutably revealed the formation of H_2_^13^C=CH^*t*^Bu and **3** (Fig. 2b) whereas using **1**-D_3_ formed only D_2_C=CH^*t*^Bu (Fig. 2c and d) and (PNP)Ti(bipy)(bipyD) (**3**-D_1_) based on a combination of ^1^H, ^13^C, and ^2^H NMR spectra.^16^ These results therefore confirm that methane is the source of the terminal methylidene unit in H_2_C=CH^*t*^Bu and that methyl migration to the neopentylidene most likely occurs by Path 1, as opposed to the less plausible Path 2 scenario involving tautomerization to **B** followed by neopentyl migration (Scheme 3). Although tautomerization of **1** to **B** should be a slow process, our labeling study cannot completely preclude the possibility of secondary isotope effects further slowing down the rate of conversion between the two, and therefore further discouraging the formation of **B**. Fig. 2 depicts NMR spectral data for the olefin formed (isolated by vacuum transfer of the mixture): ^1^H NMR spectrum when unlabeled material **1** is used in the presence of bipy or thioxanthone (a); ^1^H NMR spectrum when **1**-^13^C is used in the presence of bipy and thioxanthone (b); ^1^H NMR spectrum when **1**-D_3_ is used in the presence of bipy and thioxanthone (c); and ^2^H NMR spectrum when **1**-D_3_ is used in the presence of bipy and thioxanthone (d).

**Fig. 3:**
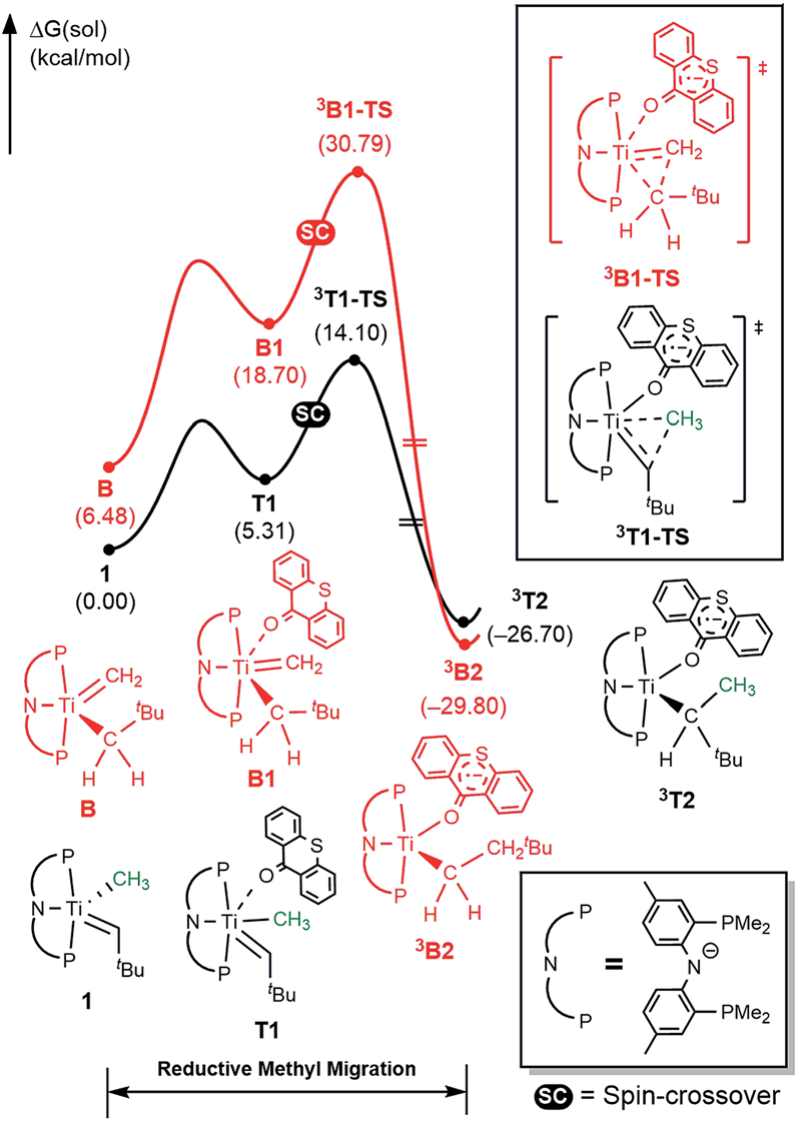
Free energy profile for reductive C–C coupling. Black and red traces represent the reaction pathways initiated from (PNP)Ti=CH^*t*^Bu(CH_3_) (**1**) and (PNP)Ti=CH_2_(CH_2_^*t*^Bu) (**B**), respectively.

To better understand the mechanism, quantum chemical calculations based on density functional theory (DFT) were carried out. The free energy profiles for the two possible pathways of reductive migration of the methyl moiety are illustrated in Fig. 3. The tautomerized intermediate **B** is located at a relative free energy that is 6.5 kcal mol^–1^ higher than **1**. Both isomers may first bind one equivalent of thioxanthone (T) to afford the intermediates **B1** and **T1** at 18.7 and 5.3 kcal mol^–1^, respectively. These six-coordinate complexes containing a methylidene-alkyl or a methyl-alkylidene moieties may then undergo the reductive C–C coupling reaction traversing the triplet transition states **^3^B1-TS** and **^3^T1-TS** associated with reaction barriers of 30.8 and 14.1 kcal mol^–1^, respectively. These calculations suggest that the reductive C–C coupling is most easily initiated from complex **1**, rather than its tautomer **B** consistent with the isotopic labeling studies (*vide supra*).

**Fig. 4:**
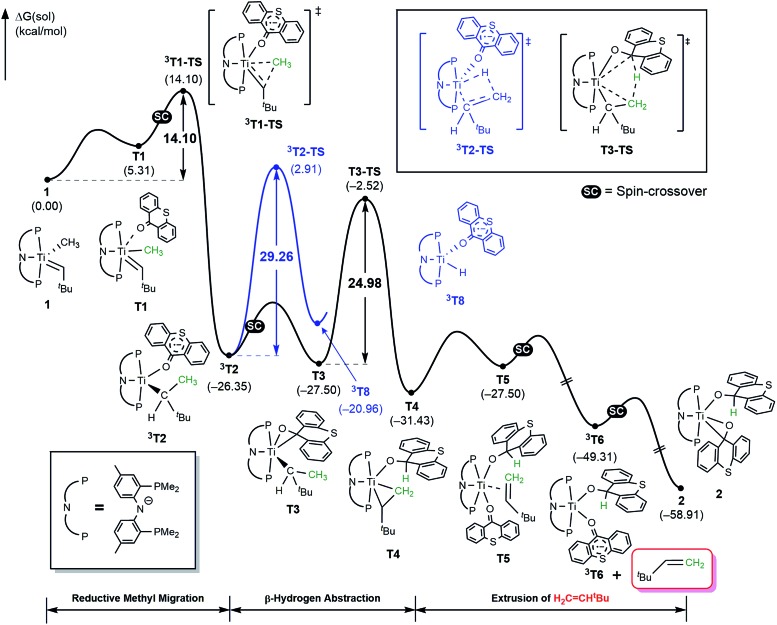
Free energy profile for the formation of H_2_C=CH^*t*^Bu using thioxanthone ligand. Black trace represents the direct β-hydrogen abstraction to thioxanthone which is the main pathway. Blue trace represents another plausible triplet pathway *via* classical β-hydride elimination.

The activation of methane by **A** at room temperature was investigated previously^12^ and, thus, we begin our mechanistic study with intermediate **1**. As shown in Fig. 4, the reaction begins with a weak coordination of thioxanthone (T) to afford the ketone adduct **T1**, which undergoes irreversible reductive methyl migration to form **^3^T2**. Interestingly, we found that the reduction of the metal is accompanied by a singlet to triplet spin-crossover, as the newly formed Ti(ii)-d^2^ center adopts a high-spin triplet configuration. In good agreement with experimental results, the barrier of this key step associated with **^3^T1-TS** is only 14.1 kcal mol^–1^, suggesting that the methyl migration will be much faster than α-hydrogen abstraction to produce CH_4_ and **A**, which typically requires much higher activation energies in excess of ∼30 kcal mol^–1^. Such a low barrier for a C–C forming reaction is rare^6^–^8^ and more detailed analysis of our computer model reveals that the low barrier results from the T-ligand assisting the delocalization of unpaired electron density, which in turn facilitates the spin-crossover.

**Fig. 5:**
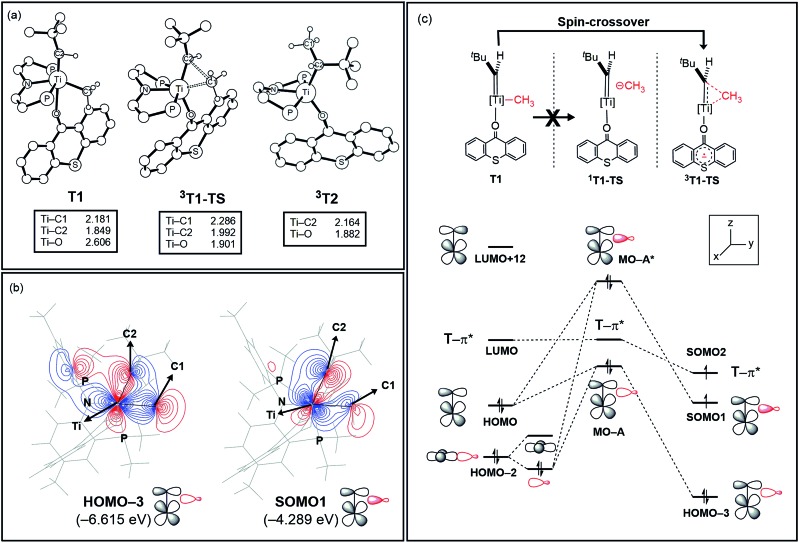
(a) Optimized structures of **T1**, **^3^T1-TS**, and **^3^T2**. Non-essential atoms have been omitted for clarity. (b) Contour plots key MOs of **^3^T1-TS**. (c) Conceptual MO-diagram explaining the role of the spin-crossover.

Fig. 5a summarizes the structural changes that the titanium complex undergoes during spin-crossover and reductive C–C coupling. In the six-coordinated intermediate **T1**, the T-ligand is weakly bound with a Ti–O distance of 2.61 Å and is arranged in *trans* disposition to the alkylidene fragment. As the reductive C–C coupling traverses through the transition state **^3^T1-TS**, significant structural and electronic rearrangements take place. First and foremost, the T-ligand binds much more tightly displaying a Ti–O distance of 1.90 Å, whereas the double-bond between Ti and the alkylidene-carbon is notably lengthened from 1.85 to 1.99 Å. The T-ligand exerts a strong *trans*-effect by removing electron-density from the Ti-alkylidene bond into a stronger Ti–O σ-bond and allowing for an easier activation of the Ti=C bond.

**Fig. 6:**
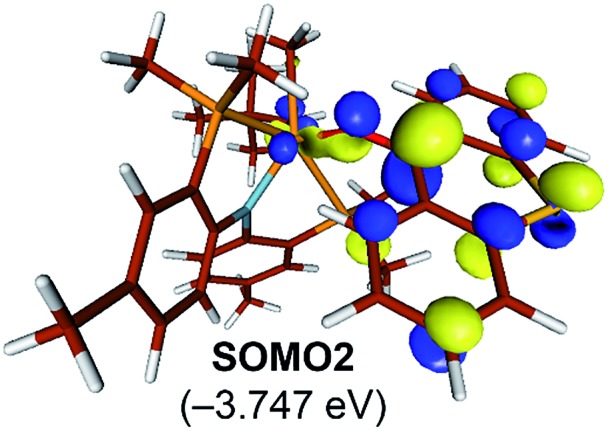
Isosurface plots of singly occupied molecular orbital of T–π* in **^3^T1-TS** (isodensity value = 0.05 a.u.).

The electronic distortion accompanying the spin-crossover is illustrated in Fig. 5c and one plausible way of understanding it is as follows: The C–C bond formation requires that the Ti–CH_3_ bond is formally cleaved to transiently give a methyl-anion, which may attack the Ti-alkylidene double-bond. As shown in Fig. 5c, this scenario leads to a filled–filled interaction between the two Lewis basic fragments in the putative singlet transition state **^1^T1-TS**. The two main orbital interactions are drawn as MO-A, the in-phase combination of the methyl lone-pair orbital with the π-orbital of the Ti-alkylidene moiety, and as MO-A*, the corresponding antibonding combination. Both orbitals are of course occupied, since these are interactions of two filled fragment orbitals. We were unable to locate this putative transition state, because the π*-orbital of the thioxanthone ligand T–π* is the lowest unoccupied molecular orbital (LUMO) of the **T1** complex and will also be low enough in energy in the putative **^1^T1-TS** which will be lower than MO-A*. Consequently, calculations naturally converge to a state where an intramolecular electron-transfer from the highly unfavorable MO-A* orbital to the low-lying T–π* orbital will afford a much more favorable electronic structure. As the T–π* orbital places the electron far away from the metal center, the metal-containing frontier orbitals will move to lower energies, giving the final orbital energy ordering found in **^3^T1-TS**. What was labeled as MO-A is found in **^3^T1-TS** as HOMO–3 at –6.62 eV and the corresponding antibonding combination, conceptually labeled MO-A*, is found as SOMO1 at –4.29 eV (Fig. 5b). Interestingly, the π* orbital of the thioxanthone ligand becomes the SOMO2 at an energy of –3.75 eV. Overall, this complex electronic reorganization leads to a situation where one electron is placed in the thioxanthone-π* orbital, shown in Fig. 6, and one remains in the antibonding orbital between the methyl and alkylidene-π orbital. One interesting question in this mechanism is whether the electron placed in the T–π* orbital originated from the methyl-anion or the Ti-alkylidene fragment formally. Whereas it is not possible to assign origins of electrons at the transition state based on quantum chemical simulations, it is of course most plausible to envision that the electron from the more polarizable Ti-alkylidene π-cloud is transferred to the T-ligand, rather than that from the methyl-group. Assigning the formal oxidation to be methyl-centered is also inconsistent with the structural features required for the C–C coupling and confirmed in our calculations. As the methyl-radical would prefer a trigonal-planar geometry, removal of the electron from the methyl moiety would lead to a geometry that is not aligned with the reaction coordinate of the C–C coupling. In contrast, removal of an electron from the Ti-alkylidene π-cloud leads to a lengthening of the Ti–C double bond, which is in good alignment with the structural distortion needed for the reductive C–C coupling. Thus, we propose that the electron temporarily placed in the T–π* orbital originates from the titanium-alkylidene double bond.

This conceptual MO-analysis is helpful, because it provides a compelling and simple explanation for why the computed barrier for the reductive C–C coupling is so low. The thioxanthone ligand acts as a temporary electron storage unit where one of the electrons can be placed during the C–C coupling reaction. Once the insertion completes and the C–C bond is formed, the SOMO1 becomes a classical Ti-centered frontier orbital to afford the one-electron reduced Ti(iii)-complex formally. In order to test the mechanistic role of the thioxanthone ligand identified in our calculations, we carried out a series of experiments. First, phosphine (PMe_3_) and pyridine ligands were used as exogenous ligands with the expectation that they will be unable to function in a similar manner as an electron acceptor if the computational results are correct. And, indeed, when complex **1** is treated with either ligand only gave activation of solvent is observed *via* α-hydrogen abstraction to form CH_4_ and alkylidyne A.^16^ We have also carried out calculations on these two systems and found that the lowest barriers for the C–C coupling in these systems were 37.8 kcal mol^–1^ and 35.0 kcal mol^–1^, respectively, emphasizing the magnitude of the impact that the electronic reorganization has on the transition state energy. Details are given in the ESI.† These findings support our proposal that the exogenous ligand must have low lying π* orbitals and act as an electron reservoir to allow for the C–C coupling to proceed. This is an interesting finding, as the exogenous ligand is proposed to not be involved in the C–C coupling process in a classical sense such as assisting the approach of the two coupling partners, but rather facilitates the reductive component of the reductive C–C coupling in a non-classical mechanism by making the Ti-alkylidene moiety cationic like.

As illustrated in Fig. 4, the formation of **^3^T2** is irreversible and highly exergonic. To push the reaction forward and form the olefin product, the alkyl moiety must be dehydrogenated, and there are several mechanistic possibilities. Some time ago, Werner reported that [(C_6_R_6_)(L)M(=CH_2_)(CH_3_)]^+^ (M = Ru, Os; L = CO, phosphine; R = H, Me) can engage in methyl migration to form a C–C bond and proposed that a subsequent β-hydride elimination may generate H_2_C=CH_2_ and [M–H].^6^ Thus, we probed for a β-hydride elimination directly from **^3^T2** and found the transition state **^3^T2-TS** to have a barrier of 29.3 kcal mol^–1^. In search of an alternative, lower energy transition state, we considered the singlet spin state analogue **T3**, which was found 1.2 kcal mol^–1^ lower in energy. And, indeed, we were able to locate the transition state **T3-TS** that gives a barrier of 25.0 kcal mol^–1^ for the dehydrogenation of the methyl group on the singlet potential energy surface. Interestingly, this transition state does not lead to the anticipated β-hydride elimination to give a metal-hydride. Instead, the hydride is transferred concertedly to the carbonyl-carbon of the thioxanthone functionality, as illustrated in Fig. 4, and the titanacyclopropane complex **T4** is formed. Our calculations suggest that this step is most difficult energetically with a barrier of 25 kcal mol^–1^, which is in good agreement with the experimental observation that this reaction does occur at room temperature. The final steps of the reaction involve product release and addition of another equivalent of the thioxanthone substrate, where either the singlet or triplet spin configurations are adopted to lower the total energy.

**Fig. 7:**
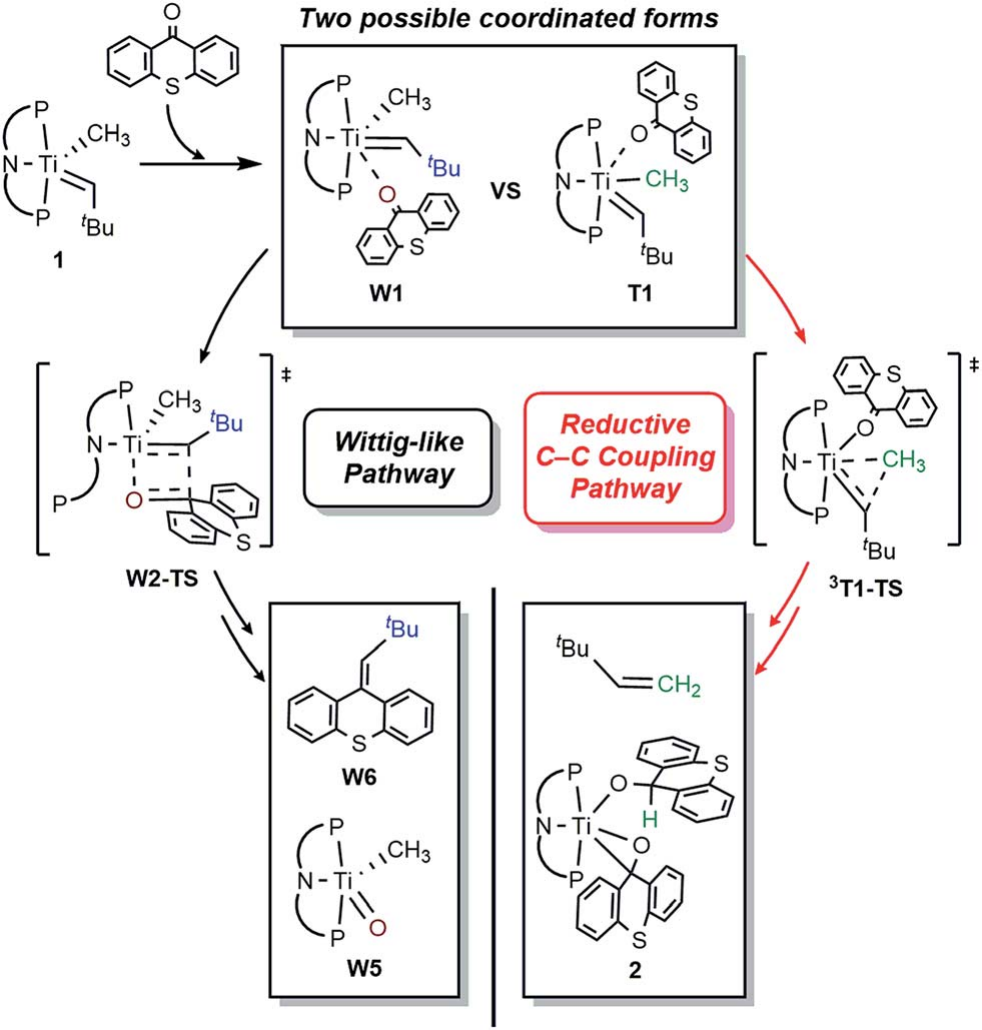
Two possible reaction pathways using thioxanthone (T) with truncated several mechanistic steps for the brief description.

**Fig. 8:**
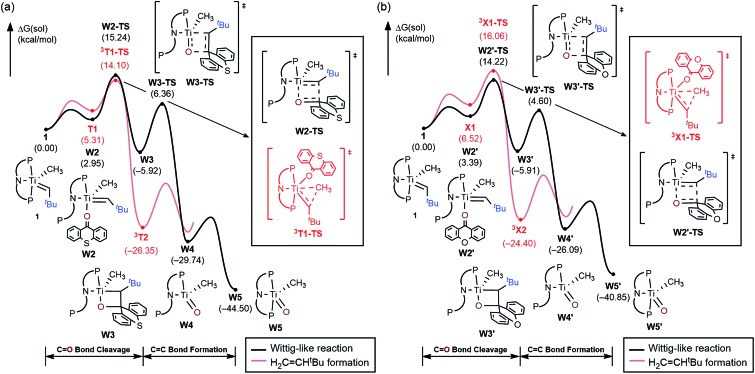
Free energy profile for the Wittig-like reaction and formation of H_2_C=CH^*t*^Bu starting from **1**. Black and red traces represent the Wittig-like reaction and truncated H_2_C=CH^*t*^Bu formation pathway respectively. (a) Thioxanthone (b) xanthone.

**Fig. 9:**
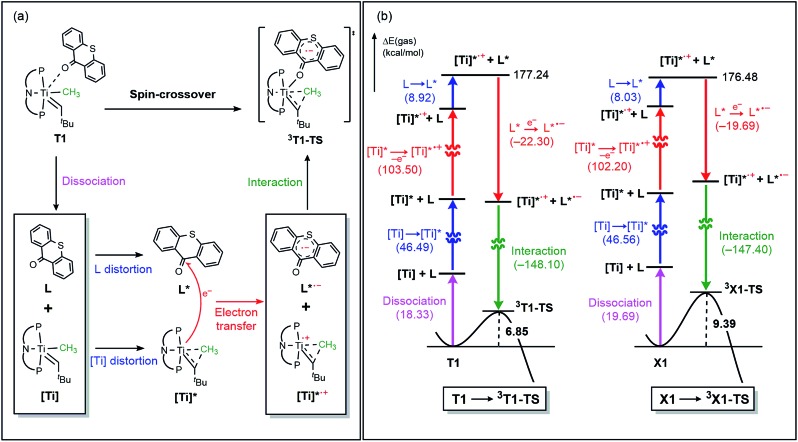
(a) Conceptual steps to reach the transition state of reductive methyl migration. Only thioxanthone case is represented. (b) Electronic energy difference for the dissociation, interaction, distortion, and electron transfer steps.

As mentioned above, the Ti-alkylidene intermediate **1** reacts with xanthone (X) selectively to afford the Wittig-like product ^*t*^BuHC=C_13_H_8_O, by probably forming the putative titanium-oxo complex **W5**, [(PNP)Ti=O(CH_3_)], which is expected to quickly decompose in solution. Mechanistically, the Wittig reaction will likely invoke a [2 + 2] cycloaddition between the alkylidene and the ketone, which may subsequently undergo bond metathesis, as illustrated in Fig. 7 for the thioxanthone (T) substrate. Note that the thioxanthone substrate led to the intriguing methane olefination product, whereas the Wittig-like reaction was minimized with this substrate, but it is apparently maximized with X. Given that the only difference between the two substrates lies in the heteroatom of the carbocycle, oxygen in X and sulfur in T, this chemoselectivity is puzzling. To better understand the origin of this selectivity, we calculated and compared two possible reaction pathways, as represented in Fig. 8 and 9. The methyl migration pathway marked in red in Fig. 8 and 9 is kinetically favored over the Wittig-like reaction when thioxanthone is employed by 1.1 kcal mol^–1^, as the transition state **^3^T1-TS** is lower in energy than **W2-TS**. Curiously, the calculated energy ordering of the two analogous transition states are reversed when X is used and the Wittig-like reaction pathway is predicted to be favored by ∼2 kcal mol^–1^, as shown in Fig. 8b. Thus, our calculations appear to reproduce the experimentally observed chemoselectivity faithfully, but the energy differences are small and they must be interpreted with some caution, as the intrinsic uncertainties of the computational method may not allow for distinguishing energy differences of such small magnitude in a physically meaningful manner.

In order to better understand the origin of the computed energy differences, we first examined the different components of the solution phase free energies and found that the entropy and solvation energy components do not give any meaningful difference. Next, we analyzed the electronic energy differences by deconstructing the total energies into chemically meaningful energy components, as illustrated in Fig. 9. Specifically, we concentrated on the steps **T1** → **^3^T1-TS** and **X1** → **^3^X1-TS**. With T, this transformation is 6.9 kcal mol^–1^ uphill, whereas 9.4 kcal mol^–1^ is found for X. The presence of the sulfur atom in T makes the C–C coupling transition state 2.5 kcal mol^–1^ lower electronically when compared to that of xanthone. This energy difference can be broken down into chemically meaningful components, as shown in Fig. 9. First, starting from the computed structures of **T1** and **X1**, we calculated the “snap dissociation energy” where the (thio)xanthone and the titanaalkylidene fragments are dissociated without each of the fragments being allowed to change their structure, which were 18.3 and 19.7 kcal mol^–1^, respectively. Next, we evaluated the energy required to change the geometry of the Ti-alkylidene fragment to that found in the transition state and they were 46.5 and 46.6 kcal mol^–1^, respectively, marked as [Ti] → [Ti]* in Fig. 9b. As described above, intramolecular electron transfer from the titanium-alkylidene-methyl moiety to the (thio)xanthone ligand takes place when the transition state is reached. Our calculations suggest that the formal oxidation of the Ti fragments require 103.5 and 102.2 kcal mol^–1^, respectively. Lastly, the structural distortion of the T or X ligands was found to by uphill by 8.9 and 8.0 kcal mol^–1^, respectively. Taken together, these energy components add to afford 177.2 and 176.5 kcal mol^–1^, respectively, and represent the energy that must be invested to take the intermediates **T1** and **X1** to the C–C coupling transition states. It is interesting that all the slight differences cancel and the energetic costs are essentially identical with the numerical difference being only 0.6 kcal mol^–1^.

**Fig. 10:**
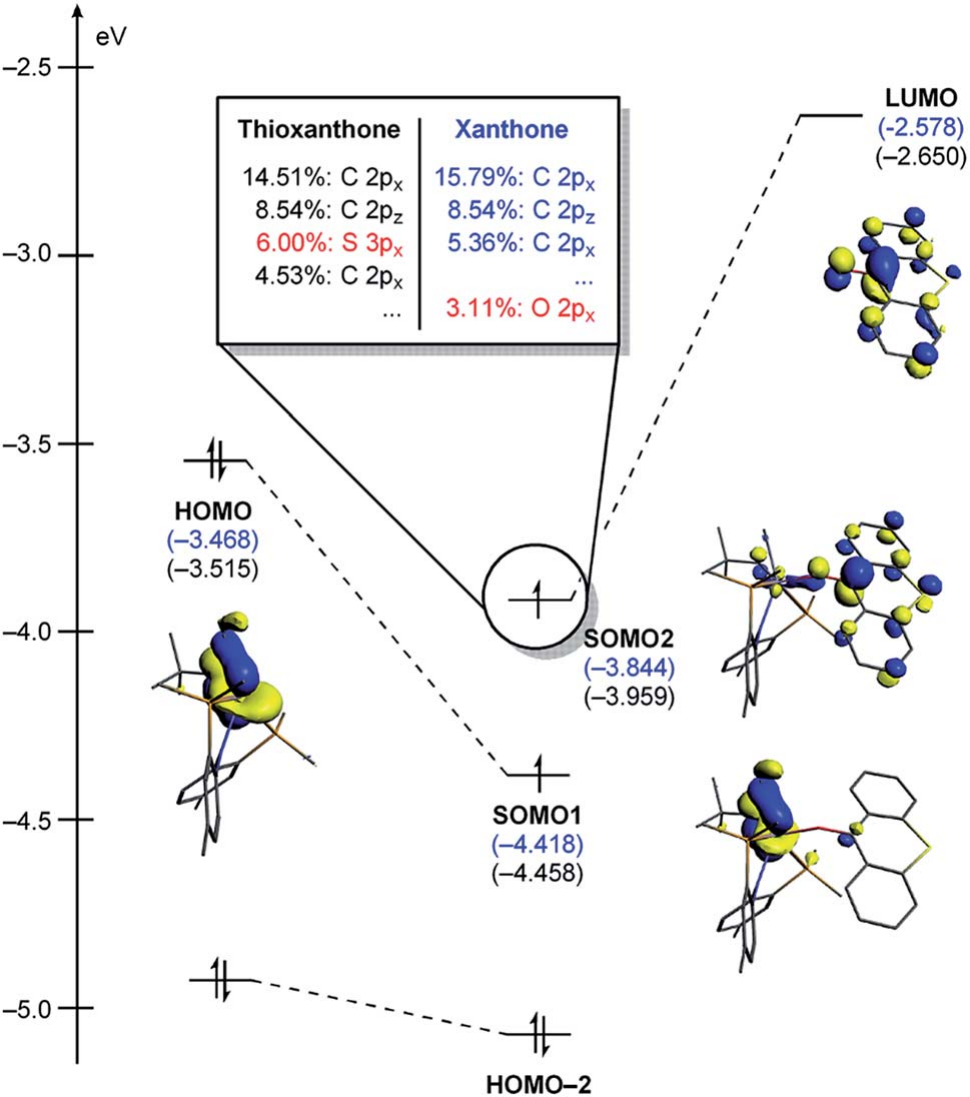
Quantitative perturbation molecular orbital diagrams of **^3^T1-TS** and **^3^X1-TS**. **^3^X1-TS** is represented with the energy values only.

As represented in Fig. 9b, there are two energy terms that are negative in our conceptual analysis. First, the T and X ligands are formally reduced and our calculations show that that process is associated with a gain in electronic energy of –22.3 and –19.7 kcal mol^–1^ for thioxanthone and xanthone, respectively. The interaction energy between the two molecular fragments are computed to be –148.1 and –147.4 kcal mol^–1^, respectively. Obviously, the most important difference in energy stems from the reduction of the T and X substrates, which act as redox-active ligands and temporarily accommodate one electron, as explained above. In Fig. 10, we visualize the LUMO of redox-active ligand and show how the Frontier orbitals evolve, as the Ti(ii)-center becomes formally a Ti(iii)-center with one electron moved to the ligand-based SOMO. Comparing T to X, it is easy to understand the energetic difference discussed above. The contribution of the 3p_*x*_ orbital of sulfur is 6.0%, whereas 2p_*x*_ of oxygen contributes only 3.1%. This result of course reflects on the fact that sulfur is much more polarizable than oxygen and, thus, better in accommodating the excess charge than oxygen would. And whereas this difference is small, it is responsible for lowering the transition state by ∼2.6 kcal mol^–1^ when T is employed compared to when X is used. This energy difference is enough to invert the energetic ordering of the transition states of the olefination and Wittig-like reaction, as illustrated in Fig. 8.

In conclusion, our computational studies highlight how spin-crossover at the metal site and the redox-activity of the substrate work in concert to couple a methyl group to an metalla-alkylidene with a very low barrier. This process leads ultimately to the olefination of methane, and requires the removal of two electrons and two protons overall. We found that T is capable of acting not only as the sacrificial oxidant that becomes reduced during the process to form TH^–^, but participates actively in the C–C coupling by acting as a reservoir of the excess electron density at the transition state. The structurally related X substrate is incapable of promoting the C–C coupling, because the lack of the sulfur heteroatom reduces its ability to accommodate the excess electron thus resulting in a higher transition state of ∼2.6 kcal mol^–1^. The carbonyl functionality present in the substrate gives rise to a competing reaction channel, where a [2 + 2] cycloaddition may afford a Wittig-like product. Our calculations show that the barriers of this reaction are very similar to those of the C–C coupling reactions, especially in the case of T. Such an additional redox stabilization of the T ligand can push the reaction to be more selective for C–C coupling, whereas in X only the [2 + 2] cycloaddition product is observed.

**Fig. 11:**
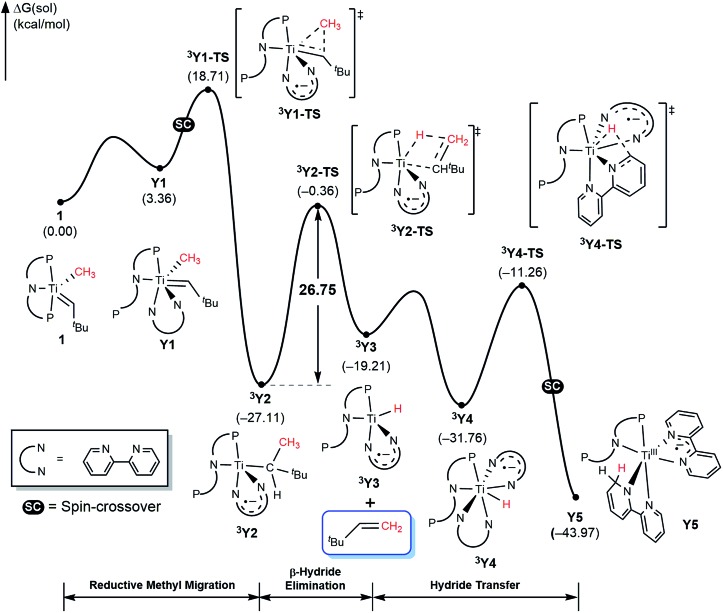
Free energy profile for formation of H_2_C=CH^*t*^Bu using bipy.

Given the detailed insights about the mechanism summarized above for T and X, bipy substrate offers several interesting features that will enhance our understanding of the mechanism. First, the lack of the carbonyl functionality prevents Wittig-like chemistry. Second, the bipy ligand is widely known to be redox active and we anticipate the that it too may act as an electron reservoir just like thioxanthone and, third, we expect that the lack of flexibility in structure and binding geometry will limit the possible spin-crossover scenarios. Thus, we examined the mechanism of methane olefination using bipy in detail and the computed reaction energy profile is shown in Fig. 11. The general features of the mechanism are overall similar to what was seen with the T and X systems, but there are some subtle but important differences. As noted previously, the reaction is initiated by coordination of auxiliary substrate bipy to **1** to form **Y1**, where one phosphine arm of PNP ligand must dissociate to accommodate the chelating nature of the bipy ligand. In the C–C coupling transition state **^3^Y2-TS** the bipy functionality acts as an electron-reservoir and becomes formally reduced, confirming our expectation that bipy is a competent redox-active ligand. Unlike what was observed previously, the C–H activation is accomplished *via* a classical β-hydride elimination mechanism when the transition state **^3^Y2-TS** is traversed with a calculated barrier of 26.8 kcal mol^–1^, which produces the olefin product H_2_C=CH^*t*^Bu. This barrier is slightly higher than the ∼25 kcal mol^–1^ observed for T and is in good agreement with the observation that with bipy, the reaction is slower. Once **^3^Y3** is formed, it may bind another equivalent of bipy to afford **^3^Y4**. Finally, the hydride migrates to the newly added bipy functionality traversing the transition state **^3^Y4-TS**, and a spin-crossover to the singlet surface affords the diamagnetic final product complex **Y6**. Thus, the bipy substrate behaves as expected based on the general mechanistic understanding and is capable of promoting the methane olefination reaction, albeit with a slightly higher barrier and with minimally different mechanistic features that were easy to be anticipated.

## Conclusions

We have shown how CH_4_ can be activated by (PNP)Ti=CH^*t*^Bu(CH_2_^*t*^Bu) *via* transient titanium alkylidyne intermediate at room temperature to generate (PNP)Ti=CH^*t*^Bu(CH_3_) and how methyl migration can be promoted with exogenous redox-active ligands to ultimately yield the dehydrocoupled product H_2_C=CH^*t*^Bu. In addition, whereas thioxanthone resulted in the formation of H_2_C=CH^*t*^Bu *via* reductive C–C coupling, the xanthone ligand only produced the olefin from a Wittig-like reaction. By combining computational and experimental methods of mechanistic inquiry, we revealed a complete pathway that unifies experimental observations and computational results. We found that the critical role of thioxanthone and bipyridine is to become redox active during the course of the reaction and accommodate an electron to enable reductive methyl migration to form a C–C bond and ultimately H_2_C=CH^*t*^Bu. The methane olefination is therefore facilitated by the redox-active ligand which acts as an electron reservoir to avoid a filled–filled interaction between the two Lewis basic fragments in the putative singlet transition state. As the C–C coupling takes place, one electron from the Ti-alkylidene π-orbital is removed and placed in the π*-orbital of the redox-active ligand. Experimentally observed chemoselectivity between thioxanthone and xanthone was also scrutinized and explained using fragment analysis. The resulting C–C coupled product then forms the olefin H_2_C=CH^*t*^Bu by hydride elimination. In the case of thioxanthone, β-hydrogen abstraction promotes olefin formation whereas for bipy, the more classical β-hydride elimination ensues. Several spin-crossover events are proposed along the reaction trajectory that helps to lower the energies of intermediates and transition states. Our strategy therefore provides a mild route to make C–C bonds with methane using an electropositive base metal that generally do not engage in two-electron redox processes.

## Conflicts of interest

There are no conflicts to declare.

## Supplementary Material

Supplementary information

Crystal structure data
