# Supplementary material for: Room temperature olefination of methane with titanium–carbon multiple bonds
Source: Chem Sci. 2018 Feb 13;9(13):3376–85. doi: 10.1039/c7sc05238c (PMC5933228; doi:10.1039/c7sc05238c)
Supplement: Supplementary file 1 [file SC-009-C7SC05238C-s001.pdf]

Electronic Supplementary Material (ESI) for Chemical Science.
This journal is © The Royal Society of Chemistry 2018

Room Temperature Olefination of Methane with a Titanium Alkylidene.

Takashi Kurogi,[a,//] Joonghee Won,[b,c,//] Bohyun Park,[b,c] Oleksandra S. Trofymchuk,[a] Patrick J. Carroll,[a] Mu-Hyun Baik,[b,c,*] and Daniel J. Mindiola[a,*]

[a]*Department of Chemistry, University of Pennsylvania, Philadelphia, PA 19104, USA.*
[b]*Department of Chemistry, Korea Advanced Institute of Science and Technology (KAIST), Daejeon 34141, Republic of Korea.*
[c]*Center for Catalytic Hydrocarbon Functionalizations, Institute for Basic Science (IBS), Daejeon 34141, Republic of Korea*
[//]*These authors contributed equally*

Table of Contents

**General Procedure**	S3
**Synthesis of [(PNP)Ti(OCH($C_{12}H_8S$)(η^2-OC($C_{12}H_8S$)] (2)**	S4-S5
**Synthesis of [(PNP)Ti(bipy)(bipyH)] (3)**	S5-S6
**NMR Spectroscopic Data**	
Complex **2**	S7-S9
Complex **3**	S9-S11
**Reaction of 1 with Thioxanthone**	S12-S14
**Reaction of 1 with Xanthone**	S14-S15
**Reaction of 1 with 2,2'-Bipyridine**	S16-S17
**Reaction of 1 with PMe_3**	S18-S19
**Reaction of 1 with Pyridine**	S19-S20
**Reaction of (PNP)Ti(=CH^{t}Bu)(CH$_2$^{t}Bu) with Thioxanthone**	S21-S22
**Reaction of 1-D_3 with Thioxanthone**	S22-S25
**Reaction of 1-D_3 with 2,2'-Bipyridine**	S25-S26
**Reaction of 1-^{13}C with Thioxanthone**	S26-S29
**Reaction of 1-^{13}C with 2,2'-Bipyridine**	S29-S31
**GC-MS Analyses**	S32-S34

**FT-IR**	S35
**UV-Vis**	S35
**X-ray Crystallography**	S36
Crystallographic data of **2**	S37
Crystallographic data of **3**	S38
**Computational Details**	S39-S44
**Total Energy Data for the Calculated Structures**	S45-S48
**Cartesian Coordinates of the Optimized Geometries**	S49-S88
**Vibration Frequencies of the Optimized Structures**	S89-S108
**References**	S109-S110

General Procedure

All operations were performed in an M. Braun glove box or using standard Schlenk techniques under a nitrogen atmosphere unless otherwise stated. Anhydrous hydrocarbon solvents were purchased from Fisher Scientific. All anhydrous hydrocarbon solvents (pentane, hexane, toluene, benzene) were purified and dried by passage through two columns of activated alumina and Q-5 drying agent in a Grubbs-type solvent system. Stabilizer-free ethereal solvents (Et_2O and THF) were purchased from Alfa Aesar and dried by passage through two columns of activated alumina. All bulk solvents were kept over sodium and 4 Å molecular sieves. Benzene-d_6 (Cambridge Isotope Laboratories) was dried and degassed over a potassium mirror prior to use. Celite and 4 Å molecular sieves were activated under vacuum overnight at 200 °C. (PNP)Ti(=CH^{t}Bu)(CH$_2$^{t}Bu),[1] (PNP)Ti(=CH^{t}Bu)(CH_3) (**1**),[2] and (PNP)Ti(=CH^{t}Bu)(CD_3) (**1-D$_3$**)[2] were prepared according to the reported procedures. (PNP)Ti(=CH^{t}Bu)($^{13}CH_3$) (**1-^{13}C**) was prepared by following the reported procedure of **1**[2] with Mg($^{13}CH_3$)$_2$. Mg($^{13}CH_3$)$_2$ was prepared from $^{13}CH_3I$ (Sigma-Aldrich, 99 atom % ^{13}C) and Mg (turnings) in Et_2O with addition of 1,4-dioxane. All other chemicals were purchased from commercial sources and degassed before being used. ^1H, ^2H, ^{13}C, ^{31}P, HSQC and COSY NMR spectra were recorded on a Bruker AV-II 500 MHz, DRX 500 MHz or AV-III 400 MHz spectrometers. ^1H, ^2H and ^{13}C NMR chemical shifts are reported referenced to the internal residual proton, deuterium or carbon resonances of C_6D_6 (δ = 7.16 ppm or 128.06 ppm). ^{31}P NMR chemical shifts are reported with respect to external H_3PO_4 (δ 0.0 ppm). The IR samples were prepared by a KBr plate method (JASCO Tablet Master) and the IR spectrum was obtained on a JASCO FT/IR-4600 spectrometer. The UV-Vis absorption spectrum was obtained on a Cary 5000 UV-Vis-NIR spectrophotometer (Agilent Technologies). Elemental analyses were performed at a Euro EA 3000 (Euro Vector) and by Midwest Microlab, Inc.

Synthesis of [(PNP)Ti(OCH($C_{12}H_8S$)(η^2-OC($C_{12}H_8S$)] (2)

To a yellowish brown solution of **1** (100 mg, 178 μmol) in benzene (5 mL) was added thioxanthone (67.1 mg, 357 μmol) suspended in benzene (3 mL). The reaction mixture was stirred for 24 hours at room temperature and changed in color to reddish brown. The reaction mixture was evaporated to dryness and the brown residue was washed with pentane (5 mL). The resulted brown solid was suspended in THF (2 mL) and filtered through glass wool in a pipette. The brown filtrate was layered with pentane (5 mL) and stored at –35 °C overnight to yield **2**·0.5(THF) as a reddish brown powder (87.1 mg, 92.9 μmol, 52% yield). The amount of co-crystallized THF has been determined by ^{1}H NMR. Brown crystals suitable for X-ray analysis were grown from concentrated toluene/hexane solution at −35 °C.

^{1}H NMR (400 MHz, Benzene-d_6, 300 K): δ 7.38 (d, $^3J_{HH}$ = 8 Hz, 1H, Ar-C*H*), 7.36 (dd, $^3J_{HH}$ = 8 Hz, $^5J_{HH}$ = 2 Hz, 1H, Ar-C*H*), 7.30 (d, $^3J_{HH}$ = 8 Hz, 1H, Ar-C*H*), 7.29-7.23 (overlapped, 2H, Ar-C*H*), 7.16 (overlapped with C_6D_5H, 2H, Ar-C*H*), 7.10 (d, $^3J_{HH}$ = 7 Hz, 1H, Ar-C*H*), 6.96-6.86 (overlapped, 6H, Ar-C*H* + OC*H*), 6.82-6.76 (overlapped, 4H, Ar-C*H*), 6.70-6.63 (overlapped, 4H, Ar-C*H*), 6.41 (virtual t, J_{HH} = 5 Hz, 1H, Ar-C*H*), 3.58 (br, 2H, THF), 3.48 (m, 1H, PC*H*(CH_3)$_2$), 2.71 (m, 1H, PC*H*(CH_3)$_2$), 2.34 (m, 1H, PC*H*(CH_3)$_2$), 2.21 (s, 3H, Ar-CH_3), 2.08 (s, 3H, Ar-CH_3), 1.80 (dd, $^3J_{HH}$ = 7 Hz, $^3J_{HP}$ = 15 Hz, 3H, PCH(CH_3)$_2$), 1.65 (m, 1H, PC*H*(CH_3)$_2$), 1.42 (br, 2H, THF), 1.33 (dd, $^3J_{HH}$ = 7 Hz, $^3J_{HP}$ = 16 Hz, 3H, PCH(CH_3)$_2$), 0.92-0.85 (overlapped, 6H, PCH(CH_3)$_2$), 0.72-0.62 (overlapped, 6H, PCH(CH_3)$_2$), 0.45 (dd, $^3J_{HH}$ = 7 Hz, $^3J_{HP}$ = 16 Hz, 3H, PCH(CH_3)$_2$). ^{13}C{^{1}H} NMR (126 MHz, Benzene-d_6, 300 K) δ 159.57 (Ar), 155.83 (Ar), 141.62 (Ar), 141.17 (Ar), 140.20 (Ar), 132.84 (Ar), 132.61 (Ar), 132.55 (Ar), 132.47 (Ar), 132.24 (Ar), 132.15 (Ar), 131.65 (Ar), 131.33 (Ar), 127.22 (Ar), 127.04 (Ar), 126.94 (Ar), 126.76 (Ar), 126.57 (Ar), 126.50 (Ar), 126.37 (Ar), 126.16 (Ar), 126.02 (Ar), 125.86 (Ar), 124.77 (Ar), 124.48 (Ar), 121.63 (Ar), 120.89 (Ar), 114.51 (Ar), 83.78 (O*C*H), 26.01 (br, 2C, P*C*H(CH_3)$_2$), 25.52 (THF), 22.96 (d, $^2J_{CP}$ = 6 Hz, PCH(*C*H_3)$_2$), 22.15 (d, $^2J_{CP}$ = 5 Hz, PCH(*C*H_3)$_2$), 21.08 (Ar*C*H_3), 20.91 (d, $^2J_{CP}$ = 12 Hz, PCH(*C*H_3)$_2$), 20.56 (Ar*C*H_3), 20.23 (d, $^2J_{CP}$ = 8 Hz, PCH(*C*H_3)$_2$), 19.82 (d,

$^2J_{CP}$ =6 Hz, PCH(CH_3)$_2$), 18.70 (d, $^2J_{CP}$ = 12 Hz, PCH(CH_3)$_2$), 17.80 (d, $^2J_{CP}$ = 4 Hz, PCH(CH_3)$_2$), 16.14 (d, $^2J_{CP}$ = 8 Hz, PCH(CH_3)$_2$). Selected ^{1}H-^{13}C HSQC NMR chemical shifts (500 MHz, Benzene-d_6, 300 K): δ^1H (δ^{13}C) 6.88 (83.8). ^{31}P{^{1}H} NMR (162 MHz, Benzene-d_6, 300 K) δ 26.03 (d, $^2J_{PP}$ = 36 Hz, PNP), 15.48 (br d, $^2J_{CP}$ = 36 Hz, PNP). IR (cm^{-1}; KBr): 3055, 2952, 2925, 2868, 1643, 1588, 1556, 1464, 1386, 1364, 1314, 1278, 1227, 1201, 1134, 1114, 1057, 1032, 930, 882, 859, 817, 770, 755, 734, 709, 683, 661, 650, 630, 621, 573, 554, 530, 495, 465, 444, 403, 337, 369, 352. Anal. Calcd. for $C_{54}H_{61}NO_{2.5}P_2S_2Ti$ (**2**·0.5(THF)): C, 69.14; H, 6.56; N, 1.49; S, 6.84. Found: C, 66.73; H, 6.35; N, 1.41; S, 6.54. The elemental analysis data shows a low carbon value, but good agreement with the calculated hydrogen, nitrogen, and sulfur values. We attribute the low carbon identification to incomplete combustion resulting in formation of metal-carbide species.

Synthesis of [(PNP)Ti(bipy)(bipyH)] (3)

To a yellowish brown solution of **1** (250 mg, 445 μmol) in benzene (10 mL) was added 2,2'-bipyridine (264 mg, 1.45 mmol) in benzene (5 mL) at room temperature. The reaction mixture was stirred at room temperature for 24 hours and changed in color to violet. The reaction mixture was filtered through glass wool in a pipette and the filtrate was evaporated to dryness under reduced pressure. The purple residue was dissolved in THF (3 mL) and filtered through glass wool in a pipette again. The filtrate was layer with pentane (5 mL) and stored at –35 °C to yield **3**·bipy as a violet crystalline solid (141 mg, 146 μmol, 33% yield). Violet crystals suitable for X-ray analysis were grown from concentrated THF solution at −35 °C. The amount of co-crystallized bipy has been determined by X-ray analysis. The ^{1}H and ^{13}C NMR spectra in benzene-d_6 show excessive bipy resonances due to insolubility of **3** in benzene-d_6 after crystallization with bipy. However, the elemental analysis data shows good agreement with the calculated values of **3**·bipy.

^{1}H NMR (400 MHz, Benzene-d_6, 300 K): δ 8.74 (d, $^3J_{HH}$ = 8 Hz, free bipy), 8.64 (d, $^3J_{HH}$ = 6 Hz, 1H, bipyH-C*H*), 7.33 (br, 2H, Ar-C*H*), 7.21 (virtual t, $^3J_{HH}$ = 8 Hz, free

bipy), 7.25-7.15 (overlapped with free bipy and C_6D_5H, 8H, bipy + Ar-C*H*), 7.01 (m, 1H, bipyH-C*H*), 6.90 (d, $^3J_{HH}$ = 7 Hz, 1H, bipy-C*H*), 6.89 (dd, $^3J_{HH}$ = 6 Hz, 8Hz, free bipy), 6.95-6.70 (overlapped with free bipy, 5H, bipyH + Ar-C*H*), 6.02 (d, $^3J_{HH}$ = 9 Hz, 1H, bipy-C*H*), 5.87 (d, $^3J_{HH}$ = 9 Hz, 1H, bipyH-C*H*), 5.31 (t, $^3J_{HH}$ = 7 Hz, 1H, bipy-C*H*), 4.92 (t, $^3J_{HH}$ = 6 Hz, 1H, bipy-C*H*), 4.83 (t, $^3J_{HH}$ = 6 Hz, 1H, bipyH-C*H*), 4.15 (t, $^3J_{HH}$ = 6 Hz, 1H, bipyH-C*H*), 3.41 (br, 2H, PC*H*(CH_3)$_2$), 2.95 (br, 2H, PC*H*(CH_3)$_2$), 2.13 (br s, 6H, Ar-C*H*$_3$), 2.00-0.90 (overlapped, 24H, PCH(C*H*$_3$)$_2$). $^{13}C\{^1H\}$ NMR (126 MHz, Benzene-*d*$_6$, 300 K) δ 156.74 (free bipy), 152.02 (bipyH), 149.36 (free bipy), 144.69 (Ar), 136.61 (free bipy), 136-132 (br, Ar), 123.68 (free bipy), 122.50 (bipy), 121.72 (bipyH), 121.15 (free bipy), 119.93 (bipyH), 119.41 (bipyH), 107.95 (bipy), 106.71 (bipyH), 24.49 (br, 4C, P*C*H(CH_3)$_2$). 20.82 (br, 8C, PCH(*C*H$_3$)$_2$), 20.45 (2C, Ar*C*H$_3$). Fluxional behavior in solution and insolubility prevented us from measuring aromatic ^{13}C signals in the PNP and bipy ligands. Selected ^1H-^{13}C HSQC NMR chemical shifts (500 MHz, Benzene-*d*$_6$, 300 K): δ ^1H (δ ^{13}C) 8.64 (152.02), 6.02 (119.41), 5.87 (119.93), 5.31 (122.50), 4.92 (107.95), 4.83 (121.72), 4.15 (106.71). $^{31}P\{^1H\}$ NMR (162 MHz, Benzene-*d*$_6$, 300 K) δ 54.98 (br, PNP), 53.05 (br, PNP). UV-Vis (THF, λ_{max}/nm (ε/M^{-1} cm^{-1})): 261 (27000), 306 (22800), 565 (8370). Anal. Calcd. for $C_{56}H_{65}N_7P_2Ti$ (**3**·bipy): C, 71.10; H, 6.93; N, 10.36. Found: C, 70.81; H, 7.10; N, 10.47.

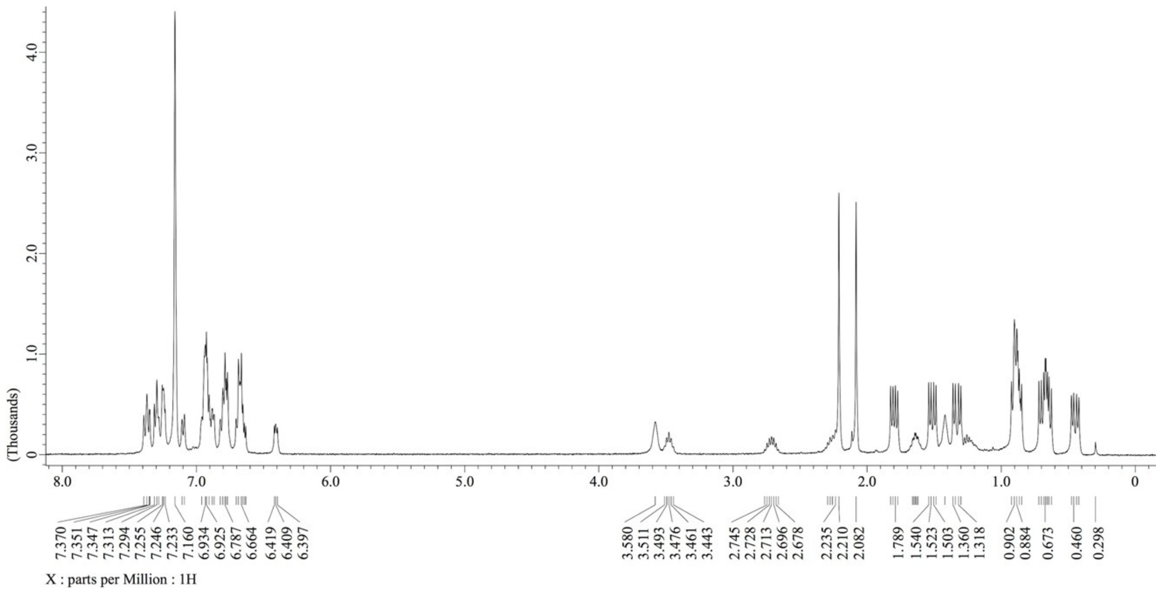

Figure S1. ^{1}H NMR spectrum of **2** (400 MHz, in Benzene-d_6, at 300 K).

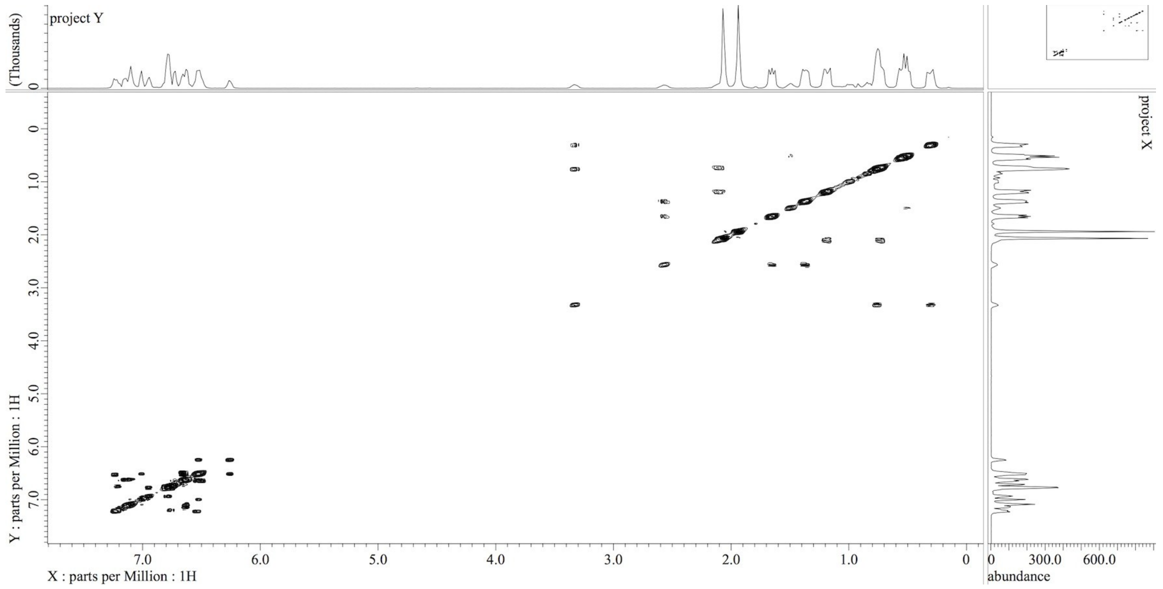

Figure S2. ^{1}H-^{1}H COSY NMR spectrum of **2** (500 MHz, in Benzene-d_6, at 300 K).

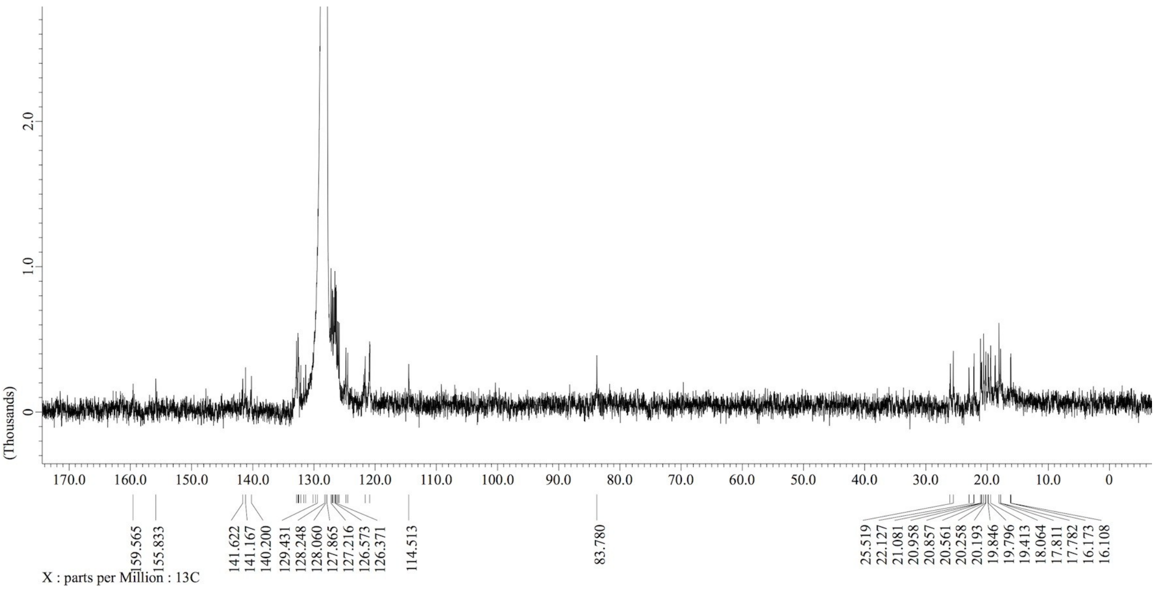

Figure S3. $^{13}C\{^{1}H\}$ NMR spectrum of **2** (126 MHz, in Benzene-d_6, at 300 K).

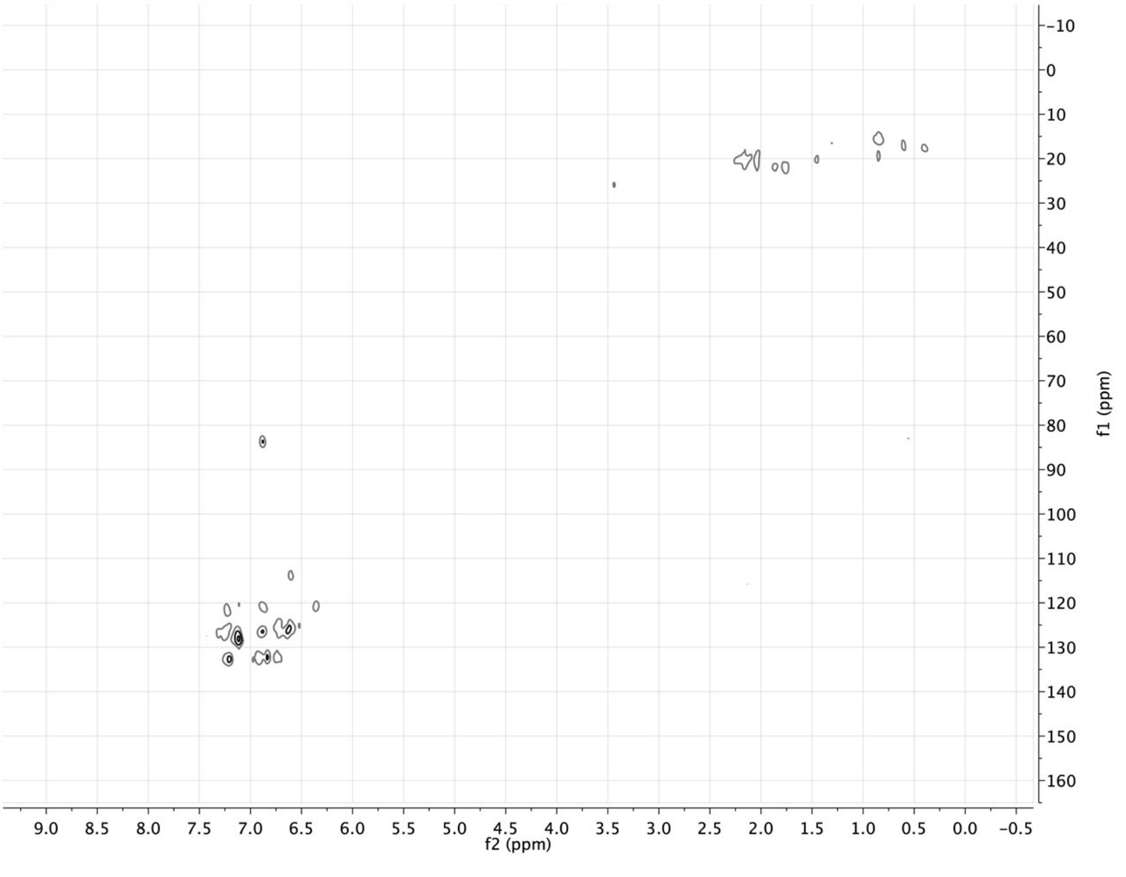

Figure S4. ^{1}H-^{13}C HSQC spectrum of **2** (500 MHz, in Benzene-d_6, at 300 K).

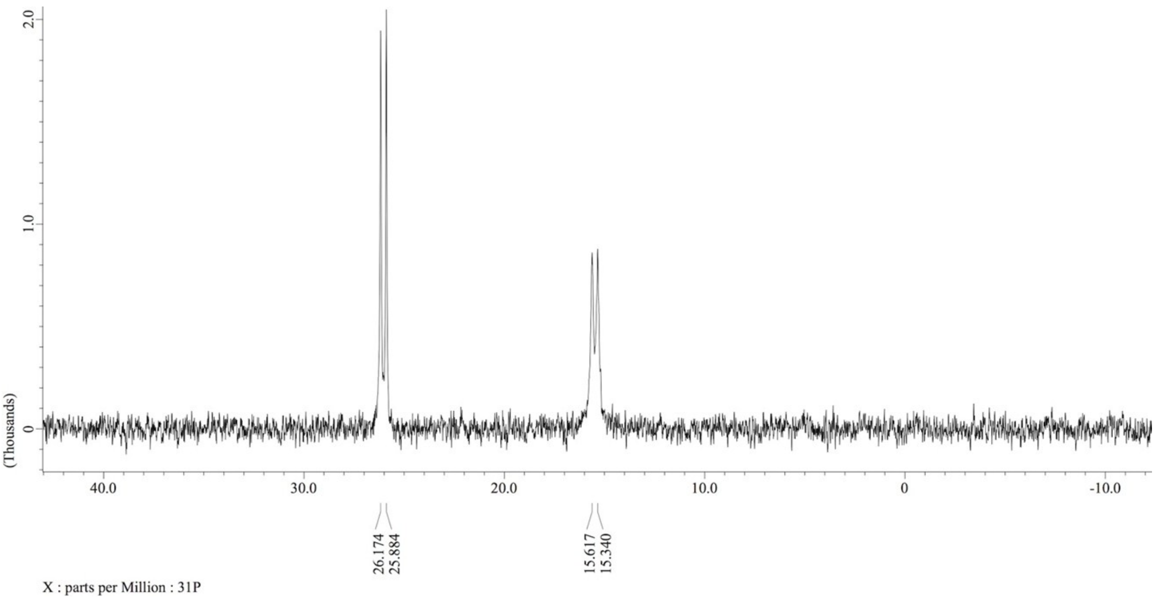

Figure S5. $^{31}P\{^{1}H\}$ NMR spectrum of **2** (162 MHz, in Benzene-d_6, at 300 K).

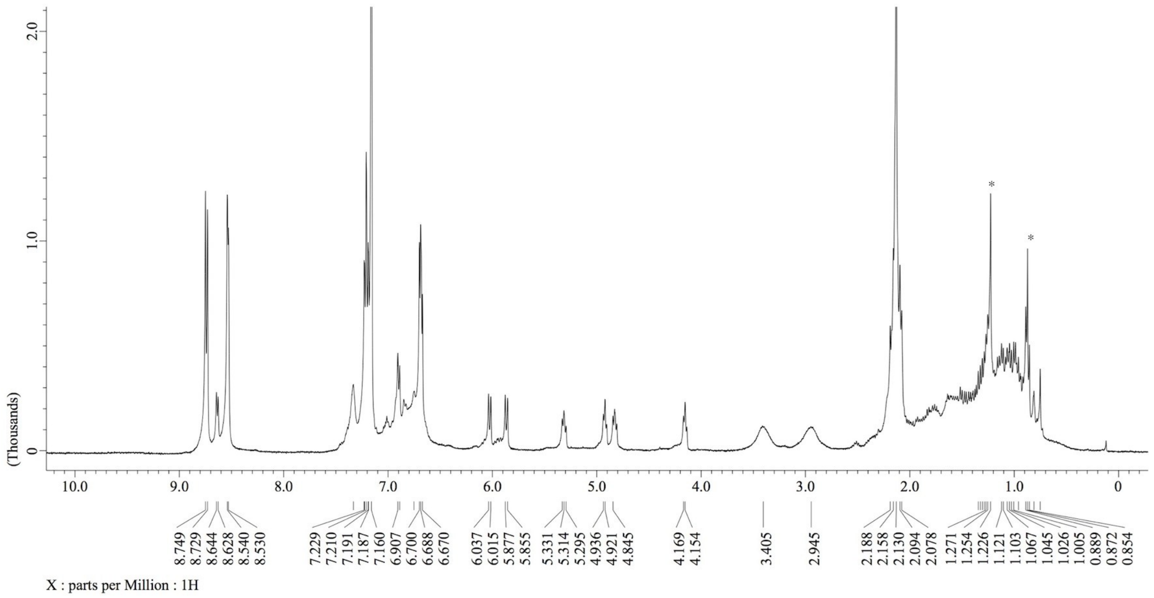

Figure S6. ^{1}H NMR spectrum of **3** (400 MHz, in Benzene-d_6, at 300 K). *: pentane.

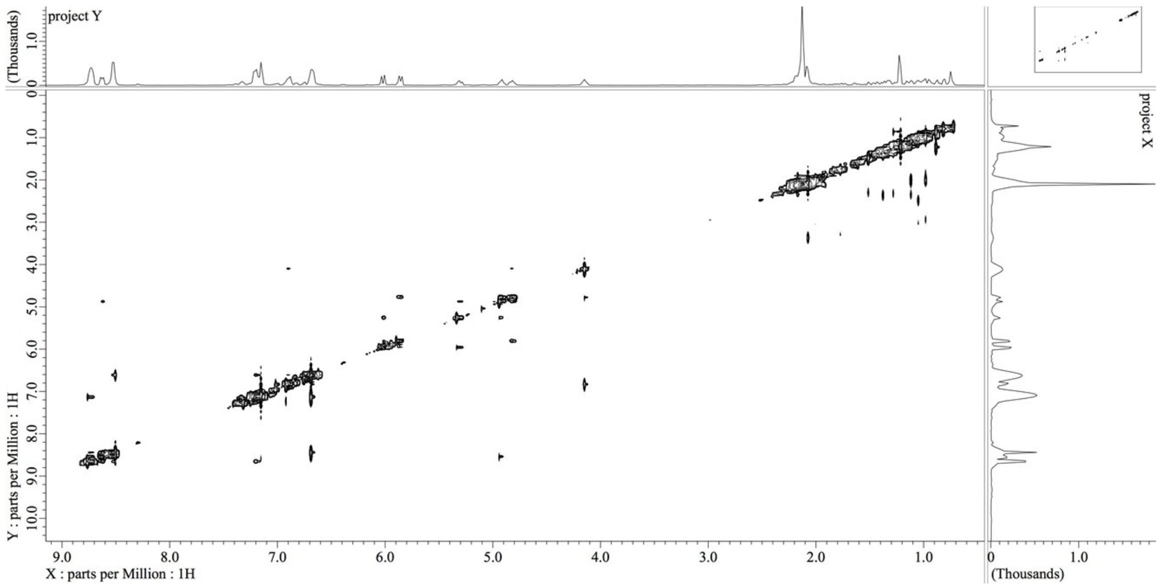

Figure S7. ^{1}H-^{1}H COSY NMR spectrum of **3** (500 MHz, in Benzene-d_6, at 300 K).

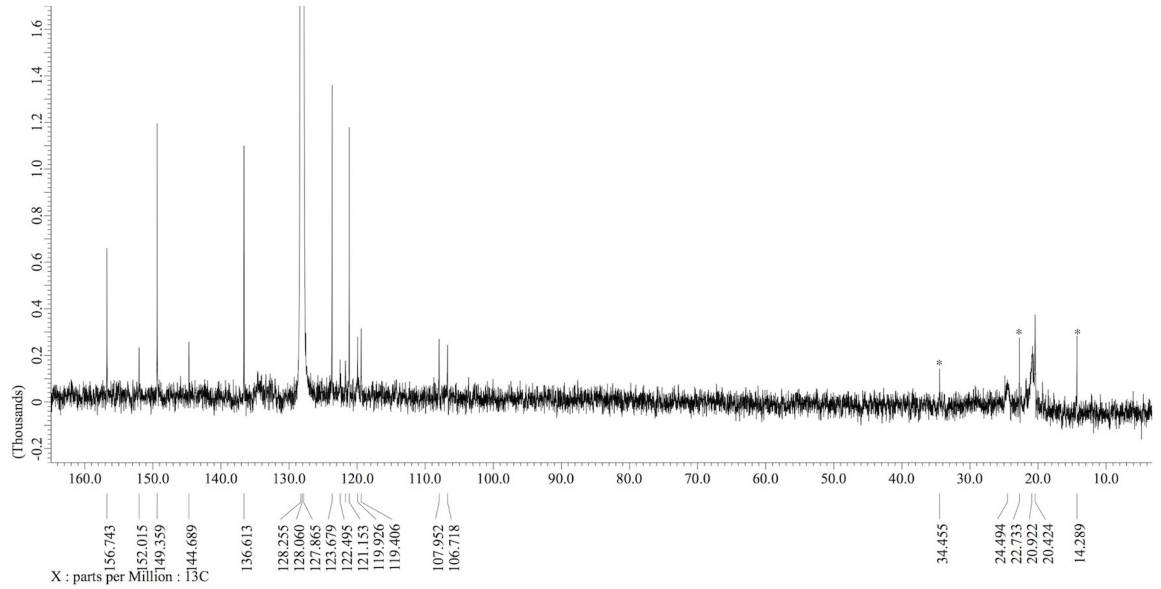

Figure S8. $^{13}C\{^{1}H\}$ NMR spectrum of **3** (126 MHz, in Benzene-d_6, at 300 K).

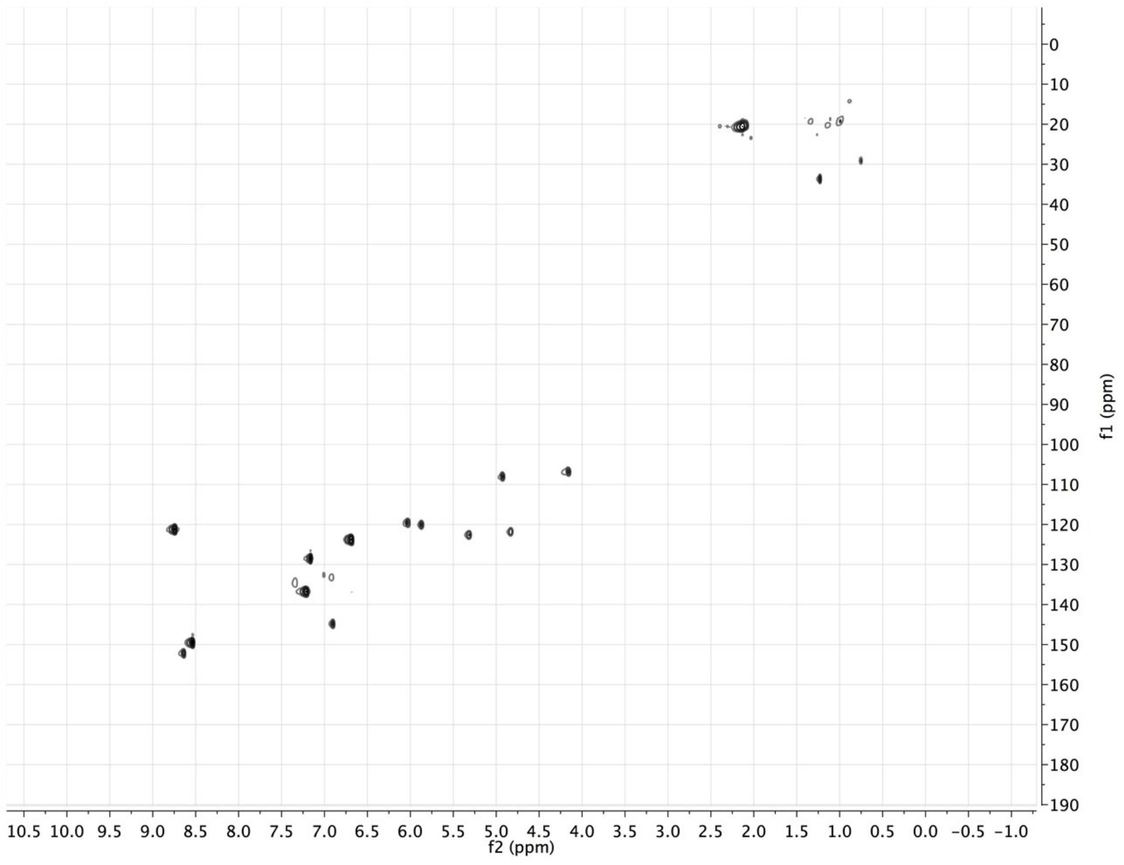

Figure S9. ^{1}H-^{13}C HSQC spectrum of **3** (500 MHz, in Benzene-d_6, at 300 K).

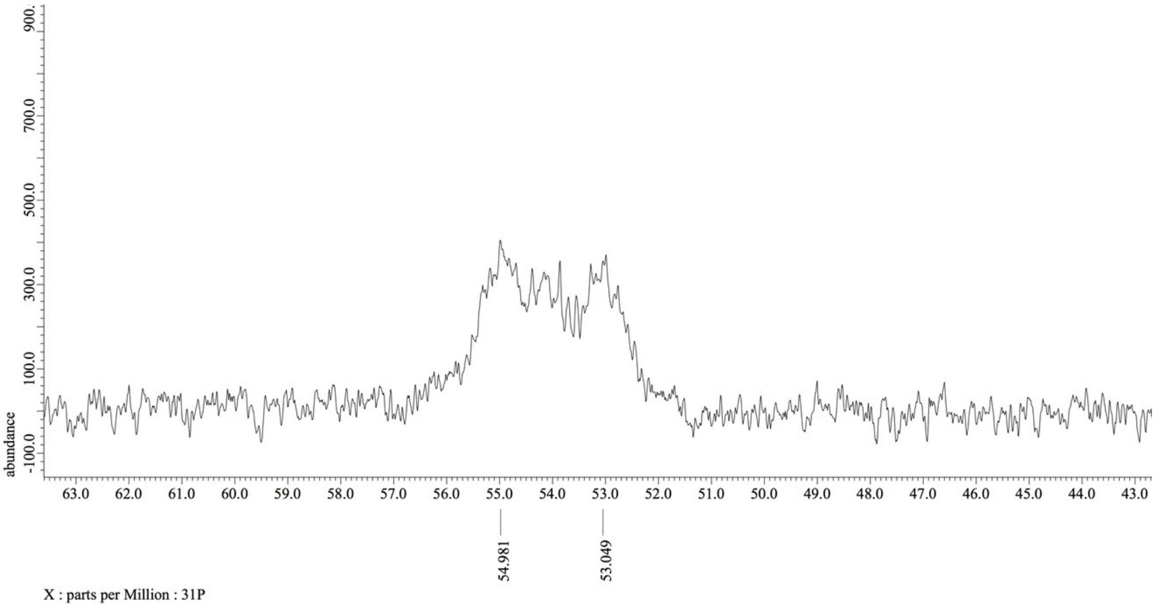

Figure S10. ^{31}P{^{1}H} NMR spectrum of **3** (162 MHz, in Benzene-d_6, at 300 K).

Reaction of 1 with Thioxanthone

In a J-Young valve NMR tube, a benzene-d_6 solution (0.5 mL) of **1** (16.1 mg, 28.7 μmol) was treated with thioxanthone (11.1 mg, 60.9 μmol) at room temperature. The reaction mixture gradually changed in color from yellowish brown to reddish brown over 18 hours at room temperature. Formation of **2**, $H_2C{=}CH^tBu$ and $^tBuHC{=}C(C_{12}H_8S)$ was observed by ^1H NMR (Figure S11 and S12). The ^{31}P NMR spectrum also showed formation of **2** and unidentified products from decomposition of "(PNP)Ti=O(CH$_3$)" (Figure S13). The NMR tube was connected the distillation apparatus and all volatile materials were vacuum-transferred into another J-Young valve NMR tube. The ^1H NMR spectrum of the transferred sample showed only $H_2C{=}CH^tBu$ (Figure S14), except for trace solvents originally in **1**. The brown residue in the original NMR tube was dissolved in toluene (0.5 mL) and quenched by MeOH, and filtered through short plug of silica gel. The filtrate and the transferred $H_2C{=}CH^tBu$ sample were used for GC-MS analyses, described below.

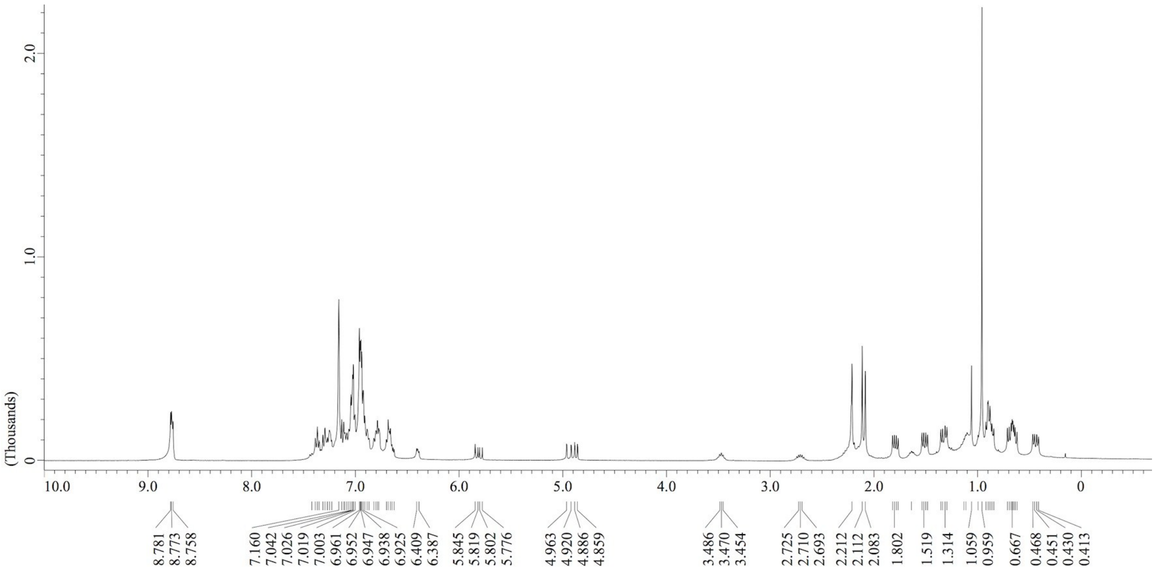

Figure S11. ^1H NMR spectrum of **1** with thioxanthone (400 MHz, in Benzene-d_6, at 300 K).

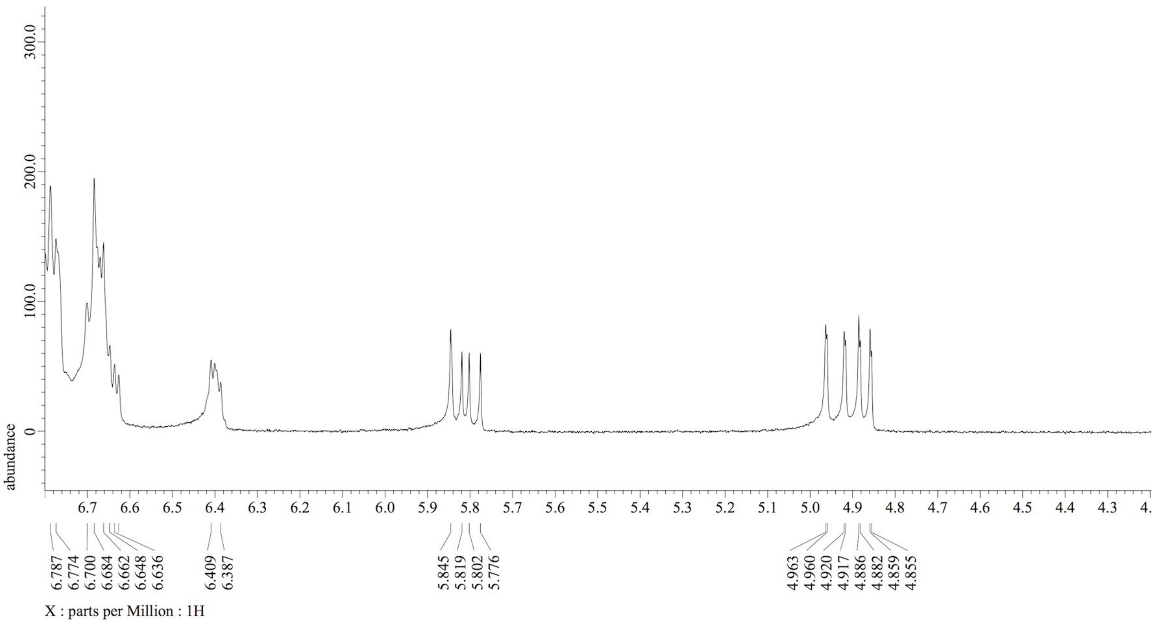

Figure S12. Expanded ^{1}H NMR spectrum around olefinic region of **1** with thioxanthone (400 MHz, in Benzene-d_6, at 300 K).

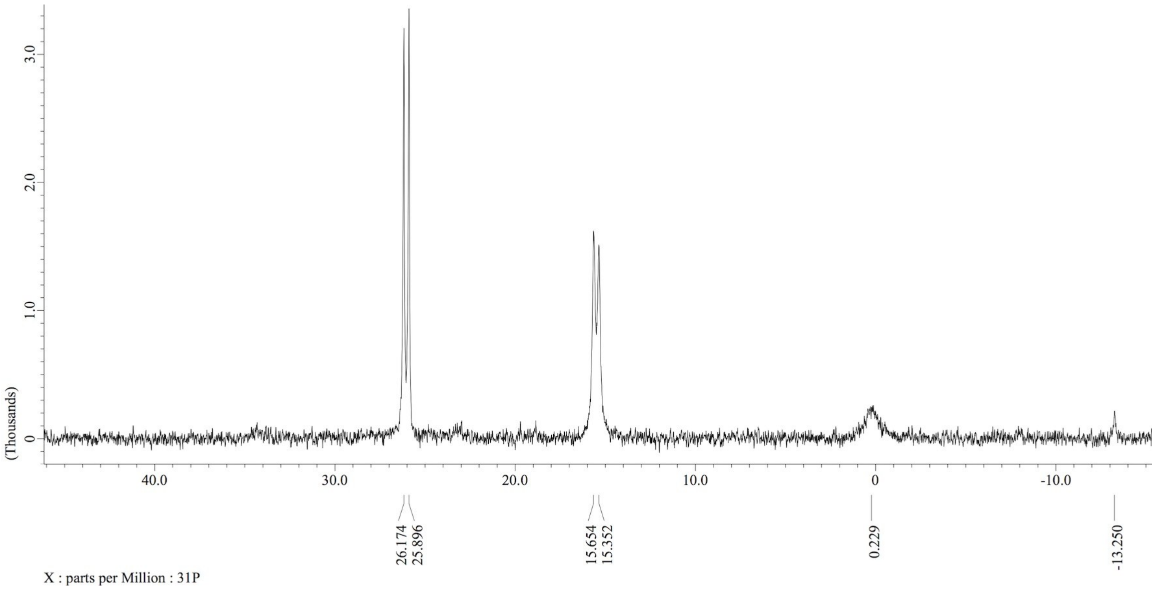

Figure S13. $^{31}P\{^{1}H\}$ NMR spectrum of **1** with thioxanthone (162 MHz, in Benzene-d_6, at 300 K).

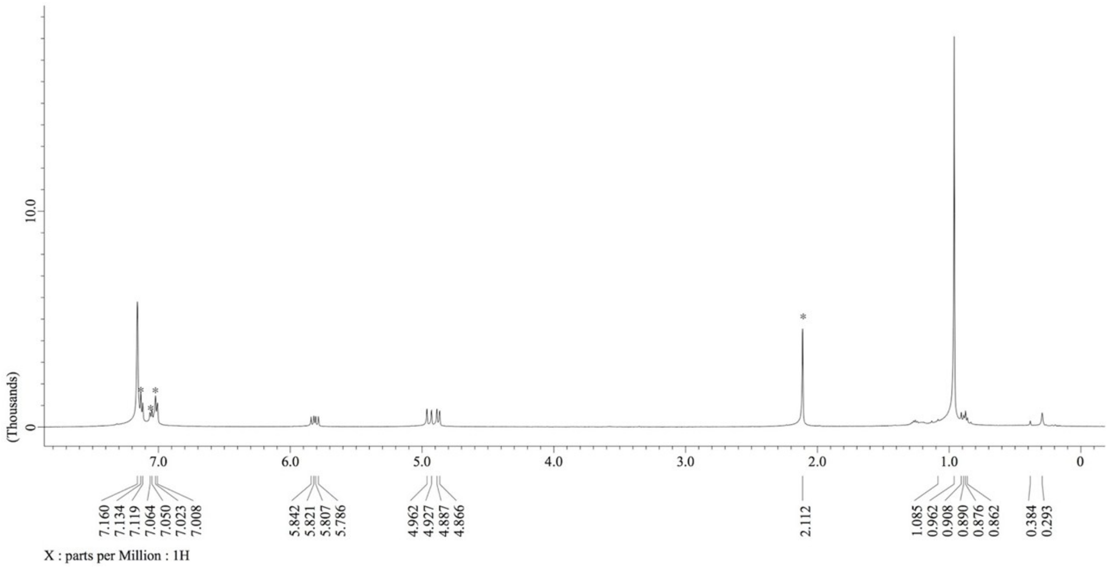

Figure S14. ^{1}H NMR spectrum of volatile materials from **1** with thioxanthone (500 MHz, in Benzene-d_6, at 300 K).*: toluene.

Reaction of 1 with Xanthone

In a J-Young valve NMR tube, a benzene-d_6 solution (0.5 mL) of **1** (10.7 mg, 19.1 μmol) was treated with xanthone (7.5 mg, 38.2 μmol) at room temperature. The reaction mixture changed in color from yellowish brown to reddish brown over 4 hours at room temperature. Formation of only ^{t}BuHC=C($C_{12}H_8O$) and unidentified PNP products was observed by ^{1}H NMR (Figure S15). The ^{31}P NMR spectrum also showed formation of unidentified products from decomposition of "(PNP)Ti=O(CH_3)" (Figure S16). The reaction mixture was quenched by MeOH, and filtered through short plug of silica gel. The filtrate was used for GC-MS analysis, described below.

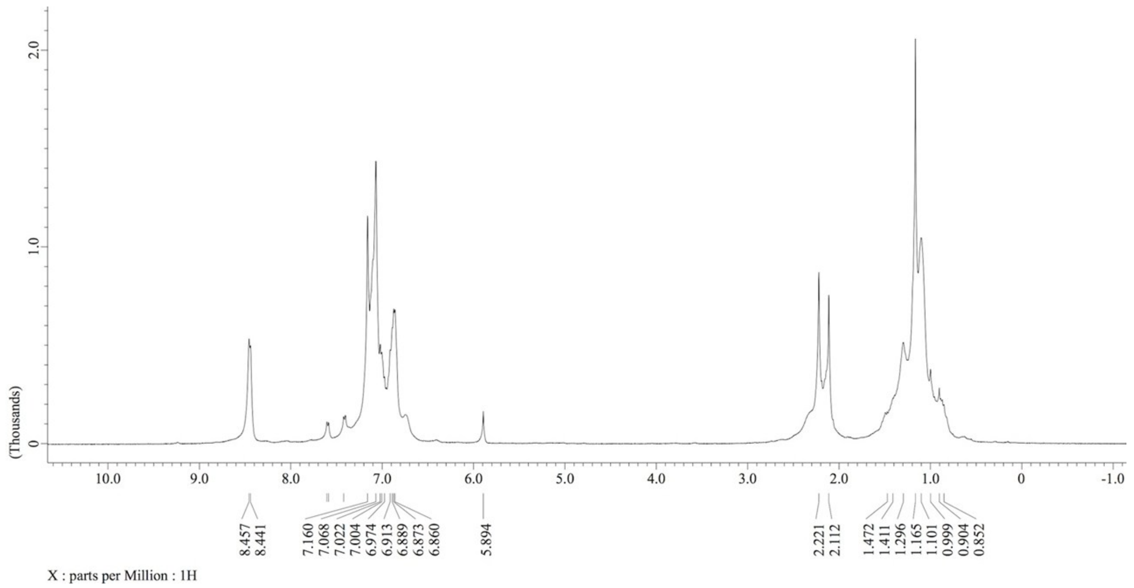

Figure S15. ^{1}H NMR spectrum of **1** with xanthone (400 MHz, in Benzene-d_6, at 300 K).

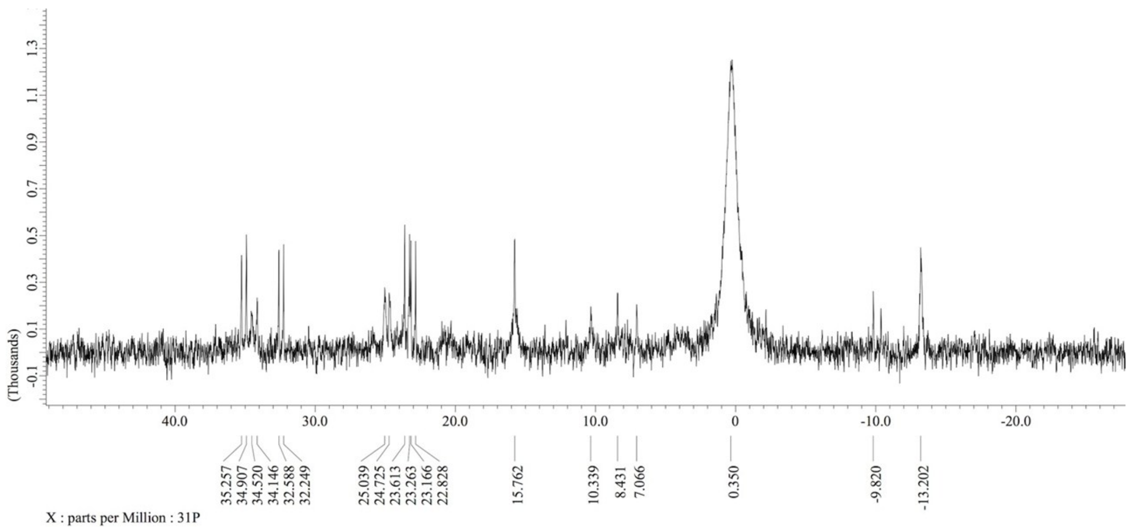

Figure S16. $^{31}P\{^{1}H\}$ NMR spectrum of **1** with xanthone (162 MHz, in Benzene-d_6, at 300 K).

Reaction of 1 with 2,2'-Bipyridine

In a J-Young valve NMR tube, a benzene-d_6 solution (0.5 mL) of **1** (11.1 mg, 14.2 μmol) was treated with 2,2'-bipyridine (6.6 mg, 42.3 μmol) at room temperature. The reaction mixture gradually changed in color from yellowish brown to violet over 24 hours at room temperature. Formation of **3** and $H_2C{=}CH^tBu$ and was observed by ^1H NMR (Figure S17 and S18). The ^{31}P NMR spectrum also showed formation of **3** (Figure S19).

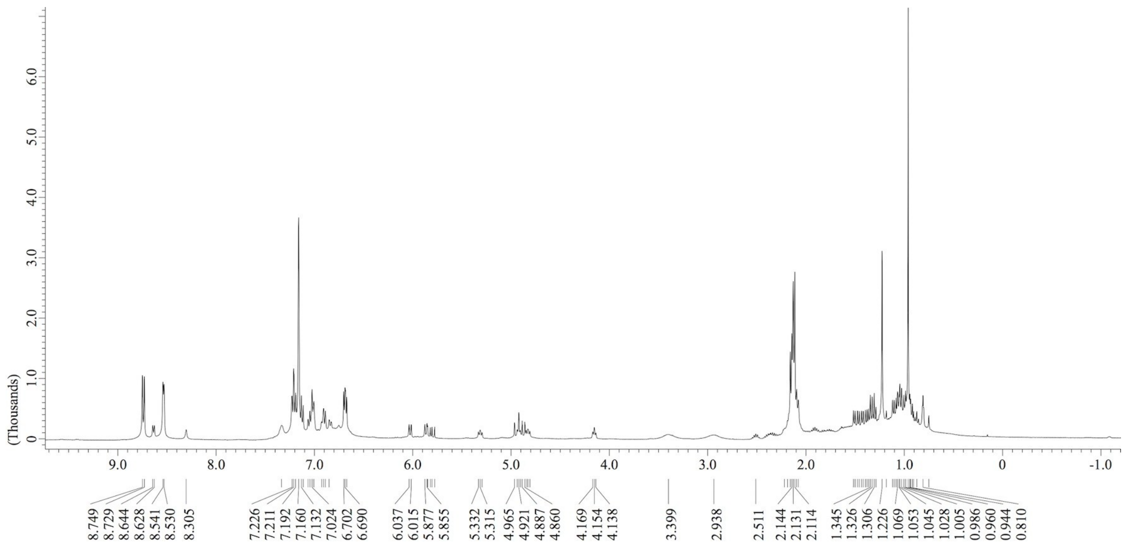

Figure S17. ^1H NMR spectrum of **1** with 2,2'-bipyridine (400 MHz, in Benzene-d_6, at 300 K).

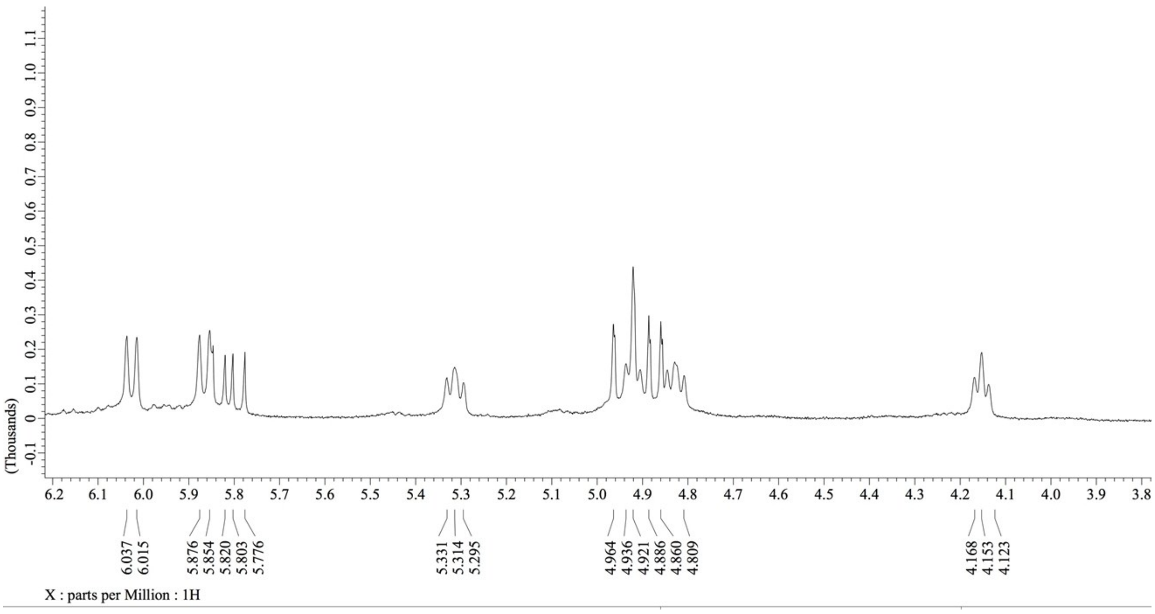

Figure S18. Expanded ^{1}H NMR spectrum around olefinic region of **1** with 2,2'-bipyridine (400 MHz, in Benzene-d_6, at 300 K).

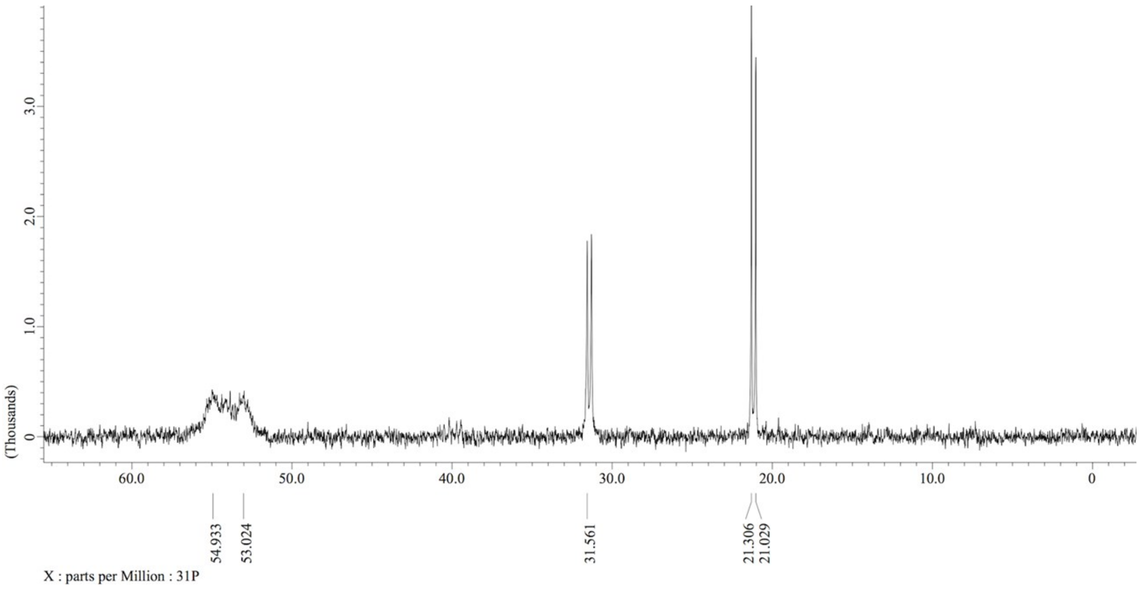

Figure S19. $^{31}P\{^{1}H\}$ NMR spectrum of **1** with 2,2'-bipyridine (162 MHz, in Benzene-d_6, at 300 K).

Reaction of 1 with PMe_3

In a J-Young valve NMR tube, a benzene-d_6 solution (0.5 mL) of **1** (10.1 mg, 18.0 μmol) was treated with PMe_3 (11 μL, 106 μmol) at room temperature. No change in color and NMR spectra was observed at room temperature for 24 hours. The reaction mixture was heated at 80 °C. The NMR spectra gradually changed, but no formation of $H_2C{=}CH^tBu$ was observed by ^1H NMR (Figure S20) even after 18 hours at 80 °C. The ^{31}P NMR spectrum also showed unreacted **1** and formation of (PNP)Ti(=CD^{t}Bu)(C_6D_5)[1] (Figure S21). Further heating at 80 °C resulted in only formation of more (PNP)Ti(=CD^{t}Bu)(C_6D_5).

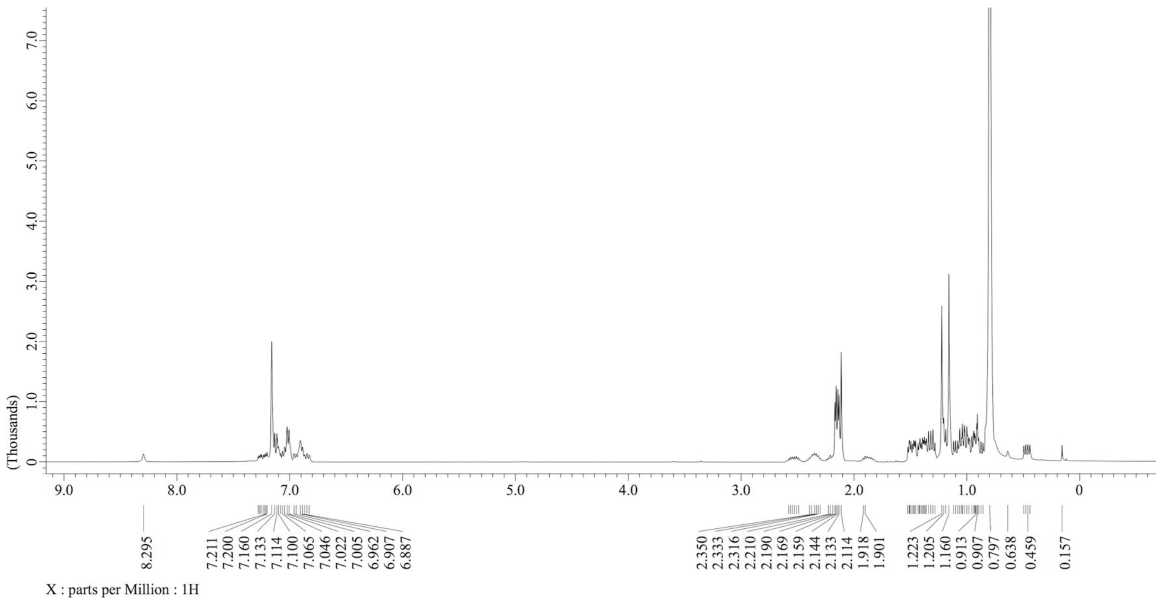

Figure S20. ^1H NMR spectrum of **1** with PMe_3 at 80 °C for 18 hours (400 MHz, in Benzene-d_6, at 300 K).

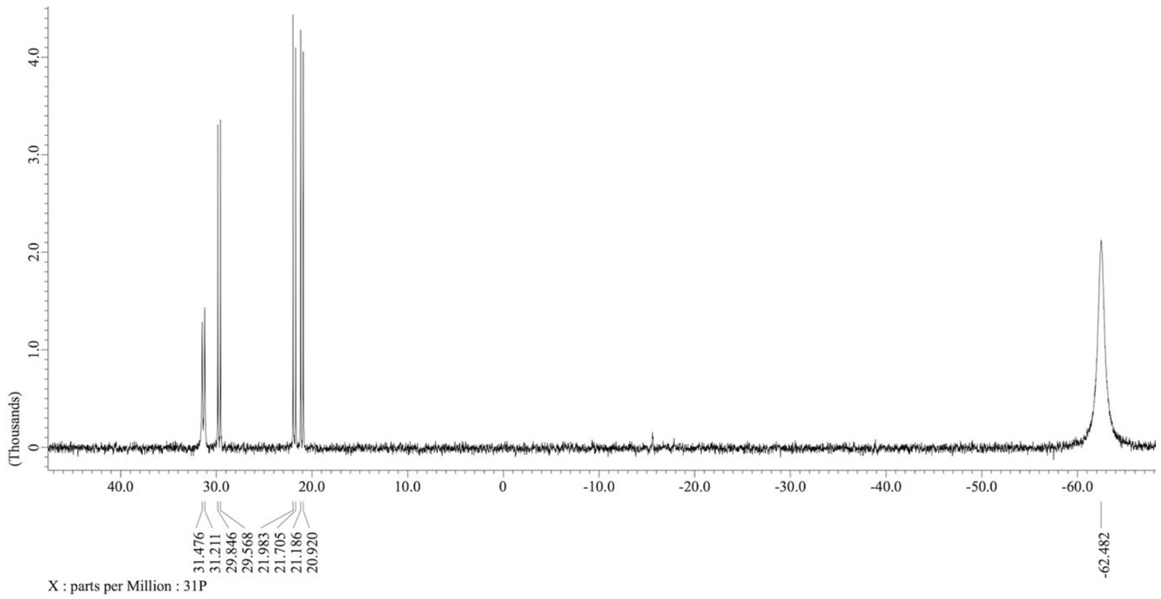

Figure S21. $^{31}P\{^1H\}$ NMR spectrum of **1** with PMe_3 at 80 °C for 18 hours (162 MHz, in Benzene-d_6, at 300 K).

Reaction of 1 with Pyridine

In a J-Young valve NMR tube, a benzene-d_6 solution (0.8 mL) of **1** (12.2 mg, 21.7 μmol) was treated with pyridine (10 μL, 124 μmol) at room temperature. No change in color and NMR spectra was observed at room temperature. The reaction mixture was heated at 80 °C. No formation of $H_2C{=}CH^tBu$ was observed by ^1H NMR (Figure S22) even after 18 hours at 80 °C. The ^{31}P NMR spectrum also showed unreacted **1** and formation of (PNP)Ti(=CD^tBu)(C_6D_5) (Figure S23). Further heating at 80 °C resulted in only formation of more (PNP)Ti(=CD^tBu)(C_6D_5).

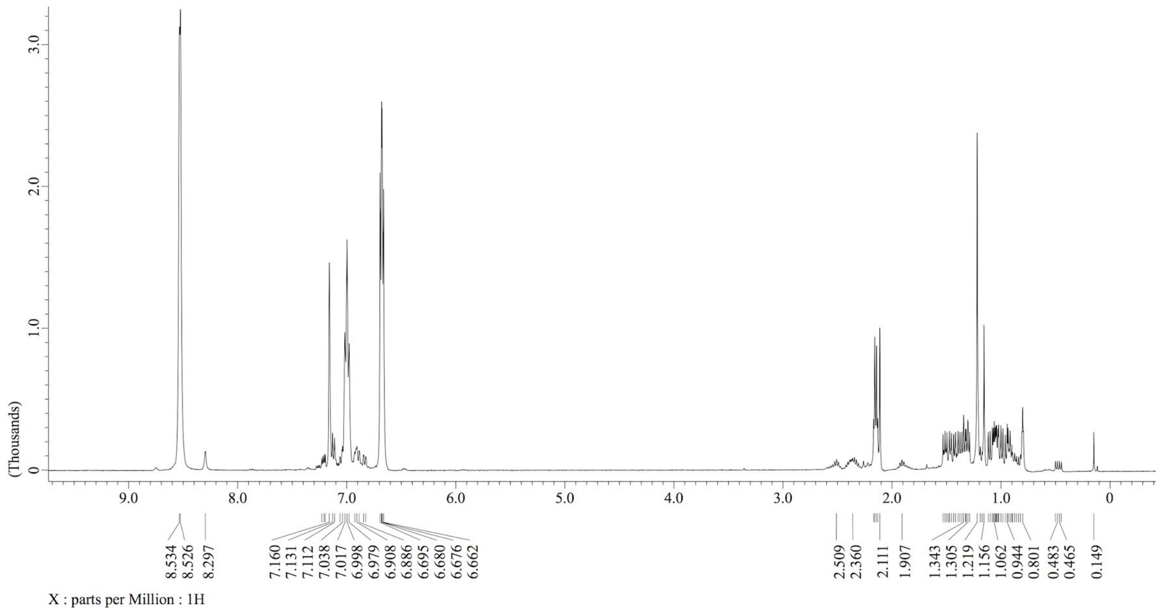

Figure S22. ^{1}H NMR spectrum of **1** with pyridine at 80 °C for 18 hours (400 MHz, in Benzene-d_6, at 300 K).

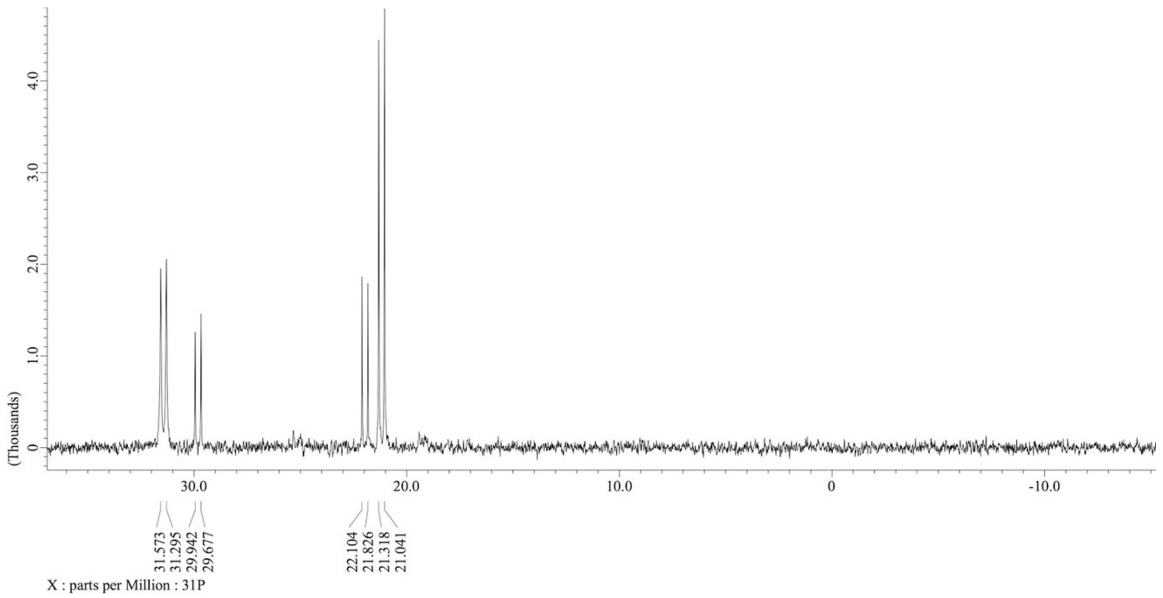

Figure S23. $^{31}P\{^{1}H\}$ NMR spectrum of **1** with pyridine at 80 °C for 18 hours (162 MHz, in Benzene-d_6, at 300 K).

Reaction of (PNP)Ti(=CH^{t}Bu)(CH$_2$^{t}Bu) with Thioxanthone

In a J-Young valve NMR tube, a benzene-d_6 solution (0.5 mL) of (PNP)Ti(=CH^{t}Bu)(CH$_2$^{t}Bu) (10.7 mg, 19.1 μmol) was treated with thioxanthone (7.1 mg, 39.0 μmol) at room temperature. The reaction mixture changed in color from yellowish green to brown over 18 hours at room temperature. Formation of ^{t}BuHC=C($C_{12}H_8S$) (5.84 ppm) and some PNP products was observed by ^{1}H NMR (Figure S24). The ^{31}P NMR spectrum also showed formation of (PNP)Ti(=CD^{t}Bu)(C_6D_5) and unidentified products from decomposition of "(PNP)Ti=O(CH$_2$^{t}Bu)" (Figure S25).

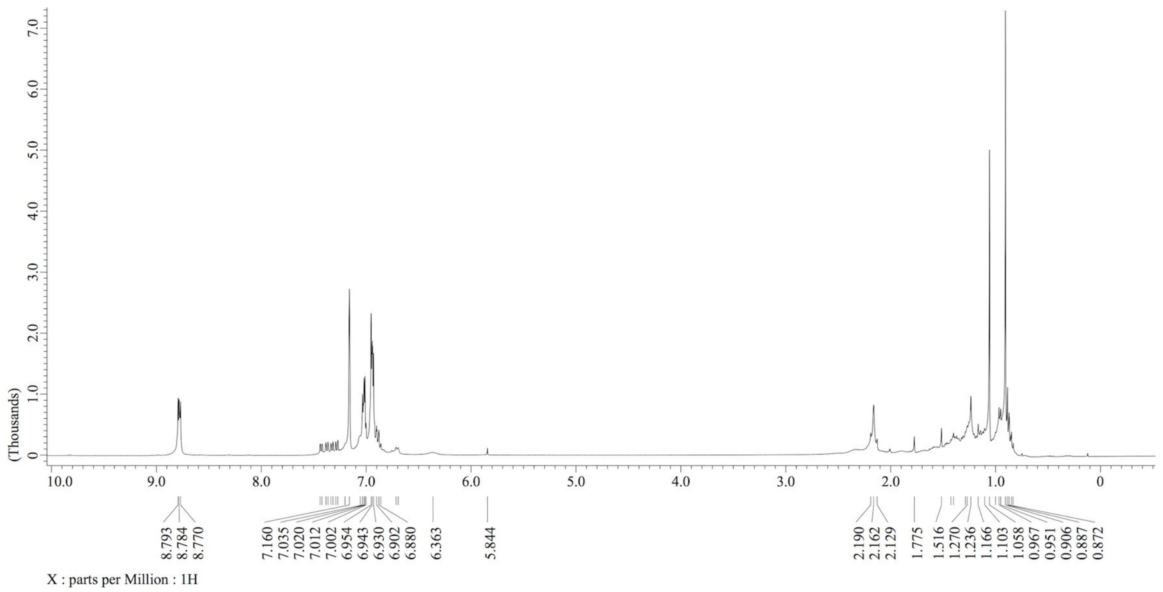

Figure S24. ^{1}H NMR spectrum of (PNP)Ti(=CH^{t}Bu)(CH$_2$^{t}Bu) with thioxanthone (400 MHz, in Benzene-d_6, at 300 K).

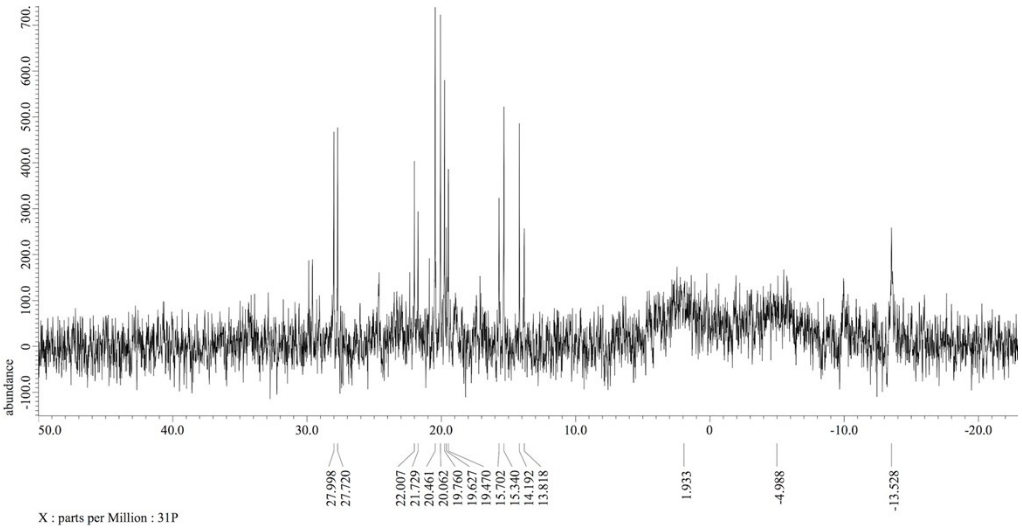

Figure S25. $^{31}P\{^1H\}$ NMR spectrum of (PNP)Ti(=CH^{t}Bu)(CH$_2{}^t$Bu) with thioxanthone (162 MHz, in Benzene-d_6, at 300 K).

Reaction of 1-D$_3$ with Thioxanthone

In a J-Young valve NMR tube, a benzene-d_6 solution (0.5 mL) of **1-D$_3$** (10.5 mg, 14.2 μmol) was treated with thioxanthone (6.9 mg, 37.9 μmol) at room temperature. The reaction mixture gradually changed in color from yellowish brown to reddish brown over 18 hours at room temperature. Formation of D_2C=CH^{t}Bu at 5.80 ppm, as well as ^{t}BuHC=C($C_{12}H_8S$) at 5.84 ppm, was observed by ^1H NMR (Figure S26 and S27). The ^{31}P NMR spectrum (Figure S28) showed no difference from that of **1** with thioxanthone to form **3**. The NMR tube was connected the distillation apparatus and all volatile materials were vacuum-transferred into another J-Young valve NMR tube. The ^1H NMR spectrum of the transferred sample showed only D_2C=CH^{t}Bu (Figure S30), except for trace solvents originally in **1-D$_3$**. The transferred H_2C=CH^{t}Bu sample was used for GC-MS analysis, described below. The ^2H NMR sample was independently prepared **1-D$_3$** (10.0 mg, 17.7 μmol) and thioxantone (7.8 mg, 42.8 μmol) in benzene (0.5 mL) with benzene-d_6 (10 μL). The ^2H NMR spectrum (Figure S29) showed **2-D** at 6.88 ppm (br m) and D_2C=CH^{t}Bu at 4.87 (d, $^3J_{HD}$ = 1.7 Hz), 4.94 (d, $^3J_{HD}$ = 2.6 Hz).

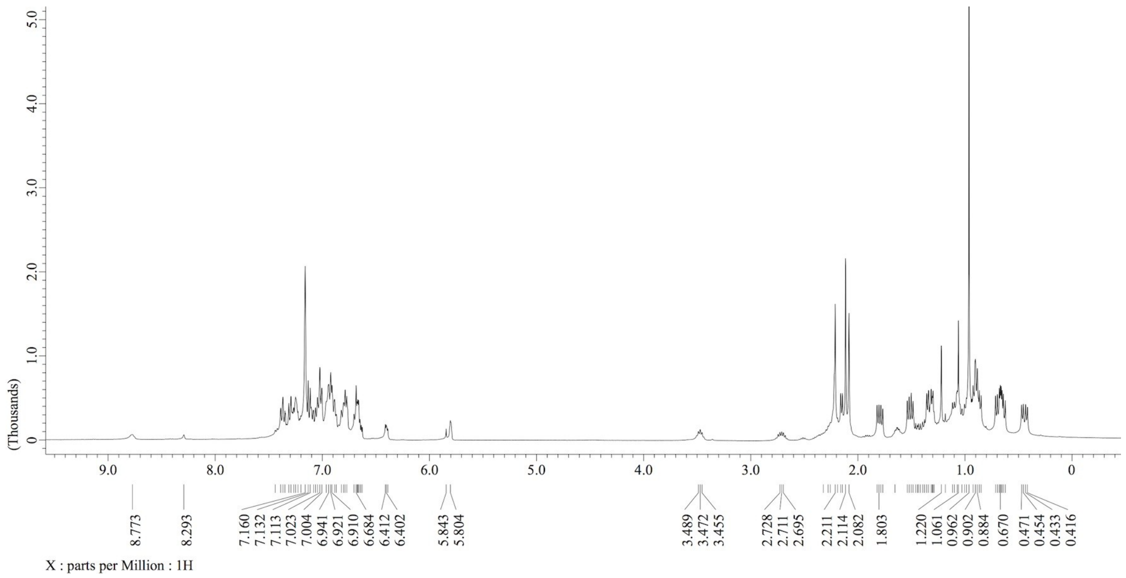

Figure S26. ^{1}H NMR spectrum of **1-D$_3$** with thioxanthone (400 MHz, in Benzene-d_6, at 300 K).

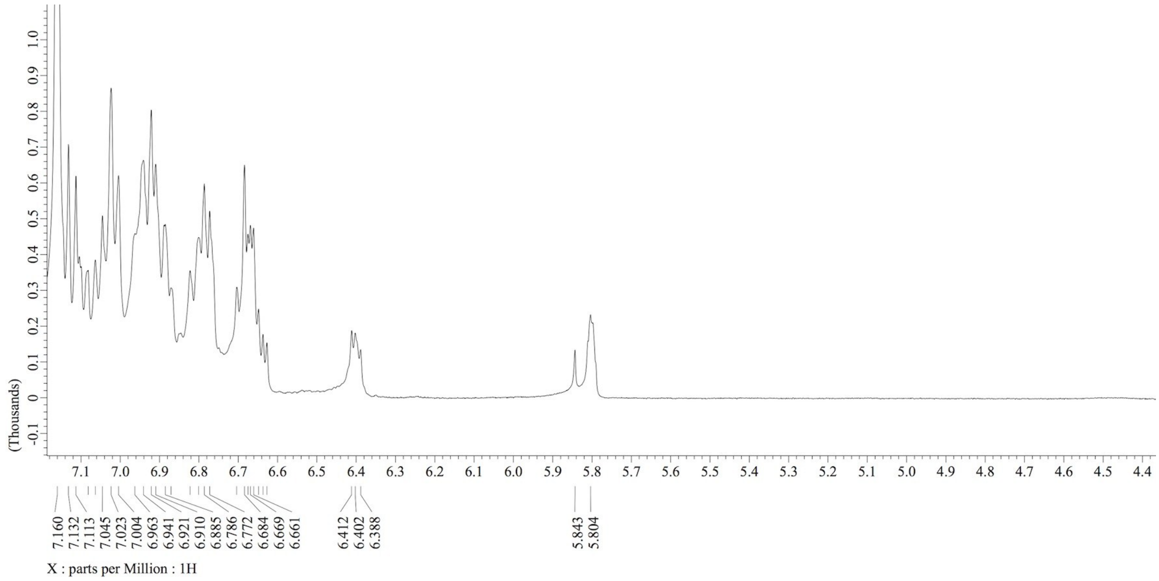

Figure S27. Expanded ^{1}H NMR spectrum around olefinic region of **1-D$_3$** with thioxanthone (400 MHz, in Benzene-d_6, at 300 K).

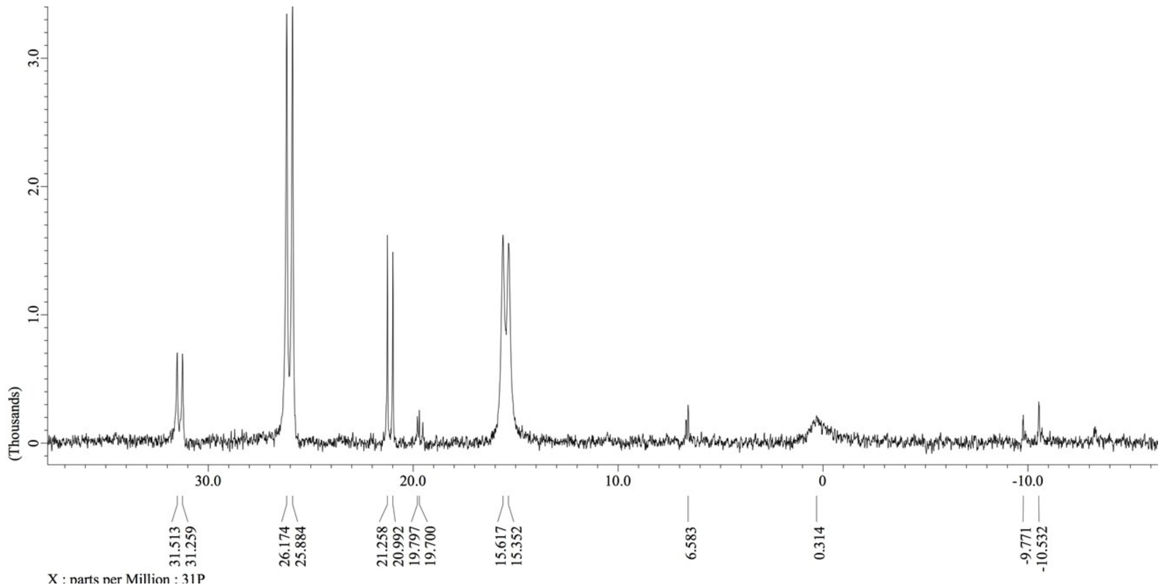

Figure S28. $^{31}P\{^1H\}$ NMR spectrum of **1-D$_3$** with thioxanthone (162 MHz, in Benzene-d_6, at 300 K).

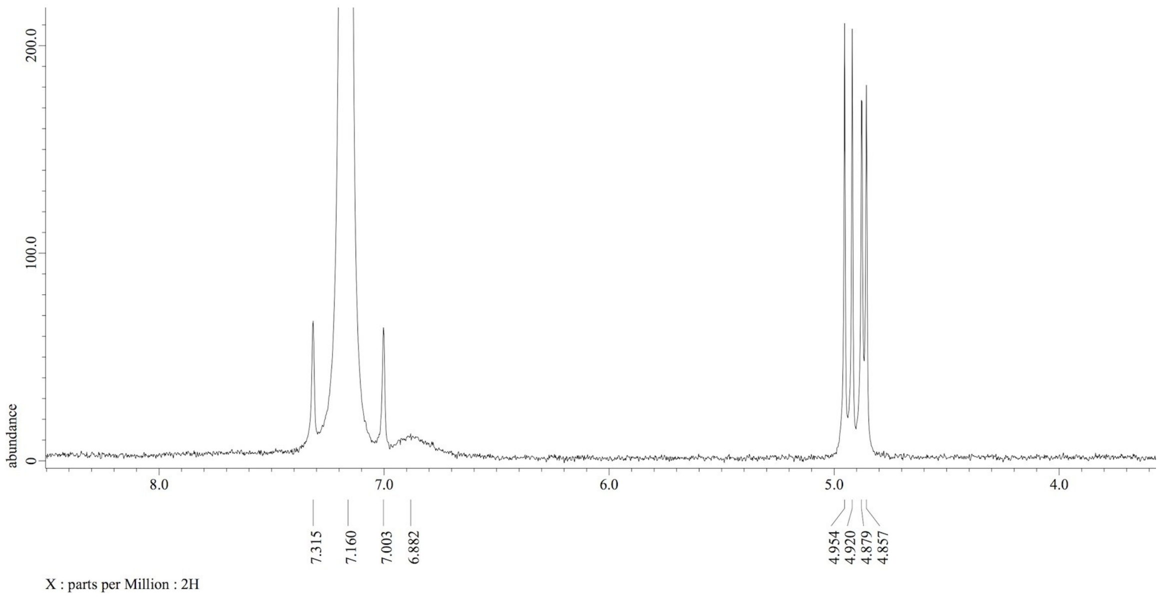

Figure S29. ^2H NMR spectrum of **1-D$_3$** with thioxanthone (76.7 MHz, in Benzene/Benzene-d_6, at 300 K).

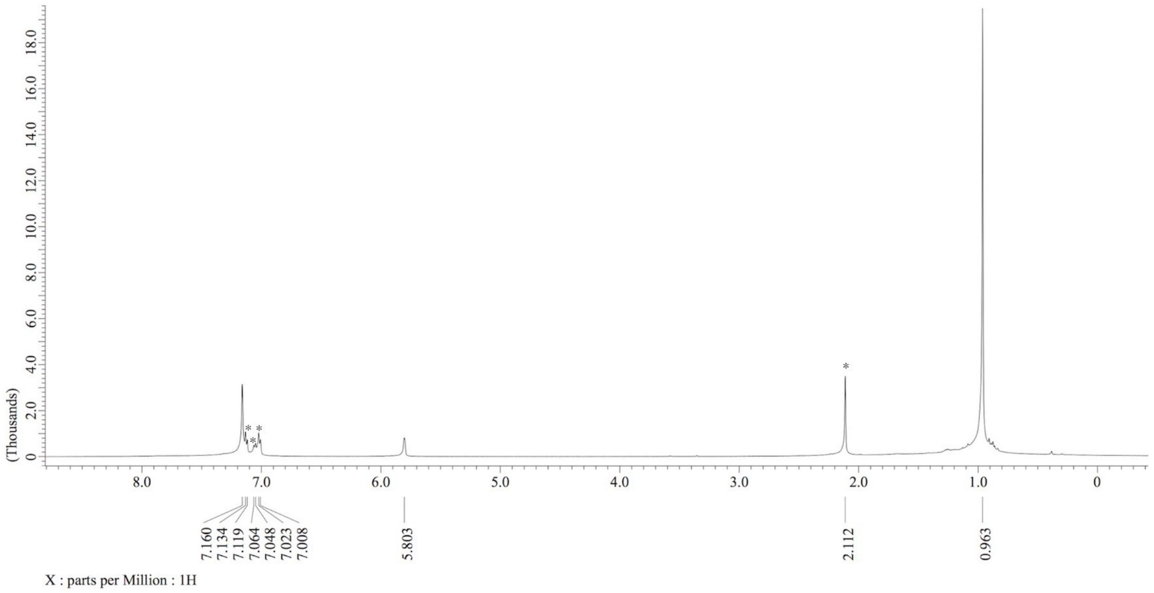

Figure S30. ^{1}H NMR spectrum of volatile materials from **1-D$_3$** with thioxanthone (400 MHz, in Benzene-d_6, at 300 K). *: toluene.

Reaction of 1-D$_3$ with 2,2'-Bipyridine

In a J-Young valve NMR tube, a benzene solution (0.5 mL) of **1-D$_3$** (10.9 mg, 19.3 μmol) was treated with 2,2'-bipyridine (8.9 mg, 48.8 μmol) and benzene-d_6 (10 μL) at room temperature. The reaction mixture gradually changed in color from yellowish brown to violet over 24 hours at room temperature. The ^{31}P NMR spectrum showed the same resonances with those of **3**. The ^{2}H NMR spectrum (Figure S31) showed **3-D** at 6.80 ppm (t, $^{3}J_{HD}$ = 5.5 Hz) and D_2C=CH^{t}Bu at 4.87 (d, $^{3}J_{HD}$ = 1.7 Hz), 4.94 (d, $^{3}J_{HD}$ = 2.6 Hz).

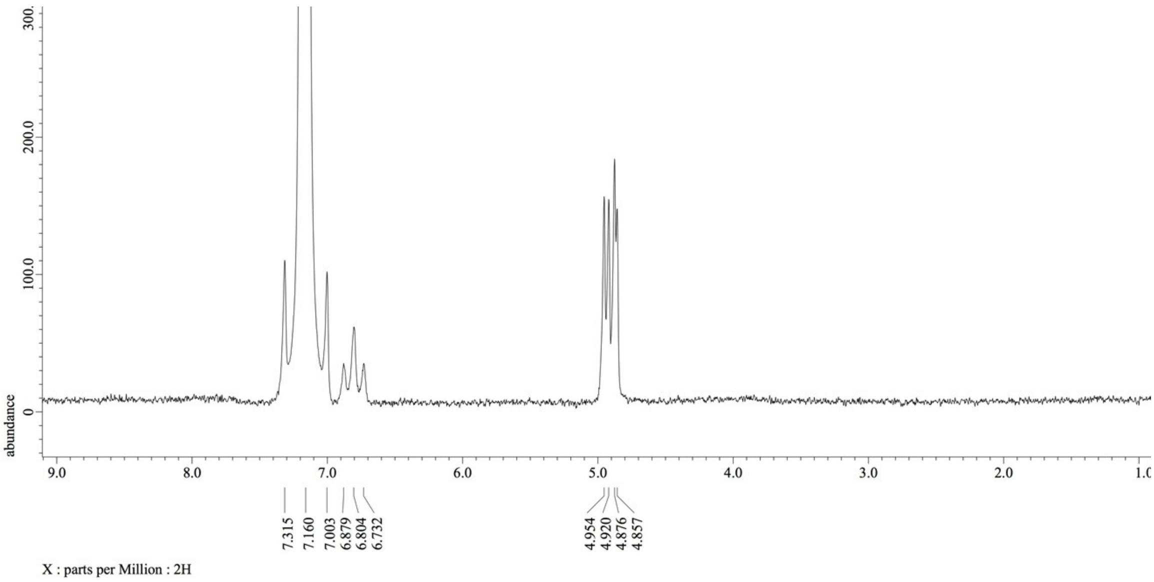

Figure S31. ^{2}H NMR spectrum of **1-D$_3$** with 2,2'-bipyridine (76.7 MHz, in Benzene/Benzene-d_6, at 300 K).

Reaction of 1-^{13}C with Thioxanthone

In a J-Young valve NMR tube, a benzene-d_6 solution (0.5 mL) of **1-^{13}C** (20.1 mg, 35.7 μmol) was treated with thioxanthone (26.1 mg, 143 μmol) at room temperature. The reaction mixture gradually changed in color from yellowish brown to reddish brown over 18 hours at room temperature. Formation of $H_2C{=}^{13}CH^{t}Bu$, as well as $^{t}BuHC{=}C(C_{12}H_8S)$ at 5.84 ppm, was observed by ^{1}H NMR (Figure S32 and S33). The ^{13}C NMR spectrum of the reaction mixture showed an enriched ^{13}C NMR resonance at 109.30 ppm (Figure S34). The NMR tube was connected the distillation apparatus and all volatile materials were vacuum-transferred into another J-Young valve NMR tube. The ^{1}H and ^{13}C NMR spectra of the transferred sample showed only $H_2C{=}^{13}CH^{t}Bu$ (Figure S35 and S36), except for trace solvents originally in **1-^{13}C**.

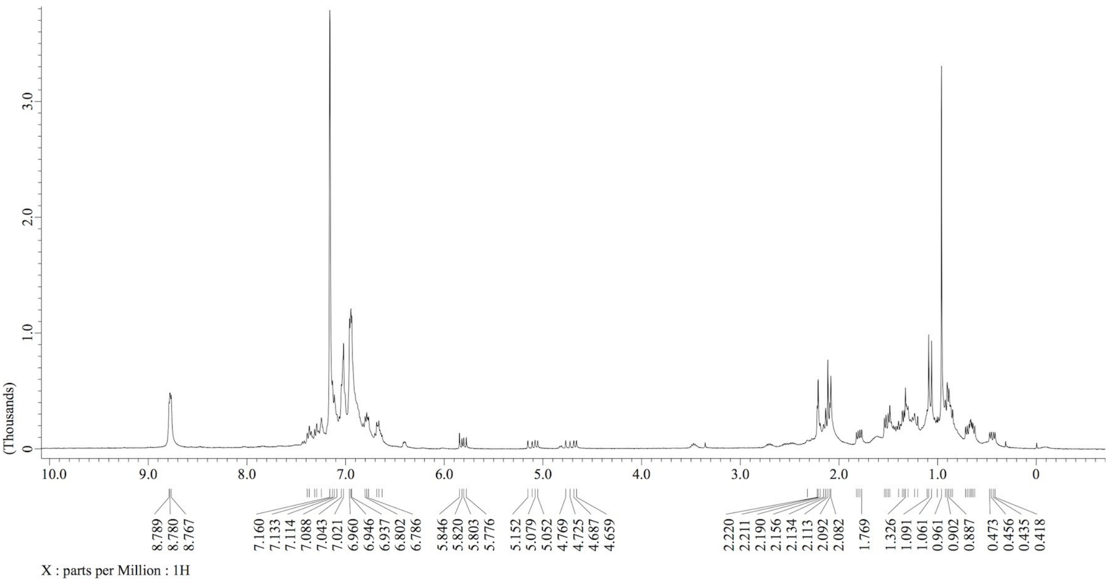

Figure S32. ^{1}H NMR spectrum of **1-^{13}C** with thioxanthone (400 MHz, in Benzene-d_6, at 300 K).

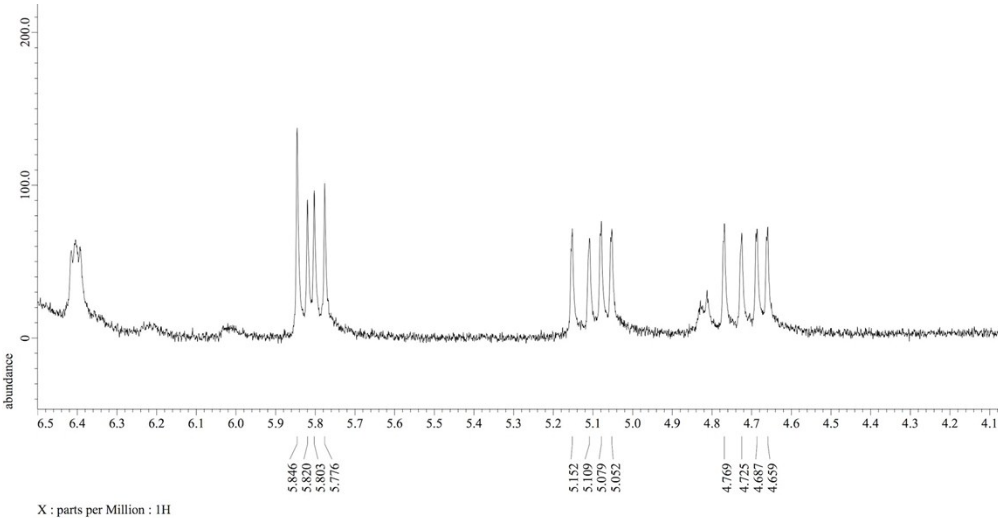

Figure S33. Expanded ^{1}H NMR spectrum around olefinic region of **1-^{13}C** with thioxanthone (400 MHz, in Benzene-d_6, at 300 K).

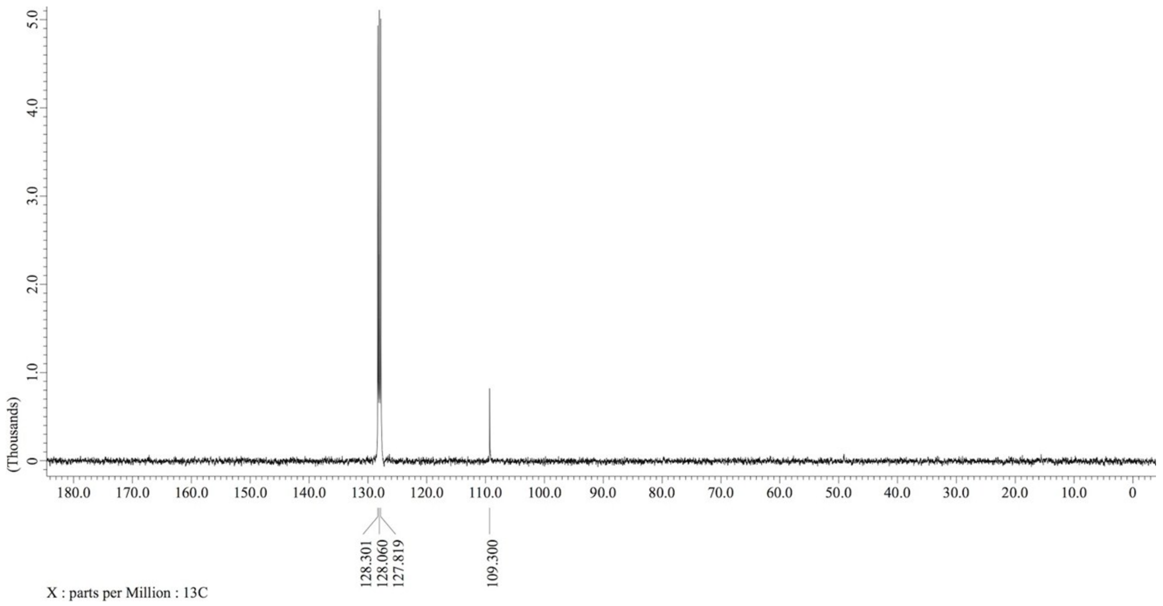

Figure S34. $^{13}C\{^{1}H\}$ NMR spectrum of **1-^{13}C** with thioxanthone (101 MHz, in Benzene-d_6, at 300 K).

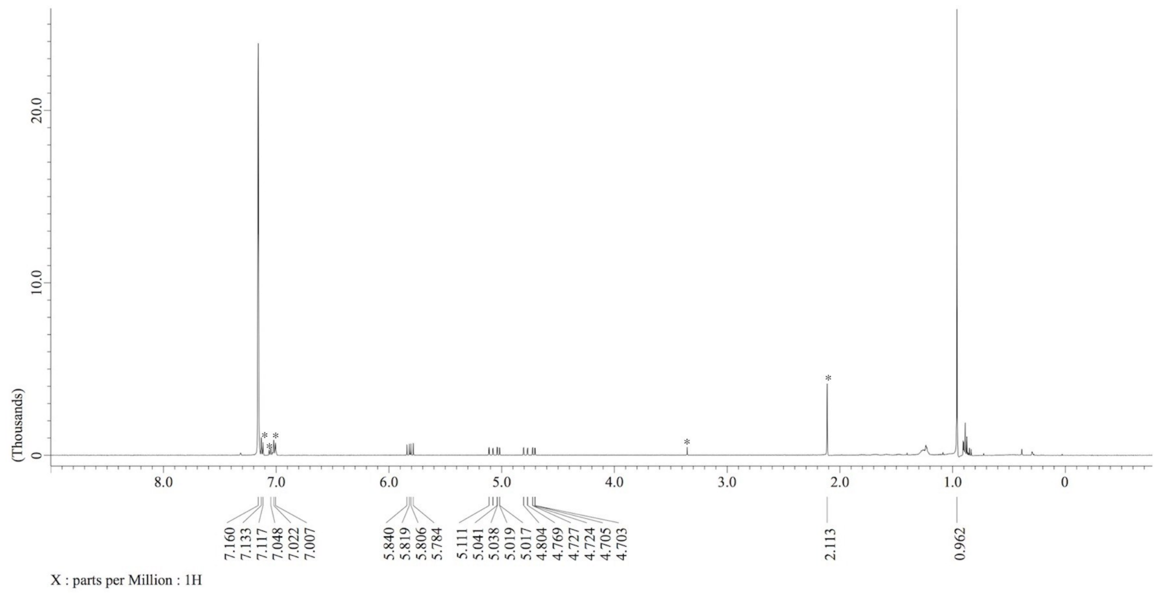

Figure S35. ^{1}H NMR spectrum of volatile materials from **1-^{13}C** with thioxanthone (400 MHz, in Benzene-d_6, at 300 K). *: toluene and 1,4-dioxane.

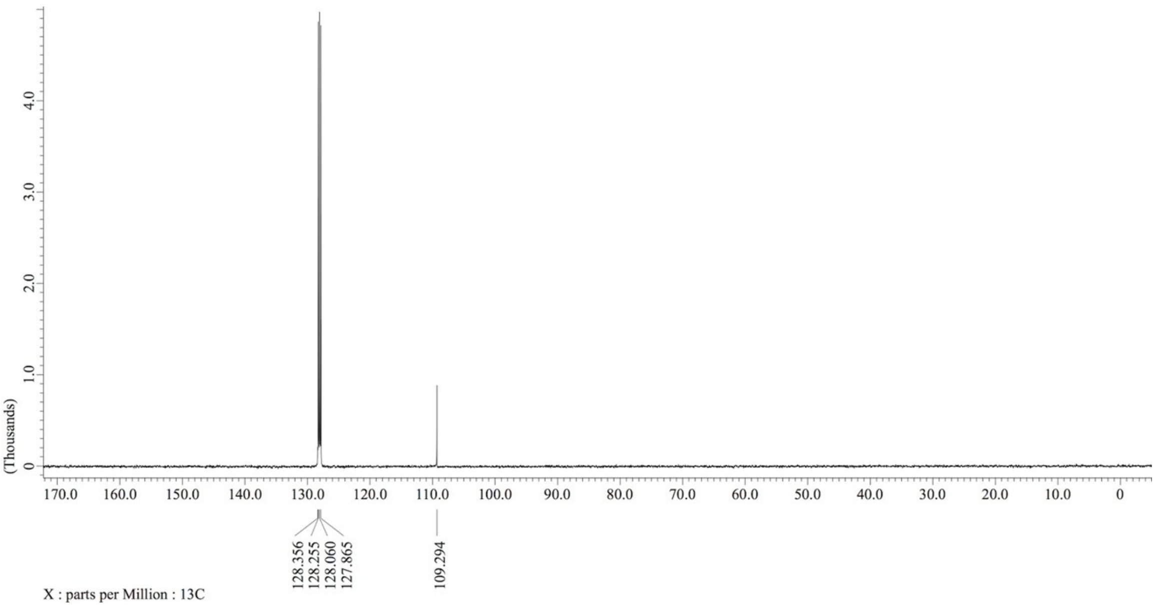

Figure S36. $^{13}C\{^1H\}$ NMR spectrum of volatile materials from **1-^{13}C** with thioxanthone (101 MHz, in Benzene-d_6, at 300 K).

Reaction of 1-^{13}C with 2,2'-Bipyridine

In a J-Young valve NMR tube, a benzene-d_6 solution (0.5 mL) of **1-^{13}C** (11.2 mg, 19.9 μmol) was treated with 2,2'-bipyridine (8.3 mg, 45.6 μmol) at room temperature. The reaction mixture gradually changed in color from yellowish brown to violet over 18 hours at room temperature. Formation of $H_2C{=}^{13}CH^tBu$ and **3** was observed by ^1H NMR (Figure S37 and S38). The ^{13}C NMR spectrum of the reaction mixture showed an enriched ^{13}C NMR resonance at 109.29 ppm (Figure S39).

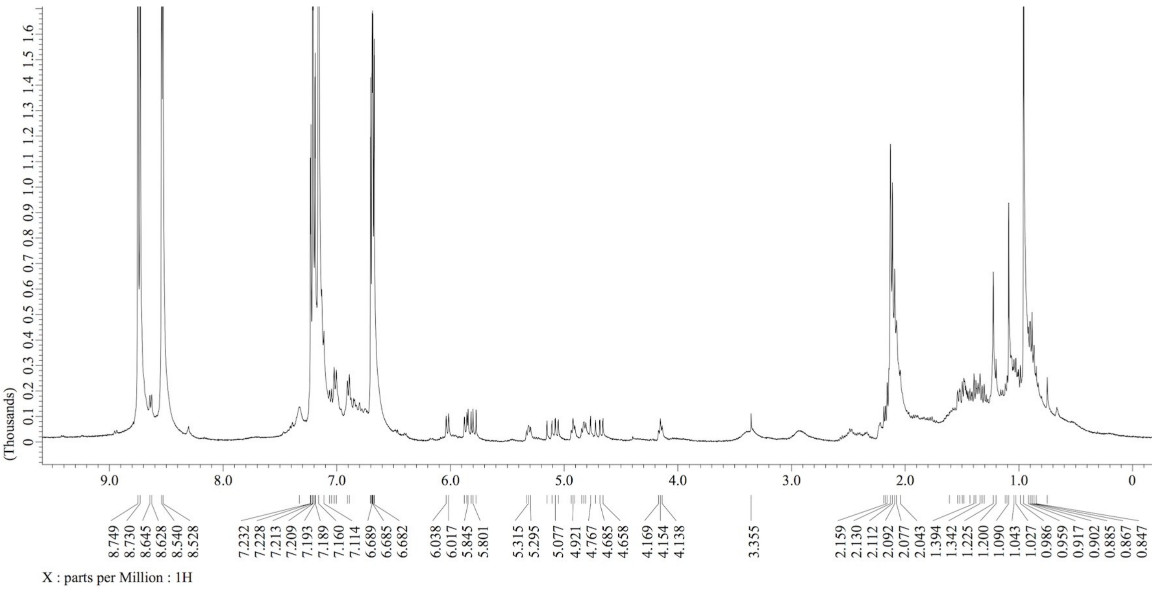

Figure S37. ^{1}H NMR spectrum of **1-^{13}C** with 2,2'-bipyridine (400 MHz, in Benzene-d_6, at 300 K).

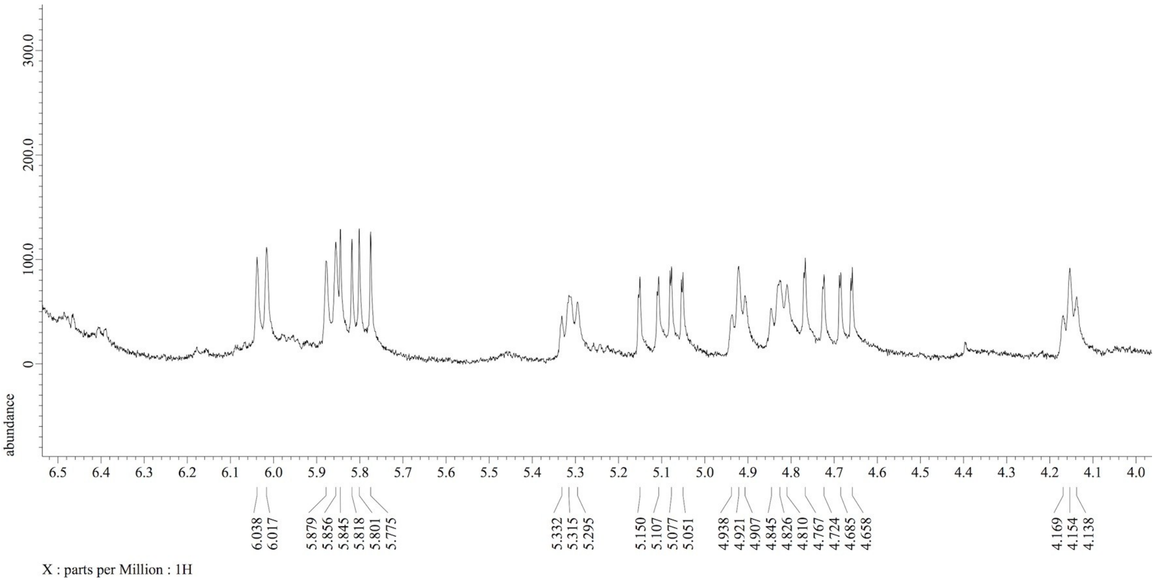

Figure S38. Expanded ^{1}H NMR spectrum around olefinic region of **1-^{13}C** with 2,2'-bipyridine (400 MHz, in Benzene-d_6, at 300 K).

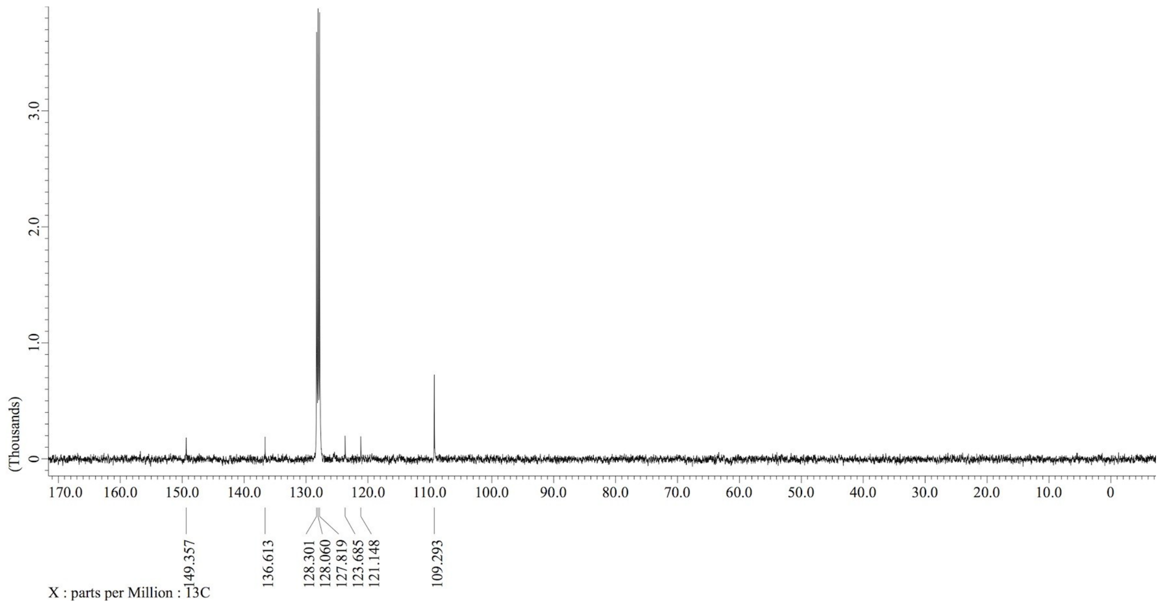

Figure S39. $^{13}C\{^{1}H\}$ NMR spectrum of **1-^{13}C** with 2,2'-bipyridine (101 MHz, in Benzene-d_6, at 300 K).

GC-MS Analyses

For GC-MS analyses of $H_2C{=}CH^tBu$ and $D_2C{=}CH^tBu$, their solutions were transferred into a sure-sealed vial and vaporized by heating. The vapor was injected (*via* PAL auto-sampler) into an Agilent Technologies 7890B GC system equipped with an Agilent Technologies 5977A MS detector. A split ratio of 400:1 (Split flow 480 mL/min) was employed with helium (1.25 mL/min) as the carrier gas. Separation was accomplished through two Agilent HP-ms Ultra Inert columns (15 m x 250 µm x 0.25 µm, from 40 °C to 325 °C with 50 °C/min rate) equipped for backflush. For liquid samples, the MS spectrum was collected from 80 °C to 250 °C for 12 minutes.

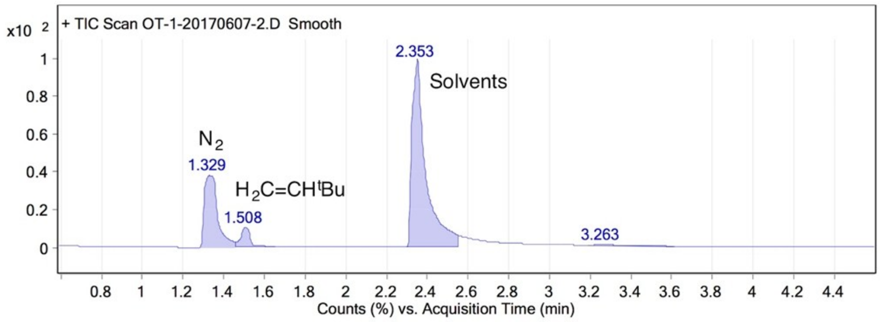

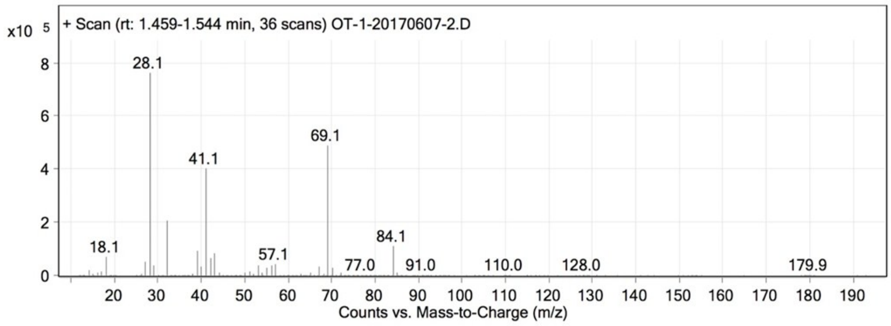

Figure S40. GC-MS spectrum of $H_2C{=}CH^tBu$ sample from **1** with thioxanthone (Top: Gas Chromatogram, Bottom: Mass Spectrum around 1.508 min).

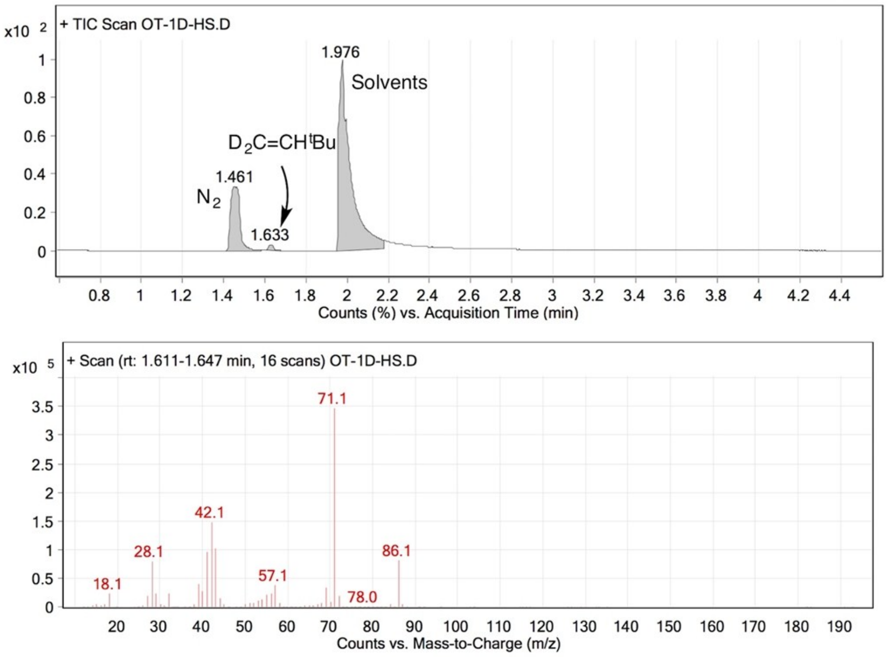

Figure S41. GC-MS spectrum of $D_2C=CH^tBu$ sample from **1-D$_3$** with thioxanthone (Top: Gas Chromatogram, Bottom: Mass Spectrum around 1.633 min).

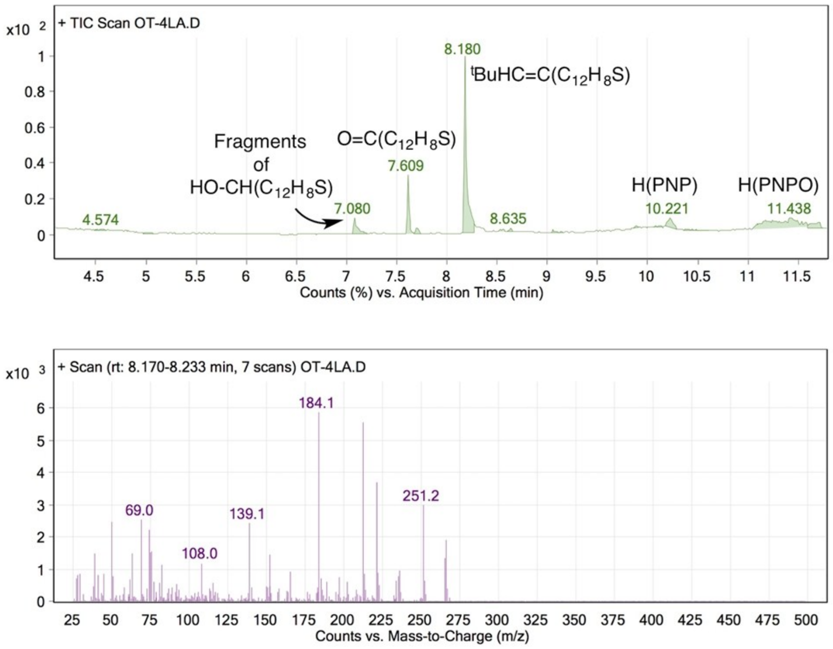

Figure S42. GC-MS spectrum of filtrate from **1** with thioxanthone (Top: Gas Chromatogram, Bottom: Mass Spectrum around 8.180 min).

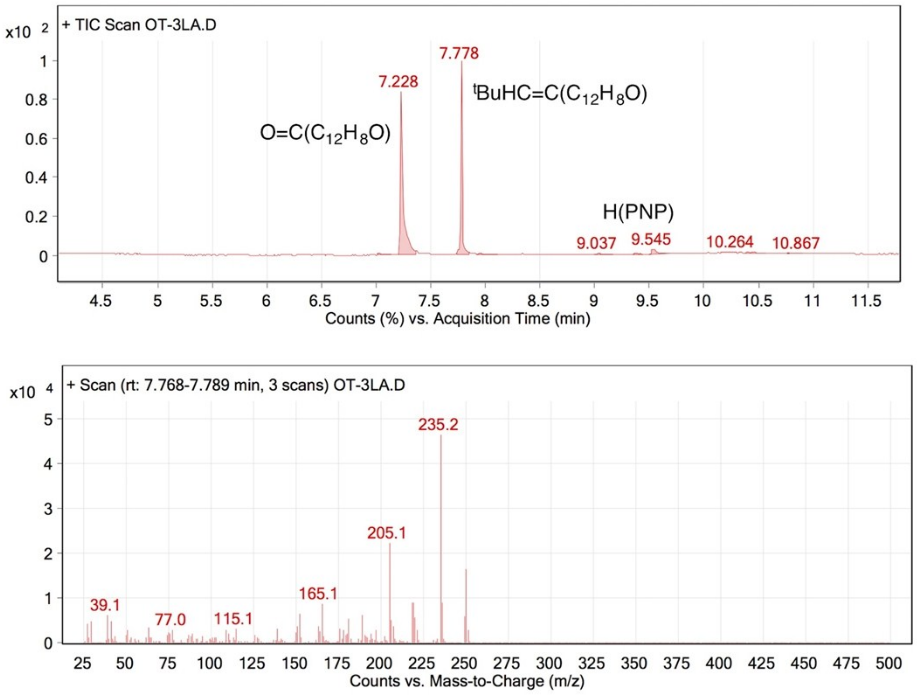

Figure S43. GC-MS spectrum of **1** with xanthone (Top: Gas Chromatogram, Bottom: Mass Spectrum around 8.180 min).

FT-IR

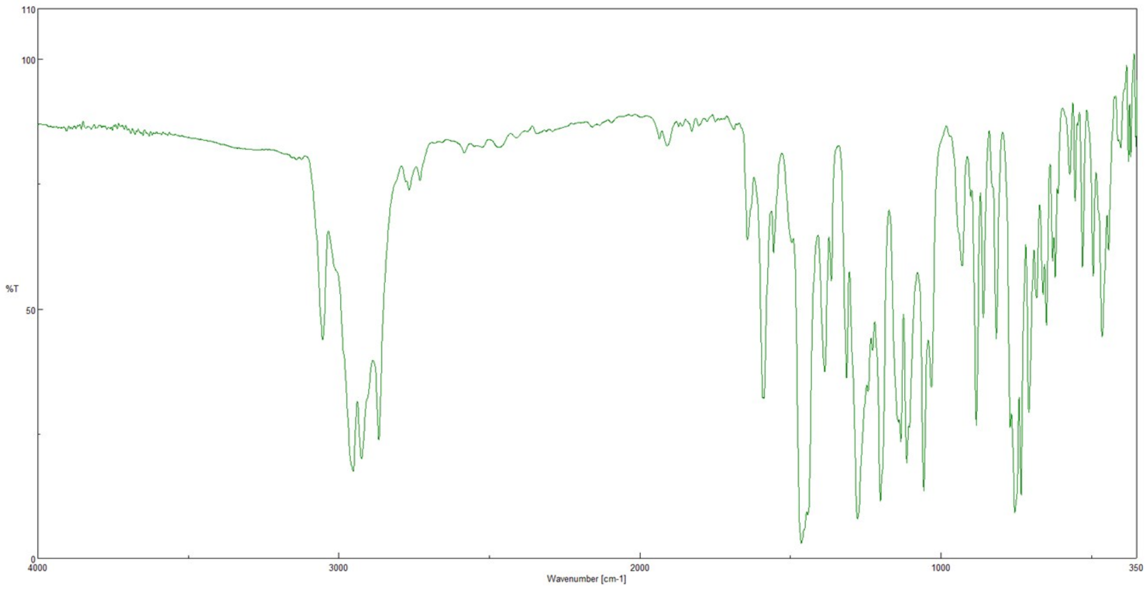

Figure S44. IR spectrum of **2** (KBr, solid, at room temperature).

UV-Vis

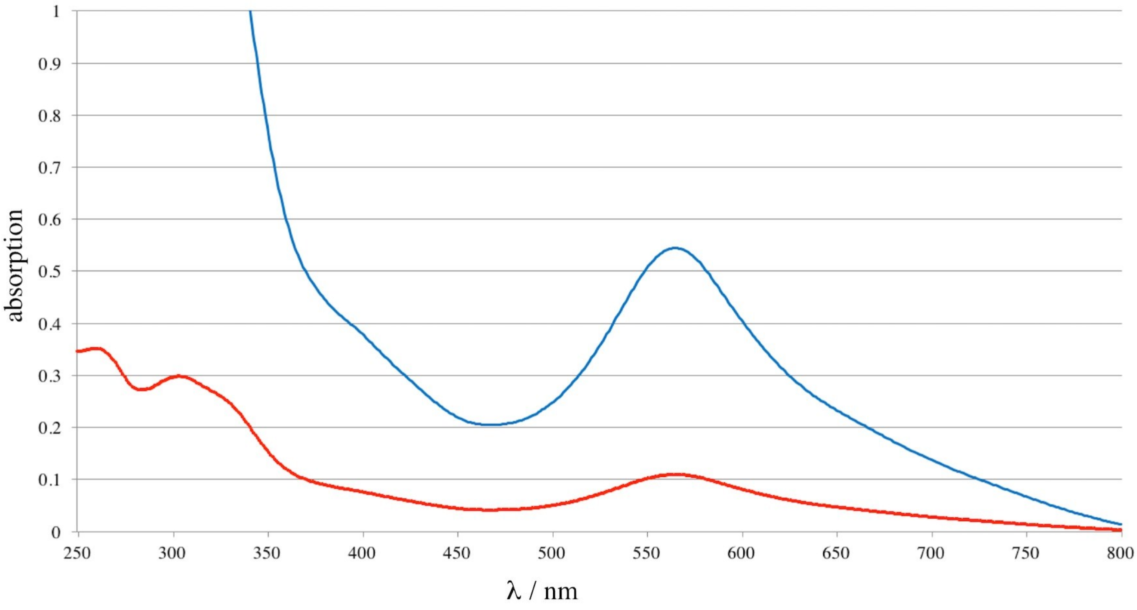

Figure S45. UV-Vis spectrum of **3** (red; 0.013 mM, blue; 0.065 mM in THF, at room temperature).

X-ray Crystallography

Crystallographic data are summarized Table S1-S2. Suitable crystals for X-ray analysis of **2** and **3** were placed on the end of a Cryoloop coated in NVH oil. The X-ray intensity data collection was carried out on a Bruker APEXII CCD area detector using graphite-monochromated Mo-Kα radiation (λ = 0.71073 Å) at 100(1) K. Preliminary indexing was performed from a series of thirty-six 0.5° rotation frames with exposures of 10 seconds. Rotation frames were integrated using SAINT,[3] producing a listing of non-averaged F^2 and $\sigma(F^2)$ values. The intensity data were corrected for Lorentz and polarization effects and for absorption using SADABS.[4] The initial structure was determined by the direct or Patterson method on SHELXS.[5] The further structure determination was performed by Fourier transform method and refined by least squares method on SHELXL.[5,6] All reflections were used during refinement. Non-hydrogen atoms were refined anisotropically and hydrogen atoms were refined using riding models, except for H39 (**2**) and H27A/B-H30 (**3**). For **2**, the methine hydrogen H39 was located from the difference map and refined isotropically. For **3**, one ^{i}Pr group of PNP was disordered over two positions. The thermal ellipsoids of these disorders were fixed by SHELXL restraint commands. The methylene hydrogens (H27A and H27B) and olefinic hydrogens (H28-H30) were located from the difference map and refined isotoropically. These results were checked using the IUCR's CheckCIF routine. The alerts in the output are related to the disordered groups.

Table S1. Summary of Structure Determination of 2

Empirical formula	$C_{52}H_{57}NO_2P_2STi$
Formula weight	901.94
Temperature	100(1) K
Wavelength	0.71073 Å
Crystal system	*Monoclinic*
Space group	$P2_1/c$ (No. 14)
Cell constants:	
a	14.314(3) Å
b	12.233(3) Å
c	27.034(6) Å
α	90 °
β	102.751(13) °
γ	90 °
Volume	4617.0(18) Å^3
Z	4
Density (calculated)	1.298 Mg/m^3
Absorption coefficient	0.387 mm^{-1}
F(000)	1904
Crystal size	0.13 x 0.10 x 0.02 mm^3
Theta range for data collection	1.459 to 27.615 °
Index ranges	$-18 \leq h \leq 18$, $-15 \leq k \leq 15$, $-35 \leq l \leq 35$
Reflections collected	102996
Independent reflections	10669 [R (int) = 0.0598]
Completeness to theta = 27.55°	99.5 %
Absorption correction	Semi-empirical from equivalents
Max. and min. transmission	0.7456 and 0.6546
Refinement method	Full-matrix least-squares on F^2
Data / restraints / parameters	10669 / 0 / 555
Goodness-of-fit on F^2	1.012
Final R indices [I>2sigma(I)]	$R_1 = 0.0426$, $wR_2 = 0.0867$
R indices (all data)	$R_1 = 0.0785$, $wR_2 = 0.0983$
Largest diff. peak and hole	0.455 and -0.486 e.Å^{-3}

Table S2. Summary of Structure Determination of 3·(bipy)

Empirical formula	$C_{56}H_{65}N_7P_2Ti$
Formula weight	945.99
Temperature	100(1) K
Wavelength	0.71073 Å
Crystal system	*Monoclinic*
Space group	$P2_1/n$ (No. 14)
Cell constants:	
a	11.835(2) Å
b	19.146(3) Å
c	22.470(4) Å
α	90 °
β	102.101(8) °
γ	90 °
Volume	4978.4(16) Å^3
Z	4
Density (calculated)	1.262 Mg/m^3
Absorption coefficient	0.281 mm^{-1}
F(000)	2008
Crystal size	0.22 x 0.20 x 0.11 mm^3
Theta range for data collection	1.411 to 27.635 °
Index ranges	$-15 \leq h \leq 14$, $-24 \leq k \leq 23$, $-29 \leq l \leq 26$
Reflections collected	102515
Independent reflections	11521 [R (int) = 0.0330]
Completeness to theta = 27.55°	99.3 %
Absorption correction	Semi-empirical from equivalents
Max. and min. transmission	0.7456 and 0.7037
Refinement method	Full-matrix least-squares on F^2
Data / restraints / parameters	11521 / 43 / 651
Goodness-of-fit on F^2	1.033
Final R indices [I>2sigma(I)]	$R_1 = 0.0409$, $wR_2 = 0.0911$
R indices (all data)	$R_1 = 0.0635$, $wR_2 = 0.1012$
Largest diff. peak and hole	0.414 and -0.460 e.Å^{-3}

Computational Details

All calculations were carried out using DFT[7] as implemented in the Jaguar 9.1 suite[8] of *ab initio* quantum chemistry programs. Geometry optimizations were proceeded with B3LYP[9-13] functional including Grimme's D3 dispersion correction[14] and the 6-31G** basis set. Ti was represented using the Los Alamos LACVP basis[15-17] that includes effective core potentials. For reducing computational cost, the PNP ligand system has been simplified by changing isopropyl groups on phosphorus to methyl groups. The energies of the optimized structures were reevaluated by additional single point calculations on each optimized geometry using B3LYP and Dunning's correlation consistent triple-ζ basis set cc-pVTZ(-f)[18] which includes a double set of polarization functions. A modified version of LACVP has been employed for Ti, designated as LACV3P, in which the exponents were decontracted to match the effective core potential with triple-ζ quality. A self-consistent reaction field (SCRF)[19-21] approach based on accurate numerical solutions of the Poisson-Boltzmann equation was employed for solvation energies. In the results reported, solvation calculations were performed with the 6-31G**/LACVP basis at the optimized gas phase geometry using the dielectric constant of $\varepsilon = 2.284$ for benzene. As is the case for all continuum models, the solvation energies are subject to empirical parametrization of the atomic radii that are used to generate the solute surface. The standard set of optimized radii in Jaguar was used for H (1.150 Å), C (1.900 Å), N (1.600Å), O (1.600Å), P (2.074 Å), S (1.900 Å) and Ti (1.587 Å).[22]

Analytical vibrational frequencies within the harmonic approximation were computed with the 6-31G**/LACVP basis to confirm proper convergence to well-defined minima or saddle points on the potential energy surface.

The energy components have been computed with the following protocol. The free energy in solution phase G(sol) has been calculated as follows:

$$G(sol) = G(gas) + G^{solv} \quad (1)$$

$$G(gas) = H(gas) - TS(gas) \quad (2)$$

$$H(gas) = E(SCF) + ZPE \quad (3)$$

$$\Delta E(SCF) = \Sigma E(SCF) \text{ for products} - \Sigma E(SCF) \text{ for reactants} \quad (4)$$

$$\Delta G(sol) = \Sigma G(sol) \text{ for products} - \Sigma G(sol) \text{ for reactants} \quad (5)$$

G(gas) is the free energy in gas phase; G^{solv} is the free energy of solvation as computed using the continuum solvation model; H(gas) is the enthalpy in gas phase; T is the temperature (298.15K); S(gas) is the entropy in gas phase; E(SCF) is the self-consistent field energy, i.e. "raw" electronic energy as computed from the SCF procedure and ZPE is the zero point energy. Note that by entropy here we refer specifically to the vibrational/rotational/translational entropy of the solute(s); the entropy of the solvent is incorporated implicitly in the continuum solvation model.

Additionally, quantitative perturbation molecular orbital diagram (Figure 11.) was carried out using DFT as implemented in the Amsterdam Density Functional (ADF) 2017.101 suite of ab initio quantum chemistry programs. Single point calculation on each optimized geometry using B3LYP-D3 and triple-ζ basis set TZP[23] with no frozen core and supported by zeroth-order regular approximation (ZORA) which supports scalar relativistic level correction.

Comparison of energy difference between reductive C–C bond formation and α-hydrogen abstraction steps

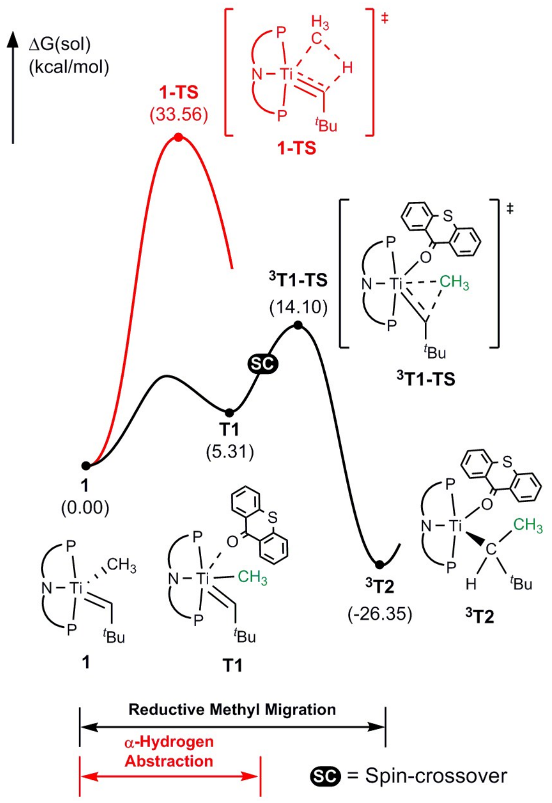

Figure S46. Free energy profile of α-hydrogen abstraction to form CH_4 and **A**

Computational calculation result of reductive methyl migration using pyridine and phosphine (PMe_3) as an additive ligand

As illustrated in Figure S47a, the energy barrier of reductive methyl migration is quite high, and Figure S47b represents that PMe_3 and pyridine ligands do not play a role as redox-active ligand.

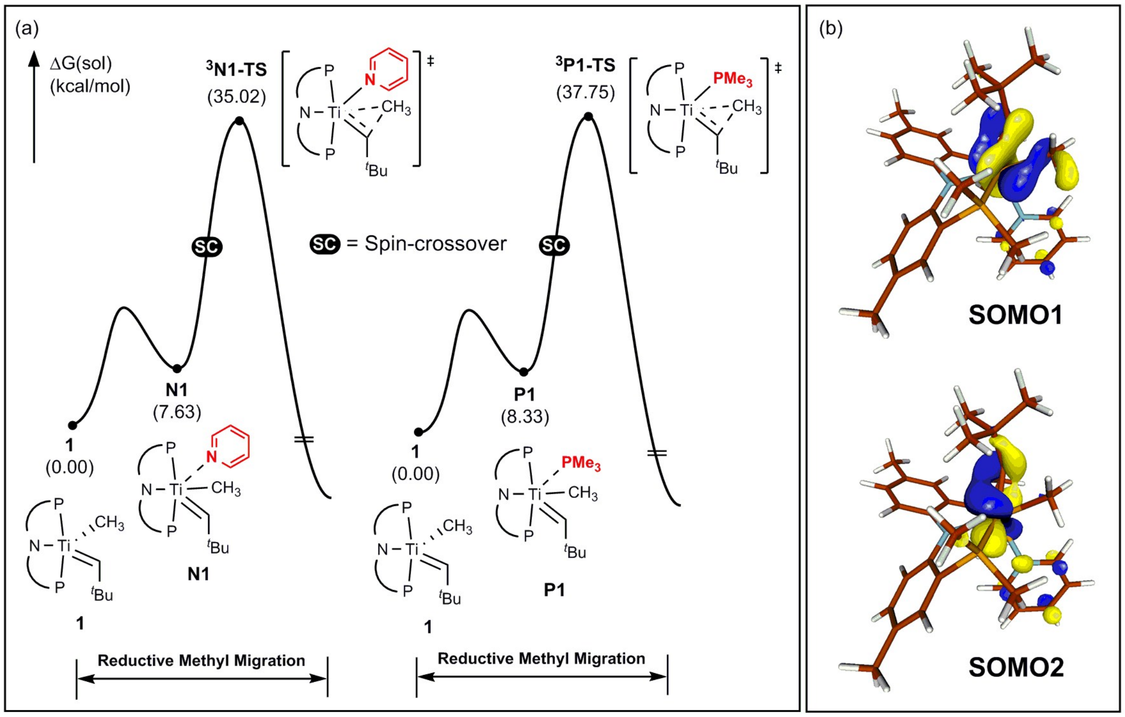

Figure S47. (a) Free energy profile for the reductive methyl migration step using pyridine and trimethylphosphine ligands. (b) Isosurface plots of singly occupied molecular orbitals of ^{3}**N1-TS**. SOMO1 and SOMO2 are represented. (isodensity value = 0.05)

Free energy profile using thioxanthone and xanthone as an additive ligand

Comparing the energy surface using thioxanthone and xanthone is proceeded to rationalize the chemoselectivity between Wittig-like reaction and $H_2C{=}CH^tBu$ formation. Especially, we focused on 2.0 kcal/mol energy difference of reductive methyl migration step.

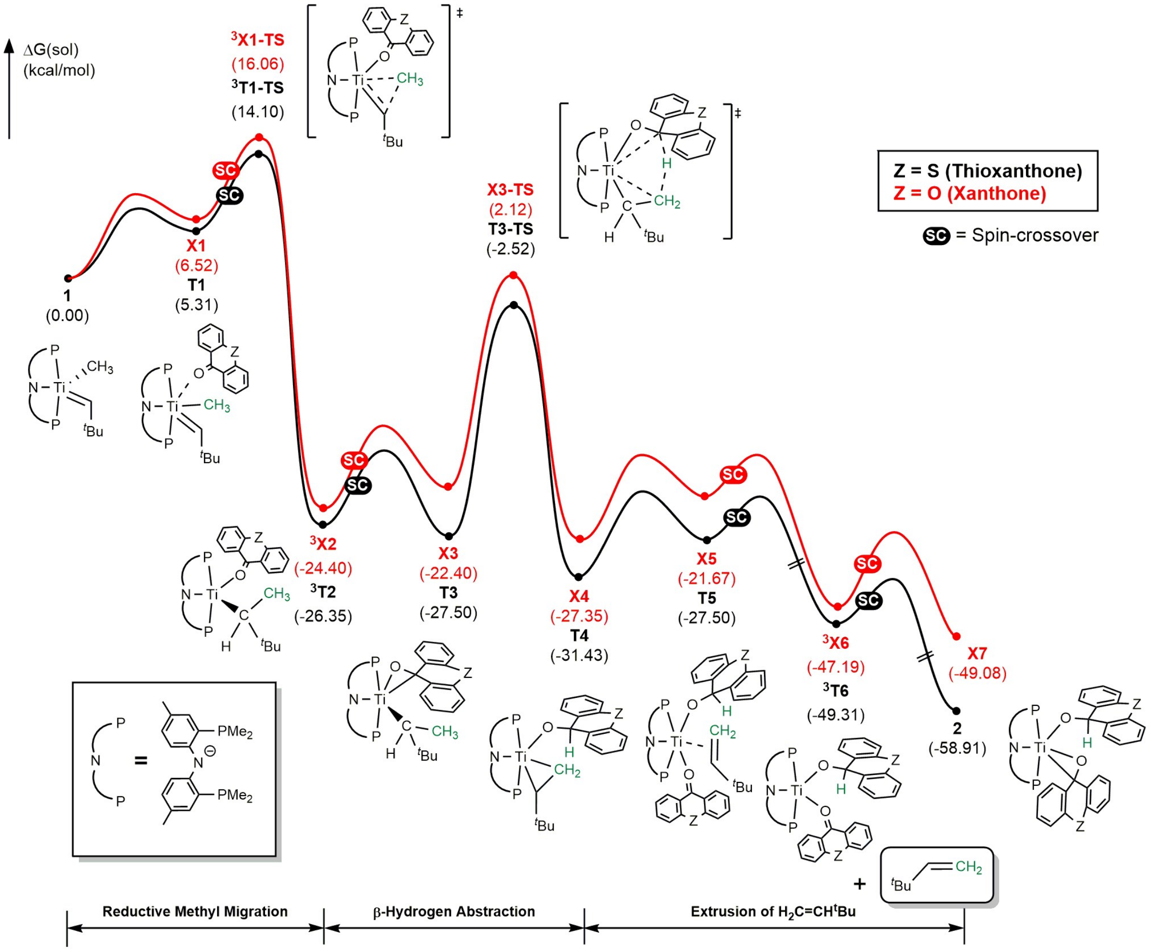

Figure S48. Free energy profile for formation of $H_2C{=}CH^tBu$ using thioxanthone and xanthone ligands. Black and red traces represent the thioxanthone and xanthone cases respectively.

Fragment analysis for the formation of four-membered (oxa)metallacycle step using thioxanthone and xanthone.

We evaluated the energies of the Ti-alkylidene and (thio)xanthone fragments independently. Even though the interaction energy of thioxanthone is stronger than xanthone, the electronic energy difference of 2.3 kcal/mol between **W2-TS** and **W2'-TS** comes from highly distorted two fragments of thioxanthone.

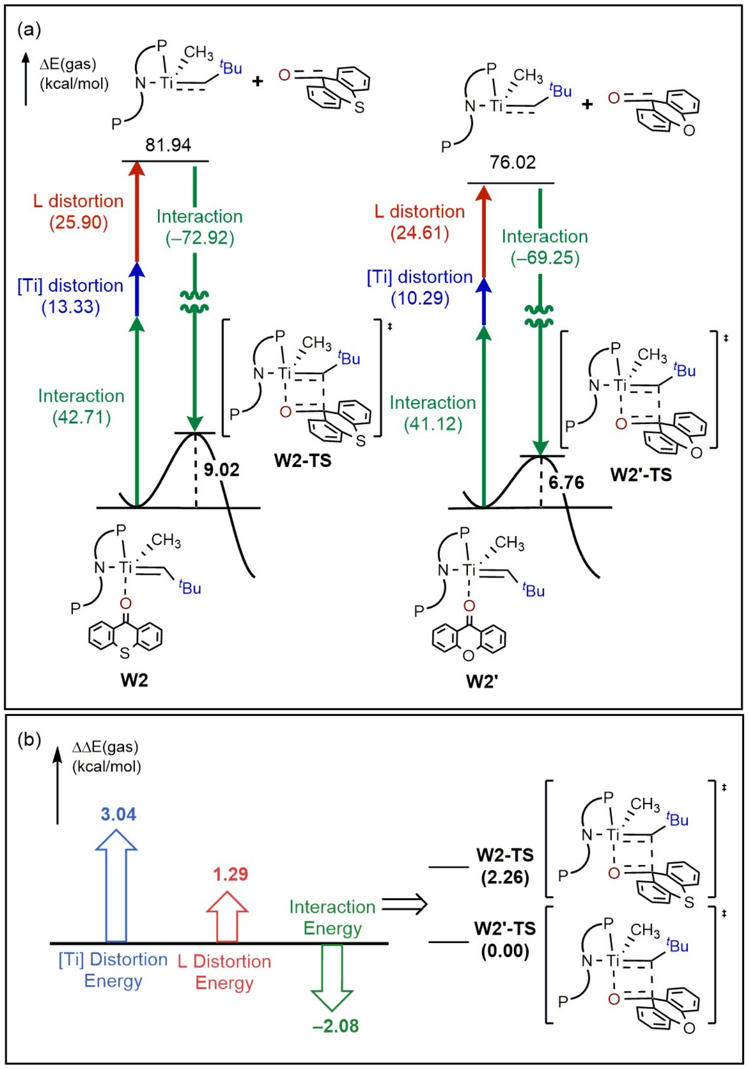

Figure S49. Fragment analysis for the formation of four-membered (oxa)metallacycle step using thioxanthone and xanthone. (a) Single point energy calculations for the fragments and interaction energy. (b) Simplification of electronic energy difference (ΔΔE (kcal/mol)) between **W2-TS** and **W2'-TS**.

Table S3. Computed energy components for optimized structures

	E(SCF)/(eV)	ZPE/(kcal/mol)	S(gas)/(cal/mol)	G(solv)/(kcal/mol)
	cc-pVTZ(-f)/LACV3P**	6-31G**/LACVP**	6-31G**/LACVP**	6-31G**/LACVP**
**thioxanthone**	-26500.041	107.837	103.476	-3.39
**H_2C=CH^{t}Bu**	-6420.425	103.810	81.829	-0.74
**xanthone**	-17711.482	109.884	99.294	-3.65
**^{t}BuHC=$C_{13}H_8S$ (W6)**	-29802.604	193.251	129.139	-2.31
**^{t}BuHC=$C_{13}H_8O$**	-21013.883	195.156	125.484	-2.36
**1**	-47154.910	342.904	217.945	-3.52
**T1**	-73655.492	452.424	275.036	-4.64
**^{3}T1-TS**	-73655.195	451.782	267.843	-4.20
**^{3}T2**	-73657.016	454.905	272.744	-4.32
**B**	-47154.683	342.920	213.927	-3.49
**B1**	-73655.008	452.489	267.903	-4.60
**^{3}B1-TS**	-73654.568	451.688	258.760	-4.58
**^{3}B2**	-73657.068	454.590	278.40	-4.57
**T3**	-73657.266	456.217	261.326	-4.42
**T3-TS**	-73656.203	453.605	252.120	-4.08
**T4**	-73657.391	455.929	264.463	-4.24
**T5**	-100158.117	565.447	314.145	-5.63
**^{3}T6**	-93737.789	459.705	286.552	-6.36
**2**	-93738.289	460.046	283.491	-5.68

**^{3}T2-TS**	-73655.656	451.970	268.498	-4.75
**^{3}T8**	-67235.352	346.625	239.098	-7.92
**W2-TS**	-73655.328	453.314	258.789	-4.22
**W5**	-43854.203	259.132	193.249	-7.65
**W2**	-73655.719	452.419	265.747	-4.53
**W3**	-73656.117	453.934	268.510	-4.91
**W3-TS**	-73655.633	453.683	262.514	-5.33
**W4**	-43853.578	257.637	187.944	-7.39
**W2’**	-64867.098	454.340	264.630	-4.74
**W2’-TS**	-64866.805	455.467	255.872	-4.40
**W3’**	-64867.590	456.087	262.489	-5.08
**W3’-TS**	-64867.117	455.582	260.520	-5.56
**W4’**	-43853.578	257.637	187.944	-7.39
**W5’**	-43854.203	259.132	193.249	-7.65
**X1**	-64866.934	454.535	267.652	-4.68
**^{3}X1-TS**	-64866.527	453.696	265.647	-4.29
**^{3}X2**	-64868.395	456.790	266.908	-4.39
**Y1**	-60639.992	444.215	259.689	-4.76
**^{3}Y1-TS**	-60639.414	443.953	255.605	-3.69
**^{3}Y2**	-60641.367	443.528	256.468	-3.79

**^{3}Y2-TS**	-60640.191	443.640	255.140	-4.67
**^{3}Y3**	-54219.703	338.354	225.916	-7.07
**^{3}Y4**	-67705.750	440.257	261.486	-4.40
**^{3}Y4-TS**	-67704.727	438.401	264.345	-4.79
**Y5**	-67706.313	441.992	263.926	-4.64
**N1**	-53913.824	400.790	239.436	-3.90
**^{3}N1-TS**	-53912.600	400.090	241.060	-3.55
**P1**	-59704.821	416.170	244.693	-3.23
**^{3}P1-TS**	-59703.505	416.010	247.404	-3.19
**X3**	-64868.488	458.369	257.345	-4.67
**X3-TS**	-64867.383	455.849	252.967	-4.42
**X4**	-64868.602	457.631	263.200	-4.51
**X5**	-82580.766	569.821	305.880	-5.94
**^{3}X6**	-76160.531	462.762	282.483	-5.67
**X7**	-76160.703	463.713	277.19	-6.12
**L(T)**	-26500.033	–	–	–
**[Ti](T)**	-47154.664	–	–	–
**L*(T)**	-26499.646	–	–	–

**[Ti]*(T)**	-47152.648	–	–	–
**L*$^{\cdot -}$(T)**	-26500.613	–	–	–
**[Ti]*$^{\cdot +}$(T)**	-47148.160	–	–	–
**L(X)**	-17711.471	–	–	–
**[Ti](X)**	-47154.609	–	–	–
**L*(X)**	-17711.123	–	–	–
**[Ti]*(X)**	-47152.590	–	–	–
**L*$^{\cdot -}$(X)**	-17711.977	–	–	–
**[Ti]*$^{\cdot +}$(X)**	-47148.158	–	–	–

Table S4. Cartesian Coordinates of the Optimized Geometries

The cartesian coordinates of optimized geometries are given below in the standard XYZ format (units are in Å).

==============================

thioxanthone

==============================

C	2.076985359	-0.294294596	2.214938164
C	1.458753824	-0.254692554	0.977279484
C	1.688114882	-1.266507030	3.147952795
H	1.725795388	0.483602673	0.229152858
H	2.166275740	-1.310780168	4.122246742
C	0.445693612	-1.171654224	0.632449090
C	0.694777787	-2.181734562	2.833971024
H	0.401036918	-2.936660767	3.557798147
C	0.068876147	-2.145570040	1.574647665
H	2.855975866	0.420200944	2.462066650
C	-1.715068102	-3.029444456	-0.349419355
C	-1.206313968	-1.992401838	-1.151846409
C	-2.721084118	-3.874829292	-0.851467669
H	-3.110445023	-4.674765587	-0.228066340
C	-1.717306614	-1.834642768	-2.455754519
C	-3.207445145	-3.696369886	-2.137829542
H	-1.304480553	-1.025828719	-3.048675776
H	-3.980671644	-4.360583305	-2.513056040
C	-2.703329325	-2.670504808	-2.950403690
H	-3.083952188	-2.533641577	-3.957747698
S	-1.184056044	-3.355422735	1.300080776
C	-0.152549163	-1.036307573	-0.721791744
O	0.225951627	-0.144449055	-1.482136965

==============================

H_2C=CH^tBu

==============================

C	1.338196635	-6.305143356	-1.161465049
H	0.269997835	-6.487638474	-1.225767374
H	1.677309275	-5.726794243	-0.306903541
C	2.200335741	-6.748738289	-2.076828003
H	3.260164976	-6.522222042	-1.940631390
C	1.902863026	-7.564217567	-3.322694778
C	2.393575191	-6.766952038	-4.553447723
H	1.846245408	-5.824297905	-4.655390263
H	3.460005283	-6.528404713	-4.472064495
H	2.250340939	-7.346367836	-5.472938061
C	2.699069500	-8.887423515	-3.234677553
H	2.560359240	-9.484020233	-4.143751144
H	3.772271395	-8.697388649	-3.120743513
H	2.371677637	-9.486757278	-2.379119635
C	0.406212181	-7.876099586	-3.478819132
H	0.233424038	-8.465633392	-4.385481358
H	0.027239792	-8.453041077	-2.628689766
H	-0.186723381	-6.958860397	-3.558665037

==============================

xanthone

==============================

C	1.964725733	-0.455479711	2.314786673
C	1.452814460	-0.282603562	1.039005756
C	1.469792604	-1.487265110	3.130409241
H	1.806752563	0.502164900	0.378130823
H	1.869371772	-1.625433683	4.130708218
C	0.442729712	-1.128739953	0.550804436
C	0.471810967	-2.337384701	2.672425747
H	0.074135996	-3.141188145	3.281964540
C	-0.034518469	-2.152249813	1.382887483
H	2.744828939	0.202332571	2.684725285
C	-1.556811333	-2.908796310	-0.260898948
C	-1.150946259	-1.921433806	-1.171140790
C	-2.548531532	-3.837592840	-0.589327753
H	-2.831335306	-4.583687782	0.144957736
C	-1.763369441	-1.880714059	-2.435282469
C	-3.137238979	-3.775392056	-1.845448256
H	-1.433466554	-1.108248234	-3.122547626
H	-3.907671452	-4.494101524	-2.108652115
C	-2.746675730	-2.795804739	-2.774411440
H	-3.215014935	-2.758001328	-3.752849817
O	-1.017007470	-3.022776604	0.992289782
C	-0.103407964	-0.947348416	-0.808117509
O	0.281667769	-0.063320979	-1.570487022

==============================

^tBuHC=$C_{13}H_8S$

==============================

C	-1.657631278	0.247507572	-3.346923113
H	-2.011581421	0.967349529	-4.085279942
C	-1.182955384	-1.049747705	-3.989352226
C	-1.799422622	0.648348808	-2.067214727
C	-1.393186688	-2.304558277	-3.119256496
C	-2.022324800	-1.228175163	-5.279294968
C	0.304597586	-0.922401071	-4.392150879
H	-0.721470475	-2.343771696	-2.262187481
H	-2.419375181	-2.344930649	-2.740356445
H	-1.216107130	-3.201338053	-3.723115444
H	-1.718818188	-2.135502100	-5.812197208
H	-3.088410616	-1.312591314	-5.043448448
H	-1.893115401	-0.379430711	-5.960491180
H	0.623003304	-1.806107521	-4.957422733
H	0.470659554	-0.041502848	-5.021856308
H	0.950054944	-0.834708750	-3.513478994
C	-2.934595823	4.283651829	-2.239322662
C	-2.213661671	3.115523815	-2.478677511
C	-3.953213453	4.290042877	-1.284270763
H	-2.696113110	5.188827038	-2.790005207
H	-1.407850027	3.110192060	-3.207128763
C	-2.503034592	1.931918859	-1.786100030
H	-4.513245583	5.199337482	-1.086677194
C	-4.235090256	3.133742809	-0.558577955
C	-3.512268066	1.961794734	-0.803548872

H	-5.006405354	3.140432119	0.205775678
S	-3.920129299	0.481798917	0.113367200
C	-1.381348372	-0.084458932	-0.845834255
C	-2.288046360	-0.232226118	0.222502351
C	-1.922326088	-0.928951204	1.378557563
C	-0.075062640	-0.564468622	-0.674912930
H	-2.635957479	-1.035277486	2.190079212
C	-0.637563407	-1.456070900	1.496482253
H	0.649003863	-0.387636542	-1.463245988
C	0.294956684	-1.255339384	0.476396561
H	-0.356763780	-1.992294908	2.398109913
H	1.308966637	-1.629463315	0.581508040

==============================

$^{t}BuHC{=}C_{13}H_8O$

==============================

C	-1.652232409	0.217608631	-3.395573139
H	-2.043715954	0.927393317	-4.122943878
C	-1.172825336	-1.070207953	-4.060487270
C	-1.774229050	0.627538145	-2.112397909
C	-1.513674140	-2.346380949	-3.262400389
C	-1.915524840	-1.164188385	-5.416839600
C	0.343122691	-1.004326582	-4.361066341
H	-0.964717984	-2.426550150	-2.324848652
H	-2.581949472	-2.375220537	-3.025003433
H	-1.274967074	-3.230261803	-3.864493370
H	-1.617036223	-2.067999601	-5.958490372
H	-2.999930620	-1.200058818	-5.267517567
H	-1.690355420	-0.301213533	-6.054168701
H	0.654582679	-1.884534240	-4.936076164
H	0.586463451	-0.112961844	-4.949039936
H	0.942549646	-0.972189248	-3.447812319
C	-3.277525663	4.139626980	-2.328614473
C	-2.492282391	3.030032873	-2.631259918
C	-4.085758686	4.133956432	-1.187389970
H	-3.255597591	5.010073662	-2.977291346
H	-1.856526732	3.039947748	-3.511510372
C	-2.498545885	1.890629292	-1.813761711
H	-4.700892925	4.995908737	-0.946869433
C	-4.085520744	3.029003143	-0.340448320
C	-3.285992622	1.931102514	-0.651990116
H	-4.683153152	2.998440742	0.564474344
O	-3.313774586	0.872412503	0.230682984
C	-1.369345069	-0.070711851	-0.871252775
C	-2.202025175	0.063165270	0.254623920
C	-1.940063238	-0.605204344	1.449547291
C	-0.185395703	-0.804679155	-0.705665827
H	-2.631507158	-0.482262641	2.276380539
C	-0.791130126	-1.382910371	1.559535027
H	0.517266691	-0.848378837	-1.527557015
C	0.102197058	-1.462701440	0.487092376
H	-0.578561783	-1.900101423	2.490432739
H	1.022346377	-2.030899763	0.582212865

==============================

1

==============================

Ti	-0.279676557	-0.697261035	0.632165492
P	-2.624582291	0.189726278	0.171521708
P	2.191471338	-0.923788130	-0.011438298
N	-0.271437317	-0.406685293	-1.415385008
C	-2.592639685	-1.964519382	-3.901124477
C	-3.838232517	-1.801389933	-3.276818037
C	-1.416731238	-1.498338819	-3.325101614
H	-0.466874719	-1.658744574	-3.824740410
C	-3.859986782	-1.137286067	-2.047225237
C	-1.433695674	-0.827273965	-2.086894035
H	-4.815139771	-0.999884188	-1.545538902
C	-2.692232609	-0.646925747	-1.454280019
H	-2.542558432	-2.483985424	-4.855881214
C	1.800350428	1.307841301	-4.015838146
C	0.684877098	0.799198866	-3.361298323
C	3.098037720	1.156095505	-3.503255129
H	-0.303343862	0.946555495	-3.784939051
C	0.816435099	0.098547868	-2.143714190
C	3.229496956	0.473043263	-2.291515589
H	4.223915577	0.345988035	-1.867565513
C	2.123266220	-0.048952345	-1.613987446
H	1.660022020	1.849164367	-4.949742794
C	-5.101624966	-2.354891062	-3.893084288
H	-5.062532425	-2.320837498	-4.986973286
H	-5.258232594	-3.402850866	-3.606773615
H	-5.985209465	-1.794247985	-3.570798397
C	4.293081284	1.713093281	-4.240861893
H	5.213484287	1.596921802	-3.660378933
H	4.443279743	1.207611561	-5.203139305
H	4.169112682	2.781076193	-4.457001686
C	-2.875201225	1.978264689	-0.239716306
H	-3.816103220	2.137474060	-0.775662780
H	-2.871488333	2.573439360	0.678571999
H	-2.041603804	2.302110434	-0.868442476
C	-4.191090584	-0.260180056	1.029154778
H	-5.076771259	0.073753305	0.478970110
H	-4.222019672	-1.344457030	1.156451702
H	-4.195133686	0.205171749	2.018877029
C	3.515504599	-0.069054335	0.945997536
H	3.642519712	-0.580244184	1.904372096
H	4.472926617	-0.060703196	0.416720212
H	3.196943760	0.956850588	1.143829346
C	2.959474564	-2.553401232	-0.419228226
H	3.110080004	-3.124059200	0.501623094
H	2.268856287	-3.108173847	-1.059040189
H	3.915348530	-2.426820993	-0.937547386
C	0.297345221	0.655593514	2.181564569
H	-0.570099771	0.873921394	2.824865341
H	1.108732462	0.323549509	2.843552351
H	0.612812936	1.607030392	1.721446037
C	-0.719999850	-2.388764143	1.232316136
H	-0.502014816	-2.102392435	2.294157267
C	-1.144066930	-3.823879957	1.043713331
C	0.021860924	-4.758960724	1.440606475
H	0.897947311	-4.565337658	0.813374758

H	0.312736273	-4.599347115	2.485188484
H	-0.260422289	-5.813420773	1.322377563
C	-2.369459152	-4.144309998	1.928048015
H	-2.683989525	-5.188583374	1.803943157
H	-2.141884089	-3.980655670	2.987589121
H	-3.215162992	-3.500889301	1.662242651
C	-1.505669713	-4.065484524	-0.436502010
H	-1.775286555	-5.114552975	-0.611970365
H	-2.347940445	-3.437022209	-0.740701258
H	-0.660181224	-3.813079596	-1.085083485

==============================

T1

==============================

Ti	-0.379730105	-1.049897313	0.730397999
P	-2.753992319	-0.165769517	0.375921041
P	2.069800138	-1.159393311	0.065030344
N	-0.371665686	-0.478088140	-1.323436856
C	-2.746194124	-1.518559575	-4.024260521
C	-3.983958960	-1.467355371	-3.366415739
C	-1.560809851	-1.183014870	-3.383537054
H	-0.622183621	-1.275883794	-3.918710709
C	-3.978168011	-1.058399916	-2.031147242
C	-1.544611812	-0.758098364	-2.035990238
H	-4.925378323	-1.011827946	-1.498272896
C	-2.798488379	-0.701387823	-1.369770765
H	-2.708965540	-1.848179579	-5.060925007
C	1.790257335	1.379603148	-3.747980118
C	0.649720728	0.876809657	-3.136189699
C	3.081639767	1.089703083	-3.274615288
H	-0.327021837	1.148995161	-3.521491528
C	0.733916104	0.032832798	-2.001186848
C	3.170944929	0.287113637	-2.135354519
H	4.154775620	0.065744251	-1.724843025
C	2.035832882	-0.229418606	-1.500711083
H	1.677318573	2.032351732	-4.612093925
C	-5.260890484	-1.870485425	-4.064655781
H	-5.266670704	-1.546310425	-5.110904694
H	-5.390329361	-2.960340977	-4.065292835
H	-6.140039444	-1.438920617	-3.575606823
C	4.307385445	1.634559631	-3.970294952
H	5.215490341	1.445102334	-3.389461279
H	4.447323799	1.178259134	-4.958620548
H	4.235782146	2.718057871	-4.130188942
C	-3.170275450	1.636845589	0.252137929
H	-4.100857258	1.781317949	-0.305459440
H	-3.266616344	2.078893900	1.247465611
H	-2.350189924	2.132577896	-0.272219062
C	-4.268629551	-0.861862123	1.164123535
H	-5.188246250	-0.521553338	0.677548945
H	-4.213209152	-1.951809287	1.133776546
H	-4.291809559	-0.543715119	2.210856199
C	3.281800508	-0.225807354	1.098840356
H	3.352776527	-0.699828744	2.081274271
H	4.274842739	-0.182542220	0.640692055
H	2.897662401	0.788381398	1.231378913
C	2.974107742	-2.721156597	-0.324065268
H	3.110749006	-3.298506498	0.595004916
H	2.362720728	-3.308611393	-1.013332367
H	3.949505329	-2.520377874	-0.779778600
C	0.382059753	-0.825054824	2.761788845
H	-0.479269892	-0.868155301	3.452320337
H	1.053096652	-1.638427138	3.070768595
H	0.898651838	0.125077069	2.940263271
C	-0.853004694	-2.835819006	0.649099588
H	-0.726903081	-2.860427380	1.762379885
C	-1.197256088	-4.192639351	0.084513187
C	-0.185240313	-5.237671375	0.610310376
H	0.833789170	-4.968767643	0.312368155
H	-0.210905969	-5.293962479	1.704847455
H	-0.406032741	-6.236953735	0.212231174
C	-2.622823000	-4.600845337	0.523635983
H	-2.879675865	-5.600943089	0.149636537
H	-2.709979057	-4.612679482	1.616349459
H	-3.357826948	-3.892400265	0.127190962
C	-1.138720036	-4.162101269	-1.453743815
H	-1.312113404	-5.161819458	-1.872019529
H	-1.893242836	-3.485429049	-1.858610034
H	-0.160657242	-3.807596922	-1.796227574
C	-2.059653997	2.313718319	4.307207108
C	-1.336626887	2.049546957	3.156978369
C	-1.978373885	3.584740162	4.894620419
H	-1.372475863	1.075247407	2.691439390
H	-2.538713694	3.807728767	5.798223972
C	-0.521541059	3.028071880	2.551521540
C	-1.180795193	4.566958427	4.327314854
H	-1.120036125	5.550173283	4.786031246
C	-0.447819114	4.300288200	3.156280518
H	-2.676977873	1.539800763	4.752596855
C	1.243346810	4.973342419	1.079653740
C	1.041720867	3.654125214	0.620245636
C	2.060550213	5.856360912	0.352196753
H	2.210167885	6.871010780	0.711457610
C	1.670753479	3.260137558	-0.577980578
C	2.669256687	5.437665462	-0.822080910
H	1.503966689	2.251962900	-0.929033637
H	3.295372248	6.132153511	-1.375631809
C	2.473257780	4.132275105	-1.292837977
H	2.938661575	3.795493126	-2.212916613
S	0.526429176	5.630237103	2.546179295
C	0.197783083	2.652377367	1.312105656
O	0.081778720	1.512620568	0.836959481

==============================

^{3}T1-TS

==============================

Ti	0.023823040	-0.635112882	0.594692409
P	-2.189459562	0.710673332	-0.062069926
P	2.499764681	-1.000085115	-0.068571448
N	0.069034413	-0.462223053	-1.504180312
C	-2.453028679	-1.677813053	-3.989351511
C	-3.652761459	-1.170805097	-3.468963385

C	-1.230582237	-1.442445517	-3.368029833
H	-0.318315297	-1.851324201	-3.791632175
C	-3.576040745	-0.416242540	-2.294044256
C	-1.149934292	-0.676777661	-2.188999653
H	-4.494758129	-0.020631226	-1.865933418
C	-2.358138323	-0.169940695	-1.653037548
H	-2.478800535	-2.280015707	-4.895346642
C	2.244534731	0.969458878	-4.190383911
C	1.101795316	0.563208878	-3.515566587
C	3.536108017	0.764876962	-3.673321486
H	0.127630740	0.762873709	-3.948113918
C	1.185554266	-0.085138306	-2.259458542
C	3.625182629	0.138892666	-2.430169582
H	4.607879162	-0.019740015	-1.988786221
C	2.486666441	-0.272819996	-1.726536155
H	2.133147240	1.477223992	-5.147017956
C	-4.978750229	-1.467642069	-4.129584312
H	-4.900057316	-1.436747313	-5.221740723
H	-5.343480110	-2.467656374	-3.860688210
H	-5.746988773	-0.748605788	-3.827917099
C	4.757568836	1.219863415	-4.436539173
H	5.671013355	1.088783741	-3.848247051
H	4.882161617	0.657258868	-5.370537281
H	4.687870979	2.279841661	-4.709970474
C	-2.301023960	2.497702360	-0.538619101
H	-3.196418047	2.676880598	-1.142643094
H	-2.337169647	3.114413023	0.363382161
H	-1.412619948	2.775723696	-1.109311223
C	-3.798754215	0.459530145	0.807351232
H	-4.634480953	0.922406614	0.273281306
H	-3.988092422	-0.610469878	0.916948378
H	-3.720217943	0.907732785	1.802450657
C	3.689492464	0.008658483	0.910783827
H	3.789880991	-0.429091156	1.907881618
H	4.674084663	0.058173545	0.436431527
H	3.275972128	1.013566256	1.015996456
C	3.356076241	-2.626655102	-0.232719630
H	3.431997299	-3.097785473	0.751794159
H	2.768992424	-3.273447037	-0.889085710
H	4.358043194	-2.500753164	-0.654656649
C	0.753430605	-1.925261617	2.334970713
H	0.054975267	-2.089929104	3.159865141
H	1.181067586	-2.890108585	2.052271366
H	1.548913956	-1.268141985	2.708987713
C	-1.353605151	-1.991105437	1.074372649
H	-2.070510149	-1.749630928	1.873329282
C	-1.525869846	-3.422881842	0.609677315
C	-1.558833241	-4.397784233	1.808976889
H	-0.612156689	-4.387154102	2.355520010
H	-2.357763052	-4.128787041	2.509559155
H	-1.742003679	-5.422859192	1.463613868
C	-2.892188787	-3.485383272	-0.123411581
H	-3.087116480	-4.511518955	-0.459826708
H	-3.711727619	-3.188981771	0.541914761
H	-2.902778625	-2.828577280	-0.996143341
C	-0.416668355	-3.844636202	-0.374896288
H	-0.586732030	-4.864382267	-0.739258707
H	-0.386725903	-3.174092054	-1.238631606
H	0.563154995	-3.820532799	0.110880829
C	-2.258553743	1.262972116	4.593214512
C	-1.296614170	1.167782187	3.597874641
C	-2.772320986	2.514950514	4.954842091
H	-0.891897261	0.201921001	3.320687056
H	-3.530722141	2.600442171	5.727364063
C	-0.807126999	2.315083504	2.919493198
C	-2.300298691	3.663154840	4.316984177
H	-2.692815304	4.637840748	4.595712662
C	-1.323731184	3.577647448	3.321918964
H	-2.608030796	0.363069266	5.091643810
C	0.181687683	4.601101875	1.217636347
C	0.542596936	3.247924805	0.980831265
C	0.553524733	5.604962349	0.320315331
H	0.253450543	6.631710052	0.514287055
C	1.283880949	2.970500946	-0.195382684
C	1.292672753	5.300529480	-0.823897541
H	1.538084626	1.942570448	-0.412746429
H	1.569799900	6.093051434	-1.512903929
C	1.654590607	3.972626925	-1.079454899
H	2.211126328	3.711737156	-1.975358963
S	-0.728577673	5.109165668	2.653328180
C	0.128659457	2.162413359	1.834775925
O	0.541856766	0.938627064	1.527698755

================================

^{3}T2

================================

Ti	-0.531926513	-0.270791203	0.479830652
P	-2.019599199	-2.383060694	0.231679797
P	0.697551310	1.811321378	-0.372700602
N	-0.322395802	-0.698780775	-1.530249238
C	-2.730323792	-1.590282559	-4.249177933
C	-3.651288986	-2.409338713	-3.580312729
C	-1.615566611	-1.061214447	-3.610517979
H	-0.951487184	-0.401487023	-4.158800125
C	-3.397274017	-2.677167654	-2.234317303
C	-1.355504036	-1.325382948	-2.246901989
H	-4.105001926	-3.294419527	-1.684537768
C	-2.284940004	-2.156458378	-1.564465761
H	-2.904313564	-1.338683367	-5.293946743
C	2.558676958	-0.491286904	-3.908384085
C	1.422592521	-0.965563476	-3.265027761
C	3.168866396	0.716304541	-3.533048153
H	0.979886413	-1.907308340	-3.573554516
C	0.820075989	-0.242249712	-2.213786125
C	2.597783327	1.418492317	-2.470081806
H	3.063362360	2.348259211	-2.150668621
C	1.448672056	0.965532482	-1.810706139
H	2.989237070	-1.075749040	-4.718924522
C	-4.862416744	-2.967882395	-4.288007736
H	-5.305716038	-2.227162123	-4.962976933
H	-4.609331608	-3.846527338	-4.895407677
H	-5.635693550	-3.273019314	-3.576001644

C	4.404427528	1.217434764	-4.242770672
H	4.673305988	2.226517439	-3.915208101
H	5.268036366	0.567601025	-4.053919315
H	4.257320404	1.248111963	-5.328734875
C	-3.711603880	-2.333726883	0.968470395
H	-4.335330009	-3.162083149	0.620714903
H	-3.626783133	-2.383299828	2.057524204
H	-4.187964916	-1.388966084	0.702236116
C	-1.572550058	-4.168944359	0.397235483
H	-2.339332342	-4.800109863	-0.063129835
H	-0.614399016	-4.344115734	-0.095724143
H	-1.475629926	-4.427566051	1.455871105
C	-0.307060421	3.189389229	-1.090242743
H	-0.745243490	3.776583195	-0.277074695
H	0.307466567	3.837969780	-1.721844316
H	-1.122535944	2.769895792	-1.683928013
C	2.082000971	2.712220192	0.446411818
H	1.687843204	3.223119974	1.329266906
H	2.832793951	1.989350557	0.773662865
H	2.549115419	3.450658083	-0.212039337
C	1.486784458	-2.438118935	1.138422370
H	2.497608185	-2.771802664	1.417087674
H	0.783292830	-3.162770510	1.562207341
H	1.418311596	-2.528866529	0.046029288
C	1.171320319	-1.002417684	1.596468568
H	2.024822950	-0.376969218	1.271030545
C	1.062576652	-0.829436183	3.138287067
C	-0.225705504	-1.485512853	3.685157776
H	-1.126053929	-1.019733787	3.270400047
H	-0.262688488	-2.553301573	3.441716671
H	-0.279716223	-1.394977450	4.777424812
C	2.271882772	-1.455229998	3.868850231
H	2.235195398	-1.246518373	4.945852757
H	2.299932241	-2.542617083	3.744768143
H	3.212511301	-1.049424529	3.478047609
C	1.038936019	0.677056313	3.474193811
H	0.897601783	0.845126092	4.549045086
H	1.982271671	1.152602077	3.182337046
H	0.234255001	1.198488712	2.944135666
C	-4.183093548	1.200946927	-2.290991306
C	-3.446106911	1.073854923	-1.122869849
C	-5.541802883	1.523926616	-2.228738785
H	-2.394306898	0.832530677	-1.190538764
H	-6.128767490	1.630744696	-3.136173725
C	-4.028943062	1.241934776	0.157694936
C	-6.146511078	1.710068345	-0.986673295
H	-7.201436520	1.966220021	-0.929686308
C	-5.414505482	1.563880920	0.194295600
H	-3.695542097	1.031817913	-3.245439053
C	-5.062113285	1.766144991	2.942047834
C	-3.712378263	1.413864493	2.674294472
C	-5.467849731	2.111215591	4.234140873
H	-6.504180431	2.387125492	4.411810875
C	-2.813610077	1.417880297	3.769057274
C	-4.558688164	2.102996588	5.290422916
H	-1.782144547	1.157696962	3.579022884
H	-4.889195919	2.372344732	6.289265156
C	-3.225446224	1.751833558	5.049789429
H	-2.505722046	1.744899035	5.863410473
S	-6.326273918	1.725080013	1.703554630
C	-3.233465672	1.081097126	1.354353786
O	-1.979447007	0.662704587	1.239441633

================================

B

================================

Ti	-0.371275246	-0.242767826	0.570157826
P	-2.747959852	0.496922761	-0.080000058
P	2.009877920	-0.975264370	-0.032282010
N	-0.466571838	-0.335679173	-1.484953165
C	-2.750376940	-1.893181324	-4.007720947
C	-4.007607937	-1.637263775	-3.443577528
C	-1.570163369	-1.465173006	-3.403026104
H	-0.609749079	-1.684201121	-3.859555006
C	-4.045315266	-0.910975754	-2.246231318
C	-1.608243704	-0.749297976	-2.195142508
H	-5.010498047	-0.689406276	-1.795516372
C	-2.876470566	-0.464088589	-1.630495667
H	-2.691921473	-2.452247620	-4.939343452
C	1.706298113	1.413567066	-3.968438148
C	0.572399855	0.874116898	-3.376734257
C	2.981034756	1.270996094	-3.395338535
H	-0.400519341	1.017997742	-3.836081505
C	0.658387065	0.150597587	-2.167808533
C	3.069904566	0.552732468	-2.202041626
H	4.047083855	0.428939432	-1.740535498
C	1.944185019	-0.010652602	-1.588761091
H	1.601145267	1.975417495	-4.894687653
C	-5.274539948	-2.150941849	-4.085943699
H	-5.199620724	-2.151638746	-5.178450584
H	-5.490704536	-3.181323290	-3.775280476
H	-6.140338421	-1.540511370	-3.809010267
C	4.198032379	1.876987576	-4.053877831
H	5.097854614	1.727771282	-3.449256897
H	4.384123802	1.435058594	-5.040659428
H	4.074790955	2.956333399	-4.204871178
C	-2.758692741	2.245298147	-0.680478096
H	-3.652918816	2.460158110	-1.274012685
H	-2.703687906	2.919841766	0.178239107
H	-1.864534259	2.393515587	-1.290575027
C	-4.388717175	0.347848982	0.747650802
H	-5.195571899	0.723753214	0.110662460
H	-4.581044197	-0.696362495	1.000201106
H	-4.367561340	0.932813108	1.671371460
C	3.457998037	-0.315399140	0.897618890
H	3.538011789	-0.852186501	1.847687721
H	4.398820877	-0.430147529	0.349724591
H	3.269180775	0.738646269	1.110011339
C	2.629034758	-2.632800579	-0.587346554
H	2.799908161	-3.277156115	0.280182034
H	1.871962309	-3.099474907	-1.222086906
H	3.560493708	-2.528848171	-1.152720094

C	0.292563379	1.372617006	1.253866792
H	0.352622747	1.529250264	2.341276407
H	-1.655332685	-0.977736175	2.782364130
H	0.692644835	2.228337288	0.695153773
C	-0.940210044	-1.523731947	2.142585278
H	0.019499943	-1.506494761	2.700741529
C	-1.394747734	-2.985175848	1.955608487
C	-0.412470579	-3.723785877	1.024154067
H	-0.387028873	-3.259893179	0.030435639
H	0.604440093	-3.694091082	1.432275653
H	-0.693565905	-4.776551247	0.896482170
C	-1.434584141	-3.715435505	3.315743446
H	-1.763786077	-4.757656574	3.207276583
H	-0.442320377	-3.722005606	3.782212973
H	-2.122675180	-3.212871790	4.005409241
C	-2.801170588	-3.026325226	1.330175281
H	-3.148338556	-4.058191776	1.196224689
H	-3.523745537	-2.513097763	1.975914836
H	-2.811802387	-2.539512396	0.348470718

===============================

B1

===============================

Ti	-0.317663103	-0.073558867	1.041032195
P	-2.670396328	0.900757134	0.539559424
P	2.086876869	-0.277322024	0.310239583
N	-0.435931563	-0.002775357	-1.156544805
C	-3.073932171	-1.500687957	-3.352199554
C	-4.261231899	-1.071297884	-2.734939814
C	-1.823372364	-1.162399650	-2.857271910
H	-0.935436010	-1.538613319	-3.353821039
C	-4.126793861	-0.319804281	-1.569077730
C	-1.673563242	-0.349581212	-1.705322266
H	-5.027673244	-0.002514028	-1.046759725
C	-2.874930143	0.029161960	-1.046241045
H	-3.135073662	-2.131245613	-4.237778187
C	1.695411801	0.809985876	-4.123782635
C	0.567879200	0.589292943	-3.348317623
C	2.998027086	0.656803012	-3.616210461
H	-0.410666853	0.765902162	-3.780397415
C	0.660550654	0.179936931	-1.990463734
C	3.105143547	0.286985725	-2.275473833
H	4.097344875	0.188459292	-1.836629033
C	1.981425166	0.059222657	-1.470566392
H	1.564964414	1.139295697	-5.153602600
C	-5.611523628	-1.430308938	-3.307060480
H	-5.782362938	-0.941729724	-4.274981499
H	-5.702654839	-2.510226011	-3.473966599
H	-6.424008846	-1.128082037	-2.638590097
C	4.211102009	0.894251704	-4.484605789
H	5.117556572	1.016708136	-3.882862091
H	4.387045383	0.060144145	-5.176610947
H	4.096741199	1.796253920	-5.098689556
C	-3.265148640	2.610901117	0.164906725
H	-4.260068893	2.548769712	-0.286786020
H	-3.308583498	3.216020107	1.072525740
H	-2.584456444	3.083840609	-0.545666754
C	-4.020277977	0.264486611	1.624265075
H	-5.013668060	0.425301641	1.194781065
H	-3.864207745	-0.803284585	1.790028214
H	-3.959562540	0.776193678	2.588981390
C	3.278681755	0.958928943	0.971440971
H	3.426369905	0.764945805	2.037117481
H	4.241207600	0.919777215	0.452448756
H	2.831442118	1.947353482	0.853322983
C	3.018821239	-1.862808228	0.462459624
H	3.175847769	-2.095062017	1.518917561
H	2.432284832	-2.665852308	0.010608000
H	3.985888958	-1.793251753	-0.045635235
C	0.222556800	0.449762255	2.739241838
H	-0.145113438	-0.525232077	3.129533768
H	-0.245146394	-2.446184158	0.234858721
H	0.767849028	1.002025366	3.507324457
C	-0.917520821	-2.106920004	1.047501087
H	-1.912792563	-2.083145380	0.568733037
C	-0.924647987	-3.164863586	2.168701410
C	-1.910397530	-2.762088537	3.281565428
H	-1.643196225	-1.796705246	3.722951174
H	-2.928406954	-2.676186800	2.882050037
H	-1.931996465	-3.509647369	4.084435940
C	-1.376343608	-4.523835659	1.586520195
H	-1.393875837	-5.306779861	2.356319904
H	-2.381956816	-4.449348450	1.156310558
H	-0.697645247	-4.848869324	0.789243102
C	0.487124205	-3.340782166	2.760233402
H	0.493861645	-4.099473476	3.552825689
H	1.190937400	-3.663570642	1.984190464
H	0.862554312	-2.402896404	3.180435896
C	-1.672534227	4.179517269	3.368508816
C	-0.890531659	3.603286266	2.380801916
C	-2.218796015	5.454959393	3.162446737
H	-0.496850342	2.598273516	2.497884512
H	-2.837619305	5.916758537	3.926895857
C	-0.633158505	4.271691799	1.166322589
C	-1.972362280	6.139964104	1.980572820
H	-2.393822193	7.129730701	1.827268243
C	-1.174255729	5.560397625	0.978084922
H	-1.863391161	3.640452862	4.291450500
C	0.185088068	5.561101913	-1.426806211
C	0.612740278	4.263280869	-1.072612524
C	0.648135006	6.155044556	-2.613399982
H	0.312597364	7.153170586	-2.882009029
C	1.512351394	3.597076893	-1.929342508
C	1.535090327	5.474081516	-3.435731173
H	1.827820420	2.599355459	-1.656822085
H	1.885728478	5.948256493	-4.348357677
C	1.973857045	4.187655449	-3.093753576
H	2.660324335	3.644131184	-3.733880997
S	-0.949445665	6.519451141	-0.479771346
C	0.161635116	3.552424431	0.145833001
O	0.462864786	2.361374378	0.311901927

================================

[3]B1-TS

================================

Ti	-0.777488708	0.482834220	0.625526845
P	-3.144768953	0.712729037	-0.303983361
P	1.719086170	-0.412955880	0.109997317
N	-0.693329394	-0.080880485	-1.425524473
C	-2.653692245	-2.131379843	-3.858284473
C	-3.973098278	-1.898133755	-3.437138319
C	-1.561876297	-1.542291522	-3.231281519
H	-0.555438578	-1.743369579	-3.584982872
C	-4.157385349	-1.035891533	-2.352834940
C	-1.744295239	-0.676265717	-2.133625746
H	-5.169622421	-0.830880284	-2.009259701
C	-3.072625637	-0.435564607	-1.708440542
H	-2.478763819	-2.801360607	-4.698037624
C	1.448719144	1.490473747	-4.061573505
C	0.333588332	0.937609792	-3.441885948
C	2.700422764	1.532094836	-3.432224274
H	-0.628104150	0.955323100	-3.944886684
C	0.423919111	0.385098755	-2.149964333
C	2.802863598	0.956079125	-2.162345886
H	3.763862610	0.976587594	-1.654579878
C	1.699149013	0.378461838	-1.530812144
H	1.336952567	1.934085727	-5.048686981
C	-5.137644291	-2.579124451	-4.116260529
H	-5.036174297	-2.554475069	-5.207100391
H	-5.211986065	-3.634690285	-3.824288845
H	-6.087818623	-2.100746632	-3.858614206
C	3.875572205	2.226041555	-4.075946808
H	4.827659607	1.866234779	-3.673109770
H	3.888804913	2.077682018	-5.161347866
H	3.827151299	3.306778193	-3.892835617
C	-3.581258535	2.347226620	-1.046032190
H	-4.563432217	2.308098793	-1.527174115
H	-3.575330257	3.120278120	-0.272686332
H	-2.826191425	2.593142748	-1.796164274
C	-4.650449276	0.259542882	0.649552047
H	-5.547364712	0.270206720	0.023253910
H	-4.518179893	-0.739486933	1.072807193
H	-4.776411057	0.972108126	1.468864441
C	3.388020754	-0.079393655	0.821635902
H	3.440830231	-0.533796906	1.814508319
H	4.176719666	-0.503381491	0.192273527
H	3.545610428	0.993798196	0.930210114
C	1.870458961	-2.195020199	-0.380949110
H	1.929320335	-2.844982386	0.495128810
H	0.986156106	-2.464260101	-0.963596106
H	2.762207270	-2.334931374	-0.999425590
C	-1.775595546	0.500927329	2.313654900
H	-2.724946022	0.124967679	2.690631628
H	-0.560573101	-2.092069387	0.639026105
H	-1.163480520	0.896932602	3.140275955
C	-1.089880466	-1.662559867	1.497957230
H	-2.153800964	-1.879230380	1.336089730
C	-0.605260611	-2.351251841	2.791997194
C	-1.612001657	-2.177511930	3.945905447
H	-1.662194848	-1.140456438	4.286210060
H	-2.619610786	-2.482856274	3.639030457
H	-1.322618604	-2.795398951	4.804408550
C	-0.467414916	-3.868191004	2.512907982
H	-0.127076238	-4.408591270	3.405680895
H	-1.429081798	-4.295798779	2.206668615
H	0.252023101	-4.060882568	1.709258914
C	0.760690928	-1.805911660	3.253900051
H	1.066026926	-2.268856525	4.200602531
H	1.546067595	-2.015807390	2.522304296
H	0.721114933	-0.721464694	3.397438288
C	2.619522333	2.530312061	3.863052368
C	1.722091794	2.487847567	2.807021856
C	3.876745224	3.123524666	3.695369959
H	0.741689920	2.045346260	2.935494184
H	4.583343029	3.164270163	4.518856525
C	2.045191050	3.024314404	1.535177231
C	4.218658447	3.674516916	2.459566593
H	5.189785480	4.143212795	2.323123217
C	3.325827122	3.625495434	1.387080312
H	2.339598417	2.107013226	4.823840618
C	2.404557943	4.414555550	-1.104702353
C	1.214530826	3.727147341	-0.750586033
C	2.458588123	5.203944206	-2.254443645
H	3.377224445	5.731613159	-2.497654676
C	0.103815556	3.859786749	-1.615005851
C	1.346025229	5.312823772	-3.090291023
H	-0.801851869	3.330681562	-1.347514272
H	1.401994586	5.925188065	-3.985470057
C	0.167450920	4.633760929	-2.763771296
H	-0.701707125	4.708807945	-3.411633730
S	3.906965733	4.218091011	-0.181451291
C	1.099293470	2.942301273	0.453727663
O	0.008698987	2.198584080	0.610901892

================================

[3]B2

================================

Ti	-0.956611753	0.096930049	0.752548754
P	-3.332324266	0.243377343	-0.231349722
P	1.492682457	-0.605241597	0.881484807
N	-0.575749695	-0.365894169	-1.215623736
C	-2.281506062	-2.454001427	-3.812110424
C	-3.640446186	-2.222815514	-3.547906637
C	-1.274253011	-1.847058058	-3.074148655
H	-0.238465369	-2.071310759	-3.303970575
C	-3.938115597	-1.365117669	-2.488870382
C	-1.566837549	-0.951782465	-2.017920256
H	-4.983289242	-1.182630777	-2.244925022
C	-2.943614721	-0.743480563	-1.722816229
H	-2.005164623	-3.137888670	-4.612720966
C	2.245529413	0.456782639	-3.537437439
C	0.958449721	0.223212346	-3.070489168
C	3.367925644	0.338242739	-2.702846766
H	0.113720998	0.342087239	-3.741273642

C	0.720328748	-0.161847115	-1.733457565
C	3.137337208	-0.006513126	-1.370267391
H	3.989017248	-0.082614161	-0.697861791
C	1.850799084	-0.253239751	-0.875705123
H	2.383877039	0.753827333	-4.575177670
C	-4.721543789	-2.917918444	-4.340651512
H	-4.479422569	-2.950031996	-5.408661842
H	-4.855807781	-3.956112862	-4.010183811
H	-5.687022209	-2.413933516	-4.231543541
C	4.761112213	0.583817959	-3.232039452
H	5.504918098	0.559441268	-2.429668427
H	5.048610210	-0.173388705	-3.972286701
H	4.836242199	1.559673667	-3.726464033
C	-4.293823242	1.695852160	-0.846709788
H	-5.138509750	1.377640724	-1.465969443
H	-4.665930748	2.271475077	0.006081948
H	-3.634833097	2.335428715	-1.439234018
C	-4.610679150	-0.761275470	0.646334708
H	-5.476868153	-0.978922427	0.014353285
H	-4.148067951	-1.697450399	0.966757059
H	-4.942526340	-0.217514500	1.535627604
C	2.838731289	0.196290582	1.852899551
H	2.657683372	-0.004164033	2.913147449
H	3.831923723	-0.179110050	1.588159800
H	2.800629616	1.276535749	1.693537951
C	1.822531700	-2.410178423	1.101418138
H	1.676915646	-2.682139397	2.151042461
H	1.103330374	-2.973660946	0.501903415
H	2.840201855	-2.665686607	0.791246057
C	-0.555670857	2.129576445	1.269351244
H	-1.304118276	2.840950727	0.889234126
H	0.198836491	1.381714702	3.156744719
H	0.411745906	2.431079388	0.833504140
C	-0.498843223	2.158192635	2.803146362
H	-1.487726450	1.872493625	3.198489428
C	-0.090582654	3.476427078	3.524711609
C	-1.061658621	4.610242367	3.158148289
H	-1.028619647	4.827010632	2.085890770
H	-2.093138695	4.338956356	3.415241003
H	-0.812615395	5.531490326	3.698071718
C	-0.150498316	3.227369785	5.045061111
H	0.131420821	4.123848915	5.610429287
H	-1.162428021	2.940150023	5.357010365
H	0.531181633	2.418796301	5.337961197
C	1.344255090	3.872979403	3.136051655
H	1.656079412	4.783714294	3.660929441
H	2.053692579	3.077789783	3.397539139
H	1.428040028	4.060072899	2.060596704
C	-1.956729412	-4.770047188	-0.256261885
C	-1.814517260	-3.609931231	0.490278035
C	-1.724155188	-6.016475201	0.336206704
H	-1.992429614	-2.651596546	0.022613809
H	-1.833096981	-6.931966305	-0.237870082
C	-1.430463791	-3.641080141	1.854724765
C	-1.343465924	-6.081572533	1.676867962
H	-1.154957652	-7.047009945	2.139950037
C	-1.191641212	-4.917062759	2.433726072
H	-2.242959261	-4.695295334	-1.301436663
C	-0.614100993	-3.486023903	4.760640621
C	-0.916017115	-2.337400436	3.979619503
C	-0.242877409	-3.355431557	6.101395607
H	-0.014790274	-4.246307373	6.681471825
C	-0.836875558	-1.075366497	4.621119976
C	-0.161027119	-2.098003626	6.699148655
H	-1.081062555	-0.198167562	4.038528919
H	0.131561473	-2.016768932	7.741810799
C	-0.461720496	-0.953329623	5.950500011
H	-0.407576233	0.031924825	6.404794693
S	-0.633535862	-5.132031918	4.102852345
C	-1.280475616	-2.410919666	2.587411642
O	-1.462661028	-1.272165060	1.931583166

==============================

T3

==============================

Ti	-0.406726032	-0.259422094	1.085536361
P	-1.073297739	-2.787253141	0.682694256
P	0.045728274	2.115340233	-0.063457616
N	-0.127589151	-0.655287802	-0.888394177
C	-2.459456682	-1.773450971	-3.583467484
C	-3.167352438	-2.793436527	-2.932590485
C	-1.436979175	-1.068580270	-2.953352451
H	-0.915535748	-0.272868961	-3.476187944
C	-2.799003839	-3.101757765	-1.616974592
C	-1.081399560	-1.372782707	-1.627704382
H	-3.324137211	-3.900462627	-1.097266555
C	-1.772534847	-2.416559935	-0.968955398
H	-2.723861217	-1.515329361	-4.606968880
C	2.883572102	-0.052228663	-2.996814251
C	1.764534354	-0.682909787	-2.465304852
C	3.216983795	1.266531587	-2.646276712
H	1.539548278	-1.714936972	-2.716293335
C	0.933482707	-0.010084756	-1.553301334
C	2.367589474	1.943728089	-1.765529752
H	2.605951548	2.970090866	-1.495479107
C	1.229898810	1.332398653	-1.226766109
H	3.527874470	-0.599032581	-3.681897879
C	-4.266991615	-3.549044132	-3.640758991
H	-4.947986126	-2.868980169	-4.164629459
H	-3.859898090	-4.238090992	-4.391738415
H	-4.861749172	-4.142027378	-2.939338684
C	4.472074986	1.912743330	-3.184253216
H	4.468958855	2.994857550	-3.022764921
H	5.366324902	1.509778857	-2.692043781
H	4.588014126	1.732829690	-4.258743286
C	-2.342972994	-3.781498909	1.573161840
H	-2.626323700	-4.675152302	1.008521795
H	-1.908468843	-4.087225437	2.528779030
H	-3.232662916	-3.177748680	1.766153693
C	0.161284968	-4.074209690	0.188777015
H	-0.290380508	-4.739620686	-0.552523732
H	1.037025809	-3.581972599	-0.238401905

H	0.474522322	-4.651114941	1.058255196
C	-1.014265895	3.130930662	-1.190395474
H	-1.709656000	3.735873699	-0.600544333
H	-0.404779434	3.787820816	-1.818679571
H	-1.593631864	2.457968235	-1.827806473
C	1.030533910	3.370214462	0.852038622
H	0.361049503	3.920298338	1.519609451
H	1.763548613	2.832146883	1.455921769
H	1.532778144	4.081560612	0.189516395
C	-3.093512297	0.689917088	0.487062961
H	-4.177960396	0.516668856	0.464880168
H	-2.936455488	1.771930099	0.456662178
H	-2.708014727	0.285938621	-0.456668973
C	-2.438980103	0.054379586	1.734451056
H	-2.856122017	-0.961215496	1.819924593
C	-2.781671286	0.770485103	3.071043015
C	-4.277727604	1.156376958	3.139436960
H	-4.538608074	1.925474763	2.405282736
H	-4.915121555	0.283681005	2.953973293
H	-4.528180599	1.550022006	4.132477760
C	-2.498819113	-0.204795957	4.230538368
H	-2.614876509	0.284106672	5.204821587
H	-3.195247650	-1.051631331	4.194238663
H	-1.485851884	-0.605518401	4.180613995
C	-1.943789840	2.052069902	3.244144678
H	-2.193675995	2.562232018	4.182577610
H	-0.874685049	1.826681495	3.254306078
H	-2.130226374	2.756052256	2.424099684
C	0.760364532	0.135963768	6.070841789
C	0.941542447	0.254865587	4.695193291
C	0.696432352	-1.126882076	6.663804531
H	1.000752211	1.229393244	4.224707127
H	0.551972806	-1.226511598	7.735827446
C	1.032318234	-0.880778491	3.874239206
C	0.856789112	-2.267270088	5.876564026
H	0.864611030	-3.253437519	6.332678318
C	1.028135419	-2.143201351	4.496011257
H	0.672111750	1.029989362	6.681978226
C	2.374758482	-2.960839510	2.263916016
C	2.224155426	-1.636580467	1.800926805
C	3.334637403	-3.808371544	1.707851768
H	3.445425987	-4.819188595	2.091299295
C	3.068567038	-1.194687128	0.769817591
C	4.137805462	-3.361579418	0.656309068
H	2.961574793	-0.174664199	0.418767065
H	4.872275352	-4.031004429	0.217350915
C	4.000763893	-2.052324533	0.187899053
H	4.622078419	-1.692517996	-0.627231538
S	1.259343982	-3.605459213	3.505341768
C	1.198767066	-0.751502216	2.405232906
O	1.105625868	0.523493886	1.913243175

==============================

T3-TS

==============================

Ti	-0.394330889	-0.144276932	0.900295556
P	-0.447716802	-2.717369556	0.176024705
P	0.110209443	2.306174278	-0.053177930
N	-0.323287636	-0.290928572	-1.184034586
C	-2.555596352	-1.775145650	-3.785124063
C	-2.887854815	-3.007680178	-3.198649406
C	-1.686854124	-0.879345596	-3.166912794
H	-1.458839059	0.077310875	-3.628268242
C	-2.288172483	-3.330260515	-1.975946784
C	-1.114573121	-1.192762852	-1.922701716
H	-2.511924505	-4.289144516	-1.512586355
C	-1.408915043	-2.446207762	-1.348659396
H	-3.004095793	-1.504998803	-4.738947392
C	2.654299021	0.332881659	-3.351369858
C	1.507789373	-0.275799155	-2.849831581
C	3.119691849	1.559867740	-2.853402853
H	1.181299210	-1.232487082	-3.246090889
C	0.768097043	0.327775210	-1.812222242
C	2.362784624	2.175076246	-1.848487139
H	2.691616058	3.137523890	-1.461580753
C	1.205592155	1.587020040	-1.331979036
H	3.212882757	-0.166107953	-4.140875816
C	-3.849165916	-3.954159975	-3.879494429
H	-4.784435272	-3.449548960	-4.148645878
H	-3.425050974	-4.359468937	-4.806777000
H	-4.102312565	-4.801186562	-3.234513760
C	4.403020859	2.172161818	-3.363230705
H	4.460438728	3.239398956	-3.126780510
H	5.282184124	1.691381216	-2.914072275
H	4.497463226	2.063254118	-4.449350834
C	-1.273460746	-4.111950397	1.053879738
H	-1.267627358	-5.026621819	0.452531815
H	-0.741449773	-4.292345524	1.991924405
H	-2.307399750	-3.843974829	1.285609007
C	1.070558071	-3.509963274	-0.515995324
H	0.791995525	-4.355376720	-1.152538657
H	1.605582118	-2.765012741	-1.108372211
H	1.726408601	-3.846425772	0.287111998
C	-0.870877743	3.521945953	-1.051351666
H	-1.496977687	4.132721424	-0.394687474
H	-0.201453567	4.172620296	-1.622665644
H	-1.515963435	2.977445841	-1.745804429
C	1.187458992	3.419173479	0.946162343
H	0.562667787	3.942809820	1.676372051
H	1.907926083	2.800878763	1.483475327
H	1.706411481	4.163219452	0.333655208
C	-1.823935866	-0.863378346	2.491863489
H	-1.577356219	-0.933952689	3.552004099
H	-2.439545393	-1.704915643	2.177241564
H	-0.285451472	-1.188289642	2.327785015
C	-2.171041250	0.447003633	1.961007237
H	-1.899846554	1.268142700	2.633550167
C	-3.551144600	0.674500942	1.311944604
C	-4.648340702	0.367209852	2.358660221
H	-4.609714508	-0.681160748	2.672724724
H	-4.518008232	0.987489283	3.252964497
H	-5.648765564	0.562604785	1.951580405

C	-3.684982777	2.142708063	0.878025234
H	-4.682590008	2.342615128	0.469175667
H	-3.525861740	2.821215630	1.725164175
H	-2.951185465	2.382457495	0.107508928
C	-3.762891769	-0.224576354	0.079807058
H	-4.781862259	-0.119791456	-0.311387748
H	-3.067722559	0.039262675	-0.722004056
H	-3.611776114	-1.283924699	0.312276393
C	0.403558284	0.672228575	5.967728615
C	0.657676101	0.626917899	4.597220898
C	0.454202592	-0.497453153	6.728642941
H	0.612478912	1.522394300	3.986696482
H	0.258090377	-0.467517763	7.796735287
C	0.955429196	-0.585654438	3.965545654
C	0.773450375	-1.710704088	6.119109631
H	0.838651419	-2.621840954	6.707292557
C	1.014992476	-1.754391909	4.743367195
H	0.163730636	1.619867682	6.441953182
C	2.459900379	-2.788161278	2.668205023
C	2.245907307	-1.551022768	2.033322334
C	3.508393288	-3.622730494	2.268940687
H	3.671659708	-4.564985752	2.784636497
C	3.106151581	-1.173494697	0.990924120
C	4.339251518	-3.241675615	1.214132547
H	2.933717966	-0.220989123	0.500135422
H	5.146541595	-3.898108244	0.902091742
C	4.136416435	-2.014867306	0.575498044
H	4.780938625	-1.711423874	-0.244410351
S	1.353527427	-3.325872898	3.963880777
C	1.132274866	-0.658245504	2.476035357
O	1.085369587	0.561191380	1.839979410

==============================

T4

==============================

Ti	-0.658434451	-0.331197917	0.856168032
P	-0.329761863	-2.829532623	0.272021085
P	0.236185208	2.116187334	0.222311214
N	-0.346541047	-0.382268667	-1.177105427
C	-2.584114313	-2.039702892	-3.664643288
C	-2.817273855	-3.281052113	-3.048555136
C	-1.748986602	-1.086302757	-3.094042063
H	-1.607043505	-0.119588807	-3.567910671
C	-2.156388760	-3.547629833	-1.846980095
C	-1.099091530	-1.348527908	-1.875169396
H	-2.316088200	-4.507451534	-1.361357331
C	-1.291231275	-2.612042904	-1.269158125
H	-3.089740276	-1.810301900	-4.600337029
C	2.449661732	0.483810604	-3.489995480
C	1.358399868	-0.190496579	-2.954324007
C	2.928798676	1.681017280	-2.933508873
H	1.024346471	-1.120920539	-3.402216911
C	0.687313795	0.305897951	-1.814789176
C	2.249700069	2.182642937	-1.818640947
H	2.595954180	3.113790035	-1.373457670
C	1.153475046	1.522504687	-1.253061533
H	2.950617552	0.064963445	-4.360696316
C	-3.740047693	-4.295041561	-3.682999134
H	-4.688609123	-3.837161779	-3.985532284
H	-3.292782784	-4.733285427	-4.583785534
H	-3.968994379	-5.115268707	-2.996004581
C	4.142039299	2.375611782	-3.505629301
H	4.222176075	3.405596018	-3.144480228
H	5.069920540	1.858690262	-3.227857590
H	4.108276367	2.406786203	-4.600511551
C	-1.074526668	-4.284391880	1.123618960
H	-1.080466390	-5.175796032	0.488421351
H	-0.485687941	-4.501668453	2.019708872
H	-2.095588207	-4.045185089	1.429035544
C	1.277936935	-3.509836435	-0.348932266
H	1.112493753	-4.418575287	-0.936551929
H	1.759344101	-2.751969337	-0.970913887
H	1.942299724	-3.728410959	0.491865367
C	-0.472547650	3.713868856	-0.399172604
H	-0.912262976	4.277917385	0.428445995
H	0.309813797	4.317110538	-0.870504737
H	-1.253272891	3.506513119	-1.135383487
C	1.544937849	2.726571083	1.375814557
H	1.055771589	3.227166176	2.217175007
H	2.092216492	1.866332173	1.763902426
H	2.234110117	3.428355932	0.896989405
C	-2.402075768	-1.145552039	1.788257122
H	-2.145660400	-1.653017163	2.720119238
H	-3.045732260	-1.747790933	1.144005060
H	1.011436462	-2.197246313	2.692301512
C	-2.534545898	0.285775661	1.760195374
H	-2.309937477	0.796670139	2.701730251
C	-3.690441847	0.957112253	0.995297372
C	-5.033709526	0.470015556	1.587606788
H	-5.145911217	-0.612212837	1.465939879
H	-5.087885380	0.691118360	2.659741163
H	-5.884624481	0.958449721	1.095436335
C	-3.606108427	2.481906652	1.170390248
H	-4.421740532	2.988344908	0.640216649
H	-3.671936035	2.757096052	2.230021477
H	-2.660068035	2.863476992	0.783509493
C	-3.655590534	0.618604779	-0.508767962
H	-4.534192562	1.026819944	-1.022894621
H	-2.760151148	1.031935930	-0.985624075
H	-3.647761583	-0.462656647	-0.681042433
C	1.398046970	0.303663909	6.222741604
C	1.185660362	0.135362685	4.852855206
C	2.220218420	-0.583745897	6.920763493
H	0.539422214	0.803760529	4.293345928
H	2.387471676	-0.454528749	7.986254692
C	1.785533190	-0.923910260	4.173899174
C	2.847226143	-1.632117987	6.246055126
H	3.511373758	-2.309958935	6.774758339
C	2.627784252	-1.800484538	4.876114845
H	0.917800784	1.125473022	6.746441841
C	3.744595051	-2.347085953	2.451433897
C	2.823849440	-1.413907051	1.949606061

C	4.902995586	-2.660137892	1.733319163
H	5.611176014	-3.378497839	2.136615276
C	3.101812840	-0.772819877	0.743371665
C	5.147888184	-2.035967350	0.508973837
H	2.395941019	-0.053751145	0.351738781
H	6.044224739	-2.287456512	-0.051397581
C	4.253627777	-1.082873344	0.019072400
H	4.434475899	-0.586494327	-0.930136144
S	3.409130812	-3.159365892	4.009488583
C	1.516939402	-1.210408330	2.706290722
O	0.697624028	-0.251514196	2.113020420

==============================

T5

==============================

Ti	-0.508679748	-0.169851288	1.079155564
P	-0.872409403	-2.635825872	0.269119531
P	0.641821742	2.150186539	0.491789222
N	-0.314154238	-0.219448969	-1.164274693
C	-2.712440729	-1.462768674	-3.762889862
C	-3.205020905	-2.635555267	-3.168478966
C	-1.736277699	-0.680752397	-3.156845331
H	-1.437451363	0.252618134	-3.622574329
C	-2.643727779	-3.007645845	-1.944521427
C	-1.177015066	-1.033749580	-1.904474497
H	-3.007917166	-3.907011986	-1.453179598
C	-1.654651642	-2.240646839	-1.324477553
H	-3.138620853	-1.128363729	-4.706874371
C	2.153157234	0.965576947	-3.701300859
C	1.162684917	0.216101304	-3.088696480
C	2.685980320	2.122061968	-3.105593443
H	0.813796580	-0.690730691	-3.570817709
C	0.617028356	0.572417796	-1.824438930
C	2.172927380	2.472925186	-1.859724164
H	2.587410688	3.347838163	-1.361012936
C	1.173496366	1.732520700	-1.209861279
H	2.543534994	0.634128392	-4.662493706
C	-4.304696083	-3.437039614	-3.821643353
H	-5.137236118	-2.789448738	-4.124161243
H	-3.954188585	-3.952686310	-4.725121021
H	-4.704129219	-4.195403576	-3.140899420
C	3.801834106	2.897116661	-3.764295101
H	3.965035200	3.862009525	-3.273820877
H	4.750932217	2.346113920	-3.725469112
H	3.589449167	3.091801167	-4.822233200
C	-1.892284870	-3.977869034	1.027091861
H	-1.923794150	-4.872496605	0.396602750
H	-1.443822503	-4.243113041	1.988968015
H	-2.910706282	-3.629917622	1.204065084
C	0.588767648	-3.618119955	-0.318506747
H	0.238964573	-4.429855347	-0.963675082
H	1.248325467	-2.960982323	-0.886308610
H	1.152122021	-4.039189339	0.518262267
C	0.165239796	3.949603558	0.369452149
H	0.235949621	4.407079220	1.359635472
H	0.847916067	4.475377083	-0.305073261
H	-0.856540263	4.076699734	0.008170845
C	2.237134457	2.343175173	1.408510447
H	2.016683578	2.641654730	2.437648535
H	2.748061180	1.380903721	1.423175097
H	2.876588821	3.099943876	0.945204496
C	-1.371142149	-0.831279397	2.956378937
H	-0.761630177	-1.621383905	3.397150278
H	-2.429362774	-1.089426279	2.909707308
H	1.532435894	-2.529388189	2.043583870
C	-1.002490401	0.539299548	3.139800072
H	-0.026841136	0.665292442	3.607076645
C	-1.962658882	1.622170329	3.662605524
C	-2.420131207	1.202872276	5.080508709
H	-2.982707500	0.264305919	5.041145802
H	-1.555143952	1.043006778	5.734393120
H	-3.058707952	1.969160199	5.539327145
C	-1.205066562	2.957128286	3.773130417
H	-1.838551283	3.742569923	4.203256130
H	-0.317389995	2.855676889	4.410035133
H	-0.877007544	3.291628599	2.787042141
C	-3.208688021	1.836435318	2.787232876
H	-3.896294355	2.546628714	3.263535976
H	-2.930907249	2.230170012	1.805457592
H	-3.750421047	0.899658620	2.626008034
C	3.130508661	-0.110693187	5.299905300
C	2.544253588	-0.228174791	4.038106918
C	3.967797279	-1.122201681	5.774928570
H	1.885009050	0.537764490	3.651493549
H	4.426239491	-1.038350940	6.756447792
C	2.776716471	-1.354661703	3.252233744
C	4.242829323	-2.236534834	4.980449677
H	4.925316334	-3.006539822	5.328938007
C	3.651653767	-2.349323750	3.719519377
H	2.930418015	0.765190125	5.910536289
C	4.062757492	-2.920141935	1.098519921
C	3.157627821	-1.876740456	0.855621159
C	4.988062382	-3.295531511	0.120420188
H	5.688975334	-4.101665974	0.318723798
C	3.223934889	-1.179647446	-0.349131644
C	5.014707088	-2.618102789	-1.100333810
H	2.526213646	-0.372940123	-0.522210002
H	5.731448650	-2.916377544	-1.860939503
C	4.143832207	-1.550352216	-1.331865907
H	4.163344383	-1.010046482	-2.273701191
S	4.009838104	-3.762387514	2.677120209
C	2.099721670	-1.589095592	1.910438538
O	1.225108624	-0.581647217	1.532863975
C	-2.184928179	3.180527449	-2.416279078
C	-2.367079020	2.240890980	-1.419096112
C	-3.099004507	3.237986565	-3.477987766
H	-1.330659747	3.848717451	-2.384380817
H	-1.675286651	2.145722628	-0.597053230
C	-3.432356119	1.323824644	-1.454467416
H	-2.966486931	3.966095448	-4.273244381
C	-4.175119400	2.362055540	-3.528910398
C	-4.349283218	1.394649863	-2.526043177

C	-3.458716154	0.289490879	-0.418852478
H	-4.874378681	2.408776999	-4.359203815
S	-5.686343670	0.282138169	-2.743894100
C	-4.558449268	-0.677365303	-0.366298288
O	-2.543732166	0.231274381	0.435182899
C	-5.553772926	-0.785976470	-1.360306382
C	-4.598212242	-1.565533757	0.728433311
C	-6.529433250	-1.794938326	-1.265669107
H	-7.283344746	-1.887238979	-2.042503834
C	-6.532865524	-2.665709972	-0.188303664
H	-3.828746319	-1.456658959	1.482887864
C	-5.569112778	-2.544284821	0.825858057
H	-7.293321609	-3.439189911	-0.129579663
H	-5.582994461	-3.216462612	1.678297520

================================

^{3}T6

================================

Ti	1.127757430	-1.486498713	-1.452925444
P	-1.364108682	-1.342489958	-2.195385456
P	3.610391617	-0.886393845	-1.268426895
N	1.286191821	-0.185135677	-3.027733564
C	-0.180043817	-0.077396519	-6.469562054
C	-1.498084903	-0.511086822	-6.252855778
C	0.731940866	0.031988747	-5.428523064
H	1.747058272	0.352523834	-5.638463497
C	-1.842388868	-0.862825572	-4.947921753
C	0.374398023	-0.273228616	-4.094628811
H	-2.849659443	-1.228643775	-4.756229877
C	-0.941384852	-0.765025795	-3.880002975
H	0.140497029	0.168070525	-7.480264664
C	3.045166731	3.075488091	-3.547693968
C	2.090139627	2.074051142	-3.643296480
C	4.227446079	2.908175230	-2.808630943
H	1.179579377	2.258752584	-4.202580929
C	2.261648178	0.820343435	-3.005248070
C	4.387547016	1.692595601	-2.145367622
H	5.281532764	1.542697549	-1.542251110
C	3.438149929	0.664709568	-2.218158484
H	2.862401962	4.024257183	-4.049241543
C	-2.493525505	-0.587586701	-7.385747910
H	-2.044946909	-1.023107648	-8.285926819
H	-3.361257076	-1.198016882	-7.116286755
H	-2.865902662	0.407572955	-7.661802769
C	5.266130924	4.001970768	-2.740969181
H	6.008693695	3.803257942	-1.961829066
H	5.805470467	4.104741573	-3.691543818
H	4.809480667	4.975092411	-2.525586367
C	-2.765557289	-0.266874343	-1.655999899
H	-3.616426945	-0.393450439	-2.333309650
H	-3.064592123	-0.533997059	-0.638289034
H	-2.450457096	0.776532769	-1.659615755
C	-2.219439745	-2.952253342	-2.473414898
H	-3.066020250	-2.851258993	-3.158977032
H	-1.504716873	-3.672478437	-2.872639179
H	-2.582127571	-3.331103802	-1.514830709
C	4.232278347	-0.392309517	0.393984556
H	4.429065228	-1.302958965	0.968548656
H	5.143180370	0.212025329	0.350858152
H	3.435489655	0.167671859	0.888472259
C	5.062015533	-1.732622743	-2.034460783
H	5.332953930	-2.602868080	-1.431941628
H	4.788523197	-2.066637516	-3.038781881
H	5.916234493	-1.051573634	-2.102813959
H	2.201304674	-5.008350849	-2.530711174
C	-0.855932772	3.112667561	-1.240122318
C	-0.193819880	2.002458811	-0.734065711
C	-2.015904665	3.587756395	-0.615571856
H	0.705755293	1.642041445	-1.211847186
H	-2.539845467	4.455718994	-1.004785180
C	-0.671495020	1.310927510	0.405207396
C	-2.489728689	2.946026802	0.532432437
H	-3.379686594	3.316993713	1.034256816
C	-1.835424542	1.822071314	1.040376902
H	-0.459910631	3.610105276	-2.121224403
C	-1.232028008	0.024850663	3.056518316
C	-0.135407522	-0.345458031	2.234009743
C	-1.321236134	-0.433315009	4.371677876
H	-2.163461208	-0.128213868	4.987296104
C	0.847664237	-1.196798444	2.795634031
C	-0.340481728	-1.277234674	4.898000717
H	1.690122128	-1.470740080	2.171547890
H	-0.422283828	-1.625017047	5.923542023
C	0.746609151	-1.656289458	4.101465225
H	1.522929788	-2.296292543	4.512697220
S	-2.584927320	0.983280241	2.416116238
C	0.020761469	0.141419008	0.888195872
O	0.953356624	-0.422719061	0.125461504
C	4.649235725	-4.625514030	0.633030474
C	3.855640411	-4.767435551	-0.505875587
C	4.056226730	-4.278153419	1.849502802
H	5.722064972	-4.785211563	0.572948456
H	4.311496258	-5.021687984	-1.459857821
C	2.476475477	-4.552789211	-0.454447597
H	4.663638115	-4.171045780	2.743956327
C	2.675808191	-4.098268986	1.929627895
C	1.886260629	-4.234672070	0.782009423
C	1.630853772	-4.546646118	-1.713271618
H	2.205632210	-3.868869066	2.880064964
S	0.124679156	-3.980102777	0.912693262
C	0.339244425	-5.311830521	-1.518373966
C	-0.432973266	-5.088355541	-0.366150439
C	-1.672689676	-5.718446732	-0.212355629
C	-0.144261912	-6.185628414	-2.495838165
H	-2.253957272	-5.553120613	0.690667450
C	-2.150066137	-6.567365170	-1.210156918
H	0.454006821	-6.358384609	-3.387413025
C	-1.385013819	-6.807790756	-2.354048252
H	-3.113719940	-7.052807808	-1.084210873
H	-1.751177907	-7.476247787	-3.127475023
O	1.335210323	-3.213375092	-2.071882486

===============================

2

===============================

Ti	0.650205255	-1.330776572	-1.909713864
P	-1.151596785	-2.962914467	-2.844834328
P	2.482269764	0.439111590	-2.490858316
N	-0.059552334	-0.397243530	-3.573934555
C	-0.376668215	-1.475588083	-7.133120060
C	-0.832929552	-2.794970751	-6.994577408
C	-0.156293467	-0.650145233	-6.034786701
H	0.225465730	0.355436683	-6.179476261
C	-1.078283310	-3.254245996	-5.696506023
C	-0.381754369	-1.123192430	-4.726487637
H	-1.433152318	-4.273891449	-5.559891224
C	-0.861303627	-2.449611425	-4.576752186
H	-0.168116704	-1.089369893	-8.129179955
C	-0.846197724	3.216228247	-4.069906235
C	-0.965132177	1.830855608	-4.124826431
C	0.283464223	3.834997177	-3.511060953
H	-1.856262565	1.367534637	-4.537312984
C	0.057330925	1.007071853	-3.626398563
C	1.308699131	3.012478352	-3.032302618
H	2.196271896	3.475844860	-2.606756210
C	1.217057943	1.618607044	-3.091998100
H	-1.655809522	3.833691120	-4.452205181
C	-1.057805061	-3.675133705	-8.201096535
H	-0.272113651	-3.532999992	-8.951439857
H	-1.067769408	-4.734481812	-7.926602364
H	-2.014738798	-3.453401089	-8.690839767
C	0.375466585	5.338505268	-3.395682812
H	1.414663553	5.681339264	-3.433307886
H	-0.173868850	5.837631226	-4.200260162
H	-0.048077442	5.689748764	-2.445712566
C	-2.995150566	-2.941372633	-2.766234398
H	-3.405350447	-3.413710833	-3.663664579
H	-3.330772877	-3.473347187	-1.876219749
H	-3.336896420	-1.905484200	-2.705967426
C	-0.715246260	-4.748425961	-2.759397507
H	-1.250188947	-5.352967739	-3.497691870
H	0.362716287	-4.839600086	-2.906476498
H	-0.962547719	-5.108037949	-1.756484866
C	3.362114906	1.301787019	-1.127735853
H	4.187133789	0.668259680	-0.788489640
H	3.766599178	2.273568630	-1.426590204
H	2.653508186	1.422338963	-0.305210680
C	3.720569611	0.337352842	-3.856480122
H	4.537967682	-0.325014204	-3.555398941
H	3.241061449	-0.102277555	-4.734175682
H	4.118707180	1.325515747	-4.107206821
H	3.728837490	-2.268948078	-1.736003995
C	1.722384453	-3.331575155	2.390214920
C	1.385739565	-2.311303854	1.503063917
C	0.761170208	-4.263456821	2.785925627
H	2.128945827	-1.590341568	1.180090547
H	1.014649510	-5.060655594	3.478759289
C	0.089638092	-2.201809168	0.971079946
C	-0.543316901	-4.150282383	2.301663160
H	-1.309073925	-4.850740433	2.624919653
C	-0.875552833	-3.134446621	1.399951100
H	2.740159035	-3.406106710	2.762173414
C	-2.727242947	-1.400581956	0.255305052
C	-1.611647844	-0.609552562	-0.090114996
C	-4.023820400	-0.897352338	0.126887470
H	-4.870030403	-1.516861081	0.412593663
C	-1.845294595	0.695596576	-0.560597718
C	-4.234481812	0.388220191	-0.376977712
H	-0.987835586	1.304987550	-0.825681567
H	-5.248038292	0.762922347	-0.488395512
C	-3.140085459	1.184744954	-0.722279608
H	-3.290162802	2.186980247	-1.113429427
S	-2.534574270	-3.106143236	0.750949562
C	-0.237213835	-1.150126100	-0.020367989
O	0.778498530	-0.290009201	-0.339008838
C	2.919594049	-6.036303043	0.275727332
C	2.534257650	-4.953299522	-0.515810311
C	4.233691216	-6.504463196	0.230018944
H	2.195155382	-6.497584820	0.940322042
H	1.527248144	-4.556100845	-0.464365482
C	3.461966038	-4.328486919	-1.347979069
H	4.536599636	-7.347074032	0.845724642
C	5.165279388	-5.901168346	-0.616501987
C	4.778532982	-4.812443256	-1.403184891
C	3.119827271	-3.091970682	-2.160823584
H	6.182518959	-6.277985573	-0.673876405
S	5.971487999	-4.012554646	-2.473739624
C	3.570431232	-3.249096870	-3.608314276
C	4.901344299	-3.621382475	-3.852678537
C	5.392063618	-3.678713083	-5.160908222
C	2.729517698	-2.986343384	-4.689351082
H	6.421442986	-3.977086067	-5.338817596
C	4.549757957	-3.370432377	-6.230329990
H	1.697338223	-2.725451231	-4.503098011
C	3.213440418	-3.037842035	-5.997764587
H	4.936198235	-3.413153410	-7.244967937
H	2.542794943	-2.815529585	-6.822476864
O	1.770484805	-2.762180567	-2.063927889

===============================

^{3}T2-TS

===============================

Ti	-0.420195788	-0.442756951	0.776226938
P	-1.795486808	-2.614215136	0.366018146
P	0.592399776	1.774946928	0.058352206
N	-0.265339822	-0.638179481	-1.368310213
C	-2.674429417	-1.677765369	-4.042016029
C	-3.409137249	-2.703449965	-3.424762726
C	-1.644309402	-1.016232491	-3.392408371
H	-1.138050556	-0.208414227	-3.907655001
C	-3.073930025	-2.996994019	-2.106126308
C	-1.256894469	-1.338717103	-2.062334299
H	-3.651384830	-3.756765842	-1.582234144
C	-2.046336889	-2.335781574	-1.417508960

H	-2.930171728	-1.377659917	-5.057082176
C	2.272687674	0.320815951	-3.958493471
C	1.276899695	-0.389186203	-3.300532818
C	2.764251232	1.540260196	-3.467003584
H	0.949709713	-1.342078209	-3.703120708
C	0.697714925	0.082629867	-2.099514723
C	2.222494602	2.001032591	-2.266820431
H	2.604412794	2.927685261	-1.843815207
C	1.217248321	1.302389741	-1.586356997
H	2.695522547	-0.092664108	-4.872244835
C	-4.517049789	-3.423104048	-4.155144691
H	-5.184655666	-2.717247248	-4.663672447
H	-4.123529911	-4.101398945	-4.923421383
H	-5.126844406	-4.020979881	-3.470345020
C	3.827515841	2.312108755	-4.209943771
H	4.294218063	3.068670750	-3.571375608
H	4.619337559	1.650414705	-4.578991413
H	3.410092831	2.830920696	-5.082370758
C	-3.503952742	-2.617742062	1.072484374
H	-4.125009060	-3.411358595	0.646917999
H	-3.444012165	-2.751355886	2.156593084
H	-3.970010996	-1.651884794	0.869038701
C	-1.342730522	-4.407466888	0.489348173
H	-2.120991707	-5.027450085	0.032944098
H	-0.400950879	-4.587952614	-0.034661014
H	-1.224715471	-4.695184708	1.538688660
C	-0.726476729	3.033252001	-0.252303839
H	-1.147133231	3.357345343	0.704225481
H	-0.327849180	3.896947384	-0.793566704
H	-1.524632692	2.569971561	-0.835445106
C	1.920370698	2.785845041	0.843750060
H	1.579486251	3.071554899	1.842870235
H	2.827313423	2.186074734	0.944101751
H	2.144588232	3.692826986	0.273489326
C	1.189139605	-1.656046510	2.431021452
H	0.388125032	-2.078847885	3.028383970
H	1.886848211	-1.031695485	2.978296041
H	0.246782005	-0.004190505	2.333858252
C	1.516581297	-2.191468239	1.209618807
H	0.914705575	-3.023975372	0.859973133
C	2.890488625	-2.095994234	0.542616963
C	3.444152117	-0.661656380	0.512867808
H	2.848850250	-0.031845570	-0.150216132
H	3.451226234	-0.209305868	1.510725021
H	4.474324226	-0.656244874	0.138363928
C	3.864366770	-2.992629290	1.347190142
H	4.851023674	-3.023941278	0.868077457
H	3.990418196	-2.614652157	2.367552042
H	3.487714529	-4.019612789	1.413207650
C	2.789250135	-2.641858816	-0.893363833
H	3.758843422	-2.592254877	-1.401855707
H	2.468866110	-3.691294193	-0.887024343
H	2.065433979	-2.071162462	-1.476100445
C	-2.926575422	0.776807070	5.123201847
C	-2.637155056	0.644699574	3.773187399
C	-4.165071487	1.288874388	5.529003143
H	-1.674884677	0.261169761	3.451013327
H	-4.401709080	1.392865539	6.583823204
C	-3.573122978	1.019104481	2.775963545
C	-5.102691174	1.670198321	4.571153641
H	-6.064229965	2.069814205	4.883217812
C	-4.820262909	1.545030475	3.207590580
H	-2.187208891	0.483143598	5.862879753
C	-5.438846588	1.697985172	0.490989923
C	-4.143097878	1.154242873	0.293590933
C	-6.271306992	1.956268072	-0.602711022
H	-7.261243820	2.372625113	-0.434020281
C	-3.740879536	0.884633839	-1.038642526
C	-5.846252918	1.680681348	-1.901732445
H	-2.760594606	0.460081846	-1.200865984
H	-6.509242058	1.884810448	-2.737474918
C	-4.573159695	1.138727307	-2.117400646
H	-4.230401516	0.900269389	-3.119502544
S	-6.084294319	2.093003750	2.092420816
C	-3.244184017	0.851381600	1.381858945
O	-2.061467886	0.358939886	1.072423935

================================

^{3}T8

================================

Ti	0.411127478	-0.379937798	-1.729515076
P	-1.693013906	-0.621483624	-3.146868706
P	2.846104860	0.246393576	-1.233518004
N	1.135940552	-0.001551640	-3.620987892
C	0.682457805	-1.649961114	-6.952451706
C	-0.679728568	-1.986425042	-6.937333107
C	1.288558006	-0.998358548	-5.885337353
H	2.348612070	-0.770109296	-5.924887180
C	-1.405763149	-1.656763673	-5.791462898
C	0.552253664	-0.632389307	-4.740372181
H	-2.458243132	-1.927627921	-5.745615959
C	-0.820076168	-0.993534684	-4.707522392
H	1.286087513	-1.917374492	-7.817557812
C	3.218078852	2.749440432	-5.077252865
C	2.243578672	1.765020728	-4.963917732
C	4.160733700	2.982577801	-4.064261436
H	1.520166397	1.635058641	-5.761762142
C	2.159818649	0.945315063	-3.816500187
C	4.069780350	2.188146830	-2.919895411
H	4.771691799	2.362751961	-2.106646299
C	3.096464872	1.193636298	-2.778067350
H	3.237910271	3.367121935	-5.973326206
C	-1.327376366	-2.670030355	-8.117747307
H	-0.716977775	-3.503894091	-8.482373238
H	-2.314539671	-3.066080093	-7.860346794
H	-1.459653616	-1.977029920	-8.958486557
C	5.220334053	4.047785759	-4.212567329
H	5.679833412	4.292476177	-3.249900341
H	6.023248672	3.726571798	-4.888461590
H	4.802458763	4.972219467	-4.627145290
C	-2.581397057	0.971992075	-3.460515261
H	-3.231806755	0.901558101	-4.337867737

H	-3.177613497	1.233335376	-2.580941677
H	-1.841007113	1.760891318	-3.616356611
C	-3.062902212	-1.848234296	-3.027433634
H	-3.760100603	-1.777766228	-3.868245363
H	-2.636878490	-2.853359699	-2.986696720
H	-3.611212492	-1.665537834	-2.098948717
C	3.130021572	1.479680300	0.110266708
H	2.962238312	0.997479320	1.077310681
H	4.142700195	1.892935753	0.087971255
H	2.406661749	2.290309668	-0.002938418
C	4.315848351	-0.864595473	-1.106056333
H	4.288908958	-1.393568873	-0.148956567
H	4.273457527	-1.601506114	-1.911772490
H	5.246698856	-0.293704271	-1.183820844
H	0.484400272	-2.032672882	-1.207630396
C	0.533395410	4.419485092	-2.611165285
C	0.257869631	3.249150753	-1.920643806
C	0.317331493	5.658415318	-1.998093486
H	0.428434789	2.301237583	-2.411683798
H	0.531411946	6.582723618	-2.526390791
C	-0.229334623	3.257586718	-0.588854969
C	-0.174845502	5.704587460	-0.694363117
H	-0.345070541	6.664724827	-0.213831484
C	-0.448091865	4.528623104	0.010480394
H	0.928826094	4.357697487	-3.620614290
C	-1.175873160	3.075636625	2.274602652
C	-0.878261685	1.932504296	1.487203360
C	-1.573287249	2.937116146	3.607831955
H	-1.793589592	3.824754715	4.195309162
C	-0.997768223	0.662654102	2.104793549
C	-1.687257051	1.674714923	4.186267853
H	-0.767680228	-0.214008734	1.509917974
H	-1.997864723	1.582969189	5.222817421
C	-1.395951748	0.535463929	3.426320791
H	-1.478856206	-0.452314109	3.870460033
S	-1.095009685	4.729622364	1.646366000
C	-0.456314981	2.016946316	0.111196674
O	-0.228527516	0.878984451	-0.526910245

==============================

W2-TS

==============================

Ti	-1.148366570	0.242497027	1.009678364
P	-3.415380955	0.704444706	-0.153816879
P	0.465657800	1.355530977	-1.798789144
N	-1.180052638	-0.881840348	-0.765599251
C	-3.231118441	-2.288279295	-3.579552889
C	-4.480715752	-1.689405918	-3.364989758
C	-2.130485535	-2.038770437	-2.766310215
H	-1.190409184	-2.524312735	-2.995616198
C	-4.570276260	-0.787850976	-2.301575899
C	-2.228486538	-1.166386724	-1.656119466
H	-5.512355804	-0.271765739	-2.121374607
C	-3.480582237	-0.527767181	-1.468768120
H	-3.111726522	-2.974956989	-4.415881634
C	1.700900435	-3.130076408	-1.583723903
C	0.454466224	-2.693967342	-1.140468478
C	2.643263578	-2.220787048	-2.075540543
H	-0.270528823	-3.402992725	-0.756151795
C	0.103309222	-1.339858532	-1.195712447
C	2.283858538	-0.866722703	-2.139284611
H	3.014589787	-0.162966669	-2.528847694
C	1.026457071	-0.410106093	-1.726110935
H	1.949931383	-4.187255859	-1.526757598
C	-5.669813633	-2.020426750	-4.234728336
H	-5.376121521	-2.152694702	-5.282030106
H	-6.154302120	-2.953153372	-3.916568756
H	-6.427582264	-1.231042385	-4.196733952
C	4.026175976	-2.673889875	-2.483217716
H	4.455227375	-2.021633148	-3.250760317
H	4.712883472	-2.659139156	-1.626592994
H	4.015899658	-3.696680307	-2.873609543
C	-3.465456963	2.320614338	-1.048727036
H	-4.330879688	2.368761301	-1.716548324
H	-3.508216143	3.138112068	-0.324410588
H	-2.547618151	2.419437647	-1.632383227
C	-5.054293156	0.674576759	0.689680278
H	-5.856976032	0.951766491	-0.000508267
H	-5.245441914	-0.327876121	1.076526403
H	-5.044698715	1.380411506	1.524538040
C	-0.361689538	1.342430353	-3.472367287
H	-0.677034497	2.359583855	-3.728932619
H	0.305798769	0.965879500	-4.254790306
H	-1.248057723	0.704076886	-3.429666519
C	2.042297840	2.247657061	-2.225378752
H	1.810564637	3.309919596	-2.351244688
H	2.749071836	2.151003361	-1.395716429
H	2.515669823	1.886576653	-3.145270824
C	-1.812590241	2.125801086	1.828163743
H	-2.881871700	2.333986521	1.940153122
H	-1.369287252	2.213982105	2.823327780
H	-1.380760312	2.912630081	1.191577315
C	-1.492349386	-0.912312984	2.553843260
H	-1.718829036	-0.300750822	3.436095476
C	-1.946324706	-2.374779463	2.699299097
C	-0.879347503	-3.407424450	2.284755230
H	-0.364085883	-3.100277662	1.374218464
H	-0.125257522	-3.545616150	3.059148073
H	-1.353741288	-4.379752636	2.106274128
C	-2.429311752	-2.676063299	4.133091927
H	-2.795955658	-3.707484245	4.205921650
H	-1.621048808	-2.548516273	4.855866909
H	-3.251120567	-2.007335186	4.416235924
C	-3.161209583	-2.542986631	1.745881319
H	-3.585436106	-3.550107479	1.848424673
H	-3.946717024	-1.821276546	1.990554214
H	-2.866858721	-2.393498182	0.704773188
C	0.976937950	3.002162218	4.610984325
C	0.925574660	2.149520397	3.513565540
C	0.916727662	2.478560448	5.905941010
H	0.959218264	2.539006948	2.502995491
H	0.948564768	3.138796091	6.768067837

C	0.798258841	0.760962605	3.679357290
C	0.861176252	1.100366354	6.093926430
H	0.879396319	0.681763470	7.096149921
C	0.824920177	0.243916571	4.987386703
H	1.060522199	4.074126720	4.457347393
C	1.727724552	-2.026744604	3.808389664
C	1.566246033	-1.350878477	2.582602739
C	2.544698954	-3.157570601	3.895581245
H	2.663083792	-3.662451029	4.850059509
C	2.289790630	-1.801479340	1.472221255
C	3.215594053	-3.620426893	2.764990091
H	2.200164318	-1.253638864	0.544985056
H	3.848410606	-4.500568867	2.838112831
C	3.096830368	-2.931902409	1.555949688
H	3.629141569	-3.273261070	0.673459351
S	0.860183597	-1.505139351	5.267420292
C	0.725900471	-0.128526360	2.479534626
O	0.722393095	0.510247767	1.293715954

================================
W5
================================

Ti	-0.359083295	-0.146054134	0.972056329
P	-2.746674538	-0.127016619	-0.015473665
P	2.171814919	-0.041021626	0.542204499
N	-0.123668596	-0.850812495	-0.992420673
C	-1.777962446	-3.744982004	-2.686462641
C	-3.117392778	-3.583394289	-2.296776772
C	-0.784896672	-2.861722231	-2.282323360
H	0.243448913	-3.018436909	-2.592386961
C	-3.424490690	-2.490435600	-1.483994842
C	-1.090518594	-1.753174186	-1.468313694
H	-4.456152916	-2.345314264	-1.171406746
C	-2.442295551	-1.581233859	-1.076874256
H	-1.507679701	-4.591949940	-3.313782930
C	1.820955276	0.137102976	-4.032377720
C	0.779073656	-0.345701188	-3.250352859
C	3.030040741	0.583110452	-3.475288868
H	-0.143768117	-0.666385710	-3.722570181
C	0.895621002	-0.411902279	-1.844238043
C	3.146882534	0.530517578	-2.084542990
H	4.069512844	0.876401603	-1.622574210
C	2.113888025	0.046859551	-1.275759459
H	1.688342214	0.181270748	-5.111852169
C	-4.174808502	-4.573966503	-2.722534657
H	-4.076226711	-4.834670544	-3.782151699
H	-4.098600388	-5.508642197	-2.152575016
H	-5.182766914	-4.176931381	-2.567716837
C	4.150536060	1.090391994	-4.351578712
H	4.952548504	1.539111495	-3.757382631
H	4.593820572	0.281804234	-4.946455002
H	3.795265913	1.849691510	-5.058206081
C	-3.261188984	1.203736305	-1.188149095
H	-4.131572723	0.895001292	-1.775206208
H	-3.497018337	2.116859674	-0.633764982
H	-2.425494432	1.408572197	-1.862183690
C	-4.272581577	-0.499239653	0.940496266
H	-5.129950047	-0.696213186	0.289621234
H	-4.083185196	-1.368033290	1.575317621
H	-4.503961563	0.355984509	1.581463695
C	3.212652445	1.370076656	1.098398089
H	3.300596476	1.329495549	2.187605381
H	4.213983059	1.345363617	0.658668518
H	2.714713812	2.303883076	0.828202546
C	3.193209410	-1.528736591	0.921866477
H	3.311112881	-1.625596523	2.004775763
H	2.661490917	-2.408503294	0.551668823
H	4.175526142	-1.464850545	0.443958253
C	-0.271286249	1.810797572	1.807611823
H	-1.247357607	2.024885893	2.271461487
H	0.480583876	1.931060076	2.598779678
H	-0.086093403	2.585292578	1.046344042
O	-0.558448553	-1.285879016	2.129043102

================================
W2
================================

Ti	-0.288167268	-1.365073681	0.831273735
P	-2.693835497	-0.752609253	0.392193615
P	0.720858693	1.918910742	-0.787636697
N	-0.353253931	-0.846135199	-1.210790038
C	-2.642020226	-0.876001418	-4.179173470
C	-3.907260656	-0.890152574	-3.576702356
C	-1.462555647	-0.863199711	-3.441077709
H	-0.513573170	-0.832677603	-3.963117123
C	-3.939793587	-0.871516049	-2.178575754
C	-1.487320900	-0.878295958	-2.026538610
H	-4.904985905	-0.857365668	-1.674529910
C	-2.771914721	-0.867916763	-1.414244533
H	-2.574607372	-0.869988441	-5.265998363
C	2.713638783	-1.496504545	-3.251397848
C	1.494434357	-1.683884978	-2.604223490
C	3.389492750	-0.276399642	-3.163461685
H	0.975766420	-2.634326935	-2.668269396
C	0.895548880	-0.652659357	-1.868022561
C	2.797551394	0.750868797	-2.416036844
H	3.325462341	1.697672606	-2.348837376
C	1.555003285	0.596487880	-1.782196522
H	3.153671265	-2.316499710	-3.814393282
C	-5.171007156	-0.944464624	-4.402307510
H	-5.076685905	-0.352067232	-5.319505215
H	-5.414315224	-1.971415997	-4.706769466
H	-6.031211853	-0.559288085	-3.844490767
C	4.733727932	-0.083830193	-3.824524641
H	4.915802956	0.966888487	-4.071915150
H	5.546219826	-0.409046322	-3.163291454
H	4.811588764	-0.667386711	-4.747829437
C	-3.080693483	1.024893403	0.737812042
H	-4.032075882	1.320215464	0.284261942
H	-3.117042542	1.183977246	1.819304824
H	-2.273398876	1.635973573	0.326734573
C	-4.182084084	-1.607138038	1.060561180

H	-5.110643864	-1.173485518	0.675792038
H	-4.136542797	-2.666583300	0.802843750
H	-4.172272682	-1.513583422	2.150546551
C	-0.553355396	2.496449471	-2.025479794
H	-1.036444783	3.400212288	-1.638958097
H	-0.100787103	2.721693754	-2.996903658
H	-1.313540339	1.725091934	-2.163738728
C	1.928995609	3.329720497	-0.951067686
H	1.482505083	4.209582806	-0.477122784
H	2.853153467	3.097467184	-0.415269613
H	2.165528297	3.579531908	-1.991403341
C	-0.686813295	-0.383753538	2.718579769
H	-1.617737055	-0.626375079	3.247043848
H	0.147150204	-0.651672363	3.386749744
H	-0.659379840	0.705715120	2.566555500
C	-0.509806335	-3.199190378	1.049104810
H	-0.626821756	-3.124748707	2.156104326
C	-0.697506130	-4.593399525	0.501090765
C	0.247207209	-5.581399918	1.225152850
H	1.293641329	-5.278848648	1.105924249
H	0.030598385	-5.612019539	2.299025774
H	0.137869865	-6.598546028	0.826234400
C	-2.161056757	-5.045518875	0.718111396
H	-2.309973001	-6.080605507	0.382329643
H	-2.436414242	-4.986291409	1.777098298
H	-2.841558933	-4.402647018	0.151061177
C	-0.392336339	-4.609215736	-1.010919213
H	-0.589201093	-5.597984314	-1.444060802
H	-1.006064892	-3.869260550	-1.534913063
H	0.657904088	-4.362105846	-1.197901011
C	3.200897932	2.624585629	2.491976738
C	2.709707022	1.400252223	2.072436571
C	4.480525494	3.033884287	2.086834192
H	1.717436790	1.067620039	2.352151394
H	4.871218204	3.997061968	2.402796268
C	3.482665062	0.547671497	1.251466513
C	5.259640217	2.217073441	1.278448939
H	6.250319004	2.540473700	0.970935106
C	4.773114204	0.966913640	0.859688044
H	2.593467712	3.268159389	3.119971991
C	4.969945908	-1.496212959	-0.377513260
C	3.653758287	-1.715363622	0.082988329
C	5.650285244	-2.518805742	-1.058726907
H	6.660019398	-2.346179008	-1.420904875
C	3.058453083	-2.976958275	-0.133598834
C	5.040378571	-3.750844717	-1.258523226
H	2.045685053	-3.127437592	0.228030771
H	5.581574917	-4.532278538	-1.784648776
C	3.741916180	-3.988667965	-0.784917891
H	3.265944242	-4.952173710	-0.937993824
S	5.840012550	0.015563342	-0.162653014
C	2.880311728	-0.707670271	0.801749349
O	1.674323797	-0.922919452	1.079286933

==============================

W3

==============================

Ti	-0.817196190	0.405745625	0.892436504
P	-3.273945093	-0.086216412	-0.184092030
P	-0.444814801	2.133086681	-2.946041107
N	-0.581878722	-0.239254296	-1.004958510
C	-1.571844101	-3.090984106	-3.216673374
C	-2.935189009	-3.217973948	-2.915069103
C	-0.771256030	-2.119857788	-2.619528055
H	0.280456483	-2.054527283	-2.873023272
C	-3.479974747	-2.311597824	-1.996841669
C	-1.315668344	-1.223338366	-1.679016829
H	-4.538262367	-2.384456396	-1.758315325
C	-2.700283051	-1.323322773	-1.392212152
H	-1.121569514	-3.772614241	-3.935413837
C	3.110863686	-0.524158776	-1.509425879
C	1.820551157	-0.750066757	-1.042473435
C	3.368938208	0.497481883	-2.431491852
H	1.625666380	-1.528143406	-0.313544452
C	0.745261490	0.034292281	-1.484931707
C	2.293023348	1.279199719	-2.866277695
H	2.498505116	2.068623066	-3.582429171
C	0.978963971	1.070775747	-2.418338776
H	3.926703453	-1.147701144	-1.150754213
C	-3.777533770	-4.303652287	-3.543405533
H	-3.540682077	-4.431351662	-4.605463982
H	-3.610619068	-5.273656368	-3.057266712
H	-4.845991135	-4.079208851	-3.464235067
C	4.772181511	0.753434420	-2.927896500
H	4.776339531	1.401514888	-3.809448957
H	5.381673336	1.239210486	-2.155985355
H	5.276240349	-0.182349488	-3.193945408
C	-3.743731499	1.394143820	-1.189506292
H	-4.441763401	1.116791010	-1.985125303
H	-4.213885307	2.131171703	-0.531091928
H	-2.841925621	1.834861279	-1.620795131
C	-4.939264297	-0.688985229	0.341742426
H	-5.600779057	-0.753747344	-0.527239740
H	-4.868115902	-1.668022513	0.817206383
H	-5.372373581	0.012732346	1.057807326
C	-1.220546246	1.078108191	-4.278558254
H	-1.961903453	1.683827877	-4.810575485
H	-0.477393776	0.712673366	-4.994686604
H	-1.730529666	0.222324267	-3.833717585
C	0.381915241	3.387435198	-4.047154903
H	-0.401305825	4.037399769	-4.449770927
H	1.068073750	4.009474754	-3.465052605
H	0.925312519	2.941499949	-4.887398720
C	-0.698429227	2.474889994	0.947739899
H	-1.595409036	2.837727785	1.473882079
H	0.185996816	2.867868185	1.462517262
H	-0.714857101	2.851483107	-0.083068870
C	-1.300424695	-0.195872396	2.878575325
H	-1.051819563	0.830531716	3.212649584
C	-2.545188904	-0.632413924	3.665531397
C	-3.133020401	-1.971949458	3.174982786
H	-3.184504747	-2.008318424	2.085274458

H	-2.522320509	-2.820079327	3.491318226
H	-4.141707420	-2.120038748	3.579026461
C	-2.243318558	-0.752407849	5.178802967
H	-3.169623137	-0.929211497	5.740449905
H	-1.567308664	-1.582609177	5.388906956
H	-1.783172727	0.164364457	5.563799381
C	-3.591137886	0.492817193	3.504731178
H	-4.534874439	0.226287931	3.994894981
H	-3.229211569	1.423117161	3.958860159
H	-3.793806076	0.701909423	2.452345848
C	2.323580503	1.253711104	5.054988861
C	1.542754769	0.768042028	4.011750698
C	2.586658478	0.436985165	6.159213066
H	1.349151254	1.388757944	3.143911123
H	3.200407267	0.795956850	6.980318069
C	0.987702250	-0.522781789	4.035556793
C	2.075484753	-0.854866445	6.192100048
H	2.300560713	-1.508260727	7.031068802
C	1.279819727	-1.336969495	5.139332771
H	2.731260061	2.259180069	5.003967762
C	0.186070383	-3.396251917	3.664729834
C	-0.024829062	-2.439503908	2.659319401
C	-0.017290235	-4.762358189	3.401646852
H	0.162425593	-5.487358093	4.191236973
C	-0.405747265	-2.911972284	1.393047452
C	-0.432073444	-5.193617821	2.147242785
H	-0.539054811	-2.206286907	0.585593224
H	-0.587939560	-6.253438950	1.965566397
C	-0.621943593	-4.259096622	1.126651406
H	-0.930927336	-4.565653801	0.131568342
S	0.673918128	-2.995310307	5.321319580
C	0.077425353	-0.928327024	2.875338793
O	0.655024946	-0.347292125	1.665577769

==============================

W3-TS

==============================

Ti	-0.919520855	0.230395615	1.348337770
P	-3.482473612	0.163120851	0.089353956
P	-0.168530613	1.898610711	-2.425374269
N	-0.707398117	-0.445968956	-0.553915381
C	-2.031950951	-3.272156000	-2.618190289
C	-3.405417919	-3.217690229	-2.349198341
C	-1.134300947	-2.362301350	-2.067535162
H	-0.079017483	-2.445569038	-2.298541069
C	-3.851695776	-2.175445795	-1.529046655
C	-1.574750900	-1.339625359	-1.199775815
H	-4.917091846	-2.096192122	-1.328366160
C	-2.974986792	-1.239665270	-0.970630050
H	-1.648807883	-4.052492619	-3.273356915
C	2.897073746	-1.333597660	-1.060697436
C	1.581642032	-1.366426706	-0.616345286
C	3.332150936	-0.325076133	-1.930189610
H	1.261426687	-2.132330656	0.078660138
C	0.651030004	-0.402398854	-1.030353308
C	2.403039694	0.633379340	-2.340426207
H	2.743817091	1.413188696	-3.014589787
C	1.063708782	0.616244435	-1.915277600
H	3.597914696	-2.092563391	-0.719842315
C	-4.355124474	-4.250247478	-2.908392429
H	-4.111742020	-4.498373032	-3.947541475
H	-4.312708855	-5.186277390	-2.335909367
H	-5.391689777	-3.899303198	-2.882364273
C	4.770619392	-0.265689522	-2.388326168
H	4.892625809	0.400025249	-3.248313904
H	5.424831867	0.103978150	-1.588870049
H	5.140200615	-1.256951571	-2.673887968
C	-3.661220074	1.575516701	-1.092763066
H	-4.294732571	1.300828099	-1.941517234
H	-4.105655193	2.423814297	-0.562534809
H	-2.670833349	1.869884610	-1.445567489
C	-5.264062881	-0.175084606	0.467076361
H	-5.852198601	-0.265151918	-0.451662838
H	-5.372234344	-1.086947799	1.057170510
H	-5.661877155	0.660798967	1.049051404
C	-1.078219295	0.973764837	-3.771713495
H	-1.739255190	1.673416495	-4.294237614
H	-0.388097852	0.525003254	-4.493822098
H	-1.690177202	0.183592856	-3.333522320
C	0.848406911	3.016695738	-3.516544342
H	0.170796961	3.762793064	-3.943625212
H	1.598441005	3.547065735	-2.922436476
H	1.346748114	2.491818190	-4.339162350
C	-0.953616500	2.318474770	1.435036540
H	-1.698304772	2.627926111	2.185215235
H	0.014262692	2.764510155	1.687738657
H	-1.264281034	2.699231386	0.454046339
C	-1.654595017	-0.567422569	3.261807203
H	-1.721162319	0.420263112	3.735251665
C	-2.924963951	-1.361252308	3.676925421
C	-3.361149549	-2.476677179	2.706331015
H	-3.380823374	-2.134837866	1.668873429
H	-2.691124916	-3.336118221	2.751740217
H	-4.365463257	-2.830607891	2.969340324
C	-2.703039646	-1.988917232	5.073637009
H	-3.628305197	-2.447353125	5.443298340
H	-1.934525609	-2.766186953	5.042665005
H	-2.384964943	-1.230903149	5.797456264
C	-4.056512833	-0.319678634	3.803321362
H	-5.009690285	-0.804689527	4.041498661
H	-3.836489677	0.396156639	4.603990555
H	-4.175030231	0.246055305	2.877709627
C	1.331781387	1.599236965	5.635396957
C	0.643431544	0.967019737	4.611539841
C	1.951347113	0.834428549	6.630451679
H	0.197036237	1.563394070	3.826159239
H	2.501426935	1.314644575	7.434538841
C	0.539604723	-0.436008543	4.537758827
C	1.878742456	-0.548770130	6.575235367
H	2.376474380	-1.151376963	7.330118656
C	1.180943847	-1.188653946	5.535126209
H	1.398562431	2.682808638	5.650998116

C	0.647894681	-3.389455080	3.983073473
C	-0.008094893	-2.499700785	3.114476681
C	0.891776979	-4.718301773	3.594200373
H	1.409081459	-5.384921551	4.278552055
C	-0.392344832	-2.993563652	1.854528904
C	0.497833133	-5.173027992	2.343746662
H	-0.885706425	-2.337595701	1.150775671
H	0.705497384	-6.198691845	2.052516699
C	-0.149827734	-4.300850868	1.462716818
H	-0.458620250	-4.626657963	0.474180579
S	1.130213737	-2.955905437	5.624375343
C	-0.294268489	-1.075115204	3.465998173
O	0.613065600	-0.197051778	1.994249582

==============================

W4

==============================

Ti	-0.868452907	0.518757224	0.598198414
P	-3.179460049	0.091750436	-0.439052135
P	-0.248581007	2.104051828	-2.798580408
N	-0.372952342	-0.341987610	-1.125053525
C	-1.502823949	-3.391963005	-2.948169708
C	-2.875921965	-3.404335022	-2.656537533
C	-0.653046906	-2.404073000	-2.465894461
H	0.404494941	-2.429593325	-2.705905437
C	-3.375234604	-2.358264208	-1.876851678
C	-1.149176002	-1.365434766	-1.652778864
H	-4.441169262	-2.331309795	-1.659555435
C	-2.548331976	-1.340613723	-1.391207576
H	-1.087574959	-4.184142590	-3.567983389
C	3.333775997	-0.560749114	-1.466759086
C	2.024235010	-0.878825843	-1.117979169
C	3.611906528	0.568304360	-2.247151852
H	1.803273320	-1.739427805	-0.492867768
C	0.956527352	-0.081614643	-1.554882050
C	2.540534258	1.360704184	-2.678914309
H	2.763891935	2.236924171	-3.280533314
C	1.212246060	1.057703376	-2.351978064
H	4.150991917	-1.186802030	-1.116294861
C	-3.767809868	-4.515961170	-3.156030416
H	-3.537560701	-4.780766964	-4.193893909
H	-3.643661737	-5.426636219	-2.556004524
H	-4.824751377	-4.235653400	-3.108589172
C	5.037270546	0.941818535	-2.582025766
H	5.087083817	1.597470522	-3.456619024
H	5.510694027	1.472459316	-1.746234417
H	5.646007538	0.055139400	-2.787848949
C	-4.069908619	1.128228545	-1.678220391
H	-4.803426266	0.530866385	-2.228082895
H	-4.577260017	1.955548882	-1.172921062
H	-3.338108301	1.540581465	-2.375714064
C	-4.506291389	-0.571237028	0.646894157
H	-5.282780170	-1.095354915	0.082117952
H	-4.037252426	-1.251333714	1.361368060
H	-4.965762615	0.255334228	1.196135402
C	-0.981381178	1.086563945	-4.183977127
H	-1.789363742	1.657499790	-4.654065609
H	-0.233010009	0.838874400	-4.943657398
H	-1.398782372	0.159257546	-3.786156654
C	0.534426451	3.449316263	-3.821002483
H	-0.269160062	4.063666344	-4.238729954
H	1.152843475	4.092932701	-3.188678980
H	1.142827034	3.064435244	-4.646872520
C	0.233416051	2.137427568	1.362839580
H	-0.121377170	2.453425169	2.494392872
H	1.406513214	1.771004200	1.428274751
H	0.127959982	3.047493935	0.598148882
O	-1.103641272	-0.609293461	1.759211063

==============================

W2’

==============================

Ti	-1.173646927	-0.193474919	1.411829591
P	-3.246013165	0.209195077	0.048699159
P	0.905620754	1.185574412	-1.590440035
N	-0.769541562	-1.066437483	-0.466998667
C	-2.514030457	-2.871092319	-3.250475168
C	-3.833910465	-2.420471430	-3.113637209
C	-1.488776445	-2.449395895	-2.409402370
H	-0.484299451	-2.821793318	-2.572686911
C	-4.081287861	-1.493660808	-2.095723391
C	-1.738881111	-1.542295933	-1.352374792
H	-5.089759350	-1.101377010	-1.973889828
C	-3.073261023	-1.060539722	-1.233447790
H	-2.276610851	-3.575927973	-4.045879364
C	2.329501152	-3.152037621	-0.645577371
C	1.008328676	-2.764976025	-0.437454730
C	3.272563457	-2.238035440	-1.125106335
H	0.281482369	-3.470498562	-0.050624952
C	0.575464249	-1.461807609	-0.719742835
C	2.839355707	-0.938461483	-1.417360425
H	3.575080156	-0.232564077	-1.791777134
C	1.506915689	-0.533212543	-1.246000767
H	2.635761738	-4.167321682	-0.404503852
C	-4.935687542	-2.929994345	-4.012810230
H	-4.590238571	-3.044617414	-5.046634197
H	-5.304342747	-3.912096024	-3.687256336
H	-5.793065548	-2.248826265	-4.022212505
C	4.726681709	-2.618107557	-1.276552200
H	5.200936794	-2.075869799	-2.101486206
H	5.288523674	-2.379140615	-0.364759713
H	4.844430447	-3.690189600	-1.463775754
C	-3.206539392	1.802396417	-0.895525932
H	-3.980190039	1.829878211	-1.669258118
H	-3.343686342	2.636140585	-0.200802505
H	-2.221497536	1.900064707	-1.358094573
C	-4.987691879	0.161122143	0.644226551
H	-5.701533794	0.332681119	-0.167776674
H	-5.186106682	-0.804957509	1.110483885
H	-5.115464687	0.943792999	1.397745490
C	-0.067753322	0.900515795	-3.158780098
H	-0.333499581	1.873630285	-3.585342407

H	0.510581315	0.333748937	-3.895739555
H	-0.984992445	0.353160441	-2.939283609
C	2.415698767	1.962306619	-2.365525246
H	2.122991562	2.946097612	-2.746644258
H	3.195997953	2.114923716	-1.614936948
H	2.821804285	1.375685692	-3.197388411
C	-1.584555030	1.795835853	2.134960413
H	-2.617079973	2.078773499	2.375850201
H	-0.985757232	1.916381359	3.051448345
H	-1.199752688	2.507014990	1.388471603
C	-1.917418242	-1.442963600	2.566351414
H	-2.187955856	-0.660502851	3.316186666
C	-2.394963503	-2.831748486	2.923241854
C	-1.873840690	-3.847756863	1.885342002
H	-2.164748907	-3.546157598	0.873864174
H	-0.781085670	-3.908730745	1.914522767
H	-2.272542000	-4.851774216	2.078662872
C	-1.887446523	-3.228102684	4.330031872
H	-2.208273411	-4.244584084	4.594724178
H	-0.793216348	-3.194144249	4.376121998
H	-2.273341179	-2.541023731	5.091837406
C	-3.941390514	-2.873519421	2.919462919
H	-4.312481403	-3.854638815	3.245362043
H	-4.356131554	-2.111513853	3.588919640
H	-4.319839001	-2.686711550	1.909810901
C	3.646283627	3.162710190	1.035560250
C	2.671579599	2.234627724	1.363706708
C	4.991789818	2.764883280	0.943048298
H	1.620980501	2.499343872	1.416681767
H	5.752815723	3.491580486	0.673831582
C	3.026959419	0.896868289	1.622551322
C	5.366056442	1.449979663	1.189548135
H	6.397981644	1.122280836	1.124950409
C	4.381681919	0.522790015	1.538236618
H	3.370748281	4.192887306	0.834279954
C	3.900579691	-1.694513559	2.186084270
C	2.522136450	-1.428639412	2.268176079
C	4.414040089	-2.951520920	2.513568401
H	5.481251240	-3.123873949	2.426726103
C	1.657100320	-2.448271513	2.708119154
C	3.539979458	-3.939485073	2.948828697
H	0.594594359	-2.225333452	2.758368254
H	3.933008909	-4.918695927	3.207369328
C	2.160524130	-3.688921452	3.056425095
H	1.487929463	-4.470417023	3.395649672
O	4.803106785	-0.752646267	1.781170368
C	2.022585869	-0.112651192	1.916510820
O	0.801116645	0.164789781	1.893606782

==============================

W2'-TS

==============================

Ti	-1.011884212	0.353360683	0.832873046
P	-3.297983646	0.737674713	-0.309916168
P	0.414522231	1.372135043	-1.729614377
N	-1.085260034	-0.941702247	-0.841101468
C	-3.255980015	-2.585079670	-3.427614927
C	-4.495146275	-1.964506626	-3.213479519
C	-2.121146441	-2.267274380	-2.689716339
H	-1.192264676	-2.774023771	-2.918601036
C	-4.534315586	-0.964637399	-2.240213394
C	-2.168139219	-1.299644232	-1.657329321
H	-5.465786457	-0.426045060	-2.071879625
C	-3.408783436	-0.630832314	-1.483053803
H	-3.174086094	-3.346419573	-4.201508045
C	1.904940009	-3.022077799	-1.747235417
C	0.630017221	-2.681720972	-1.299597502
C	2.810108900	-2.040444851	-2.165626526
H	-0.043656845	-3.451343298	-0.939525485
C	0.205688521	-1.345557928	-1.292773843
C	2.383616209	-0.704774737	-2.158469439
H	3.080990314	0.060713872	-2.489011288
C	1.095483303	-0.346247733	-1.749661684
H	2.209562302	-4.066306591	-1.744927764
C	-5.723498821	-2.374343157	-3.990030527
H	-5.482673168	-2.593881369	-5.036222458
H	-6.182497501	-3.279133558	-3.569791079
H	-6.486063480	-1.588809133	-3.980030298
C	4.219822884	-2.400985241	-2.572273731
H	4.622225761	-1.692519665	-3.303627491
H	4.892943859	-2.390178680	-1.705537558
H	4.268186092	-3.403418541	-3.009585381
C	-3.457217455	2.261422873	-1.346077561
H	-4.387396812	2.246003389	-1.922756553
H	-3.441134930	3.139981270	-0.695077002
H	-2.609451056	2.318255424	-2.029685259
C	-4.897267342	0.774295688	0.609589458
H	-5.737236500	0.943504095	-0.071546152
H	-5.043907642	-0.170521215	1.132495642
H	-4.870433331	1.583445311	1.344323039
C	-0.355489880	1.414700508	-3.425428867
H	-0.736404300	2.419471502	-3.636503458
H	0.364050955	1.134638190	-4.201583862
H	-1.191778779	0.710264623	-3.449111223
C	1.926343441	2.416342974	-1.991684318
H	1.618024707	3.464707136	-2.050406218
H	2.586585999	2.302151680	-1.126938105
H	2.477246284	2.164286613	-2.904394388
C	-1.659324646	2.285475492	1.567214370
H	-2.722025633	2.544997931	1.608527541
H	-1.277988195	2.383757830	2.586964130
H	-1.153627038	3.030317783	0.935740709
C	-1.474054217	-0.709728420	2.409376383
H	-1.733111739	-0.028292166	3.231490374
C	-1.940167189	-2.136597157	2.699344873
C	-1.216805100	-3.205303431	1.861678481
H	-1.240238667	-2.925117731	0.806867778
H	-0.176426291	-3.329367638	2.162605047
H	-1.717960715	-4.173635960	1.978134751
C	-1.880905271	-2.499630690	4.198287010
H	-2.327721357	-3.487223387	4.367873669
H	-0.859355748	-2.524176598	4.574516773

H	-2.441131592	-1.770803332	4.796402454
C	-3.435326576	-2.146183968	2.273714066
H	-3.884287834	-3.118098497	2.515944958
H	-4.003180981	-1.371941447	2.801166296
H	-3.528992653	-1.983219624	1.196375489
C	0.867680848	2.795958519	4.921853542
C	0.847679198	2.107408047	3.712216139
C	0.868367016	2.088640213	6.129099369
H	0.851831615	2.634469271	2.765792608
H	0.871369421	2.621870518	7.075357437
C	0.809939682	0.707531035	3.685897827
C	0.907043278	0.695772409	6.125245571
H	0.967569530	0.119779088	7.042674541
C	0.909180760	0.021493174	4.906669617
H	0.881677270	3.881702185	4.925168514
C	1.571392179	-1.958160639	3.828456640
C	1.486516953	-1.357862592	2.562082291
C	2.181806803	-3.196409464	4.008283138
H	2.201795340	-3.630983591	5.002213001
C	2.145702839	-1.972681522	1.493726373
C	2.770768404	-3.828708887	2.913217783
H	2.142796278	-1.471636772	0.536538601
H	3.249596119	-4.794382095	3.046020985
C	2.775702477	-3.202505589	1.661949396
H	3.259439945	-3.674039125	0.812354922
O	1.050214410	-1.347580791	4.949407578
C	0.756461740	-0.076942176	2.425590992
O	0.851633668	0.594352901	1.267866015

==============================

W3’

==============================

Ti	-0.807549357	0.476708531	0.822876215
P	-3.302719593	0.158451200	-0.256501347
P	-0.402118117	2.106609106	-3.040269136
N	-0.597405016	-0.219735473	-1.048954010
C	-1.789030790	-3.056263924	-3.179677486
C	-3.158445835	-3.080848217	-2.879418850
C	-0.923758984	-2.128785133	-2.603713989
H	0.130117923	-2.140212297	-2.857362509
C	-3.639084339	-2.114403009	-1.987390876
C	-1.404555678	-1.170628667	-1.689821720
H	-4.700740814	-2.105494022	-1.752732277
C	-2.794751167	-1.165088177	-1.405722260
H	-1.385817409	-3.785321712	-3.879426479
C	3.072915554	-0.660382211	-1.624303818
C	1.784778595	-0.828571737	-1.128407836
C	3.350608110	0.330620944	-2.573566675
H	1.579900503	-1.581124425	-0.376653880
C	0.729200304	-0.016130615	-1.567495346
C	2.295233250	1.140310168	-3.005877972
H	2.515112400	1.907910705	-3.741292477
C	0.983589470	0.990419269	-2.527893305
H	3.872427702	-1.304529905	-1.265641332
C	-4.072651386	-4.122163296	-3.480894089
H	-3.840123415	-4.298067093	-4.536942482
H	-3.976775169	-5.086295128	-2.964834929
H	-5.122829437	-3.820480585	-3.415569544
C	4.753163815	0.528170466	-3.098209620
H	4.764673233	1.162127852	-3.989952803
H	5.393296719	1.003713250	-2.344929218
H	5.218979359	-0.428878754	-3.357952833
C	-3.691854239	1.605572939	-1.340063930
H	-4.399249554	1.322014213	-2.124963999
H	-4.127057552	2.399159670	-0.724318981
H	-2.767266750	1.977312803	-1.786958814
C	-5.001138687	-0.315598190	0.294176489
H	-5.659890652	-0.433338523	-0.571246743
H	-4.984309673	-1.241705894	0.869724154
H	-5.403160095	0.479021430	0.927296579
C	-1.261628985	1.064794898	-4.331846714
H	-1.992080569	1.695223212	-4.850342274
H	-0.556524575	0.658896923	-5.064558029
H	-1.794210076	0.237247527	-3.860163450
C	0.449466884	3.303511858	-4.186834335
H	-0.317188084	3.973947048	-4.587976933
H	1.170699835	3.911873817	-3.633442879
H	0.957674265	2.819555759	-5.028079033
C	-0.719955385	2.548677444	0.867277801
H	-1.540325284	2.918141127	1.501071453
H	0.226520851	2.939372301	1.258886933
H	-0.861873686	2.917423010	-0.157177106
C	-1.297258377	-0.213016793	2.772218227
H	-1.138028622	0.805541277	3.175546885
C	-2.511096239	-0.813224196	3.498390198
C	-3.052906513	-2.084486008	2.810797930
H	-3.119453192	-1.955031276	1.727777243
H	-2.402992964	-2.943725824	2.986462116
H	-4.048607826	-2.336438656	3.195859194
C	-2.175451279	-1.162591219	4.968435764
H	-3.081114769	-1.478498936	5.501653194
H	-1.454397798	-1.979433775	5.035318375
H	-1.754826665	-0.298682690	5.493946552
C	-3.601734638	0.279072523	3.524372101
H	-4.534013271	-0.101983361	3.957498550
H	-3.275927782	1.134246826	4.128639221
H	-3.812993526	0.651100576	2.519966841
C	2.136976719	1.169214487	5.315524101
C	1.425270438	0.816962183	4.172761917
C	2.402912617	0.204208761	6.293920040
H	1.219890475	1.551264524	3.399754047
H	2.956058979	0.469797969	7.190356255
C	0.961838901	-0.490081429	3.981384039
C	1.972054601	-1.105549574	6.114158154
H	2.178295135	-1.884237885	6.841302872
C	1.266099215	-1.441653609	4.957339764
H	2.487463474	2.189150810	5.444134712
C	0.503940165	-3.215047121	3.605615616
C	0.151909247	-2.353826523	2.561023712
C	0.472752094	-4.604273319	3.452352524
H	0.751529932	-5.222923756	4.299151897
C	-0.191581801	-2.935188532	1.333437324

C	0.106612004	-5.153892040	2.228705406
H	-0.454174697	-2.295052052	0.500279546
H	0.085923672	-6.233335972	2.108165741
C	-0.219536647	-4.314570904	1.158034682
H	-0.499751449	-4.727495193	0.193550751
O	0.887738049	-2.761395216	4.844665527
C	0.121107645	-0.850339770	2.770991802
O	0.698566735	-0.199383482	1.598771214

==============================

W3'-TS

==============================

Ti	-0.914189279	0.214732111	1.365303159
P	-3.483167410	0.278869182	0.077301122
P	-0.218652204	1.764884830	-2.523140907
N	-0.712274194	-0.493662655	-0.511185348
C	-2.156505823	-3.194571733	-2.656844854
C	-3.533967018	-3.016728878	-2.477828026
C	-1.220577955	-2.377302170	-2.030528784
H	-0.163049608	-2.545078278	-2.198611975
C	-3.937762499	-1.960137486	-1.653611779
C	-1.624347687	-1.326839566	-1.180033326
H	-5.002994061	-1.793895364	-1.514454007
C	-3.021534443	-1.116318107	-1.016840339
H	-1.802739620	-3.996800184	-3.301825762
C	2.885411501	-1.380469680	-1.044773698
C	1.581316352	-1.390467167	-0.564644992
C	3.287711620	-0.439012378	-1.999584675
H	1.282428145	-2.103388786	0.194035873
C	0.632681310	-0.466741651	-1.022979617
C	2.339468718	0.480750293	-2.454305649
H	2.656516790	1.211135387	-3.192343712
C	1.014244199	0.491689742	-1.988785386
H	3.601926088	-2.106499195	-0.666912675
C	-4.536571980	-3.942755222	-3.124988317
H	-4.213255882	-4.246819019	-4.126545429
H	-4.673207760	-4.860787392	-2.537951946
H	-5.518983841	-3.468786240	-3.219874382
C	4.713667393	-0.397750705	-2.497499228
H	4.793594837	0.144281924	-3.445015192
H	5.370022774	0.104055472	-1.775222301
H	5.113968372	-1.405969739	-2.650302887
C	-3.640521288	1.722913384	-1.069085121
H	-4.298303604	1.486103535	-1.911052108
H	-4.049972534	2.571726561	-0.512061119
H	-2.648970127	1.995002151	-1.436852932
C	-5.272566795	-0.030434715	0.451571435
H	-5.871965408	-0.062934920	-0.463672906
H	-5.397887707	-0.967041492	0.998080730
H	-5.645465374	0.786389887	1.075758457
C	-1.165143490	0.831466258	-3.838292122
H	-1.807454586	1.541183352	-4.370810032
H	-0.494047076	0.348615468	-4.556264877
H	-1.799345851	0.069608830	-3.382224083
C	0.789623797	2.849944115	-3.655534506
H	0.112101823	3.598015308	-4.079568863
H	1.560371995	3.379385471	-3.087871790
H	1.261384487	2.305962563	-4.481471062
C	-0.997524023	2.310175657	1.451740026
H	-1.870047808	2.612689495	2.051932812
H	-0.095594041	2.762670994	1.877250791
H	-1.131121039	2.691840887	0.431076080
C	-1.685837388	-0.630814791	3.221346617
H	-1.814383864	0.360275358	3.675894499
C	-2.944395542	-1.470592022	3.589295864
C	-3.362271547	-2.524286270	2.543818951
H	-3.371520758	-2.115928650	1.530311584
H	-2.686575413	-3.381309509	2.546484947
H	-4.366890430	-2.902371645	2.768956900
C	-2.728985548	-2.182538748	4.945025921
H	-3.654228926	-2.670644999	5.274111271
H	-1.954340816	-2.951174021	4.872482777
H	-2.426601171	-1.468382597	5.718760967
C	-4.090528488	-0.452238888	3.772390842
H	-5.040763855	-0.962314963	3.965553045
H	-3.887549162	0.210332289	4.621998310
H	-4.205994606	0.173206061	2.885564089
C	1.095713735	1.527632713	5.896231174
C	0.405322522	0.959995031	4.836777210
C	1.860018015	0.716295540	6.747888565
H	-0.155711904	1.592560768	4.157706261
H	2.401597500	1.154503107	7.581152439
C	0.455740184	-0.423367560	4.587798119
C	1.938834667	-0.649170458	6.517750263
H	2.532652617	-1.305706263	7.144814968
C	1.251103401	-1.205533504	5.434128284
H	1.051477194	2.600640297	6.056429386
C	0.934986293	-3.140050411	4.128993511
C	0.101429142	-2.465763330	3.223244667
C	1.334270835	-4.463890553	3.915511370
H	1.968692183	-4.935309887	4.658591270
C	-0.261354089	-3.152004242	2.050076008
C	0.936227620	-5.123445988	2.760738373
H	-0.847603559	-2.638664722	1.297195315
H	1.257124424	-6.146172047	2.586734056
C	0.143858850	-4.458015919	1.813835740
H	-0.146502629	-4.950980663	0.891353071
O	1.394817591	-2.561192513	5.277369976
C	-0.317390949	-1.071603298	3.497910261
O	0.617463589	-0.149548247	2.041567802

==============================

W4'

==============================

Ti	-0.868452907	0.518757224	0.598198414
P	-3.179460049	0.091750436	-0.439052135
P	-0.248581007	2.104051828	-2.798580408
N	-0.372952342	-0.341987610	-1.125053525
C	-1.502823949	-3.391963005	-2.948169708
C	-2.875921965	-3.404335022	-2.656537533
C	-0.653046906	-2.404073000	-2.465894461
H	0.404494941	-2.429593325	-2.705905437

C	-3.375234604	-2.358264208	-1.876851678
C	-1.149176002	-1.365434766	-1.652778864
H	-4.441169262	-2.331309795	-1.659555435
C	-2.548331976	-1.340613723	-1.391207576
H	-1.087574959	-4.184142590	-3.567983389
C	3.333775997	-0.560749114	-1.466759086
C	2.024235010	-0.878825843	-1.117979169
C	3.611906528	0.568304360	-2.247151852
H	1.803273320	-1.739427805	-0.492867768
C	0.956527352	-0.081614643	-1.554882050
C	2.540534258	1.360704184	-2.678914309
H	2.763891935	2.236924171	-3.280533314
C	1.212246060	1.057703376	-2.351978064
H	4.150991917	-1.186802030	-1.116294861
C	-3.767809868	-4.515961170	-3.156030416
H	-3.537560701	-4.780766964	-4.193893909
H	-3.643661737	-5.426636219	-2.556004524
H	-4.824751377	-4.235653400	-3.108589172
C	5.037270546	0.941818535	-2.582025766
H	5.087083817	1.597470522	-3.456619024
H	5.510694027	1.472459316	-1.746234417
H	5.646007538	0.055139400	-2.787848949
C	-4.069908619	1.128228545	-1.678220391
H	-4.803426266	0.530866385	-2.228082895
H	-4.577260017	1.955548882	-1.172921062
H	-3.338108301	1.540581465	-2.375714064
C	-4.506291389	-0.571237028	0.646894157
H	-5.282780170	-1.095354915	0.082117952
H	-4.037252426	-1.251333714	1.361368060
H	-4.965762615	0.255334228	1.196135402
C	-0.981381178	1.086563945	-4.183977127
H	-1.789363742	1.657499790	-4.654065609
H	-0.233010009	0.838874400	-4.943657398
H	-1.398782372	0.159257546	-3.786156654
C	0.534426451	3.449316263	-3.821002483
H	-0.269160062	4.063666344	-4.238729954
H	1.152843475	4.092932701	-3.188678980
H	1.142827034	3.064435244	-4.646872520
C	0.233416051	2.137427568	1.362839580
H	-0.121377170	2.453425169	2.494392872
H	1.406513214	1.771004200	1.428274751
H	0.127959982	3.047493935	0.598148882
O	-1.103641272	-0.609293461	1.759211063

==============================

W5’

==============================

Ti	-0.359083295	-0.146054164	0.972056329
P	-2.746674538	-0.127016619	-0.015473673
P	2.171814919	-0.041021626	0.542204499
N	-0.123668596	-0.850812554	-0.992420673
C	-1.777962446	-3.744982004	-2.686462641
C	-3.117393017	-3.583394289	-2.296776772
C	-0.784896672	-2.861722231	-2.282323122
H	0.243448913	-3.018436909	-2.592386961
C	-3.424490690	-2.490435600	-1.483994842
C	-1.090518594	-1.753174186	-1.468313694
H	-4.456152916	-2.345314264	-1.171406746
C	-2.442295551	-1.581233859	-1.076874256
H	-1.507679701	-4.591949940	-3.313782930
C	1.820955276	0.137102976	-4.032377720
C	0.779073656	-0.345701188	-3.250352859
C	3.030040741	0.583110452	-3.475288868
H	-0.143768132	-0.666385710	-3.722570181
C	0.895621002	-0.411902308	-1.844238043
C	3.146882534	0.530517578	-2.084542990
H	4.069512844	0.876401663	-1.622574210
C	2.113888025	0.046859551	-1.275759459
H	1.688342214	0.181270763	-5.111852169
C	-4.174808502	-4.573966503	-2.722534657
H	-4.076226711	-4.834670544	-3.782151699
H	-4.098600388	-5.508642197	-2.152575016
H	-5.182766914	-4.176931381	-2.567716837
C	4.150536060	1.090391994	-4.351578712
H	4.952548504	1.539111495	-3.757382631
H	4.593820572	0.281804264	-4.946455002
H	3.795265913	1.849691629	-5.058206081
C	-3.261188984	1.203736305	-1.188149095
H	-4.131572723	0.895001292	-1.775206208
H	-3.497018337	2.116859674	-0.633764982
H	-2.425494432	1.408572197	-1.862183690
C	-4.272581577	-0.499239624	0.940496206
H	-5.129950047	-0.696213126	0.289621204
H	-4.083185196	-1.368033290	1.575317621
H	-4.503961563	0.355984539	1.581463695
C	3.212652445	1.370076656	1.098398089
H	3.300596476	1.329495549	2.187605381
H	4.213983059	1.345363617	0.658668518
H	2.714713573	2.303883076	0.828202546
C	3.193209410	-1.528736591	0.921866477
H	3.311112881	-1.625596523	2.004775763
H	2.661490917	-2.408503294	0.551668823
H	4.175526142	-1.464850426	0.443958253
C	-0.271286249	1.810797572	1.807611942
H	-1.247357607	2.024885893	2.271461487
H	0.480583876	1.931060076	2.598779678
H	-0.086093403	2.585292578	1.046344042
O	-0.558448553	-1.285879016	2.129043102

==============================

X1

==============================

Ti	-0.264411300	-1.092679620	0.770030499
P	-2.609103918	-0.109877251	0.466915876
P	2.154995918	-1.293963790	0.018299885
N	-0.284061164	-0.475989878	-1.275118232
C	-2.728728056	-1.431584597	-3.940406799
C	-3.948810816	-1.386613607	-3.249272346
C	-1.524881601	-1.112261891	-3.325809240
H	-0.599272966	-1.199135184	-3.884844065
C	-3.909055948	-0.990876794	-1.910425425
C	-1.473485351	-0.716779292	-1.970394135

H	-4.842415333	-0.944397092	-1.353819251
C	-2.710613489	-0.651413500	-1.274915457
H	-2.720034122	-1.746307850	-4.982330799
C	1.860266685	1.468162656	-3.641980886
C	0.722456753	0.940561771	-3.044872522
C	3.153013229	1.150613189	-3.193751097
H	-0.257715315	1.234950423	-3.405269861
C	0.815192282	0.046258528	-1.951779723
C	3.250729322	0.283690333	-2.101734638
H	4.238112450	0.034600403	-1.716711998
C	2.119947672	-0.259836316	-1.484131575
H	1.743577957	2.164290667	-4.470945358
C	-5.242387772	-1.781324148	-3.921168089
H	-5.317055702	-1.359606147	-4.929981232
H	-5.325737476	-2.871331453	-4.021672249
H	-6.111727238	-1.438645601	-3.350852251
C	4.377720833	1.718527675	-3.871742010
H	5.259210110	1.656752110	-3.225605249
H	4.613964558	1.180662155	-4.799334526
H	4.236072540	2.771580696	-4.142971516
C	-2.997508287	1.700338721	0.321927875
H	-3.929057837	1.844721794	-0.233925730
H	-3.085220575	2.157794237	1.310511231
H	-2.180170536	2.183740854	-0.219165891
C	-4.127636433	-0.745521247	1.297376990
H	-5.045264721	-0.395640105	0.813907087
H	-4.101814270	-1.836522937	1.300331831
H	-4.125859261	-0.391950279	2.332993984
C	3.446282387	-0.496807665	1.068015456
H	3.529875278	-1.046441555	2.009211302
H	4.423953056	-0.462891370	0.577539980
H	3.111956358	0.519241333	1.290683746
C	2.959005356	-2.872689724	-0.501737118
H	3.096469641	-3.511695623	0.375763267
H	2.292905331	-3.384931803	-1.200059772
H	3.927076340	-2.695493937	-0.981831729
C	0.575643361	-1.019555092	2.788993120
H	-0.253484994	-1.059390426	3.518710613
H	1.218185186	-1.877652645	3.028993607
H	1.145977974	-0.104324706	2.988459349
C	-0.863838196	-2.833822012	0.627113640
H	-0.723154306	-2.869964838	1.740863085
C	-1.322602868	-4.158453941	0.067964926
C	-0.510844231	-5.305086136	0.711938500
H	0.559369266	-5.170372963	0.520452380
H	-0.658269107	-5.328295231	1.798064590
H	-0.811381400	-6.280232430	0.306686342
C	-2.825358152	-4.362235069	0.371995002
H	-3.171600819	-5.339867592	0.010899282
H	-3.017885923	-4.310675621	1.450191021
H	-3.417783260	-3.586884737	-0.124465413
C	-1.116678476	-4.189080238	-1.458915710
H	-1.400640845	-5.165562153	-1.872426152
H	-1.716758370	-3.420313120	-1.948614120
H	-0.066967219	-4.002060890	-1.710461020
C	-1.935833097	2.349511862	4.340356827
C	-1.172698736	1.942565322	3.258093357
C	-2.038637400	3.717773676	4.646896839
H	-1.077132821	0.895207524	3.002989292
H	-2.636619329	4.041173458	5.494034290
C	-0.498562545	2.883817196	2.456722260
C	-1.379583240	4.667024612	3.878705978
H	-1.440740705	5.727997780	4.095952988
C	-0.610451281	4.246487617	2.788673878
H	-2.449483156	1.614087105	4.951338768
C	0.778573513	4.905091286	0.997269154
C	0.956473351	3.575655460	0.575808048
C	1.382808208	5.967325211	0.318900079
H	1.217877746	6.978922367	0.673897207
C	1.761990786	3.326582193	-0.550526083
C	2.172545910	5.693959713	-0.790613592
H	1.889315248	2.300789356	-0.870964408
H	2.642472744	6.516001225	-1.323285937
C	2.365090847	4.372726917	-1.228579164
H	2.978880167	4.164910316	-2.099127054
O	0.008702285	5.233762741	2.077798605
C	0.292853355	2.475425720	1.287407517
O	0.392224252	1.300369859	0.910252452

================================

^{3}X1-TS

================================

Ti	-0.040854022	-0.745258808	0.702525556
P	-2.262333632	0.598336160	0.080554001
P	2.422949314	-1.126547933	-0.008542806
N	-0.043267056	-0.600498855	-1.389647007
C	-2.605192900	-1.810658693	-3.830914974
C	-3.796187639	-1.310321808	-3.283449888
C	-1.371766090	-1.572771311	-3.233314991
H	-0.464485735	-1.974587917	-3.674659729
C	-3.700268030	-0.557236552	-2.109539509
C	-1.273210526	-0.812416196	-2.052963972
H	-4.612140656	-0.165157855	-1.664145708
C	-2.470309019	-0.306279898	-1.493500948
H	-2.647477388	-2.408910990	-4.738896847
C	2.039190292	1.021260262	-4.035581589
C	0.922743320	0.525443852	-3.376440763
C	3.341339588	0.862194300	-3.531367302
H	-0.063970849	0.698433340	-3.792124987
C	1.047852278	-0.165417030	-2.147877216
C	3.471540689	0.176937446	-2.322589874
H	4.464441299	0.049384929	-1.894577742
C	2.361403227	-0.326351345	-1.634662509
H	1.896187067	1.569589257	-4.965184689
C	-5.131343365	-1.606224179	-3.924923897
H	-5.099592209	-1.450362325	-5.009109974
H	-5.431540489	-2.648580313	-3.758264780
H	-5.921854496	-0.968064368	-3.518140554
C	4.534790039	1.417288065	-4.272080421
H	5.422916412	1.453063607	-3.633440495
H	4.785336971	0.809333324	-5.151090622
H	4.343151569	2.434983015	-4.631947517

C	-2.356376648	2.374268770	-0.440894157
H	-3.246077776	2.545720816	-1.055343986
H	-2.393337488	3.015275240	0.443959653
H	-1.461953759	2.628504276	-1.013845801
C	-3.860694885	0.388654500	0.980005622
H	-4.702740192	0.833458126	0.440572798
H	-4.052483082	-0.675550163	1.134183288
H	-3.766686440	0.875868917	1.955287218
C	3.637205839	-0.154380769	0.977532268
H	3.757396460	-0.623504221	1.958156228
H	4.612921715	-0.088908143	0.487299740
H	3.223546743	0.845468044	1.123212695
C	3.279332638	-2.740820169	-0.270374328
H	3.388672829	-3.253835201	0.689991534
H	2.671936035	-3.362100601	-0.933014333
H	4.266558647	-2.594175100	-0.719501615
C	0.678034306	-1.987048149	2.488761902
H	-0.002665683	-2.028439760	3.343731880
H	1.039302349	-2.994849443	2.276582956
H	1.523052454	-1.351548553	2.782212257
C	-1.405635595	-2.095369339	1.239678979
H	-2.125198603	-1.855935335	2.036589622
C	-1.563911200	-3.535407543	0.792719364
C	-1.598988295	-4.498622894	2.001017809
H	-0.660276055	-4.470268250	2.560415983
H	-2.409917593	-4.232341766	2.688653946
H	-1.764782429	-5.529632092	1.664538622
C	-2.927129984	-3.611299992	0.054253057
H	-3.117721319	-4.643297195	-0.266370416
H	-3.750613213	-3.306500196	0.710767269
H	-2.935544252	-2.968389034	-0.828478038
C	-0.449472666	-3.960923195	-0.184550032
H	-0.612674892	-4.985537052	-0.538150012
H	-0.422810674	-3.299704075	-1.056071997
H	0.529909909	-3.924667835	0.301291198
C	-2.151934147	1.719330072	4.707344532
C	-1.244835377	1.391982555	3.705684900
C	-2.557884216	3.047590017	4.888207436
H	-0.916496277	0.368583590	3.556463957
H	-3.267591715	3.303427219	5.669110775
C	-0.717605352	2.389208317	2.852994680
C	-2.048844814	4.053807735	4.059916496
H	-2.345443726	5.091627121	4.172607899
C	-1.142717361	3.725506544	3.060925484
H	-2.544849396	0.939481139	5.353764057
C	0.122173890	4.488127232	1.192612410
C	0.579750061	3.176787376	0.916107833
C	0.444076121	5.559807777	0.371636510
H	0.063015088	6.543556213	0.626387477
C	1.364001632	2.985836983	-0.243497878
C	1.231421471	5.345182419	-0.765217006
H	1.692417622	1.983263731	-0.486922950
H	1.477528811	6.182449818	-1.411550403
C	1.684207797	4.056162357	-1.071134686
H	2.278237104	3.879420519	-1.963139176
O	-0.685801804	4.757975101	2.275038004
C	0.179955035	2.103975296	1.776035428
O	0.563112736	0.864905536	1.519443512

================================

^{3}X2

================================

Ti	-0.546341360	-0.383918941	0.441077709
P	-1.996322393	-2.504028082	0.169391558
P	0.561929047	1.764374137	-0.369755596
N	-0.356824040	-0.754336715	-1.586917877
C	-2.682120323	-1.797346234	-4.328886509
C	-3.531309605	-2.705103874	-3.675899506
C	-1.625048041	-1.178032756	-3.672744751
H	-1.010204077	-0.463881075	-4.210129261
C	-3.281099558	-2.943106174	-2.323556185
C	-1.352678061	-1.431954980	-2.308135986
H	-3.941060305	-3.618448973	-1.781510949
C	-2.230228424	-2.326056004	-1.635962605
H	-2.863741398	-1.557118773	-5.374833584
C	2.532915592	-0.382224172	-3.938010454
C	1.407969832	-0.911600113	-3.318175793
C	3.091463089	0.841727674	-3.534169912
H	1.008646011	-1.865425587	-3.647967577
C	0.764505625	-0.231866494	-2.261916161
C	2.479320288	1.502566934	-2.468386650
H	2.904466629	2.443313599	-2.125088453
C	1.340965033	0.993535638	-1.832005858
H	2.997671127	-0.936925471	-4.750599861
C	-4.663978100	-3.383727551	-4.407938480
H	-5.193156719	-2.683008671	-5.063788891
H	-4.301867008	-4.204725742	-5.040257931
H	-5.395707130	-3.804932117	-3.711347342
C	4.316980362	1.401945710	-4.216099262
H	4.561834335	2.400989532	-3.842242241
H	5.194190502	0.763543427	-4.052928448
H	4.172926903	1.478285789	-5.300379276
C	-3.701867342	-2.373550177	0.866890430
H	-4.362891674	-3.159341574	0.489626288
H	-3.643619061	-2.445393324	1.956880331
H	-4.116905212	-1.398387313	0.605220735
C	-1.609634876	-4.294899940	0.416128159
H	-2.382990360	-4.924540997	-0.035412084
H	-0.645319998	-4.520864964	-0.043509599
H	-1.546511769	-4.509137154	1.487104058
C	-0.537594676	3.097890854	-1.028139234
H	-0.990970075	3.636059284	-0.190054417
H	0.020288525	3.799573183	-1.656146169
H	-1.340349913	2.641148567	-1.611944079
C	1.901235700	2.714019537	0.470415264
H	1.482015371	3.175249338	1.369024038
H	2.690597534	2.022687912	0.774796784
H	2.325422287	3.497283697	-0.165355861
C	1.507726908	-2.506309271	1.082458973
H	2.517641068	-2.830204487	1.375991106
H	0.807594299	-3.251006842	1.476712346
H	1.464359164	-2.577021599	-0.013055764

C	1.158553958	-1.086329341	1.565442085
H	2.003879070	-0.435185015	1.270796418
C	1.016106367	-0.948837578	3.108846188
C	-0.264689445	-1.644751430	3.621931553
H	-1.166437149	-1.190041065	3.199012280
H	-0.272609621	-2.708469629	3.358736753
H	-0.337269902	-1.576222539	4.714918613
C	2.229106188	-1.558234811	3.848062754
H	2.167894840	-1.372748017	4.928234100
H	2.287982464	-2.641834021	3.702858210
H	3.165255308	-1.119699359	3.482455730
C	0.945253730	0.548704565	3.474041224
H	0.809865355	0.689963460	4.553866386
H	1.870973229	1.060220599	3.184572697
H	0.116185494	1.047882318	2.962067127
C	-4.397054195	1.157639146	-2.107386827
C	-3.548368931	0.948330522	-1.026922584
C	-5.634915352	1.781439304	-1.916875362
H	-2.589959383	0.474460393	-1.188338280
H	-6.302120209	1.947967291	-2.757325411
C	-3.913115025	1.341472507	0.280778825
C	-6.020640373	2.191864729	-0.637128413
H	-6.973544121	2.678229809	-0.454788536
C	-5.172795773	1.969408512	0.440117538
H	-4.091881275	0.821088314	-3.093731165
C	-4.849750519	2.177036047	2.787513494
C	-3.584896326	1.545895457	2.715294361
C	-5.380210400	2.617425203	3.992948294
H	-6.354160786	3.096006155	3.987391710
C	-2.872537136	1.369989276	3.922477484
C	-4.653446674	2.433024168	5.171904087
H	-1.903699756	0.888175547	3.880696774
H	-5.066224575	2.775744677	6.116046429
C	-3.400667191	1.807887912	5.131011963
H	-2.836197615	1.664194703	6.047940254
O	-5.620950222	2.389184713	1.668250799
C	-3.094607353	1.115409136	1.438979864
O	-1.919577360	0.524769604	1.343719006

==============================

Y1

==============================

Ti	-0.492291391	-0.597208023	0.838615000
P	-2.985944510	-0.739288628	0.253515095
P	-0.844872296	-3.601651907	-2.790859222
N	-0.644328892	-0.846109748	-1.335121274
C	-2.687722921	0.585352063	-4.114215374
C	-3.953099489	0.714272499	-3.520111561
C	-1.589974284	0.076536894	-3.429423094
H	-0.629890919	0.005201470	-3.929939747
C	-4.066538334	0.312933534	-2.185495615
C	-1.698101401	-0.341092944	-2.078110933
H	-5.031437397	0.407436013	-1.689183235
C	-2.978999615	-0.194867581	-1.472654819
H	-2.556080341	0.901234269	-5.148325920
C	2.841021299	-0.731663704	-2.648975849
C	1.634852409	-0.368121386	-2.056611538
C	2.975196123	-1.969475389	-3.288843393
H	1.532618999	0.591204166	-1.561200857
C	0.519419014	-1.223510027	-2.051232100
C	1.852450252	-2.804610014	-3.327808380
H	1.951342940	-3.756146669	-3.843488455
C	0.627521932	-2.466025352	-2.732154131
H	3.685481310	-0.045927752	-2.614230633
C	-5.131959915	1.248420477	-4.297947884
H	-4.856299400	2.123522520	-4.898190975
H	-5.535683155	0.499200672	-4.992063999
H	-5.947456837	1.547724962	-3.631869316
C	4.290569782	-2.399290323	-3.897193432
H	4.147466183	-3.167730093	-4.663907528
H	4.813312054	-1.555747747	-4.361372471
H	4.966103554	-2.818544149	-3.139273643
C	-4.212634087	0.317908376	1.130623937
H	-5.199616909	0.278917998	0.659337401
H	-4.294250011	-0.017893936	2.167419195
H	-3.851943016	1.349416494	1.126826048
C	-3.804891586	-2.398099899	0.210113063
H	-4.834822655	-2.310684681	-0.148607031
H	-3.245902777	-3.033293724	-0.483620852
H	-3.801746845	-2.848825932	1.207277894
C	-0.038860735	-5.245093346	-3.170883894
H	-0.825728536	-6.005403042	-3.207208395
H	0.493415803	-5.262854576	-4.128187180
H	0.660189152	-5.510878563	-2.373704433
C	-1.500232577	-3.244266748	-4.503684521
H	-2.254623890	-3.997689486	-4.755395889
H	-1.971577168	-2.259857416	-4.515891552
H	-0.703688025	-3.271727324	-5.255597591
C	-0.585757196	1.554875135	0.660494506
H	-1.609000683	1.766803265	0.313099086
H	-0.425596803	2.164048195	1.560998201
H	0.076469317	1.921410799	-0.135188341
C	-0.825396955	-1.065712571	2.623863459
H	-0.594646871	0.018049976	2.812069893
C	-1.157432199	-1.789988518	3.900891066
C	0.005987397	-1.612331033	4.906105518
H	0.940394223	-1.993450165	4.480398655
H	0.153620914	-0.553573251	5.147681713
H	-0.192459166	-2.150869370	5.842110634
C	-2.451337576	-1.208412409	4.516757488
H	-2.690245867	-1.696021795	5.471134663
H	-2.347402096	-0.132934973	4.698408604
H	-3.298769712	-1.355427861	3.838657618
C	-1.356526494	-3.293347120	3.629703045
H	-1.625491619	-3.828199148	4.549077034
H	-2.155842543	-3.454592228	2.897707939
H	-0.441372126	-3.740144014	3.229762793
C	2.584085941	-1.372372866	0.895368218
N	1.773625612	-0.291758001	1.044524550
C	3.980008364	-1.268361211	0.972886980
C	2.354302406	0.899787128	1.286116242
C	4.562067986	-0.030730102	1.204186678

C	3.728566170	1.077949643	1.370946169
H	4.601758480	-2.144840717	0.839727163
H	1.675983310	1.733582616	1.403188467
H	5.642227173	0.069497444	1.254396319
H	4.132787228	2.067720652	1.555963159
C	1.891799688	-2.647277355	0.672475994
C	2.537062168	-3.885128498	0.541620851
N	0.544970989	-2.551269531	0.643539667
C	1.773470879	-5.036159039	0.394983619
C	-0.187972143	-3.664905071	0.486653894
C	0.380159646	-4.926144600	0.372238368
H	3.617825747	-3.949330330	0.557808936
H	2.254829407	-6.004355431	0.295563281
H	-1.260703921	-3.526301384	0.461942345
H	-0.256297529	-5.795801163	0.250916988

===============================

^{3}Y1-TS

===============================

Ti	-0.589360595	-0.924140513	0.740582705
P	-3.118384600	-0.950577736	-0.033570495
P	-0.565099239	-3.391935110	-2.535076141
N	-0.640565574	-0.562655568	-1.322447062
C	-2.556131363	0.887852788	-4.183316231
C	-3.876462221	0.832687974	-3.709414005
C	-1.477532864	0.431948453	-3.434175730
H	-0.474060357	0.491375566	-3.841589689
C	-4.073061943	0.287740499	-2.437766790
C	-1.671701789	-0.102865905	-2.138875246
H	-5.086783886	0.217004195	-2.046262741
C	-3.005053997	-0.171524808	-1.663717389
H	-2.366777658	1.304398417	-5.171383381
C	2.844314337	-0.206525937	-2.617728233
C	1.600375414	0.095705137	-2.077769279
C	3.119675875	-1.493564963	-3.097293139
H	1.397199035	1.084876657	-1.679633141
C	0.588442266	-0.872495174	-1.981518507
C	2.092664719	-2.440001488	-3.057613611
H	2.300072908	-3.430246115	-3.453690290
C	0.825489283	-2.158574820	-2.522393703
H	3.618454218	0.557231128	-2.647699118
C	-5.026350498	1.325979233	-4.555514812
H	-4.819994926	2.318582535	-4.973125458
H	-5.220963478	0.656802654	-5.403812885
H	-5.951098919	1.396357059	-3.974194527
C	4.495728970	-1.850020885	-3.609482288
H	4.479205608	-2.762914658	-4.213171482
H	4.918071747	-1.047195435	-4.224056244
H	5.191838741	-2.017780066	-2.777159691
C	-4.428187847	-0.015601757	0.869401276
H	-5.363136768	0.021096991	0.302430987
H	-4.621182919	-0.492927700	1.834523916
H	-4.075543404	1.003145576	1.048239350
C	-3.925580263	-2.582855940	-0.355148315
H	-4.871307850	-2.458810568	-0.891742527
H	-3.237752914	-3.178958416	-0.961291194
H	-4.109075069	-3.097149611	0.593882501
C	0.350939363	-4.993993282	-2.813972712
H	-0.383928776	-5.805381298	-2.829125881
H	0.912175477	-5.023496628	-3.754518747
H	1.034375668	-5.172988415	-1.979539156
C	-1.184502244	-3.107159376	-4.275723457
H	-1.929628015	-3.871183157	-4.521664619
H	-1.661608577	-2.125590801	-4.331076622
H	-0.371490359	-3.149260998	-5.008926868
C	-0.756783307	1.104446888	1.667906165
H	-1.756155252	1.425814390	1.973431587
H	-0.066796005	1.315909386	2.488671541
H	-0.471838593	1.708503246	0.791301250
C	-1.494212627	-1.127150893	2.555180788
H	-2.550297022	-0.882465482	2.721461535
C	-0.874947369	-1.406206250	3.915401936
C	0.662680864	-1.484398603	3.910541296
H	1.017626882	-2.277315140	3.249660969
H	1.104421973	-0.544778347	3.570322037
H	1.035362005	-1.693533540	4.920444965
C	-1.318414688	-0.329722643	4.933342934
H	-0.918771684	-0.553159475	5.930435181
H	-0.955191851	0.658819735	4.635904312
H	-2.410930634	-0.280627459	5.008887291
C	-1.438927650	-2.784529686	4.353775024
H	-1.069174051	-3.037945032	5.355558395
H	-2.534329414	-2.776441097	4.389710426
H	-1.121509194	-3.572118998	3.663261175
C	2.337330818	-1.819089055	0.785275757
N	1.572976232	-0.673444629	0.906576693
C	3.744413853	-1.750467777	0.715273201
C	2.231275320	0.509873509	0.992048562
C	4.385465145	-0.533708513	0.802527964
C	3.603564024	0.632331669	0.955734313
H	4.319429874	-2.661679029	0.593119919
H	1.605420828	1.386997819	1.084908247
H	5.468603134	-0.475431561	0.750143051
H	4.060273647	1.613422394	1.031622767
C	1.588452101	-3.044974089	0.765177965
C	2.144225359	-4.342690945	0.742374122
N	0.221478090	-2.874365568	0.827328503
C	1.323849559	-5.448932648	0.812579274
C	-0.570011795	-3.976090431	0.894330740
C	-0.078301601	-5.261287212	0.899539649
H	3.220012426	-4.465619087	0.683854103
H	1.747763991	-6.448587894	0.803819478
H	-1.634322166	-3.776269913	0.944127023
H	-0.759018719	-6.103728294	0.952673018

===============================

^{3}Y2

===============================

Ti	-0.394336790	-0.699652970	0.398977667
P	-2.968603134	-0.781808078	-0.239079282
P	0.354885489	-3.101085663	-2.012883902
N	-0.487090945	-0.294717759	-1.667572618

C	-2.643365860	0.996700704	-4.438850880
C	-3.927565813	0.947022498	-3.872705460
C	-1.509157300	0.587005377	-3.751821280
H	-0.545656979	0.642075717	-4.244180679
C	-4.021433830	0.432426512	-2.580277205
C	-1.587383270	0.106996372	-2.418549061
H	-5.004888058	0.346492589	-2.120527029
C	-2.894347668	0.014686271	-1.862799048
H	-2.528609276	1.370110869	-5.455288887
C	2.754511833	0.686348617	-3.246468067
C	1.483961225	0.777922451	-2.687644958
C	3.357535362	-0.562154472	-3.458001137
H	1.029211760	1.746190190	-2.496354580
C	0.766935647	-0.377209097	-2.333280802
C	2.634785175	-1.709159970	-3.116554976
H	3.105424881	-2.675359726	-3.273964643
C	1.344683886	-1.642334938	-2.571085453
H	3.297526121	1.594783306	-3.500905037
C	-5.139766216	1.406462669	-4.647952557
H	-5.015216351	2.431267262	-5.018622398
H	-5.325602055	0.771009088	-5.523477554
H	-6.042727947	1.386875391	-4.029439449
C	4.759755135	-0.643314481	-4.016742706
H	5.003992081	-1.804130197	-4.295496941
H	4.842082500	0.068509132	-5.023411751
H	5.552416801	-0.230238050	-3.169808388
C	-4.263014317	0.121668771	0.718039751
H	-5.225768089	0.138446689	0.198888928
H	-4.394879818	-0.357396722	1.693053007
H	-3.922824621	1.147643805	0.880092144
C	-3.760551453	-2.417410851	-0.591303587
H	-4.709244251	-2.289967775	-1.121513128
H	-3.072457314	-2.993710995	-1.215355515
H	-3.928983450	-2.971192122	0.336840242
C	1.531239390	-4.510273457	-2.320981741
H	1.012300730	-5.445176125	-2.088200331
H	1.896834731	-4.562107086	-3.352655411
H	2.384108782	-4.418396473	-1.641038060
C	-0.785494387	-3.289656162	-3.477711201
H	-1.403394699	-4.184385777	-3.346862316
H	-1.441418409	-2.416538954	-3.530947924
H	-0.231062591	-3.371145964	-4.418555737
C	-0.707419336	2.195905209	0.617879450
H	-0.998103976	3.146983147	1.089471221
H	0.248387828	2.373628616	0.110352151
H	-1.445176244	1.999762177	-0.169761702
C	-0.611164868	1.027075291	1.620204687
H	-1.640358448	0.787235498	1.963664770
C	0.191142514	1.335928321	2.915697336
C	1.588204265	1.904657125	2.604124546
H	2.172098637	1.203425884	2.003828049
H	1.528834462	2.853981256	2.061293125
H	2.141959190	2.091587543	3.532409906
C	-0.573124170	2.360747337	3.787754536
H	-0.021202946	2.584648371	4.709567547
H	-0.728263736	3.308413506	3.261126518
H	-1.557385921	1.969473600	4.072939396
C	0.358409852	0.042665977	3.737313986
H	0.822248757	0.250365317	4.709877491
H	-0.612620234	-0.432520688	3.922358274
H	0.984974265	-0.681651235	3.209825754
C	1.939483643	-2.425534248	1.139327526
N	1.669354081	-1.179203153	0.588249207
C	3.263086557	-2.929247379	1.142456770
C	2.713155746	-0.440571874	0.130874693
C	4.298378468	-2.163906097	0.652670622
C	4.019934177	-0.869122267	0.147218183
H	3.461660624	-3.916474819	1.544394732
H	2.449144602	0.524516940	-0.284408778
H	5.314350128	-2.547734976	0.657647967
H	4.805061817	-0.231239811	-0.242875606
C	0.810273826	-3.115359545	1.692898154
C	0.875186563	-4.374053955	2.335853338
N	-0.403645456	-2.448926687	1.590697408
C	-0.254183382	-4.928245068	2.895746708
C	-1.493809104	-3.005897760	2.176042080
C	-1.479780555	-4.215473175	2.830380678
H	1.822174072	-4.898457527	2.396860361
H	-0.203918666	-5.892668247	3.392160654
H	-2.404660940	-2.420579910	2.103596449
H	-2.386202097	-4.604440212	3.281200171

================================

^{3}Y2-TS

================================

Ti	-0.177156955	-0.166230187	0.496800601
P	-2.816583157	-0.229201317	0.127196535
P	-0.766148031	-3.222326994	-2.261083603
N	-0.426602960	-0.244616538	-1.516313672
C	-2.632150173	0.717691243	-4.365811825
C	-3.887433290	0.821534395	-3.747399807
C	-1.488192201	0.366355687	-3.658805370
H	-0.533743918	0.298376113	-4.170191765
C	-3.947216988	0.553719342	-2.377060890
C	-1.546209335	0.103313722	-2.272351503
H	-4.911549568	0.612856269	-1.874683499
C	-2.812350273	0.197527036	-1.643501282
H	-2.548477888	0.921645164	-5.431934357
C	2.932558775	-0.496561378	-3.110936165
C	1.784652948	0.024661299	-2.528279543
C	3.013333797	-1.857674599	-3.433043003
H	1.727235675	1.073036432	-2.254477024
C	0.685797215	-0.793344319	-2.224269867
C	1.896975636	-2.659060955	-3.178988457
H	1.955585003	-3.710630894	-3.446494341
C	0.727063596	-2.160632372	-2.583460808
H	3.781806231	0.155211523	-3.303869247
C	-5.120089054	1.176604986	-4.544962883
H	-4.927930355	2.010793686	-5.229476452
H	-5.461478710	0.331892103	-5.157652855
H	-5.950915813	1.465431929	-3.893534184
C	4.283828259	-2.438665390	-4.008258343

H	4.122493267	-3.442036152	-4.414247036
H	4.686311245	-1.810574174	-4.811062813
H	5.063206673	-2.514873028	-3.239079952
C	-3.943006992	1.022720098	0.893819451
H	-4.927167892	1.036148429	0.416950077
H	-4.069527626	0.799985468	1.958031178
H	-3.484772205	2.010483265	0.794855952
C	-3.849910021	-1.766718268	0.183260590
H	-4.826074600	-1.603263140	-0.284163207
H	-3.315050125	-2.547365904	-0.365067929
H	-3.996334076	-2.088017225	1.220106840
C	-0.006745788	-4.926612377	-2.215908766
H	-0.802132428	-5.647500515	-2.000830173
H	0.475877464	-5.221008778	-3.154229641
H	0.723374307	-4.976394653	-1.403910398
C	-1.472892523	-3.266613483	-3.991128922
H	-2.288007498	-3.997110605	-4.028056622
H	-1.877947450	-2.281801462	-4.236998081
H	-0.714935958	-3.537324190	-4.734430790
C	-0.780059457	1.359893441	2.518286467
H	-1.729575753	1.746645212	2.173303604
H	-0.049346719	2.120360136	2.770598888
H	-0.170862257	1.591423273	0.783050776
C	-0.675393760	0.084576592	3.028439283
H	-1.568360925	-0.541506052	2.993686676
C	0.322925329	-0.311786711	4.128922939
C	1.716757655	0.316470593	3.951475620
H	2.195560932	-0.036392421	3.037746429
H	1.673836470	1.409982324	3.909946918
H	2.356223106	0.048329789	4.800571442
C	-0.285032034	0.202134997	5.461170673
H	0.354072392	-0.064661123	6.312419415
H	-0.395422757	1.291565299	5.442459583
H	-1.276110053	-0.233129144	5.634372711
C	0.474112689	-1.841091037	4.233836651
H	1.009034276	-2.107192039	5.153147221
H	-0.503406167	-2.336753845	4.262533188
H	1.032411337	-2.250088930	3.391951799
C	2.556079149	-1.381281137	0.656886518
N	1.924938202	-0.149114534	0.578811884
C	3.959365845	-1.475338459	0.535935342
C	2.695483446	0.960786998	0.430145323
C	4.717274189	-0.335576236	0.370394588
C	4.066657066	0.922707498	0.325667113
H	4.439906597	-2.446578026	0.575352311
H	2.140629292	1.892198563	0.387752920
H	5.797192574	-0.402254701	0.278602809
H	4.627954483	1.842997789	0.204059571
C	1.679502726	-2.498212576	0.888520241
C	2.108438253	-3.832077026	1.071907282
N	0.336049587	-2.184309006	0.977180719
C	1.193740129	-4.822542667	1.363028169
C	-0.543320715	-3.174188375	1.258988738
C	-0.177093938	-4.486615658	1.464074969
H	3.163011312	-4.072528839	0.996115923
H	1.524121642	-5.846082687	1.513084888
H	-1.580135822	-2.865639687	1.319150567
H	-0.929348230	-5.235406876	1.685687065

================================

^{3}Y3

================================

Ti	-0.570369303	-0.494728833	0.450433642
P	-3.079165220	-0.747794271	-0.220404759
P	-0.423923105	-3.209830523	-2.500329733
N	-0.502820909	-0.279085189	-1.538633704
C	-2.479579210	0.782236516	-4.519014835
C	-3.796056986	0.718797743	-4.039300442
C	-1.388152480	0.446418881	-3.724452972
H	-0.381381094	0.519646525	-4.122814655
C	-3.976234674	0.284616917	-2.723040581
C	-1.569281459	0.029116003	-2.389504910
H	-4.989477158	0.203857705	-2.332258701
C	-2.899421930	-0.069121242	-1.906687617
H	-2.303765059	1.110986948	-5.541613102
C	3.037178516	-0.074182875	-2.700702190
C	1.787491441	0.278522968	-2.204523563
C	3.281625509	-1.377048016	-3.157821655
H	1.601073503	1.282558799	-1.836735249
C	0.742174923	-0.656140268	-2.128161907
C	2.232334614	-2.298789024	-3.098197699
H	2.420301914	-3.307184935	-3.457844973
C	0.965780914	-1.975094795	-2.586824656
H	3.833345175	0.666206121	-2.729635000
C	-4.969162464	1.067659140	-4.924396515
H	-4.757604599	1.947789550	-5.541578770
H	-5.216173172	0.246002406	-5.609119892
H	-5.865938187	1.281947136	-4.334473610
C	4.643156052	-1.769400716	-3.682898998
H	4.628998756	-2.766477585	-4.133427143
H	4.998008728	-1.063089013	-4.442382336
H	5.390379906	-1.778986812	-2.879674435
C	-4.358231068	0.298097342	0.597474456
H	-5.285800934	0.347393662	0.019274302
H	-4.573010445	-0.115836605	1.586979508
H	-3.946953058	1.300825238	0.730495036
C	-3.979133606	-2.339513302	-0.511823297
H	-4.901365280	-2.172071218	-1.076012254
H	-3.319613457	-2.998144150	-1.083096862
H	-4.223589897	-2.812073708	0.445279270
C	0.513191402	-4.811988831	-2.318392992
H	-0.215520233	-5.628175735	-2.278907061
H	1.212462664	-5.018263817	-3.135611773
H	1.057934999	-4.801850319	-1.370231390
C	-0.845654845	-3.291649103	-4.317981720
H	-1.574285626	-4.091619015	-4.486706257
H	-1.299573421	-2.341376781	-4.613904953
H	0.037843391	-3.473858833	-4.939987659
H	-1.219860435	0.667872727	1.594626307
C	2.159990549	-1.298643470	1.322413325
N	1.412862897	-0.163603276	1.055771828
C	3.550251007	-1.189264417	1.552742004

C	2.031608582	1.043395758	1.061522126
C	4.161115646	0.045164391	1.532143950
C	3.379566431	1.202318907	1.287173390
H	4.135999203	-2.080874681	1.746610999
H	1.381872058	1.892282605	0.869646311
H	5.229473114	0.129607067	1.707500339
H	3.823369741	2.191657543	1.275993943
C	1.414992929	-2.525790453	1.344297767
C	1.961137772	-3.800101042	1.617079854
N	0.054773148	-2.397692204	1.076175094
C	1.153848290	-4.917163372	1.639445066
C	-0.718900383	-3.516302586	1.103837848
C	-0.233946040	-4.772676468	1.380428433
H	3.023265123	-3.896275759	1.814789891
H	1.576028109	-5.894608974	1.852298379
H	-1.767189384	-3.355867147	0.879333913
H	-0.902105510	-5.626706600	1.383553982

================================

^{3}Y4

================================

Ti	-0.658317268	-0.540014327	0.522018313
P	-3.094333410	-0.393907696	-0.242889285
P	-0.720751882	-3.603399992	-2.502985716
N	-0.567310512	-0.674605548	-1.540075779
C	-2.211349487	0.598750770	-4.641359806
C	-3.520159006	0.838884532	-4.196230412
C	-1.227845907	0.086803481	-3.800036669
H	-0.222933918	-0.075341269	-4.175892353
C	-3.806370735	0.539081037	-2.859752893
C	-1.515947342	-0.215836048	-2.449808359
H	-4.812719822	0.718566060	-2.484673023
C	-2.839359760	0.023430469	-1.996510625
H	-1.952068925	0.827667952	-5.673491478
C	3.036788940	-0.817755759	-2.481151104
C	1.787878156	-0.338490307	-2.097823143
C	3.192200661	-2.152431726	-2.869925737
H	1.673493624	0.687930882	-1.765138268
C	0.659307182	-1.171107888	-2.070251226
C	2.053822994	-2.967401505	-2.896481276
H	2.179723740	-3.998388529	-3.213940859
C	0.783904731	-2.510741711	-2.515499830
H	3.898352623	-0.154210612	-2.457558632
C	-4.577149391	1.379503846	-5.129876137
H	-4.186004639	2.192644358	-5.752145290
H	-4.949863434	0.604151547	-5.812059879
H	-5.437816620	1.768779874	-4.576569557
C	4.554581165	-2.707396507	-3.216253042
H	4.477849960	-3.630826235	-3.798887730
H	5.146451473	-1.990750194	-3.796406269
H	5.128515720	-2.940051079	-2.309422016
C	-4.287606239	0.864018142	0.395290524
H	-5.259103298	0.782516420	-0.101614438
H	-4.423052788	0.711482406	1.469977379
H	-3.878505945	1.863217950	0.235269696
C	-4.112466335	-1.930058956	-0.295276046
H	-5.037025928	-1.782588005	-0.862037063
H	-3.506460905	-2.708538055	-0.766634166
H	-4.346225739	-2.240181684	0.727835715
C	0.036686223	-5.297478676	-2.704576731
H	-0.781162202	-6.024652004	-2.735332012
H	0.628680944	-5.411628723	-3.619449854
H	0.665125310	-5.525147915	-1.839473605
C	-1.325695634	-3.385002136	-4.259663105
H	-2.105431080	-4.130264759	-4.452075481
H	-1.759090185	-2.390850544	-4.383648396
H	-0.519129276	-3.516669750	-4.989405632
H	-1.657153726	-1.822465420	0.955663621
C	2.414166212	-0.772752941	1.033622146
N	1.427583933	0.179232955	0.964815795
C	3.771537304	-0.411890924	1.178107142
C	1.799759030	1.474320173	1.095070481
C	4.125793457	0.916886568	1.282462478
C	3.108625174	1.890064597	1.252780557
H	4.535583496	-1.178986907	1.202869892
H	0.999860406	2.198133945	1.063109159
H	5.168150425	1.202511549	1.390951276
H	3.330903292	2.947369576	1.349224806
C	1.954230428	-2.136616468	0.949105382
C	2.814848661	-3.252495050	0.995311618
N	0.587254584	-2.286197901	0.826910496
C	2.300133944	-4.531139374	0.933588326
C	0.106582589	-3.560356855	0.774473071
C	0.901875734	-4.685715199	0.826311350
H	3.884666204	-3.103995562	1.081093311
H	2.956630468	-5.395195007	0.971626520
H	-0.968288958	-3.632368326	0.684932292
H	0.441277951	-5.667006493	0.781148076
C	-1.558425188	1.349144101	2.753144979
N	-1.178027987	0.041059457	2.513887167
C	-1.963753343	1.738017559	4.055756569
C	-1.494420767	2.221066713	1.617365241
C	-1.144771457	-0.833040714	3.545339823
C	-1.934031367	0.828032315	5.088752747
H	-2.291604757	2.755819082	4.236764908
C	-1.497568965	-0.497157425	4.835918427
H	-0.821758628	-1.835831642	3.283914089
H	-2.239168406	1.124688268	6.088242531
H	-1.444604516	-1.237454176	5.626682281
C	-1.773165226	3.610020638	1.649963975
N	-1.095723748	1.624203563	0.429449975
C	-1.636819243	4.375697136	0.513645947
H	-2.083817720	4.070954800	2.581301212
C	-0.951666951	2.401363850	-0.669309080
C	-1.205568314	3.757806063	-0.686651587
H	-1.848048329	5.440706730	0.542431593
H	-0.617877424	1.883949637	-1.558894396
H	-1.077361465	4.322132587	-1.603492737

================================

^{3}Y4-TS

================================

Ti	-0.647246003	-1.034508228	0.465033263
P	-3.126273394	-0.930941999	-0.128522009
P	-0.572605669	-3.675852537	-3.099526405
N	-0.608176470	-0.945529461	-1.590865731
C	-2.534169674	0.384387165	-4.499575138
C	-3.823627949	0.517464817	-3.961603165
C	-1.470898032	-0.108777054	-3.750701666
H	-0.482128531	-0.174253881	-4.192730904
C	-4.003565311	0.122774996	-2.632581949
C	-1.647722960	-0.505040705	-2.403413534
H	-4.994058132	0.213097140	-2.188828230
C	-2.956593752	-0.382721066	-1.859099507
H	-2.354683161	0.690506697	-5.528781414
C	2.969342709	-0.681703031	-2.561993122
C	1.700401425	-0.367713660	-2.089464903
C	3.208319426	-1.899569988	-3.209869146
H	1.518576741	0.571396947	-1.577385902
C	0.628437281	-1.263918877	-2.217734337
C	2.126527786	-2.767880917	-3.383463860
H	2.306617498	-3.705157518	-3.904670954
C	0.840336323	-2.484010696	-2.901336670
H	3.785665035	0.024419509	-2.422111034
C	-4.967205524	1.049062729	-4.792708874
H	-4.672249794	1.941018105	-5.357791424
H	-5.317900658	0.307973981	-5.522809505
H	-5.822901249	1.321589112	-4.166529179
C	4.596466064	-2.274975777	-3.674251556
H	4.566616535	-3.022152185	-4.473807812
H	5.144085407	-1.402760029	-4.046841145
H	5.188102722	-2.700542212	-2.852901459
C	-4.234342098	0.305930793	0.680812240
H	-5.215209007	0.355929255	0.198493138
H	-4.366230488	0.026698407	1.730371356
H	-3.757442474	1.287179708	0.641980290
C	-4.195626736	-2.432577610	-0.230417341
H	-5.148531437	-2.216117620	-0.724078238
H	-3.653022289	-3.192054272	-0.799015045
H	-4.379902363	-2.813974142	0.778755128
C	0.320320606	-5.316237450	-3.023144960
H	-0.418592274	-6.114216805	-3.151480913
H	1.094759107	-5.442218304	-3.787029505
H	0.779043853	-5.431427956	-2.036066294
C	-0.826599300	-3.554232597	-4.944713116
H	-1.526627541	-4.330933571	-5.269605160
H	-1.262961745	-2.577917576	-5.172161102
H	0.114484116	-3.664463282	-5.494908810
H	-1.370011568	-2.772646427	0.792626560
C	2.386855841	-1.164339662	1.570133805
N	1.573294759	-0.175268456	1.132589698
C	3.654333115	-0.903881133	2.100872040
C	2.002655029	1.091282129	1.222452044
C	4.091560364	0.413628936	2.185190678
C	3.250780821	1.433988810	1.737749696
H	4.285180092	-1.725849509	2.421793938
H	1.315962553	1.851622701	0.867500067
H	5.074424744	0.641692340	2.588142633
H	3.549890041	2.475852966	1.787223816
C	1.800918818	-2.512833834	1.451400638
C	2.107177973	-3.594066381	2.270050287
N	0.843223691	-2.566130638	0.508374929
C	1.277012348	-4.733102322	2.174194574
C	-0.016777365	-3.635111094	0.438550353
C	0.217900306	-4.768281460	1.292542219
H	2.913670063	-3.546376228	2.991411924
H	1.479246140	-5.594040871	2.806958437
H	-0.475522697	-3.800255537	-0.532494426
H	-0.432780236	-5.633560658	1.222788572
C	-1.127440214	0.390402466	3.120589256
N	-0.883477271	-0.884530544	2.638602257
C	-1.282424450	0.591500521	4.517469406
C	-1.195210814	1.430705786	2.136131048
C	-0.793060422	-1.908745766	3.509775639
C	-1.180284739	-0.470931709	5.387942314
H	-1.480077028	1.586580634	4.901140213
C	-0.927188218	-1.765889287	4.879160881
H	-0.608638704	-2.880320311	3.066218376
H	-1.296474099	-0.311950356	6.456689835
H	-0.842686474	-2.628295660	5.531208515
C	-1.387052655	2.806065559	2.429133892
N	-1.036664486	1.032092810	0.810934603
C	-1.395065904	3.744709969	1.424488544
H	-1.516999722	3.118803501	3.459303379
C	-1.045666575	1.975450635	-0.164396048
C	-1.214545488	3.321109056	0.077905491
H	-1.536075711	4.795975208	1.658093572
H	-0.914962947	1.595642924	-1.171139240
H	-1.216207385	4.026583672	-0.745475233

==============================

Y5

==============================

Ti	-0.447260052	-0.546579301	0.563602984
P	-2.948851109	-0.785855293	-0.067360133
P	-0.307803452	-3.164473295	-2.793542624
N	-0.436104894	-0.350541830	-1.472250581
C	-2.488035917	0.830054581	-4.348186493
C	-3.784500360	0.806687593	-3.811695099
C	-1.377221584	0.434371084	-3.611341715
H	-0.386550397	0.479456127	-4.052861691
C	-3.923887730	0.345685899	-2.499372721
C	-1.514540195	-0.006225766	-2.275806427
H	-4.921330929	0.295891106	-2.064681053
C	-2.827483892	-0.064833060	-1.737902880
H	-2.343952179	1.178699851	-5.369550228
C	3.104101658	0.016583927	-2.564232349
C	1.837690949	0.302383780	-2.065931797
C	3.379585266	-1.227650285	-3.141488791
H	1.633977413	1.266965032	-1.611722112
C	0.806200087	-0.648532808	-2.097656965
C	2.337157011	-2.158356667	-3.212125063
H	2.543464661	-3.120254040	-3.675808191
C	1.056732416	-1.902969360	-2.701521873

H	3.889084816	0.767307997	-2.499720812
C	-4.976224899	1.235901356	-4.634698391
H	-4.784991741	2.182162046	-5.154250622
H	-5.226733208	0.493090808	-5.403271198
H	-5.864747524	1.374009252	-4.010429382
C	4.765998840	-1.565484405	-3.637530804
H	4.739286900	-2.333219528	-4.417376041
H	5.272912025	-0.685028255	-4.046737194
H	5.392718792	-1.950236917	-2.822656870
C	-4.149155617	0.278626740	0.845151722
H	-5.112062454	0.349734783	0.331016004
H	-4.304894924	-0.136496350	1.845309377
H	-3.715926647	1.275633454	0.951309502
C	-3.919466019	-2.335075617	-0.352027237
H	-4.878124714	-2.112953901	-0.831067264
H	-3.333030224	-2.986816645	-1.004551768
H	-4.097505569	-2.847078562	0.598904371
C	0.636590421	-4.726434231	-2.401547909
H	-0.053794716	-5.574233532	-2.464617729
H	1.477487922	-4.916316509	-3.077017784
H	1.010361671	-4.670237541	-1.375526428
C	-0.429112524	-3.317502499	-4.652319431
H	-1.101029754	-4.143930912	-4.906216621
H	-0.857408762	-2.392271757	-5.049434662
H	0.543333590	-3.491047859	-5.126449108
H	-1.713871956	-3.335923433	1.381443381
C	2.323466778	-1.542377949	1.073385358
N	1.652583241	-0.362410605	1.010758996
C	3.720646620	-1.551478624	1.194560528
C	2.322357416	0.801243246	1.129097819
C	4.409940720	-0.351042032	1.290897846
C	3.697971344	0.855885088	1.274456859
H	4.257047653	-2.491998672	1.210667372
H	1.709155083	1.695544600	1.089311123
H	5.492014885	-0.351305753	1.385517597
H	4.200893402	1.812971830	1.363038659
C	1.468001485	-2.745536804	1.035224557
C	1.983416796	-3.997575283	1.279963136
N	0.134643242	-2.478399754	0.852836430
C	1.063226819	-5.091444492	1.483006239
C	-0.815452397	-3.609703541	0.803174078
C	-0.260542721	-4.913958073	1.311450839
H	3.042983532	-4.153392792	1.431949377
H	1.452625632	-6.052827835	1.810167551
H	-1.176193714	-3.729724407	-0.232086807
H	-0.966085613	-5.718281269	1.506574631
C	-1.366741776	1.043193340	3.002995968
N	-1.038639188	-0.241662353	2.588152409
C	-1.805636048	1.270300746	4.330236435
C	-1.232626677	2.055950403	2.001337767
C	-1.123777270	-1.255314708	3.494146347
C	-1.903512239	0.224907026	5.220007420
H	-2.069576263	2.275056839	4.641510963
C	-1.544856310	-1.081102490	4.790690422
H	-0.809935331	-2.224854469	3.124434948
H	-2.243241549	0.397519648	6.236817837
H	-1.587479711	-1.929365516	5.465616226
C	-1.467393637	3.436991215	2.214540005
N	-0.848316252	1.593920827	0.750527978
C	-1.319932580	4.337008476	1.184232473
H	-1.755617738	3.783156872	3.201252460
C	-0.725451410	2.504284859	-0.251917988
C	-0.939427853	3.855766773	-0.093251228
H	-1.492060065	5.396424770	1.349521637
H	-0.451198071	2.088666916	-1.213255882
H	-0.823681772	4.524225235	-0.939721107

==============================

N1

==============================

Ti	-0.320480049	-1.154794335	0.760837138
P	-2.700265408	-0.300226301	0.480066746
P	2.109779119	-1.290407419	0.069175027
N	-0.342341572	-0.550046921	-1.307995558
C	-2.808326960	-1.491164207	-3.965704441
C	-4.030650616	-1.418286085	-3.281665087
C	-1.601951361	-1.196002603	-3.345778942
H	-0.679554224	-1.311929345	-3.903838396
C	-3.981983185	-1.039148211	-1.939231634
C	-1.540471196	-0.792847216	-1.990489602
H	-4.914385796	-0.986405194	-1.381405354
C	-2.779049873	-0.729182363	-1.293707848
H	-2.799453735	-1.808419108	-5.006864071
C	1.861162186	1.168427706	-3.800843239
C	0.711599648	0.706220865	-3.174921274
C	3.146252871	0.856826544	-3.326517344
H	-0.258758157	0.999002874	-3.561528683
C	0.776466727	-0.115482911	-2.019806385
C	3.219003439	0.076810353	-2.171841860
H	4.199029922	-0.161597043	-1.761689067
C	2.075243950	-0.398039281	-1.519194365
H	1.760207057	1.805708528	-4.677855492
C	-5.330585003	-1.779879928	-3.959835768
H	-5.381095409	-1.373097301	-4.976109028
H	-5.450295925	-2.868078232	-4.043267250
H	-6.193299770	-1.398614168	-3.404157877
C	4.381167412	1.354874134	-4.038925171
H	5.280483723	1.211480141	-3.431840181
H	4.538775921	0.829064965	-4.989442825
H	4.305316925	2.423171759	-4.274683475
C	-3.229791880	1.478362203	0.520444512
H	-4.210533619	1.601362586	0.050053082
H	-3.274453878	1.836481810	1.553567886
H	-2.493224621	2.077159643	-0.020473899
C	-4.145510197	-1.128844619	1.271542668
H	-5.099947929	-0.806872249	0.843311250
H	-4.034213066	-2.208678961	1.162293553
H	-4.143268108	-0.883920193	2.338011265
C	3.330265284	-0.344235480	1.082189441
H	3.438361168	-0.833824754	2.053630352
H	4.309636593	-0.274390280	0.598986447
H	2.930507183	0.659399331	1.245505333

C	3.013529062	-2.862091303	-0.277646393
H	3.148365259	-3.412560940	0.658045053
H	2.405997038	-3.469737530	-0.951923668
H	3.990135670	-2.672238350	-0.735272467
C	0.432036549	-1.075107813	2.806913376
H	-0.456352979	-0.981454670	3.460698366
H	0.943348944	-1.992279887	3.128382683
H	1.097142220	-0.232117131	3.031219721
C	-0.824911416	-2.921725988	0.552132964
H	-0.727202535	-2.987247467	1.668434858
C	-1.162650347	-4.255775452	-0.068563640
C	-0.263227761	-5.354231358	0.543881893
H	0.793807447	-5.122572899	0.374294430
H	-0.420570701	-5.433217525	1.625973344
H	-0.474806964	-6.334348202	0.096840799
C	-2.645647764	-4.603693962	0.199889943
H	-2.900356770	-5.587629318	-0.216116831
H	-2.855724812	-4.625926018	1.275744200
H	-3.298717022	-3.858704090	-0.266283125
C	-0.932953417	-4.206474304	-1.592303991
H	-1.103919864	-5.191966534	-2.044440985
H	-1.605698943	-3.489856243	-2.067462683
H	0.093000174	-3.897266865	-1.819704175
C	-0.187513143	4.255054951	1.404089808
C	-0.488929182	3.363239765	2.432363510
C	-0.330516011	1.997767806	2.203268051
N	0.096374288	1.487841606	1.034867883
C	0.384614021	2.356160641	0.049964499
C	0.260893583	3.738685608	0.189941138
H	-0.301291287	5.326254845	1.545489073
H	-0.845258534	3.712338686	3.396481991
H	-0.558245838	1.274380684	2.978354454
H	0.721847177	1.924412847	-0.883341670
H	0.509860098	4.387707233	-0.643886745

==============================

^{3}N1-TS

==============================

Ti	-0.054813389	-0.747807205	0.711194754
P	-2.402491093	0.356838197	0.212333426
P	2.396478653	-1.156732321	0.003208852
N	-0.051697053	-0.376230091	-1.380110383
C	-2.470723391	-1.537724257	-3.988111973
C	-3.708213329	-1.235591650	-3.400556564
C	-1.269402266	-1.252236962	-3.350535631
H	-0.330322683	-1.512666345	-3.828641415
C	-3.687524557	-0.638981462	-2.137608528
C	-1.241244435	-0.636024714	-2.081408501
H	-4.636324406	-0.405574918	-1.659626603
C	-2.490876913	-0.337714970	-1.477867484
H	-2.448925257	-2.019644499	-4.963927269
C	2.164632559	1.270914793	-3.903351307
C	1.013071895	0.811176300	-3.278810978
C	3.445770741	0.996594846	-3.393863678
H	0.042117260	1.058736801	-3.695263386
C	1.076438546	0.036059048	-2.094094515
C	3.514599085	0.245487943	-2.219769478
H	4.493336678	0.026081365	-1.795740843
C	2.369167566	-0.229024082	-1.568799257
H	2.070049524	1.873190522	-4.805264950
C	-5.005155563	-1.573853016	-4.097177982
H	-4.996240139	-1.249271035	-5.144177914
H	-5.190037251	-2.655808449	-4.098434448
H	-5.859807014	-1.096148133	-3.607753277
C	4.685859203	1.493080974	-4.098859310
H	5.573269844	1.397153139	-3.465296030
H	4.878533840	0.930553019	-5.021635056
H	4.591399670	2.548074007	-4.382387638
C	-2.637496948	2.179362059	-0.047208752
H	-3.555660009	2.389107227	-0.605041981
H	-2.671665668	2.689677238	0.920685589
H	-1.781289697	2.564469337	-0.606366813
C	-4.003934860	-0.127384707	0.997155488
H	-4.871964931	0.256549358	0.452552915
H	-4.057608128	-1.217614889	1.041077375
H	-4.031032085	0.266661674	2.017360210
C	3.658141375	-0.271289080	1.030499101
H	3.747363329	-0.780133963	1.995135546
H	4.641648769	-0.243248031	0.551426530
H	3.312706709	0.749220073	1.209127665
C	3.323044062	-2.707294464	-0.417231470
H	3.509618998	-3.281610012	0.495263427
H	2.718141317	-3.316058636	-1.092384934
H	4.277692318	-2.472661257	-0.899066210
C	0.598396182	-1.765663147	2.713329077
H	-0.102946289	-1.765540957	3.554618835
H	1.070068359	-2.747857094	2.665904284
H	1.395139337	-1.042818069	2.946495771
C	-1.136537433	-2.360817194	1.269164205
H	-1.981227279	-2.143754959	1.948412061
C	-1.058951616	-3.864300728	1.075344205
C	-1.166805148	-4.661666393	2.393261671
H	-0.314335376	-4.462278366	3.048782825
H	-2.078820944	-4.389988899	2.937953472
H	-1.198534250	-5.741529465	2.199276686
C	-2.297029495	-4.187510967	0.193246722
H	-2.329502106	-5.261673927	-0.031346150
H	-3.228886604	-3.923855543	0.707386494
H	-2.261015654	-3.633662224	-0.750189483
C	0.205421910	-4.279901028	0.305875123
H	0.190849423	-5.350952148	0.070070572
H	0.277626038	-3.716282129	-0.630444348
H	1.105813146	-4.072961807	0.890096426
C	0.742922664	3.916382313	2.265325546
C	0.476401538	2.898680925	3.185476303
C	0.410545230	1.582739592	2.747798443
N	0.584824741	1.222098470	1.447949171
C	0.857227743	2.220042467	0.562025726
C	0.941027462	3.554054499	0.927230895
H	0.799478829	4.954052925	2.578874111
H	0.321251541	3.118188381	4.237518787
H	0.206951991	0.771910131	3.438677788

H	1.003376484	1.911277175	-0.466428906
H	1.161963105	4.298547745	0.168537781

==============================

P1

==============================

Ti	-0.413210481	-1.028807759	0.762640715
P	-2.838044167	-0.290049762	0.357315302
P	2.012726784	-1.339904904	0.063792154
N	-0.360930473	-0.515298605	-1.314133525
C	-2.650704622	-1.579914451	-4.067347527
C	-3.906724930	-1.556403279	-3.441207409
C	-1.491020918	-1.225291491	-3.394159555
H	-0.536381304	-1.295817137	-3.904286861
C	-3.941647530	-1.158007979	-2.104481459
C	-1.514892817	-0.811835349	-2.041790485
H	-4.903060913	-1.134954453	-1.595482230
C	-2.787178993	-0.788637877	-1.402965426
H	-2.579680204	-1.902454138	-5.104386330
C	1.874622226	1.443085790	-3.593286991
C	0.719023466	0.864138067	-3.081992149
C	3.137892246	1.218057990	-3.022938013
H	-0.239515111	1.098064423	-3.533081055
C	0.762682736	0.012638147	-1.953061342
C	3.191288948	0.370301127	-1.913295150
H	4.156253815	0.171564698	-1.450374365
C	2.042500734	-0.226346970	-1.383713961
H	1.792461038	2.108084679	-4.451319218
C	-5.155058384	-1.980944395	-4.177061081
H	-5.167588234	-1.595600247	-5.202736855
H	-5.228403568	-3.074311733	-4.243990421
H	-6.059164047	-1.623278737	-3.674118996
C	4.378599167	1.858337998	-3.599403381
H	5.239926338	1.733116150	-2.935778141
H	4.645153999	1.420579314	-4.569929123
H	4.237520695	2.933417797	-3.762471676
C	-3.647521973	1.384659171	0.280476093
H	-4.575119495	1.321595311	-0.297427386
H	-3.884629011	1.736272454	1.289483428
H	-2.986149073	2.108202696	-0.197947398
C	-4.218744755	-1.228605151	1.144266725
H	-5.169279099	-1.094199657	0.618631363
H	-3.959247112	-2.285005808	1.183520436
H	-4.335052013	-0.860299289	2.168468237
C	3.453490257	-0.848843873	1.109283328
H	3.420337915	-1.429092646	2.035236359
H	4.407516956	-1.030267119	0.604694963
H	3.379322529	0.207746089	1.367394090
C	2.621443033	-2.937958479	-0.639035285
H	2.753856897	-3.665129423	0.166953430
H	1.874828100	-3.320752621	-1.336906910
H	3.571862698	-2.793544531	-1.162992120
C	0.239371330	-0.714115143	2.815770149
H	-0.677412987	-0.401688248	3.350168705
H	0.589712918	-1.631361961	3.308097839
H	0.996604264	0.057961401	3.002517700
C	-0.932872534	-2.812141657	0.668145001
H	-0.818487942	-2.823089838	1.784476995
C	-1.273658514	-4.183431149	0.130275190
C	-0.224950641	-5.202764988	0.638281047
H	0.778144419	-4.927268028	0.301461160
H	-0.214089975	-5.240806580	1.733816147
H	-0.444580764	-6.211402416	0.264620870
C	-2.669583559	-4.635861874	0.617623270
H	-2.875423908	-5.668075562	0.305929542
H	-2.742624044	-4.590250015	1.710400820
H	-3.450061798	-3.999828339	0.189573094
C	-1.272797108	-4.172748566	-1.410928130
H	-1.442522883	-5.181766033	-1.807636619
H	-2.054464102	-3.515445232	-1.799246430
H	-0.317395121	-3.806870699	-1.798987031
P	0.183695659	1.863567472	0.853773475
C	1.874368548	2.517140150	1.257761717
H	1.866601348	3.606881380	1.374307036
H	2.230062962	2.069654703	2.189437866
H	2.561544180	2.249624491	0.449475676
C	-0.224961728	2.991231918	-0.564223468
H	0.555342913	2.892400742	-1.322690606
H	-1.166586637	2.693624496	-1.031494021
H	-0.290146530	4.037639141	-0.246414393
C	-0.771218002	2.639872551	2.243477106
H	-1.842068553	2.495862722	2.088527679
H	-0.495249182	2.137322187	3.174586773
H	-0.563320041	3.711922884	2.333922148

==============================

^{3}P1-TS

==============================

Ti	-0.216898039	-0.697343171	0.741570830
P	-2.623208523	0.226889193	0.063902795
P	2.166237593	-1.416571617	0.020057186
N	-0.056329072	-0.239728481	-1.367609859
C	-2.162650347	-0.892686486	-4.383365631
C	-3.462611675	-0.678432286	-3.895128727
C	-1.045271516	-0.750686467	-3.573803663
H	-0.057168256	-0.935146153	-3.983703613
C	-3.585748672	-0.332174540	-2.549860001
C	-1.159621477	-0.373928994	-2.214407921
H	-4.583021641	-0.169211641	-2.145275831
C	-2.472815037	-0.182902500	-1.710749984
H	-2.027475119	-1.186645865	-5.422901154
C	2.451430559	1.776914954	-3.280187607
C	1.231818080	1.214740276	-2.921017885
C	3.650805950	1.426619887	-2.639233112
H	0.324859291	1.540167093	-3.420197725
C	1.141546607	0.260852665	-1.880053401
C	3.575348854	0.462862700	-1.629535079
H	4.492503643	0.159139544	-1.128398538
C	2.361069202	-0.122315377	-1.255814314
H	2.472560406	2.525961399	-4.070074081
C	-4.665353298	-0.836853325	-4.795192719
H	-4.577136040	-0.218103677	-5.696607590

H	-4.782840252	-1.875013232	-5.131124496
H	-5.589909554	-0.549293876	-4.284703255
C	4.965407372	2.046258688	-3.051318645
H	5.747136593	1.860056996	-2.307935476
H	5.320582867	1.642355800	-4.008422852
H	4.876734257	3.131781578	-3.176628828
C	-3.342641115	1.942835212	0.042268876
H	-4.243219376	1.980656028	-0.579194307
H	-3.598645210	2.252143621	1.060939074
H	-2.611138582	2.646101236	-0.362253398
C	-4.101315022	-0.738709569	0.619855642
H	-5.003680229	-0.465012252	0.064962588
H	-3.892500401	-1.800722480	0.479501665
H	-4.273071289	-0.554910481	1.684910417
C	3.676561594	-1.313344479	1.082267165
H	3.618148088	-2.106451273	1.833623528
H	4.599525928	-1.444160700	0.508112550
H	3.707058430	-0.353997439	1.601078749
C	2.543583393	-2.950035095	-0.956372082
H	2.606328487	-3.817128897	-0.294385523
H	1.732929587	-3.110112429	-1.671593428
H	3.486697197	-2.832346916	-1.499232769
C	0.337813169	-1.222971797	2.966663837
H	-0.392575800	-1.273382068	3.776921272
H	1.023860455	-2.066910028	3.061127186
H	0.933148682	-0.312902689	3.133485317
C	-1.180181026	-2.245290041	1.607157707
H	-2.077094316	-1.884521961	2.144027948
C	-1.054935217	-3.734970570	1.888907671
C	-1.190226078	-4.094644070	3.385543108
H	-0.350101858	-3.707097292	3.967312098
H	-2.111965895	-3.674370050	3.804939747
H	-1.221928954	-5.182786465	3.524126053
C	-2.246200085	-4.376017570	1.125596046
H	-2.224936008	-5.467922688	1.237432003
H	-3.205596924	-4.017425537	1.516338110
H	-2.200257063	-4.136260509	0.058337037
C	0.246721566	-4.325946331	1.324839473
H	0.286973864	-5.411876678	1.476261377
H	0.314345032	-4.122390270	0.252959937
H	1.123416424	-3.884584665	1.809186697
H	0.690340161	4.089891434	0.364461303
C	0.523443937	3.086700439	-0.042684987
H	1.257128954	2.881319046	-0.824515224
H	-0.467185855	3.039044142	-0.500101089
P	0.669908106	1.794104695	1.270901799
H	0.002071249	3.550531387	2.894546747
C	-0.333473146	2.538377762	2.642569542
H	-1.382732868	2.573424816	2.336116314
H	-0.259318501	1.900913358	3.528498888
H	2.515604973	3.185827494	2.159061670
C	2.389381886	2.136000633	1.872333884
H	2.613560915	1.502568722	2.735568047
H	3.090333462	1.898904800	1.066914320

==============================

X3

==============================

Ti	-0.450638086	-0.174236476	1.149488807
P	-1.187731385	-2.678994417	0.887815535
P	0.072371870	2.161472321	-0.026023520
N	-0.146321744	-0.641530156	-0.812025726
C	-2.351387739	-1.996377468	-3.509610176
C	-3.037838697	-3.015048742	-2.832927465
C	-1.386688828	-1.214940429	-2.881339073
H	-0.884499252	-0.425199807	-3.430612803
C	-2.715348959	-3.226535320	-1.487771153
C	-1.060442686	-1.432777762	-1.529637575
H	-3.232689381	-4.011877537	-0.940560400
C	-1.745326877	-2.461307526	-0.838914216
H	-2.586763620	-1.801992536	-4.554190159
C	2.774039507	-0.038296778	-3.050182104
C	1.701733589	-0.678572357	-2.443626881
C	3.097150564	1.297311902	-2.757042170
H	1.489704967	-1.720755816	-2.660308123
C	0.894380033	0.000363853	-1.513809085
C	2.285965919	1.974205971	-1.843517423
H	2.516377211	3.010846376	-1.607738137
C	1.190632582	1.353295803	-1.230466485
H	3.392854214	-0.591934144	-3.753322363
C	-4.065699100	-3.863586664	-3.543186426
H	-4.740720272	-3.250828981	-4.151118755
H	-3.589885235	-4.585304737	-4.219495296
H	-4.676413536	-4.431243420	-2.834522724
C	4.299278736	1.957530856	-3.389751434
H	4.282480240	3.042605877	-3.250175238
H	5.233248711	1.583721399	-2.951286316
H	4.347051144	1.757344007	-4.465978622
C	-2.585671663	-3.491517544	1.776294231
H	-2.821437359	-4.468188763	1.342325687
H	-2.292581081	-3.631443024	2.820873022
H	-3.474717140	-2.858238697	1.744606972
C	0.001438544	-4.078398705	0.708215058
H	-0.475668401	-4.890949726	0.152272955
H	0.880942643	-3.728812218	0.164209306
H	0.319946110	-4.426168442	1.692315102
C	-0.971505344	3.243818045	-1.105793715
H	-1.633813262	3.857502937	-0.487397552
H	-0.347510248	3.895663261	-1.725284576
H	-1.584753513	2.611749887	-1.752849460
C	1.124076247	3.363449812	0.885834515
H	0.487709194	3.931346178	1.570642591
H	1.842726707	2.790639400	1.474614620
H	1.642741084	4.061144829	0.221829772
C	-3.100510836	0.730172873	0.417111278
H	-4.190097332	0.593940616	0.387724996
H	-2.907809496	1.796193242	0.269711256
H	-2.711488962	0.210764036	-0.465826422
C	-2.498255968	0.220687792	1.747332454
H	-2.970924616	-0.750244379	1.951878786
C	-2.838839769	1.118636370	2.974542141
C	-4.298359871	1.626078963	2.917628527

H	-4.459836006	2.325262547	2.090699673
H	-4.999020100	0.791729391	2.794334888
H	-4.558680058	2.148064852	3.846879244
C	-2.693036795	0.261851847	4.245156765
H	-2.783882856	0.867907405	5.155115604
H	-3.471859217	-0.509871542	4.278294086
H	-1.726916909	-0.243519962	4.275295258
C	-1.907376170	2.345348358	3.061070919
H	-2.170491934	2.979868889	3.916669130
H	-0.859550893	2.048079252	3.164494753
H	-1.990498900	2.962230444	2.157971859
C	0.395780623	-0.802858055	6.127429008
C	0.577718914	-0.324843079	4.828310013
C	0.643514931	-2.144580364	6.421848774
H	0.392433733	0.715919912	4.587317944
H	0.497799605	-2.523255348	7.429399967
C	0.988053083	-1.178112507	3.797333717
C	1.116106510	-3.000245094	5.422575951
H	1.368729949	-4.036012650	5.627455235
C	1.299337745	-2.507195711	4.137742996
H	0.057843745	-0.126315027	6.907207012
C	2.567933559	-2.792115688	2.173341274
C	2.296099663	-1.481344819	1.741216779
C	3.559462786	-3.568421602	1.588843942
H	3.722026587	-4.576235294	1.957986355
C	3.105162621	-0.948517680	0.730136991
C	4.323991776	-3.031696081	0.548305273
H	2.926601410	0.071100600	0.409642667
H	5.096812725	-3.636128902	0.081911042
C	4.101629257	-1.718786836	0.129174784
H	4.698215961	-1.290329933	-0.670998216
O	1.817248821	-3.373927116	3.187672853
C	1.187456965	-0.747268796	2.395409107
O	1.018309116	0.574910402	2.097704887

==============================

X3-TS

==============================

Ti	-0.459462047	-0.049008392	0.974380732
P	-0.721799076	-2.661783457	0.341696471
P	0.016740987	2.385797501	-0.027483510
N	-0.376273125	-0.254418701	-1.104532003
C	-2.387105942	-1.800851226	-3.844177961
C	-2.753184795	-3.033452034	-3.276891708
C	-1.592432737	-0.884124398	-3.164128304
H	-1.330123425	0.064995326	-3.622507334
C	-2.273653984	-3.321073055	-1.996241450
C	-1.125131488	-1.169063926	-1.867815852
H	-2.533373594	-4.272487640	-1.536458373
C	-1.470411062	-2.413038015	-1.299799442
H	-2.746325731	-1.550687313	-4.840493679
C	2.726837873	0.287042975	-3.105320692
C	1.543129921	-0.292955071	-2.664034605
C	3.168995380	1.528357625	-2.622764587
H	1.246135473	-1.270004511	-3.032084942
C	0.738807678	0.355162680	-1.705088139
C	2.348214388	2.190821409	-1.704285622
H	2.662669659	3.161936283	-1.328461885
C	1.148638725	1.634033203	-1.252182245
H	3.338887930	-0.249787614	-3.827668428
C	-3.628812551	-4.003709793	-4.034748077
H	-4.579114914	-3.541229963	-4.327263355
H	-3.142512560	-4.346812248	-4.956326962
H	-3.861304760	-4.888227940	-3.433916330
C	4.499152184	2.101809263	-3.051303864
H	4.553867817	3.177952051	-2.859313250
H	5.329187393	1.631604075	-2.507493973
H	4.682606220	1.940825224	-4.119321346
C	-1.823744893	-3.862356901	1.209802985
H	-1.866475582	-4.823114872	0.686938882
H	-1.435366273	-4.028229237	2.218800783
H	-2.832706690	-3.451660395	1.284600258
C	0.736070931	-3.721787930	-0.064595819
H	0.422507346	-4.549425602	-0.708288550
H	1.487385154	-3.117910385	-0.576948583
H	1.182458520	-4.112814426	0.850708902
C	-0.958650231	3.564856529	-1.071819186
H	-1.620608807	4.165212154	-0.440989286
H	-0.286655635	4.227591991	-1.626094103
H	-1.569956422	3.000302553	-1.778867841
C	1.069660544	3.531274557	0.961290240
H	0.431734741	4.063896179	1.673281550
H	1.788200736	2.930706501	1.520997405
H	1.589327693	4.267488480	0.340093791
C	-1.856686592	-0.556116879	2.679444790
H	-1.531731248	-0.358583808	3.699832678
H	-2.412683487	-1.487228394	2.608566523
H	-0.312989324	-1.033863544	2.414910078
C	-2.296101570	0.572123945	1.881694198
H	-2.088903189	1.543673635	2.342232943
C	-3.656423092	0.594032288	1.155281186
C	-4.727926731	1.065398932	2.165610075
H	-4.770528316	0.387483150	3.025061607
H	-4.498247623	2.069509268	2.540717125
H	-5.723127842	1.096990108	1.703701019
C	-3.605314970	1.581949115	-0.023000399
H	-4.588434219	1.683031797	-0.498993754
H	-3.292294979	2.574697256	0.316838294
H	-2.888398647	1.238877416	-0.775556147
C	-4.073944569	-0.785431325	0.615198314
H	-5.031495094	-0.709665537	0.087232053
H	-3.342170954	-1.180506945	-0.093409181
H	-4.206710815	-1.514152646	1.421865225
C	0.586182654	0.037775841	6.187191010
C	0.677534819	0.279687911	4.815154552
C	0.931651533	-1.215026140	6.700341702
H	0.403007954	1.240237474	4.390316010
H	0.854736209	-1.410709739	7.766139507
C	1.111125231	-0.722997546	3.944993258
C	1.398183465	-2.217546940	5.848594189
H	1.703123927	-3.190752506	6.220127583
C	1.489986181	-1.958733797	4.485177994

H	0.239516154	0.822129369	6.853719234
C	2.608363152	-2.566232920	2.511610508
C	2.234037161	-1.379776359	1.864737153
C	3.611498594	-3.390113831	2.011190176
H	3.863647938	-4.296272278	2.552960873
C	2.930098057	-1.012032986	0.706413925
C	4.267681599	-3.025867701	0.834308088
H	2.667798758	-0.082879409	0.213387355
H	5.047721386	-3.667362213	0.434311122
C	3.931827784	-1.832394361	0.187573969
H	4.445038319	-1.536903620	-0.722767889
O	1.971818924	-2.971329451	3.673055649
C	1.127256989	-0.570959747	2.455571413
O	0.982151866	0.704526901	1.966472387

================================

X4

================================

Ti	-0.676541328	-0.417987138	0.833301246
P	-0.470115751	-2.909554243	0.237191439
P	0.337112278	1.981318355	0.225642204
N	-0.395401478	-0.463012218	-1.214167833
C	-2.689399481	-2.043938160	-3.704676628
C	-2.948549986	-3.285742283	-3.098168373
C	-1.834800601	-1.113473415	-3.124716520
H	-1.667030215	-0.148876756	-3.594090462
C	-2.300040722	-3.571732044	-1.894268394
C	-1.188240767	-1.398541808	-1.908156633
H	-2.481945515	-4.530934334	-1.414828181
C	-1.416198611	-2.659387827	-1.307903409
H	-3.185973883	-1.798039079	-4.640844822
C	2.355413914	0.363953739	-3.599334955
C	1.262505531	-0.288644075	-3.039820671
C	2.886781216	1.536582232	-3.037996531
H	0.888901174	-1.201221704	-3.493214130
C	0.640189350	0.207448661	-1.872383952
C	2.261561155	2.033382893	-1.890600324
H	2.652082443	2.942430496	-1.436552525
C	1.164770484	1.395417094	-1.302380204
H	2.816491842	-0.054156993	-4.492195606
C	-3.882637262	-4.281468868	-3.744945526
H	-4.825865269	-3.809866905	-4.043056488
H	-3.439826012	-4.715246201	-4.650435925
H	-4.121499062	-5.106552124	-3.067059994
C	4.100021839	2.207012892	-3.637447357
H	4.222280502	3.226899862	-3.259902477
H	5.020677090	1.657837272	-3.400819302
H	4.030384541	2.260697365	-4.729773998
C	-1.276164293	-4.328555107	1.094362020
H	-1.309940696	-5.226279259	0.468821734
H	-0.706883132	-4.558401108	2.000044584
H	-2.290048361	-4.046441078	1.386325955
C	1.115091324	-3.658886194	-0.365195125
H	0.930132270	-4.596576214	-0.898978353
H	1.602822065	-2.948431969	-1.036352038
H	1.786034942	-3.841529608	0.479441315
C	-0.293850750	3.644356728	-0.300252348
H	-0.657157838	4.200485706	0.568655968
H	0.503952682	4.216367245	-0.784598351
H	-1.119260788	3.517642021	-1.005200624
C	1.731422067	2.455369711	1.340643167
H	1.316407561	2.851478815	2.271870375
H	2.301647663	1.556432009	1.577463150
H	2.386760712	3.206154346	0.889261901
C	-2.462741375	-1.154714465	1.760452747
H	-2.242305517	-1.702044129	2.678492785
H	-3.141637802	-1.698701382	1.100231767
H	1.142818689	-2.343703985	2.592554569
C	-2.509851456	0.281790704	1.766599059
H	-2.240267038	0.756278276	2.715241909
C	-3.626196146	1.043634653	1.027524471
C	-4.999408722	0.593070388	1.577827454
H	-5.166473389	-0.472940713	1.392457724
H	-5.054561138	0.755048990	2.660250902
H	-5.818187714	1.152322412	1.106733680
C	-3.471653700	2.551078081	1.285082102
H	-4.242434025	3.126405478	0.758043706
H	-3.558977127	2.773735046	2.355294943
H	-2.494711399	2.901724577	0.949640214
C	-3.589084148	0.780299306	-0.491082102
H	-4.429778099	1.271682978	-0.996109068
H	-2.658891678	1.153779149	-0.932728410
H	-3.650283337	-0.289490372	-0.717631519
C	0.872941732	-0.390885264	6.338315964
C	0.711263597	-0.526834011	4.959889412
C	2.069926262	-0.789818704	6.942976952
H	-0.209768400	-0.225913122	4.469254494
H	2.201521397	-0.690835953	8.016581535
C	1.733935952	-1.055950284	4.170220375
C	3.106731892	-1.304733515	6.167734623
H	4.055678844	-1.603397012	6.601435661
C	2.930871487	-1.428691983	4.789649487
H	0.067804493	0.019789517	6.940597057
C	4.049744606	-1.661019325	2.723237038
C	2.906943321	-1.303540111	1.999922633
C	5.299776554	-1.754010558	2.109786034
H	6.157474041	-2.033150434	2.713361025
C	3.051514626	-1.005689740	0.643030643
C	5.416697502	-1.468960881	0.750970721
H	2.178843260	-0.710487664	0.074723750
H	6.389571667	-1.536186337	0.271992773
C	4.292768478	-1.082012296	0.014621269
H	4.373755455	-0.840252042	-1.041090965
O	4.000526428	-1.937677860	4.074644566
C	1.552716494	-1.320766449	2.690810919
O	0.649950504	-0.425796777	2.104904175

================================

X5

================================

Ti	-0.524593771	-0.322160065	1.136593699
P	-1.063068986	-2.720681429	0.339492559

P	0.772666872	1.927181244	0.571128428
N	-0.343854785	-0.339964002	-1.133383870
C	-2.792238235	-1.494341731	-3.729546547
C	-3.297268867	-2.672761440	-3.156676054
C	-1.802813411	-0.740868926	-3.112897635
H	-1.489377499	0.191304594	-3.568738461
C	-2.754179955	-3.060744286	-1.930359125
C	-1.242252231	-1.120344996	-1.866348505
H	-3.136795998	-3.959241867	-1.451436520
C	-1.757689834	-2.316177845	-1.292025208
H	-3.209755182	-1.140005589	-4.670592785
C	2.030088663	0.903095484	-3.743553162
C	1.066492438	0.138007626	-3.106582642
C	2.603761911	2.031908512	-3.135076761
H	0.688082993	-0.747200906	-3.605516911
C	0.581326067	0.455480754	-1.807015300
C	2.169353724	2.329590082	-1.846271157
H	2.626692772	3.173592567	-1.331980705
C	1.195165396	1.575413465	-1.174538493
H	2.364011765	0.606204391	-4.736911774
C	-4.379411221	-3.473709583	-3.840062857
H	-5.164328098	-2.821809053	-4.241510391
H	-3.983696699	-4.056779385	-4.682223797
H	-4.854095459	-4.176251411	-3.147607088
C	3.674154282	2.837451935	-3.832217693
H	3.907508612	3.755516768	-3.283397913
H	4.607473373	2.267589569	-3.930013180
H	3.368337393	3.124351501	-4.845855236
C	-2.243985891	-3.927422285	1.095593572
H	-2.277513266	-4.871433735	0.541684508
H	-1.917895436	-4.130915165	2.119549513
H	-3.247611284	-3.501707315	1.128656745
C	0.328152567	-3.868746281	-0.091568753
H	-0.033645049	-4.641662598	-0.776717365
H	1.134947896	-3.311346292	-0.568467438
H	0.717369080	-4.347953320	0.811469376
C	0.418434739	3.756521225	0.570534110
H	0.507302761	4.134720325	1.592178464
H	1.137689352	4.286473274	-0.060772561
H	-0.591253221	3.972419977	0.215872735
C	2.421625614	1.941204071	1.411988974
H	2.275118828	2.062946320	2.489038706
H	2.905061245	0.980657637	1.236929893
H	3.054973841	2.749426842	1.033666015
C	-1.334729075	-0.965027630	3.055500984
H	-0.640130937	-1.682197690	3.493467808
H	-2.360430956	-1.326399684	3.039077044
H	1.701612353	-2.740273714	1.981918097
C	-1.095205188	0.441010058	3.155112982
H	-0.127529994	0.737466991	3.562178373
C	-2.174878359	1.463560462	3.570170403
C	-1.930586100	1.804015994	5.061344624
H	-1.987711310	0.899728954	5.677169323
H	-0.937568665	2.246588230	5.206074238
H	-2.674385071	2.520227671	5.434496403
C	-2.060819864	2.761114597	2.750488520
H	-2.797105551	3.507424593	3.076823473
H	-1.066553593	3.200521231	2.867625475
H	-2.217801809	2.559209108	1.687279105
C	-3.610549212	0.922993302	3.444025993
H	-4.330504894	1.692883968	3.746580601
H	-3.836866140	0.635806620	2.417049885
H	-3.768142223	0.050524257	4.086650848
C	2.784615278	-0.708911240	5.552044868
C	2.176110744	-0.848176539	4.305220604
C	4.072024822	-1.211725593	5.758623600
H	1.174736023	-0.474995047	4.137669086
H	4.552109241	-1.109735966	6.727949619
C	2.836145163	-1.480791330	3.251100063
C	4.751643181	-1.838235259	4.716802597
H	5.758843899	-2.224311829	4.836804390
C	4.130393505	-1.965366006	3.474972486
H	2.252427101	-0.215560079	6.360321999
C	4.526301384	-2.285805225	1.178538918
C	3.251833439	-1.823085427	0.839126050
C	5.522456169	-2.446456432	0.216110930
H	6.497644424	-2.806181669	0.528735816
C	3.001892090	-1.483115315	-0.490848988
C	5.246669769	-2.119865656	-1.110754609
H	2.022104025	-1.105115652	-0.745983839
H	6.022145748	-2.238084316	-1.862873793
C	3.988793373	-1.624490142	-1.465582728
H	3.768823862	-1.341263771	-2.490496874
O	4.861446381	-2.592146397	2.483347654
C	2.175580025	-1.746271372	1.909279466
O	1.180651307	-0.825124621	1.581561208
C	-2.020562410	3.132793903	-2.436940432
C	-2.191594601	2.247107267	-1.388837934
C	-2.964963436	3.162158251	-3.477603912
H	-1.148931980	3.778403521	-2.464656353
H	-1.477136850	2.164107084	-0.585720897
C	-3.286186934	1.371147513	-1.357130289
H	-2.832484722	3.849431276	-4.308477879
C	-4.066798210	2.314506531	-3.467474461
C	-4.219893932	1.417575479	-2.408998013
C	-3.404389381	0.357452780	-0.323671669
H	-4.798990726	2.314046860	-4.267800331
O	-5.285863400	0.567474723	-2.474955797
C	-4.602361202	-0.464960098	-0.384551048
O	-2.538485527	0.193351641	0.566919506
C	-5.476805687	-0.345875978	-1.480729580
C	-4.890647888	-1.418249965	0.609592617
C	-6.595716476	-1.174644828	-1.605193377
H	-7.237792969	-1.061240911	-2.472063303
C	-6.849084854	-2.115403652	-0.617870927
H	-4.212573051	-1.483593822	1.452890873
C	-6.001904488	-2.234749317	0.499366283
H	-7.716460705	-2.762631416	-0.710534215
H	-6.219898224	-2.967758656	1.269774437

==============================

^{3}X6

==============================

Ti	1.788387299	-1.398158669	-1.337279558
P	-0.787103355	-1.754950047	-1.489246845
P	3.926431656	-0.010435912	-1.725482583
N	1.228180528	-0.296887130	-2.990574598
C	-0.703634143	-1.221137524	-6.065611362
C	-1.756119251	-1.976046801	-5.531160831
C	0.274068475	-0.652831316	-5.252866745
H	1.088239670	-0.090242341	-5.698949337
C	-1.790593863	-2.142768860	-4.142311573
C	0.233990535	-0.802357852	-3.852932930
H	-2.583743572	-2.746887445	-3.708343506
C	-0.828753769	-1.571707726	-3.307870865
H	-0.636797547	-1.089496255	-7.143872261
C	1.661956429	3.250947952	-4.064292431
C	1.107227445	1.990609169	-3.908431530
C	2.937736750	3.569761276	-3.568060160
H	0.107677549	1.796301007	-4.282130241
C	1.800428510	0.953723311	-3.237043142
C	3.622069120	2.559815884	-2.893191099
H	4.603665352	2.784508705	-2.480324268
C	3.083960533	1.277138948	-2.713275194
H	1.081582546	4.021091938	-4.569514275
C	-2.816446781	-2.588057995	-6.415568829
H	-2.412171125	-2.860061646	-7.396740913
H	-3.234838486	-3.493515968	-5.964075565
H	-3.649465322	-1.894202113	-6.590601444
C	3.522014141	4.949404240	-3.757792950
H	4.407957077	5.100568771	-3.133220196
H	3.819148302	5.123435497	-4.800071239
H	2.795812368	5.729272366	-3.499168634
C	-1.749859333	-0.280065656	-0.917699397
H	-2.710483313	-0.235435873	-1.439915776
H	-1.920253277	-0.346265554	0.159606948
H	-1.177854538	0.626810312	-1.125598788
C	-1.938715577	-3.128710747	-1.062064290
H	-2.964238167	-2.901368141	-1.370433331
H	-1.618432760	-4.059257030	-1.529931784
H	-1.914114118	-3.255496264	0.024079187
C	4.635861874	0.898058474	-0.286302030
H	5.207438469	0.199300840	0.331325203
H	5.286403179	1.724697590	-0.586035609
H	3.802104235	1.282765388	0.305728078
C	5.404672146	-0.482255191	-2.727499485
H	6.029178619	-1.179568410	-2.161216021
H	5.063265324	-0.979225814	-3.639203548
H	5.994609356	0.399179786	-2.997797012
H	3.057094336	-4.921925545	-2.215987921
C	0.606648982	3.778010607	0.132595986
C	0.941979647	2.428710222	0.125569090
C	-0.223996267	4.294599533	1.134022474
H	1.568521380	2.019334793	-0.657177567
H	-0.488162220	5.347970963	1.140014291
C	0.451970667	1.558105111	1.124976516
C	-0.731090426	3.451059341	2.128732204
H	-1.389239073	3.818331003	2.909627914
C	-0.400196433	2.103528500	2.116626501
H	0.987988949	4.424658298	-0.652538896
C	-0.782868207	-0.042704862	3.069372416
C	0.066649839	-0.662501574	2.117568970
C	-1.491878986	-0.783906996	4.005658627
H	-2.128677607	-0.257078201	4.709028721
C	0.160876930	-2.072977543	2.147849798
C	-1.377485991	-2.178510666	4.015816212
H	0.796257854	-2.568380594	1.422330856
H	-1.935323954	-2.758570194	4.744713306
C	-0.550801635	-2.816879034	3.082669497
H	-0.460573554	-3.898497581	3.077122688
O	-0.949488878	1.321616530	3.107217312
C	0.736998320	0.155402228	1.152649403
O	1.521708846	-0.375188082	0.228041187
C	1.824801803	-5.284945011	1.732805252
C	2.260083914	-4.768877029	0.515819073
C	0.678752959	-6.089138031	1.769297242
H	2.365520954	-5.059488773	2.646719933
H	3.138581753	-4.130196571	0.469442368
C	1.578660250	-5.038529873	-0.677245617
H	0.328573883	-6.499958992	2.712121010
C	-0.018422863	-6.365168571	0.599071503
C	0.437991887	-5.843554974	-0.615466475
C	2.080760717	-4.462591171	-1.986789346
H	-0.913310587	-6.979097843	0.596455216
O	-0.304753870	-6.177670956	-1.720609903
C	1.125685692	-4.781591892	-3.118567228
C	0.017721556	-5.610429287	-2.934941292
C	-0.851928294	-5.910556793	-3.987960339
C	1.336950541	-4.236364365	-4.390897274
H	-1.701400518	-6.557302952	-3.792638302
C	-0.610684633	-5.372390747	-5.246218204
H	2.171943665	-3.555195808	-4.523160458
C	0.485728681	-4.526710510	-5.450616360
H	-1.288152814	-5.598083973	-6.064902782
H	0.656040132	-4.077378273	-6.423237801
O	2.301909447	-3.076598644	-1.888408065

==============================

X7

==============================

Ti	-0.188318059	-1.454789639	-2.022019148
P	-1.965680718	-2.326558590	-3.667575836
P	2.174409389	-0.462284327	-1.738253355
N	0.196733266	-0.422575951	-3.779899120
C	0.363340467	-1.159164667	-7.440424442
C	-0.678286910	-2.084179163	-7.611703396
C	0.648585916	-0.587076306	-6.206696033
H	1.480666637	0.102711231	-6.114611626
C	-1.424674749	-2.418036461	-6.478190422
C	-0.106376268	-0.914891243	-5.061004162
H	-2.224423647	-3.150242567	-6.572650433
C	-1.158132911	-1.854746103	-5.226590633
H	0.983367860	-0.895658255	-8.295315742
C	1.015962601	3.203293085	-4.244478226

C	0.442665786	1.952751160	-4.463742733
C	1.961866021	3.414013624	-3.233749390
H	-0.309009731	1.827353477	-5.236666679
C	0.812236965	0.849740386	-3.678136587
C	2.346161604	2.310056925	-2.464069843
H	3.077113628	2.449975729	-1.671185851
C	1.798161745	1.047505975	-2.682658434
H	0.697517097	4.043793201	-4.857677460
C	-0.974302590	-2.684778690	-8.965357780
H	-0.052134585	-2.917641640	-9.509484291
H	-1.551642418	-3.610310316	-8.875697136
H	-1.553308129	-1.996260524	-9.594282150
C	2.501467228	4.790002346	-2.929774284
H	3.556214094	4.755682945	-2.635808468
H	2.412172318	5.459824562	-3.790632486
H	1.947784781	5.243882179	-2.097330570
C	-3.599503756	-1.480413795	-3.636881828
H	-4.240378380	-1.803508401	-4.462748528
H	-4.079415798	-1.688341498	-2.676947832
H	-3.427693129	-0.403562337	-3.702522278
C	-2.398434639	-4.110979080	-3.824719667
H	-3.061160803	-4.306643009	-4.673324585
H	-1.484241962	-4.698256016	-3.937374830
H	-2.899400949	-4.424371243	-2.904316425
C	3.169567347	0.045854401	-0.277861387
H	3.445908785	-0.860050201	0.268564850
H	4.081386566	0.564655006	-0.591677248
H	2.585137606	0.684791148	0.383734047
C	3.442691326	-1.304122567	-2.789287806
H	3.825665712	-2.181649208	-2.263278484
H	2.976509809	-1.632210016	-3.720582724
H	4.266293049	-0.617384315	-3.007875681
H	0.734305739	-5.108141899	-1.471896768
C	-1.152591467	3.200703859	-1.378916264
C	-1.352257371	1.821288109	-1.340000629
C	-0.356949359	3.820309401	-0.413851500
H	-1.962908983	1.323382974	-2.085991383
H	-0.198598295	4.894751072	-0.439181536
C	-0.748849630	1.032618523	-0.352161169
C	0.234130725	3.058004856	0.596828222
H	0.844233453	3.509172916	1.373112559
C	0.031857502	1.683897495	0.618679941
H	-1.609207988	3.788812876	-2.168938398
C	0.186964601	-0.282288581	1.940984249
C	-0.602089703	-1.012355208	1.036423087
C	0.535029650	-0.793679833	3.186627865
H	1.151820540	-0.190139115	3.845261574
C	-1.093148232	-2.256849051	1.452183008
C	0.068578973	-2.055533171	3.564362764
H	-1.715517044	-2.811231852	0.757839382
H	0.339776158	-2.460508585	4.535087109
C	-0.758166254	-2.779515743	2.701565742
H	-1.133118749	-3.755080700	2.997487068
O	0.664174795	0.978530169	1.630444407
C	-0.875204802	-0.440330833	-0.296559125
O	-1.808877110	-1.032737494	-1.083267212
C	2.007582903	-4.205006599	-5.473563671
C	1.388402224	-4.016223907	-4.239081383
C	3.269239902	-4.805818081	-5.520130634
H	1.508125067	-3.874418259	-6.378962994
H	0.426041931	-3.529999018	-4.196459770
C	1.999915361	-4.397360802	-3.044647455
H	3.769564390	-4.960414410	-6.472019672
C	3.904475689	-5.190643311	-4.341266632
C	3.269984007	-4.978913307	-3.116765499
C	1.331895709	-4.206097126	-1.690339446
H	4.896360397	-5.631167412	-4.341850758
O	3.975427866	-5.339858055	-1.988629699
C	2.407168388	-4.082743645	-0.619766951
C	3.654936552	-4.685266972	-0.817112684
C	4.650845528	-4.636102200	0.159355611
C	2.179028511	-3.413964033	0.584922135
H	5.606874943	-5.105912685	-0.048124257
C	4.399025440	-3.973937511	1.358481050
H	1.222903967	-2.933179617	0.738963842
C	3.162502766	-3.356955767	1.571903586
H	5.172672272	-3.932338476	2.120252609
H	2.953476667	-2.833546162	2.499833584
O	0.445584804	-3.120342731	-1.664770842

Table S5. Vibrational frequencies (in cm^{-1}) of the optimized structures

================================

thioxanthone

================================

39.46 107.54 137.48 202.66 228.48 231.76
338.62 340.30 413.45 415.82 451.68 452.78
484.77 495.18 505.34 594.84 641.90 679.37
680.73 726.01 740.33 756.23 777.51 784.62
826.77 884.60 888.69 944.48 981.94 983.59
1010.22 1011.30 1063.99 1065.11 1094.76 1095.51
1144.07 1155.41 1193.78 1202.15 1203.14 1261.87
1296.21 1334.81 1361.73 1366.71 1474.41 1477.44
1505.25 1512.57 1607.00 1621.79 1650.57 1651.46
1722.06 3181.33 3181.40 3194.23 3194.30 3206.68
3206.84 3223.75 3224.18

================================

$H_2C=CH^tBu$

================================

106.86 245.37 283.78 309.70 313.02 331.04
362.55 395.77 418.20 535.04 698.66 723.36
895.05 936.58 939.80 949.63 973.64 1028.21
1047.13 1058.69 1100.27 1234.65 1241.04 1301.87
1351.93 1413.95 1415.87 1440.52 1467.30 1496.05
1501.53 1506.46 1520.93 1521.56 1540.35 1723.10
3031.36 3034.02 3041.57 3100.28 3104.16 3110.90
3111.70 3115.54 3116.04 3120.03 3160.55 3240.75

================================

xanthone

================================

64.52 122.28 152.23 233.95 246.12 292.24
328.95 387.90 420.73 446.32 463.85 529.44
529.67 551.24 601.27 641.82 666.52 682.88
713.85 730.66 775.31 782.57 819.75 850.26
883.00 889.94 899.37 948.05 978.79 980.63
1002.65 1003.55 1056.36 1060.97 1131.21 1139.77
1182.78 1186.73 1209.25 1248.99 1251.33 1274.21
1307.61 1357.95 1382.42 1387.08 1503.53 1503.96
1513.94 1526.23 1621.15 1643.81 1662.72 1676.02
1750.57 3189.53 3189.59 3203.91 3203.98 3216.98
3217.36 3222.00 3222.41

================================

$^tBuHC=C_{13}H_8S$

================================

49.47 55.47 70.38 85.09 136.20 147.16
172.34 207.00 221.74 249.92 267.80 276.23
307.54 311.68 339.02 355.70 382.84 396.87
422.36 432.50 453.76 455.52 488.10 504.08
534.31 541.16 598.07 643.20 659.71 685.53
701.22 734.11 755.99 761.07 770.87 779.44
800.11 880.72 883.75 902.48 914.18 930.92
946.52 956.02 960.70 973.03 985.05 990.42
991.15 1055.22 1056.95 1061.83 1066.11 1083.27
1085.54 1148.77 1157.42 1195.46 1197.96 1199.55
1220.37 1233.38 1262.57 1286.98 1299.39 1321.98
1331.73 1340.60 1412.46 1419.16 1420.77 1450.54
1477.51 1478.14 1494.64 1498.58 1499.29 1504.22
1508.78 1517.40 1524.20 1537.28 1611.36 1620.04
1639.64 1649.74 1697.14 3033.17 3037.02 3049.57
3102.68 3107.53 3111.91 3117.55 3122.83 3133.01
3163.12 3179.84 3182.48 3188.41 3193.29 3197.94
3204.50 3208.43 3213.40

================================

$^tBuHC=C_{13}H_8O$

================================

56.14 67.16 79.41 94.13 140.90 156.83
201.42 208.90 238.75 255.23 263.22 275.56
316.36 331.80 366.47 378.17 402.55 411.46
442.17 467.03 484.16 506.69 531.77 545.10
565.15 607.08 620.62 647.05 675.94 714.58
731.00 757.45 767.04 771.01 778.57 838.14
870.25 874.93 879.28 888.47 917.86 928.18
944.13 951.05 954.58 971.47 980.80 982.30
985.62 1056.99 1063.67 1063.97 1070.44 1126.51
1136.14 1182.11 1190.49 1199.32 1217.10 1232.97
1240.54 1248.07 1282.92 1294.08 1307.75 1328.61
1352.86 1361.28 1413.55 1418.88 1426.83 1451.53
1493.97 1494.45 1498.57 1498.82 1504.45 1515.75
1517.31 1524.09 1528.59 1538.35 1621.65 1635.90
1653.55 1674.69 1690.11 3032.39 3037.77 3048.56
3102.29 3106.99 3109.70 3116.35 3129.38 3141.48
3165.88 3182.87 3186.49 3193.41 3202.07 3205.60
3216.02 3216.36 3238.87

================================

1

================================

25.14 27.16 36.54 40.80 42.65 51.43
53.88 64.24 71.26 85.99 93.04 106.81
108.31 123.08 128.99 139.75 143.58 147.33
155.17 162.19 163.68 182.84 190.32 194.92
201.85 207.49 211.90 225.65 239.88 245.51
247.23 249.93 265.54 273.30 286.37 297.61
302.89 318.92 326.81 340.80 349.08 369.06
371.13 377.98 407.47 413.10 448.32 454.20
462.78 468.33 470.35 484.32 489.54 518.25
532.68 541.19 552.24 565.78 600.70 629.18
656.04 674.61 680.42 703.33 710.68 731.29
731.64 735.29 749.13 791.82 832.25 834.11
846.51 847.76 848.56 870.19 887.26 892.05
893.04 902.62 903.73 923.09 928.63 929.91
931.66 935.31 966.59 967.11 969.03 975.36
976.26 979.50 1022.36 1026.16 1047.26 1060.69
1063.44 1064.52 1081.17 1090.10 1169.39 1178.46
1182.99 1186.46 1221.44 1230.98 1242.77 1245.40
1272.46 1296.34 1312.85 1317.88 1318.83 1325.01
1326.41 1327.68 1333.97 1341.98 1343.87 1402.47
1407.78 1427.36 1428.20 1436.72 1437.93 1440.53
1444.65 1452.75 1466.78 1468.41 1468.58 1469.39
1478.85 1480.58 1481.72 1483.56 1490.10 1495.47
1497.29 1498.07 1502.14 1507.18 1509.68 1511.26

1513.07 1514.53 1520.11 1529.53 1593.25 1601.90
1652.74 1658.59 2834.11 2974.17 3018.84 3022.46
3028.51 3029.51 3032.06 3043.11 3044.58 3045.16
3048.16 3052.38 3073.99 3082.00 3087.88 3088.36
3092.57 3101.22 3105.48 3112.99 3113.97 3114.26
3122.11 3131.02 3133.10 3134.86 3136.22 3145.50
3148.56 3155.09 3157.16 3158.84 3162.19 3166.70
3169.49 3200.89 3204.02

==============================

T1

==============================

14.63 15.96 21.81 26.52 28.47 39.92
41.46 48.05 52.69 58.09 61.67 65.87
70.75 77.18 91.04 95.81 106.00 112.61
115.52 122.25 133.22 142.63 152.58 154.02
160.47 166.39 167.15 172.51 178.43 193.49
203.13 207.45 209.31 214.38 219.53 222.73
232.16 234.55 239.27 246.27 252.23 253.68
256.85 270.00 275.96 283.85 287.01 305.79
309.64 318.85 325.74 339.56 341.68 343.76
356.25 369.12 375.55 383.19 407.30 415.02
417.72 418.77 430.04 447.78 455.23 457.99
459.30 471.15 480.36 483.68 487.62 495.12
497.79 504.75 528.98 531.16 541.69 553.17
568.41 599.84 627.34 640.29 641.04 645.70
673.96 680.89 681.93 682.99 702.69 711.32
723.63 727.27 728.62 733.01 743.10 750.22
756.17 779.12 788.62 800.93 826.00 831.11
832.74 847.88 849.25 850.16 865.99 881.88
885.16 885.68 890.26 894.88 900.61 906.85
918.25 928.56 933.58 935.94 936.94 943.94
967.76 971.28 976.21 977.99 979.38 983.55
984.70 991.62 1006.74 1012.77 1021.05 1024.74
1048.48 1061.81 1064.45 1065.22 1067.72 1069.64
1081.12 1089.11 1095.59 1098.44 1149.06 1161.30
1173.87 1181.92 1188.06 1189.96 1195.83 1202.71
1204.87 1222.69 1232.69 1241.93 1247.39 1267.15
1283.00 1294.62 1301.14 1317.11 1318.03 1320.33
1325.05 1325.79 1332.18 1337.18 1339.46 1340.53
1341.42 1358.50 1364.72 1401.00 1407.80 1426.44
1428.41 1436.23 1437.35 1444.68 1456.14 1464.28
1468.68 1470.48 1471.27 1472.52 1474.03 1476.77
1479.64 1481.18 1484.60 1486.68 1489.86 1495.82
1498.01 1499.27 1500.12 1504.43 1507.89 1509.38
1510.15 1513.26 1513.69 1516.68 1522.71 1531.56
1585.98 1597.01 1602.20 1615.15 1644.41 1649.10
1651.61 1658.94 1680.34 2841.81 2968.29 3016.31
3020.15 3022.88 3029.59 3032.76 3042.38 3047.98
3049.65 3050.06 3051.04 3072.89 3084.54 3086.93
3089.38 3099.35 3101.56 3106.74 3109.39 3111.27
3113.96 3126.15 3130.33 3137.64 3138.80 3149.35
3150.84 3154.26 3155.13 3157.06 3158.62 3161.58
3161.79 3165.67 3179.36 3180.83 3194.44 3196.05
3204.59 3208.10 3209.01 3219.49 3266.19 3275.30

==============================

^{3}T1-TS

==============================

-224.08 22.90 26.03 30.05 33.48 40.58
44.40 48.85 51.80 58.56 63.77 64.22
77.35 81.07 89.64 97.84 103.76 111.47
118.89 121.26 127.88 139.37 142.61 150.53
157.26 158.26 171.42 176.48 187.79 192.60
195.79 204.44 206.86 213.18 227.53 233.05
240.04 243.01 246.26 248.11 253.98 258.72
263.78 269.03 275.27 288.68 293.79 300.20
306.76 314.11 321.26 328.60 332.86 346.92
351.19 363.66 374.37 393.35 400.41 403.63
404.88 410.24 421.82 446.86 451.57 454.93
461.18 469.53 471.12 474.25 483.69 487.06
513.24 519.98 528.71 550.71 564.66 604.67
606.03 620.38 622.98 639.96 651.57 666.83
669.71 678.22 696.77 700.50 713.32 717.64
720.25 724.64 729.89 732.22 735.12 748.84
761.18 768.89 769.73 790.59 818.22 822.32
831.37 846.64 849.40 850.80 865.70 866.20
869.60 871.66 894.48 895.80 903.11 907.19
912.12 913.44 921.40 934.45 936.47 937.88
939.59 942.22 955.43 970.31 976.00 977.77
978.21 979.07 982.45 989.12 1022.41 1024.07
1036.83 1041.45 1044.59 1058.80 1063.65 1064.94
1077.42 1077.90 1079.57 1087.57 1144.22 1148.07
1150.12 1176.22 1179.80 1182.18 1188.95 1194.10
1198.60 1214.85 1222.21 1238.86 1241.57 1270.24
1271.55 1286.04 1292.46 1295.85 1307.43 1315.49
1320.98 1327.59 1328.46 1333.06 1336.04 1337.50
1343.48 1345.02 1345.56 1379.61 1406.78 1412.89
1426.38 1427.94 1434.25 1439.60 1441.98 1455.08
1461.32 1465.36 1470.29 1471.16 1472.08 1473.63
1478.43 1482.96 1483.65 1484.35 1487.17 1487.84
1496.27 1496.62 1500.17 1502.53 1503.73 1506.74
1507.25 1508.75 1510.18 1518.09 1520.55 1521.99
1535.84 1582.11 1587.78 1597.42 1599.33 1606.17
1629.57 1650.20 1659.45 3021.85 3026.56 3027.20
3028.82 3029.71 3035.26 3044.39 3047.33 3048.01
3048.46 3053.56 3079.84 3087.41 3090.03 3096.54
3107.43 3111.97 3113.83 3115.75 3130.52 3131.23
3133.89 3134.35 3137.73 3137.84 3144.73 3147.30
3149.97 3153.95 3154.94 3159.85 3160.90 3163.86
3164.20 3166.33 3174.60 3175.31 3184.39 3186.34
3197.27 3202.71 3203.13 3209.64 3233.16 3263.19

==============================

^{3}T2

==============================

13.16 18.95 23.82 36.24 39.73 41.97
44.70 46.24 48.52 49.51 58.34 68.70
79.75 86.26 90.90 98.94 106.51 112.96
113.49 119.63 130.50 138.17 148.05 148.70
156.49 161.92 165.40 174.87 183.23 195.48
197.78 211.01 215.89 223.77 228.97 233.58
241.74 243.18 247.59 248.65 253.30 268.31
273.35 284.14 285.16 297.00 301.68 306.21
314.53 326.12 333.24 339.76 344.79 352.42

369.06 375.86 383.06 397.66 404.06 412.13
415.09 419.96 438.91 452.78 455.97 468.25
474.75 475.82 483.51 485.66 497.50 514.20
516.07 530.37 541.89 551.84 563.59 606.02
628.85 632.63 665.05 676.53 678.98 688.92
705.18 710.21 718.50 725.82 727.47 730.42
732.72 734.91 739.51 748.51 763.93 768.87
813.63 832.80 838.70 846.42 847.03 848.78
867.21 868.89 871.98 876.38 889.86 894.77
895.39 902.48 905.72 913.36 923.04 931.47
933.15 937.68 938.75 940.59 941.38 943.23
966.91 968.63 977.32 979.53 980.08 982.08
990.60 1013.88 1022.44 1022.98 1025.90 1049.46
1062.56 1064.27 1064.65 1067.61 1081.29 1085.93
1087.39 1089.26 1103.04 1135.86 1149.77 1155.72
1183.36 1190.28 1191.55 1195.68 1202.88 1230.06
1237.81 1244.36 1245.62 1246.91 1263.12 1274.69
1289.26 1295.50 1313.12 1320.19 1321.51 1328.24
1330.19 1333.54 1337.22 1339.28 1343.07 1346.28
1347.06 1348.46 1374.30 1408.49 1411.37 1417.85
1427.62 1428.79 1435.65 1442.65 1443.89 1460.32
1466.47 1468.75 1469.59 1470.85 1475.79 1479.39
1479.88 1482.74 1482.96 1487.43 1494.69 1497.67
1498.41 1500.20 1501.30 1507.76 1508.97 1509.65
1510.35 1511.11 1516.46 1521.14 1523.13 1525.74
1535.25 1589.55 1590.21 1599.63 1603.80 1613.86
1634.48 1654.07 1659.60 2926.24 2993.83 3021.04
3023.14 3027.85 3030.44 3030.76 3046.80 3047.43
3047.80 3051.52 3057.25 3085.02 3085.58 3085.63
3087.89 3094.88 3096.27 3100.06 3106.43 3109.46
3114.11 3114.18 3132.46 3133.96 3135.68 3137.94
3153.29 3153.91 3158.54 3159.59 3161.58 3166.23
3167.52 3170.37 3175.50 3175.62 3186.53 3194.69
3200.19 3203.83 3204.54 3211.74 3244.65 3263.38

==============================

B

==============================

25.91 31.44 43.14 45.75 48.58 56.67
65.59 76.11 82.44 91.49 96.29 105.00
117.83 120.57 134.07 140.37 148.43 151.42
153.32 163.44 168.72 187.31 198.11 208.67
210.96 216.34 230.74 237.13 239.45 248.92
258.71 262.95 270.04 276.71 289.23 321.63
327.52 329.42 338.55 343.61 346.34 351.62
373.19 395.34 402.84 412.36 416.41 443.48
453.29 462.20 463.73 470.10 483.20 523.17
531.40 550.80 559.74 566.57 625.94 673.85
677.36 699.18 705.73 716.37 726.55 731.63
732.43 735.63 746.63 754.75 826.68 833.17
846.25 848.39 850.69 867.65 878.66 887.52
891.32 901.09 907.37 913.18 926.66 930.04
936.28 937.84 947.54 965.41 971.59 974.10
976.90 979.49 998.69 1022.07 1024.35 1026.96
1063.83 1064.78 1076.05 1085.40 1109.00 1113.65
1176.24 1183.99 1224.44 1239.81 1241.75 1250.37
1264.82 1288.98 1296.18 1316.43 1319.15 1322.72
1324.93 1326.16 1326.58 1337.56 1340.34 1342.17
1409.36 1412.52 1426.74 1427.12 1427.86 1436.25
1444.28 1447.28 1463.13 1467.51 1469.78 1470.41
1479.09 1480.20 1483.29 1484.36 1495.65 1496.81
1498.20 1501.62 1502.70 1507.00 1508.66 1510.25
1517.68 1520.15 1522.13 1532.46 1592.49 1604.22
1650.46 1656.44 2913.82 2993.17 3014.15 3018.64
3023.08 3026.49 3028.88 3029.12 3045.58 3046.32
3046.59 3052.41 3082.24 3082.44 3086.66 3087.80
3091.03 3096.23 3099.04 3103.21 3113.96 3114.39
3114.50 3127.31 3133.16 3133.34 3139.29 3149.14
3153.08 3161.01 3162.93 3164.43 3165.27 3167.27
3167.48 3198.12 3200.56

==============================

B1

==============================

21.19 27.09 30.66 37.72 40.22 53.88
56.67 59.47 60.84 61.79 66.05 76.60
78.74 80.02 86.55 99.12 103.31 114.81
119.75 127.54 130.52 132.31 143.08 150.93
156.54 161.91 172.75 179.39 184.44 194.36
199.52 204.64 211.35 219.40 220.63 232.14
232.30 240.43 243.88 251.60 252.18 258.45
264.19 273.81 281.57 287.81 292.16 305.15
312.14 328.03 335.57 339.00 341.56 350.14
354.60 364.91 376.71 385.68 402.65 408.82
417.24 421.45 423.45 442.50 457.78 458.39
460.22 478.26 479.78 482.50 482.81 485.72
497.54 505.72 510.46 528.81 541.18 552.12
563.57 600.55 625.80 640.13 679.49 681.38
684.35 685.32 706.06 714.07 725.13 726.28
727.81 731.17 737.25 742.19 749.26 762.18
763.08 782.02 783.54 788.94 822.05 827.98
833.22 848.33 848.51 853.27 864.22 884.52
887.29 892.16 896.94 897.62 900.49 904.07
918.90 925.03 936.39 940.28 940.98 943.51
956.03 969.43 971.29 980.44 984.01 984.94
987.69 989.41 1012.71 1016.73 1021.35 1023.99
1036.30 1042.28 1063.04 1064.15 1066.62 1068.81
1080.11 1085.60 1094.13 1096.48 1117.33 1138.00
1150.56 1165.63 1187.84 1191.59 1194.91 1202.21
1206.22 1232.92 1240.35 1245.78 1250.46 1268.02
1270.76 1292.66 1293.28 1307.61 1316.75 1318.86
1326.05 1328.36 1337.63 1339.79 1340.38 1342.93
1344.27 1345.93 1356.41 1362.60 1408.69 1412.55
1421.57 1425.85 1428.25 1435.85 1444.11 1444.89
1464.80 1469.06 1470.75 1472.04 1472.81 1474.06
1475.68 1480.95 1485.55 1486.89 1494.06 1496.49
1497.00 1497.54 1504.66 1504.94 1507.03 1508.68
1510.12 1516.63 1519.03 1519.78 1521.71 1532.94
1578.67 1590.11 1600.38 1613.70 1643.89 1647.49
1654.31 1661.56 1682.07 2936.53 2937.79 2989.44
3015.81 3021.38 3023.27 3026.25 3031.35 3049.88
3052.40 3053.11 3055.98 3072.55 3079.84 3083.23
3086.82 3094.69 3098.50 3107.08 3109.84 3110.74
3116.34 3134.89 3138.25 3139.15 3144.88 3146.83

3155.15 3155.22 3155.43 3155.66 3158.62 3161.56
3167.34 3168.95 3176.68 3179.28 3180.93 3193.61
3195.82 3204.12 3206.67 3210.06 3217.53 3261.72

==============================

^{3}B1-TS

==============================

-297.28 1.01 22.66 27.12 37.09 42.04
52.80 53.50 60.02 63.40 71.27 76.11
79.56 81.88 84.43 93.53 106.48 112.33
117.80 121.88 131.45 143.48 152.41 158.85
163.03 165.63 171.00 178.18 183.73 190.79
198.95 207.95 213.43 227.46 229.63 230.16
232.69 236.87 244.15 252.51 254.40 255.82
269.80 277.67 286.68 294.12 301.72 319.19
322.46 324.68 336.34 345.31 345.80 352.21
364.41 374.18 375.32 400.11 405.31 412.89
416.29 430.95 440.88 448.37 451.85 459.81
461.26 466.40 471.65 481.74 486.44 495.04
506.22 511.97 517.41 532.14 548.99 565.16
600.28 620.42 626.50 638.80 659.66 672.31
675.90 677.14 699.97 706.62 710.75 714.48
723.91 725.45 728.93 729.20 737.01 746.18
752.51 757.73 765.11 800.84 818.42 824.13
828.53 845.75 847.17 848.45 861.82 862.93
864.10 876.56 891.30 892.77 902.80 904.30
915.09 917.53 924.02 933.07 934.17 936.99
937.18 939.91 943.59 959.22 968.55 971.10
973.77 975.63 976.90 977.83 1009.75 1023.03
1026.96 1031.03 1048.81 1057.93 1064.33 1064.55
1077.01 1080.36 1083.77 1091.21 1096.97 1110.22
1143.89 1146.60 1179.81 1185.80 1187.28 1192.92
1197.91 1223.66 1233.81 1238.56 1247.31 1267.15
1272.13 1289.54 1291.09 1296.60 1314.00 1319.96
1321.22 1325.30 1326.68 1335.78 1337.83 1340.30
1342.58 1346.52 1351.61 1375.75 1384.91 1409.93
1412.78 1423.12 1427.95 1437.54 1439.34 1445.77
1459.82 1462.84 1467.09 1470.03 1471.89 1473.05
1474.46 1479.54 1479.93 1482.26 1485.26 1488.35
1496.57 1498.67 1498.80 1499.00 1500.80 1506.82
1507.37 1508.62 1509.77 1518.06 1519.41 1522.34
1532.16 1585.66 1592.74 1600.38 1602.10 1609.82
1632.39 1651.80 1656.52 3017.69 3021.52 3025.74
3028.41 3030.91 3031.49 3039.01 3050.21 3051.99
3053.31 3056.17 3076.75 3085.18 3089.00 3091.03
3094.37 3103.43 3104.71 3113.98 3114.20 3125.73
3130.49 3137.37 3139.82 3140.41 3141.06 3151.00
3155.27 3155.66 3159.86 3164.05 3166.43 3174.27
3176.72 3176.87 3180.59 3182.04 3184.15 3191.27
3197.64 3200.25 3201.33 3203.72 3231.31 3240.51

==============================

^{3}B2

==============================

16.12 18.33 22.02 26.23 29.60 35.24
37.87 39.03 41.49 45.81 46.82 54.20
61.57 71.87 84.32 92.86 100.16 101.71
110.02 112.57 118.41 126.27 141.90 145.64
149.88 160.07 164.90 174.29 182.88 189.68
196.51 198.60 208.75 214.17 219.06 226.09
228.60 240.71 244.16 249.85 254.85 260.10
271.18 280.29 288.80 295.40 299.49 319.79
327.22 335.40 343.62 346.52 349.17 353.97
367.73 378.26 394.26 411.98 414.92 418.20
428.11 437.08 451.69 452.25 470.36 471.16
476.49 478.76 483.86 486.02 504.85 517.31
527.79 552.56 562.25 564.81 593.92 606.03
629.04 632.34 666.51 676.13 678.32 689.29
705.55 710.38 718.14 724.70 730.50 730.73
731.89 739.53 742.41 749.25 764.16 770.50
815.45 830.15 831.30 840.60 847.39 848.56
850.02 866.05 868.96 869.11 891.84 894.49
895.22 898.04 902.76 905.03 921.32 932.24
938.27 939.45 939.83 941.55 942.05 948.32
969.67 972.25 979.80 981.24 983.81 984.10
987.25 1017.09 1022.67 1025.57 1032.44 1043.22
1060.84 1063.50 1064.82 1064.89 1080.73 1082.72
1085.96 1088.23 1095.62 1136.37 1150.36 1157.03
1184.04 1189.73 1193.73 1194.80 1200.87 1228.13
1238.67 1243.95 1245.50 1259.83 1269.70 1274.75
1291.43 1294.33 1309.90 1319.00 1321.12 1330.11
1330.87 1333.24 1337.16 1342.34 1345.88 1347.51
1357.16 1366.15 1375.98 1380.86 1413.01 1414.13
1427.22 1428.68 1434.80 1441.85 1442.22 1457.32
1458.88 1467.19 1467.87 1468.86 1470.64 1474.95
1478.38 1480.89 1481.67 1483.69 1487.65 1494.50
1495.84 1497.10 1499.75 1501.60 1503.56 1507.59
1509.04 1510.35 1511.27 1519.95 1523.81 1524.27
1534.99 1586.31 1587.98 1597.64 1601.82 1610.73
1632.57 1654.68 1659.93 2967.29 2981.74 3002.83
3017.93 3024.26 3028.29 3029.56 3031.56 3034.64
3046.14 3048.09 3049.14 3050.84 3083.77 3085.75
3086.13 3089.14 3091.20 3096.17 3108.70 3114.45
3114.62 3114.93 3131.73 3132.93 3134.56 3137.78
3147.44 3150.88 3152.40 3154.69 3157.54 3161.87
3165.29 3167.20 3173.78 3174.00 3186.76 3188.34
3201.84 3203.86 3204.04 3209.52 3262.14 3262.49

==============================

T3

==============================

17.58 30.35 35.21 36.44 38.82 49.38
53.97 55.46 57.37 65.40 74.09 79.75
86.17 88.69 92.45 108.44 110.61 117.28
127.15 137.21 143.15 148.61 149.37 151.18
163.26 171.92 175.98 192.06 196.74 203.55
214.16 216.65 222.13 229.13 231.55 240.35
247.11 252.17 257.93 263.30 267.30 277.87
282.00 286.02 297.05 302.50 311.06 316.73
324.28 327.21 330.96 341.09 347.70 358.39
377.07 382.91 395.70 410.09 411.46 419.00
432.37 440.14 452.14 456.06 460.86 462.36
465.59 473.60 495.42 501.76 525.11 532.89
539.41 540.46 552.65 565.11 593.19 606.88

629.93	632.45	664.58	672.29	680.01	698.49
704.97	707.91	721.78	731.03	733.14	734.96
737.59	739.63	748.50	756.19	766.65	770.67
788.88	826.23	833.32	846.21	851.02	854.26
871.70	876.56	881.93	887.30	890.54	895.65
898.29	904.38	906.45	911.59	920.52	926.19
933.78	934.38	937.99	943.41	951.18	952.76
960.13	966.53	970.99	979.19	983.32	987.81
990.63	1008.63	1022.78	1024.92	1028.81	1048.85
1057.12	1063.46	1063.60	1064.06	1073.31	1081.35
1081.71	1091.64	1105.79	1134.43	1137.09	1145.89
1174.41	1175.33	1181.11	1193.17	1195.04	1231.90
1235.03	1242.82	1244.54	1247.07	1264.40	1265.88
1280.49	1297.49	1308.00	1313.14	1319.71	1320.88
1325.09	1325.51	1327.80	1335.29	1339.00	1340.50
1340.76	1342.86	1352.84	1410.28	1414.72	1423.52
1427.99	1428.43	1437.57	1445.33	1446.12	1468.61
1470.95	1471.27	1474.68	1475.89	1477.98	1478.91
1481.29	1485.54	1486.59	1494.42	1495.37	1498.32
1498.63	1498.78	1500.77	1505.39	1508.55	1510.15
1510.76	1511.81	1516.34	1519.27	1523.22	1527.09
1537.65	1598.58	1607.09	1609.23	1620.60	1631.36
1645.06	1650.16	1656.00	2995.38	3017.39	3024.46
3028.16	3030.81	3032.64	3035.90	3045.33	3051.26
3052.01	3063.15	3075.47	3085.13	3087.55	3088.76
3089.92	3095.48	3107.79	3115.01	3117.37	3119.48
3132.15	3133.32	3136.13	3136.89	3145.70	3153.66
3155.57	3162.47	3165.20	3166.41	3166.79	3168.45
3169.96	3175.06	3175.92	3182.97	3185.95	3186.30
3199.60	3200.20	3200.88	3201.06	3222.73	3224.15

==============================

T3-TS

==============================

-1357.29	8.00	21.09	27.02	36.26	37.32
44.79	56.26	59.76	63.98	66.13	71.76
75.84	85.77	92.49	99.00	105.23	117.71
122.11	135.03	138.89	146.55	152.38	155.01
159.25	162.01	173.47	179.05	193.14	201.49
202.42	222.43	224.83	235.56	238.16	241.75
249.16	255.81	261.18	263.12	269.87	276.79
285.61	295.39	301.25	311.57	316.32	319.92
320.53	324.83	331.73	347.48	350.72	376.80
388.64	396.86	410.49	414.38	416.74	430.00
436.03	447.65	449.42	455.97	458.91	460.75
470.68	491.64	495.96	519.67	535.77	538.19
541.18	549.77	560.43	563.01	585.83	616.43
627.97	633.46	671.84	675.52	677.77	703.71
709.03	713.63	720.54	729.56	731.80	734.82
735.13	737.49	748.21	756.11	768.17	771.19
787.45	820.24	822.44	832.71	847.52	850.09
850.88	868.25	871.40	884.30	890.46	890.81
893.05	894.70	904.82	905.83	907.88	922.40
925.40	933.13	934.95	939.86	947.82	954.45
956.98	961.04	964.88	971.28	978.49	981.49
990.48	992.31	1021.78	1024.25	1031.09	1046.03
1058.24	1060.22	1062.63	1063.66	1063.88	1076.28
1080.76	1082.06	1091.03	1100.71	1133.80	1145.95
1170.43	1172.35	1182.70	1182.97	1195.42	1196.08
1225.11	1233.00	1239.59	1242.81	1243.99	1250.17
1253.27	1274.03	1280.09	1296.07	1301.49	1311.75
1317.03	1317.99	1324.87	1328.40	1330.02	1333.34
1339.98	1343.05	1344.53	1351.59	1370.50	1409.25
1413.41	1427.86	1428.42	1439.51	1442.74	1445.57
1467.90	1471.41	1474.37	1475.68	1476.03	1479.59
1479.93	1481.77	1482.06	1487.64	1488.74	1496.35
1497.76	1498.00	1499.61	1500.80	1502.91	1508.58
1510.13	1510.94	1511.22	1517.87	1519.34	1525.45
1535.13	1594.12	1605.01	1612.45	1625.41	1633.91
1646.29	1649.52	1655.09	1828.98	3023.07	3026.50
3030.66	3033.00	3039.84	3045.85	3047.89	3048.42
3058.79	3066.38	3084.09	3085.89	3088.89	3093.94
3100.65	3102.86	3108.86	3114.40	3115.82	3124.77
3131.34	3133.54	3134.47	3145.62	3148.60	3152.04
3162.88	3163.57	3163.68	3164.82	3166.09	3170.58
3174.75	3177.63	3178.43	3182.50	3188.15	3189.08
3193.39	3197.28	3201.24	3201.60	3211.43	3215.92

==============================

T4

==============================

24.67	25.75	27.38	30.48	33.97	40.63
44.14	48.53	49.83	59.50	62.33	72.26
85.22	87.07	91.33	102.33	110.96	116.98
121.15	129.91	137.09	143.60	148.33	150.64
155.99	167.53	170.12	186.72	189.07	199.11
214.50	222.75	227.10	230.17	232.71	241.79
246.38	249.83	254.18	257.60	262.01	275.81
281.88	287.37	299.45	304.25	313.34	322.05
322.43	327.92	337.70	345.94	368.64	378.45
381.62	398.95	408.16	411.49	413.38	438.25
439.37	446.06	450.58	450.89	464.77	471.52
482.04	491.84	499.39	521.89	522.78	536.52
538.11	550.76	559.50	564.75	625.06	628.89
642.31	660.90	673.74	675.12	699.06	703.10
706.64	714.59	718.64	725.16	726.10	729.62
730.97	735.66	747.84	767.38	772.17	788.05
826.81	832.74	843.18	846.42	848.90	851.51
853.67	873.06	874.96	885.66	886.82	893.57
896.28	897.32	899.88	903.04	905.80	923.99
925.78	932.27	933.41	935.06	945.44	956.05
963.89	966.23	968.44	969.95	978.09	983.16
990.31	998.55	1022.52	1024.60	1032.12	1055.39
1057.52	1064.15	1064.27	1065.22	1073.26	1076.24
1080.06	1089.97	1101.14	1135.85	1145.78	1170.02
1173.79	1185.76	1195.09	1195.83	1214.86	1219.33
1224.58	1231.50	1238.15	1243.19	1243.64	1248.97
1274.96	1290.34	1295.77	1296.57	1298.05	1312.42
1319.24	1329.38	1330.16	1333.29	1337.16	1338.54
1345.35	1346.23	1349.40	1374.00	1378.27	1407.96
1412.80	1427.92	1428.76	1437.07	1441.71	1443.49
1467.41	1467.63	1469.83	1472.87	1476.79	1478.14
1479.07	1482.17	1485.16	1485.98	1486.76	1495.49
1496.45	1498.28	1498.79	1499.92	1503.52	1508.52

1509.82 1510.26 1511.44 1518.17 1519.45 1524.73
1533.80 1591.71 1601.96 1620.13 1631.65 1640.65
1650.57 1651.41 1656.40 2892.38 3022.68 3027.35
3029.83 3033.29 3036.32 3046.08 3047.27 3049.60
3051.56 3073.42 3083.89 3084.77 3088.73 3089.89
3093.07 3098.74 3109.88 3113.51 3115.34 3118.55
3129.54 3129.85 3132.71 3137.24 3146.43 3154.21
3154.85 3156.30 3156.64 3163.10 3164.47 3167.35
3171.09 3171.99 3178.45 3178.86 3189.73 3191.98
3198.04 3201.32 3202.50 3202.67 3213.96 3260.48

==============================

T5

==============================

15.18 19.10 29.24 30.52 32.67 37.44
47.14 49.01 52.70 58.82 62.46 63.22
65.49 68.91 69.17 76.62 81.41 85.64
96.49 98.74 105.46 112.65 119.87 128.07
133.22 136.38 143.06 147.49 151.75 159.87
161.25 167.09 174.63 177.37 180.16 187.13
189.76 201.26 209.80 221.16 222.81 232.36
235.52 237.09 240.47 243.21 246.08 250.15
253.82 258.55 268.01 273.50 280.09 285.24
294.45 298.93 301.06 302.74 307.22 326.34
327.29 330.56 336.52 338.25 352.37 356.84
358.89 378.44 380.68 391.37 405.16 409.50
411.29 414.20 418.10 435.06 435.91 441.99
442.73 449.27 454.85 456.62 470.87 474.85
477.05 480.28 484.22 493.49 501.21 507.40
519.28 523.89 525.73 536.96 541.56 552.25
566.70 600.13 624.82 625.60 629.23 642.85
662.56 670.96 672.28 677.91 688.62 698.16
702.63 703.15 710.64 714.23 721.16 722.66
725.70 727.03 731.78 735.63 739.14 747.15
753.12 769.01 771.13 777.54 780.54 786.69
817.26 820.56 831.09 844.42 847.16 849.12
854.39 861.38 862.27 873.17 879.11 883.85
885.21 886.54 889.17 894.33 897.28 899.25
900.78 908.28 915.69 918.87 924.36 932.27
934.51 937.82 947.32 959.31 965.07 966.78
970.91 973.99 975.26 981.81 983.74 985.96
992.26 996.07 1005.30 1013.47 1022.25 1024.89
1036.38 1055.28 1056.08 1058.33 1061.26 1062.54
1063.20 1068.59 1072.53 1076.33 1081.61 1089.84
1094.79 1098.78 1110.83 1141.30 1147.57 1150.66
1162.65 1176.92 1188.54 1191.86 1193.26 1193.62
1194.30 1205.10 1208.06 1218.06 1220.36 1223.40
1233.90 1244.09 1246.20 1247.69 1250.43 1269.68
1287.83 1291.94 1293.58 1296.83 1300.90 1303.61
1317.11 1320.33 1327.44 1329.39 1330.39 1339.26
1344.46 1345.40 1348.92 1352.49 1353.57 1369.02
1371.90 1378.21 1384.67 1405.14 1410.92 1424.78
1427.02 1435.80 1440.39 1445.42 1470.17 1470.75
1471.75 1474.41 1479.21 1479.27 1481.05 1482.74
1483.02 1484.26 1485.81 1489.15 1491.82 1494.37
1494.60 1495.40 1497.66 1499.70 1500.91 1505.62
1507.19 1508.49 1509.98 1510.41 1514.05 1517.00
1519.28 1523.01 1532.23 1580.02 1587.18 1589.98
1599.05 1619.51 1623.80 1630.76 1641.19 1650.85
1651.66 1651.95 1660.30 1662.42 2928.11 3019.37
3022.14 3023.57 3028.03 3034.67 3051.43 3053.58
3054.09 3056.83 3074.35 3077.25 3085.03 3088.95
3091.29 3101.67 3109.27 3110.35 3110.81 3125.64
3128.64 3132.80 3136.71 3138.00 3139.53 3149.04
3150.08 3155.02 3160.82 3162.90 3164.98 3168.37
3171.70 3175.33 3176.90 3177.90 3182.11 3182.98
3184.72 3190.64 3193.22 3195.32 3197.89 3201.10
3202.45 3204.03 3205.69 3207.76 3214.32 3236.20
3249.08 3270.60 3278.69

==============================

^{3}T6

==============================

10.82 22.77 23.84 29.14 31.54 40.58
46.06 51.42 53.14 58.82 64.43 64.96
65.88 68.00 71.94 76.45 89.77 99.67
109.90 110.57 120.05 128.87 137.37 137.55
144.29 151.62 155.29 166.22 169.31 173.82
182.18 201.74 204.45 208.17 212.02 215.45
224.85 229.07 236.17 240.12 249.26 251.30
252.97 260.35 268.15 276.18 291.70 298.25
314.90 329.32 332.68 338.14 339.81 351.71
356.17 360.14 364.55 370.16 383.14 405.00
413.79 416.11 417.69 444.66 451.40 452.37
456.91 459.84 463.72 473.73 478.33 482.42
484.04 494.57 517.17 519.65 529.84 534.88
546.53 553.75 564.11 597.97 624.05 627.96
630.01 636.89 662.80 679.48 680.32 682.96
686.00 698.75 706.35 709.15 711.28 715.30
726.84 731.33 731.93 733.66 742.63 745.30
749.01 760.42 761.14 766.88 772.33 794.58
802.89 832.33 840.73 845.65 850.02 851.28
862.62 865.54 868.55 871.93 878.09 888.36
894.43 895.59 900.90 901.84 902.99 904.82
921.81 935.37 935.92 937.96 941.66 942.89
952.90 957.70 967.89 978.66 980.61 980.96
983.49 984.25 989.51 989.83 1022.97 1025.72
1054.72 1059.07 1060.26 1063.16 1064.17 1064.73
1076.11 1080.50 1080.92 1081.61 1084.12 1088.30
1100.58 1144.77 1147.65 1149.68 1166.94 1185.51
1188.46 1190.54 1191.50 1195.93 1198.52 1198.96
1215.50 1228.91 1231.63 1243.08 1245.50 1260.36
1274.79 1293.60 1294.46 1301.43 1310.76 1319.51
1325.25 1326.06 1327.81 1332.35 1334.38 1337.82
1338.53 1340.21 1343.79 1348.69 1351.86 1362.75
1388.42 1397.00 1426.79 1428.57 1434.24 1441.68
1458.98 1467.59 1468.80 1472.61 1473.99 1475.93
1478.16 1478.63 1483.82 1484.18 1485.65 1487.97
1490.72 1497.83 1498.96 1508.27 1509.26 1509.67
1509.86 1510.90 1515.04 1521.72 1584.10 1585.65
1595.87 1597.77 1605.65 1618.03 1627.84 1629.75
1643.05 1650.77 1655.47 1661.35 3019.45 3026.76
3027.12 3047.87 3049.57 3049.75 3058.91 3081.73
3082.99 3112.69 3113.12 3131.13 3132.85 3137.45

3141.96 3153.88 3155.17 3155.46 3158.76 3161.27
3163.66 3171.95 3173.14 3173.22 3175.73 3177.43
3180.90 3181.14 3181.34 3184.29 3185.04 3193.86
3199.77 3201.40 3202.13 3202.88 3206.98 3208.59
3214.61 3225.02 3269.48

==============================

2

==============================

17.28 23.43 24.11 29.45 30.49 42.57
43.89 46.54 53.01 54.72 58.01 62.44
67.58 72.14 76.26 76.77 87.23 97.57
107.51 113.82 119.39 130.90 136.36 142.48
146.04 148.01 150.16 155.47 169.54 171.47
185.21 195.32 201.56 206.14 207.51 214.26
218.06 222.00 228.70 237.75 244.64 250.13
255.62 257.18 266.26 284.06 293.70 307.49
312.40 324.76 328.85 333.74 337.60 344.45
357.73 367.64 379.80 408.43 413.05 413.55
420.08 433.39 438.00 446.80 448.64 450.26
453.79 457.81 462.28 464.09 469.84 478.46
491.25 521.85 527.05 536.08 537.93 540.73
551.85 563.64 594.16 603.65 624.81 631.39
633.20 647.02 665.12 678.56 680.96 695.17
700.09 708.33 710.65 715.01 722.87 724.37
725.91 734.90 735.79 736.55 740.41 746.91
747.34 764.66 768.90 769.01 769.51 784.43
800.31 826.95 835.50 842.61 845.95 852.01
857.33 873.41 875.63 878.70 880.54 884.09
892.52 895.92 898.33 900.46 906.14 907.08
925.14 927.50 934.13 938.31 946.07 947.46
955.81 961.94 963.50 967.32 982.42 983.27
984.12 985.20 991.32 991.91 1024.68 1027.25
1057.78 1058.83 1063.25 1064.25 1064.57 1065.03
1073.39 1075.84 1077.09 1081.99 1084.92 1091.28
1136.91 1144.91 1146.67 1148.82 1172.70 1175.72
1176.45 1186.18 1194.66 1195.55 1196.46 1196.73
1219.71 1232.78 1240.53 1243.60 1245.00 1270.67
1280.64 1289.17 1296.53 1298.66 1299.94 1313.02
1316.05 1318.71 1326.36 1329.66 1331.99 1333.59
1334.50 1335.64 1337.39 1340.46 1345.17 1348.04
1351.01 1372.03 1428.84 1429.43 1438.63 1445.04
1465.01 1466.01 1468.08 1473.38 1474.89 1475.56
1477.31 1477.64 1478.29 1483.07 1484.71 1484.98
1497.31 1499.88 1500.75 1502.92 1506.51 1509.02
1510.03 1511.82 1514.49 1518.41 1596.05 1604.04
1607.38 1619.97 1620.71 1629.26 1631.80 1640.35
1643.79 1650.42 1651.01 1657.32 2902.09 3029.52
3030.80 3046.87 3050.21 3052.96 3058.40 3086.83
3090.68 3116.03 3118.30 3130.77 3134.94 3136.55
3149.07 3152.77 3159.98 3161.78 3163.24 3164.36
3166.87 3172.54 3174.51 3175.20 3176.39 3178.68
3179.43 3185.59 3185.99 3193.16 3193.84 3200.05
3200.55 3201.09 3202.20 3203.26 3204.78 3215.19
3215.55 3229.01 3265.00

==============================

^{3}T2-TS

==============================

-264.92 19.29 20.48 23.59 31.52 36.37
42.83 46.52 58.30 60.83 62.69 70.55
76.27 86.82 91.01 97.28 103.09 106.21
110.00 116.85 117.65 133.96 140.53 141.25
155.16 157.62 171.49 174.47 178.45 185.68
199.12 204.02 205.18 214.25 223.59 229.21
235.75 236.42 241.98 249.56 256.89 261.19
267.38 279.95 281.25 286.69 289.48 309.80
314.45 325.21 327.59 348.18 356.22 361.52
366.81 372.80 378.11 394.36 405.75 409.27
418.45 422.55 436.71 445.60 454.54 474.55
479.95 481.73 483.91 487.24 494.09 500.67
515.51 523.46 528.31 552.79 565.72 595.25
614.46 626.07 635.82 664.44 675.68 677.71
690.19 705.42 711.07 718.06 720.48 725.39
728.66 732.78 734.15 738.73 748.70 755.22
766.76 772.69 821.56 827.08 832.77 845.13
848.97 851.11 864.11 873.75 874.18 880.69
889.85 893.97 895.35 902.40 905.53 921.72
932.28 934.37 937.85 941.95 946.19 947.06
948.02 956.23 968.45 975.78 977.82 981.59
988.62 991.02 993.29 1023.22 1025.72 1040.85
1055.16 1059.88 1061.64 1063.99 1064.56 1065.11
1081.28 1085.61 1088.25 1089.08 1114.56 1154.11
1161.86 1187.28 1193.01 1195.84 1197.71 1203.72
1228.41 1230.23 1244.52 1248.02 1250.52 1279.88
1280.88 1291.29 1291.48 1306.89 1315.74 1319.94
1322.64 1329.35 1330.47 1336.95 1337.92 1342.37
1345.57 1348.02 1362.15 1367.46 1410.83 1414.21
1426.46 1427.75 1428.62 1434.21 1441.64 1452.93
1462.66 1467.51 1468.41 1469.97 1473.03 1475.94
1478.06 1480.82 1484.31 1486.24 1493.09 1493.34
1497.43 1498.74 1499.17 1500.53 1503.68 1507.87
1508.73 1509.68 1514.27 1516.62 1519.57 1523.33
1534.29 1581.70 1588.32 1589.41 1593.24 1603.80
1615.08 1635.11 1655.18 1662.56 3024.34 3025.23
3029.16 3033.71 3041.40 3045.02 3049.35 3051.15
3053.59 3079.06 3085.39 3091.96 3095.56 3101.78
3108.29 3111.12 3114.29 3129.30 3133.26 3134.17
3135.85 3145.82 3152.21 3153.94 3156.47 3159.93
3162.77 3163.89 3164.37 3165.88 3166.08 3170.71
3175.20 3175.25 3184.91 3192.27 3198.51 3200.61
3202.45 3207.47 3207.73 3213.28 3245.28 3267.31

==============================

^{3}T8

==============================

16.17 18.67 22.88 32.60 35.28 39.62
47.24 54.27 55.51 62.30 71.83 87.82
96.63 100.26 108.56 110.79 122.27 139.79
146.60 156.71 161.50 171.14 183.83 195.14
195.94 196.97 204.42 211.72 216.18 224.30
236.87 240.66 247.30 257.29 267.34 271.61
283.12 290.81 323.67 336.21 340.31 348.60
363.10 369.71 396.32 407.11 411.41 417.94
441.63 449.25 451.94 462.68 471.22 472.36

478.32 481.22 486.28 515.47 522.08 530.45
554.10 566.62 608.17 623.77 632.33 668.11
672.61 676.09 695.36 702.14 707.44 717.52
727.32 729.53 730.56 732.16 738.64 748.86
763.80 771.42 824.44 832.06 836.67 845.89
847.42 848.76 860.89 869.33 870.39 889.68
891.61 895.62 902.92 905.96 917.79 932.38
937.04 937.93 941.87 942.54 967.90 978.19
978.97 980.81 984.29 984.96 1022.94 1025.50
1061.36 1064.83 1065.11 1066.55 1080.68 1084.16
1088.42 1089.50 1152.30 1158.93 1182.01 1188.43
1194.84 1197.49 1203.53 1223.20 1242.03 1245.32
1274.92 1288.77 1295.89 1307.02 1318.65 1319.64
1329.17 1330.04 1333.85 1337.73 1341.41 1345.40
1347.43 1358.59 1373.48 1427.64 1428.67 1435.28
1442.26 1460.70 1467.86 1468.87 1469.27 1469.69
1475.81 1479.50 1481.55 1482.05 1483.58 1488.86
1498.45 1499.11 1508.09 1509.29 1510.00 1510.74
1518.53 1545.53 1588.69 1590.54 1600.92 1604.10
1614.00 1634.77 1654.46 1659.43 3029.14 3029.70
3046.00 3046.18 3049.87 3053.42 3085.46 3086.16
3115.21 3116.01 3133.36 3134.82 3135.05 3136.94
3147.78 3150.20 3154.95 3158.47 3160.06 3162.11
3165.17 3168.24 3175.71 3176.20 3187.11 3191.94
3202.96 3203.94 3206.37 3206.70 3221.38 3251.76

==============================

W2-TS

==============================

-92.30 21.15 29.19 36.34 43.44 49.25
52.11 60.61 67.58 71.23 75.03 77.47
81.09 85.76 91.89 97.26 107.92 110.90
120.22 124.90 132.86 142.21 149.05 154.22
169.06 173.21 174.62 180.23 183.75 190.38
193.24 201.26 210.53 213.57 223.91 228.95
241.41 247.10 248.45 260.19 265.27 270.54
274.92 288.16 295.01 299.33 307.59 311.07
322.10 332.49 336.07 346.76 350.18 365.33
371.06 381.12 395.13 404.19 404.83 416.18
418.30 433.40 445.74 448.18 458.55 467.57
469.93 471.25 478.37 504.41 511.03 518.28
521.89 531.02 541.36 553.16 561.10 571.27
585.65 596.31 622.78 634.63 656.87 663.95
679.32 682.58 696.12 706.22 711.74 714.28
723.64 730.31 735.15 741.22 747.93 749.29
762.11 772.79 775.07 793.47 816.48 817.59
833.24 837.97 842.86 849.01 874.67 884.57
886.33 888.95 890.99 895.09 905.21 907.19
913.70 922.28 924.68 933.07 936.46 941.26
966.71 968.02 968.43 970.66 973.78 976.66
978.92 980.99 1002.00 1005.32 1025.03 1025.41
1044.30 1053.76 1057.91 1064.63 1064.90 1066.18
1078.66 1080.01 1088.61 1090.48 1138.36 1147.80
1149.44 1169.22 1178.97 1191.64 1196.80 1197.61
1198.56 1213.92 1225.95 1242.49 1244.79 1254.72
1262.53 1278.68 1284.32 1289.39 1297.22 1307.37
1311.18 1318.43 1320.54 1326.76 1327.68 1332.06
1343.30 1344.30 1347.04 1358.37 1402.48 1413.27
1427.39 1428.41 1432.53 1440.36 1444.95 1448.88
1467.55 1472.45 1474.59 1475.52 1477.36 1477.85
1479.04 1481.91 1486.62 1488.67 1492.70 1494.00
1498.02 1500.11 1502.29 1504.58 1505.35 1507.85
1508.86 1510.06 1510.67 1515.75 1517.04 1523.79
1539.09 1593.54 1606.49 1611.51 1624.54 1639.16
1646.00 1647.54 1660.23 3012.86 3025.17 3027.07
3028.64 3032.21 3034.63 3035.97 3049.51 3052.12
3054.58 3072.89 3082.66 3088.91 3090.37 3098.90
3103.17 3111.64 3116.93 3117.82 3118.31 3133.86
3135.04 3136.78 3141.42 3142.90 3144.12 3144.29
3150.55 3151.44 3158.66 3161.43 3163.45 3170.83
3178.77 3179.10 3179.92 3180.34 3191.26 3194.55
3203.70 3206.35 3212.66 3226.76 3229.54 3269.37

==============================

W5

==============================

18.60 26.31 40.91 48.55 54.01 58.25
69.49 78.54 94.92 99.58 104.04 128.40
132.47 145.24 149.34 156.57 163.23 167.61
175.61 189.22 195.08 199.16 199.84 211.33
216.42 238.03 240.83 259.84 263.68 278.76
292.34 325.62 347.27 351.56 372.89 410.28
417.10 449.19 466.57 467.83 472.86 487.20
526.13 532.70 549.15 563.70 581.84 628.07
678.68 681.13 706.81 711.52 733.39 737.16
738.08 749.74 830.88 834.76 847.38 848.35
849.37 871.82 890.50 893.27 894.86 903.08
904.11 923.74 935.55 938.56 968.37 976.36
980.53 982.11 1022.67 1025.38 1042.42 1064.04
1064.44 1081.14 1090.26 1181.33 1184.87 1188.08
1236.13 1244.13 1244.94 1296.83 1317.06 1318.91
1328.36 1329.30 1331.74 1334.90 1345.19 1345.80
1427.92 1428.73 1438.62 1439.41 1445.83 1452.14
1465.68 1467.92 1469.29 1469.70 1478.72 1479.83
1481.21 1483.80 1497.23 1498.57 1508.51 1510.50
1511.20 1521.00 1591.24 1600.71 1654.16 1660.30
2982.05 3028.96 3031.35 3047.49 3049.48 3050.84
3051.40 3054.00 3080.79 3083.21 3088.47 3114.20
3116.24 3135.51 3136.98 3138.19 3140.52 3148.07
3153.33 3156.04 3156.56 3161.56 3163.07 3166.88
3168.88 3201.03 3203.75

==============================

W2

==============================

24.91 30.77 34.77 39.42 46.37 54.90
58.72 60.00 68.24 71.65 74.48 77.85
80.19 90.22 95.81 99.12 103.16 114.84
117.24 123.27 126.03 134.83 140.63 147.47
152.82 159.84 168.96 177.03 184.77 190.13
192.74 202.58 207.12 215.32 228.58 234.84
238.92 241.62 243.19 248.71 252.53 255.48
261.01 267.07 283.04 291.41 294.59 304.49
310.68 319.86 322.19 336.92 339.40 354.05
357.67 367.21 374.03 382.31 402.90 416.29

419.49 421.74 442.09 448.23 456.82 459.96
465.61 476.92 478.26 480.94 489.79 495.42
504.67 513.31 521.93 532.26 560.63 567.15
578.73 606.70 621.82 623.76 634.04 637.61
658.04 675.93 679.25 683.50 694.80 703.50
709.73 723.43 731.13 740.75 742.20 746.62
754.34 779.47 788.56 802.85 813.60 816.92
832.87 836.97 843.07 850.74 875.89 882.83
886.33 888.89 890.56 894.81 903.93 904.57
921.37 927.94 929.60 932.44 935.70 945.01
960.57 964.52 969.41 973.56 977.54 979.32
979.52 983.99 1007.36 1024.62 1024.74 1025.20
1047.73 1059.07 1063.83 1065.42 1065.90 1069.03
1082.11 1093.59 1094.43 1098.77 1155.99 1159.37
1169.25 1171.93 1174.26 1191.94 1203.97 1207.04
1210.75 1222.59 1229.80 1245.35 1247.01 1271.67
1278.14 1296.28 1298.81 1307.52 1309.80 1310.64
1318.40 1324.02 1327.27 1330.19 1341.26 1344.88
1356.43 1367.78 1373.84 1401.93 1409.71 1425.15
1428.02 1430.35 1437.74 1445.91 1456.98 1461.24
1466.48 1470.59 1473.57 1474.75 1478.73 1478.85
1480.11 1484.32 1488.50 1490.71 1495.36 1497.27
1499.47 1500.04 1500.13 1502.99 1505.62 1509.08
1509.60 1512.63 1514.07 1517.12 1519.95 1530.88
1584.63 1592.53 1597.99 1605.84 1619.51 1644.18
1644.59 1656.42 1660.02 2889.32 2983.00 3016.97
3020.52 3021.86 3030.92 3035.61 3036.56 3039.58
3046.42 3050.05 3058.64 3078.79 3082.44 3086.91
3091.20 3094.70 3100.68 3103.74 3107.71 3114.97
3116.56 3117.27 3118.89 3119.16 3130.42 3137.00
3142.00 3152.08 3153.11 3157.22 3159.71 3164.00
3169.83 3180.14 3182.02 3185.50 3186.05 3195.82
3196.71 3209.22 3211.10 3215.28 3217.81 3243.37

================================

W3

================================

11.29 23.16 29.45 35.56 40.69 42.86
49.44 52.66 55.30 57.27 59.25 68.91
74.85 91.00 94.00 97.47 110.00 110.21
114.91 119.44 125.44 130.37 132.94 152.66
156.20 169.57 173.87 176.38 187.63 189.59
203.49 210.48 213.41 219.84 233.82 239.68
241.94 249.79 257.10 258.55 264.18 268.45
276.10 279.31 287.24 293.08 308.60 312.48
316.24 322.87 329.52 338.42 347.76 361.30
373.41 396.38 398.34 402.57 406.25 419.26
424.22 452.03 455.56 459.37 461.92 468.30
479.19 481.29 507.24 513.62 522.14 528.43
532.96 558.07 563.00 567.04 573.40 581.33
595.91 624.02 639.10 644.57 658.46 675.46
680.08 693.93 704.33 707.94 715.61 725.26
733.35 737.75 739.79 749.28 754.39 767.38
771.06 777.41 801.11 820.17 833.37 839.55
844.90 856.91 868.34 873.67 875.51 888.86
893.54 895.20 900.34 902.42 907.79 918.35
921.12 927.59 932.93 939.60 940.79 953.56
957.88 965.31 966.16 974.87 978.70 981.98
985.09 985.57 986.53 994.92 1024.91 1025.48
1057.54 1061.88 1064.50 1064.82 1066.29 1069.66
1075.16 1083.25 1093.83 1094.37 1106.97 1152.50
1172.09 1173.94 1180.62 1187.06 1194.41 1200.73
1201.59 1210.43 1219.42 1229.13 1245.17 1246.63
1254.43 1280.80 1299.75 1302.79 1306.37 1308.42
1312.95 1319.29 1328.82 1328.97 1333.35 1336.43
1346.10 1348.14 1350.62 1383.10 1406.44 1415.27
1426.40 1428.58 1428.92 1429.54 1442.81 1443.13
1444.52 1465.37 1468.20 1470.35 1474.58 1475.88
1478.17 1479.54 1488.69 1491.60 1493.26 1495.75
1498.84 1499.97 1501.81 1507.22 1507.89 1509.37
1511.52 1513.26 1516.95 1518.46 1521.11 1523.52
1537.66 1599.37 1603.69 1611.58 1627.44 1643.03
1646.87 1651.25 1654.10 2936.40 2994.37 3028.54
3028.89 3034.29 3035.24 3036.56 3040.18 3042.51
3045.70 3061.20 3082.19 3086.59 3092.11 3093.26
3098.30 3113.89 3114.52 3119.14 3119.33 3121.30
3125.36 3132.97 3134.29 3137.13 3144.26 3145.73
3148.43 3152.03 3164.93 3168.37 3173.12 3173.64
3174.69 3175.10 3175.33 3186.28 3188.45 3193.56
3202.22 3202.37 3218.52 3219.48 3227.55 3249.61

================================

W3-TS

================================

-310.55 21.68 26.73 29.93 36.04 41.82
58.01 61.62 62.51 64.34 66.31 69.00
78.62 84.47 95.48 98.99 109.53 113.01
113.98 115.27 119.30 127.48 131.83 146.26
154.77 156.08 167.24 173.78 179.67 183.63
202.65 203.57 207.81 232.62 235.49 237.74
243.53 244.75 249.27 255.94 259.60 263.09
275.41 280.02 282.05 287.26 301.23 302.75
312.15 319.24 325.84 327.83 338.74 352.80
362.32 370.30 375.58 396.28 405.61 407.04
415.05 422.36 450.05 454.27 461.04 465.73
469.23 475.48 478.09 507.62 511.25 520.95
525.03 530.81 539.41 560.58 565.81 580.94
606.15 611.19 622.74 637.34 649.49 658.01
674.60 685.11 694.86 703.47 705.94 718.24
719.68 733.32 739.12 741.76 747.97 764.35
771.53 788.31 801.03 815.47 830.72 838.68
844.51 855.59 872.67 873.42 877.84 887.11
888.10 889.88 893.08 899.05 901.10 907.48
917.99 926.87 932.92 934.22 939.01 947.24
961.05 963.91 965.83 968.56 970.87 974.25
985.28 987.76 990.35 994.69 1024.60 1025.26
1061.49 1063.38 1064.45 1064.60 1071.91 1074.26
1082.33 1085.10 1093.21 1094.27 1144.09 1159.11
1174.47 1180.35 1184.52 1189.55 1205.05 1208.56
1220.56 1221.25 1230.69 1245.96 1246.61 1247.47
1266.60 1277.30 1292.06 1302.97 1309.67 1311.63
1321.31 1325.37 1327.62 1328.12 1332.06 1342.07
1345.01 1349.22 1359.22 1410.73 1413.93 1421.81
1425.98 1428.19 1429.96 1430.84 1439.81 1446.66

1455.50 1466.22 1473.44 1474.65 1476.33 1476.40
1479.00 1486.25 1488.37 1493.00 1496.69 1499.29
1499.67 1499.74 1504.41 1507.55 1508.95 1511.86
1512.43 1513.31 1520.73 1521.19 1522.33 1525.84
1540.38 1595.11 1606.73 1608.44 1624.73 1647.88
1650.10 1654.51 1655.21 2996.36 3026.55 3032.94
3033.97 3034.94 3038.23 3040.03 3041.30 3042.53
3048.23 3052.07 3083.99 3087.48 3090.47 3098.73
3104.83 3113.54 3116.48 3117.56 3120.04 3120.30
3122.52 3130.18 3130.27 3131.82 3132.52 3148.75
3153.16 3154.64 3161.72 3162.14 3162.49 3169.97
3176.47 3179.50 3181.73 3190.97 3192.11 3194.68
3206.15 3207.10 3220.40 3237.51 3240.43 3241.02

================================
W4
================================

23.14 31.35 41.42 45.83 57.97 61.63
69.58 76.46 98.85 100.38 109.64 118.62
137.10 139.05 147.13 157.35 172.85 185.77
189.85 200.20 202.92 213.87 229.56 237.58
242.20 253.07 269.72 275.87 289.00 307.79
321.82 335.31 354.31 372.05 403.96 416.63
455.08 469.05 471.95 510.02 520.88 553.13
562.40 565.36 630.16 659.10 681.44 694.52
703.61 708.03 736.36 740.93 748.16 794.02
824.68 834.35 839.98 846.86 854.65 872.46
874.21 889.40 893.70 901.49 905.05 906.63
920.63 925.77 933.04 938.88 968.15 973.34
975.15 986.29 1025.11 1026.18 1042.83 1064.45
1064.54 1079.87 1093.00 1167.50 1184.65 1231.31
1242.99 1244.36 1297.32 1310.76 1319.67 1325.66
1326.60 1329.12 1331.99 1346.13 1346.43 1349.07
1427.16 1428.82 1432.76 1442.04 1466.52 1470.13
1475.29 1477.42 1477.82 1479.44 1484.03 1488.15
1491.77 1495.02 1499.01 1501.01 1506.42 1509.47
1514.19 1517.61 1595.95 1604.56 1646.20 1657.58
2004.57 2044.75 2284.73 3029.76 3034.36 3037.13
3040.80 3050.27 3053.81 3087.15 3094.10 3114.93
3120.27 3120.77 3121.08 3134.47 3135.94 3137.50
3156.20 3160.59 3161.23 3163.11 3167.23 3173.16
3187.86 3197.64 3209.42

================================
W2’
================================

21.11 28.72 32.19 38.70 42.32 54.70
59.29 66.51 69.18 71.02 74.03 78.52
81.58 84.98 96.01 97.50 104.87 113.14
117.65 120.32 127.15 134.03 139.75 147.75
150.23 161.43 162.22 176.28 180.39 189.76
195.83 200.17 206.96 217.62 223.32 237.50
243.50 246.87 253.06 254.26 257.51 262.81
268.55 284.93 289.19 293.50 299.47 304.44
311.96 319.34 322.26 333.86 342.78 358.21
367.92 372.87 382.63 395.30 401.81 420.74
422.66 442.63 447.59 451.06 458.82 466.46
469.22 478.15 489.08 513.35 521.99 526.02
534.24 551.32 561.35 566.75 569.19 574.47
600.42 610.42 623.98 632.41 637.41 659.60
666.08 677.62 681.65 694.17 701.72 707.95
721.65 730.26 731.50 740.23 745.66 775.00
785.86 789.95 808.24 814.99 832.85 835.73
843.45 850.18 858.39 875.15 882.82 888.56
889.06 890.03 893.83 900.76 903.93 904.78
920.10 927.71 929.29 932.57 935.13 951.11
960.09 966.25 968.16 973.64 976.50 976.85
978.84 985.39 999.96 1023.17 1024.43 1026.35
1048.75 1058.48 1059.42 1063.81 1066.91 1068.94
1081.70 1093.85 1138.04 1149.69 1162.42 1169.20
1172.31 1190.19 1191.20 1192.98 1222.47 1223.37
1229.52 1244.80 1247.47 1255.34 1266.58 1271.52
1286.55 1296.26 1300.46 1308.99 1310.10 1317.01
1321.00 1323.32 1327.13 1329.82 1341.32 1345.12
1380.16 1386.76 1394.40 1402.37 1409.70 1426.53
1428.25 1430.95 1437.77 1446.59 1451.01 1455.68
1466.12 1471.35 1474.29 1478.63 1480.00 1486.85
1488.58 1491.42 1497.29 1498.53 1499.91 1500.02
1501.16 1502.64 1503.56 1505.84 1509.10 1510.36
1514.34 1514.66 1517.15 1523.03 1530.37 1531.43
1591.87 1604.54 1613.90 1618.52 1645.66 1649.85
1659.33 1659.83 1682.27 2876.36 2984.38 3016.25
3020.02 3022.09 3030.63 3032.89 3034.65 3042.24
3046.97 3051.25 3059.82 3078.44 3085.52 3085.91
3090.42 3092.31 3099.88 3104.78 3108.17 3115.77
3115.86 3116.28 3120.16 3120.28 3130.67 3136.45
3137.88 3153.83 3154.32 3157.03 3168.04 3170.91
3171.23 3174.72 3184.58 3190.31 3191.78 3207.68
3207.83 3215.64 3219.79 3219.86 3220.16 3225.99

================================
W2’-TS
================================

-95.25 25.30 31.57 35.80 47.62 49.92
55.36 67.26 68.79 70.18 74.62 77.72
82.68 86.79 90.67 99.29 104.72 116.76
117.79 124.15 130.60 139.56 148.46 152.95
157.97 170.41 173.14 179.01 184.98 187.80
193.97 205.21 214.63 219.67 232.70 236.10
243.44 246.65 247.14 257.20 259.57 273.58
279.54 282.25 297.12 304.34 316.93 322.01
326.66 332.84 344.52 355.09 360.93 372.50
382.63 389.38 397.87 405.49 415.45 419.62
443.94 449.22 454.00 457.93 469.67 474.18
479.59 496.02 507.91 516.60 521.61 546.89
548.33 558.45 560.29 569.84 571.62 594.39
596.72 623.81 634.53 653.56 661.01 667.33
680.68 697.22 701.84 712.68 714.65 724.57
726.15 737.91 738.16 746.77 771.02 776.73
779.85 786.14 815.57 835.13 836.99 838.83
842.42 844.09 850.86 872.00 879.78 884.41
885.41 888.96 892.42 893.43 904.19 906.76
915.22 920.38 932.41 939.65 941.48 944.45
962.50 963.17 965.46 970.49 974.67 978.42
980.52 986.04 992.22 995.66 1025.07 1026.05

1046.32 1058.62 1060.98 1064.07 1064.75 1065.88
1080.50 1089.74 1110.57 1125.26 1157.83 1167.95
1173.97 1186.63 1190.40 1192.65 1195.96 1213.58
1226.42 1240.82 1243.22 1245.41 1249.63 1266.91
1283.57 1295.80 1296.14 1297.68 1302.98 1313.36
1319.69 1325.76 1327.53 1331.58 1332.86 1338.35
1342.43 1345.44 1365.76 1372.33 1405.10 1417.99
1427.12 1428.44 1434.67 1444.28 1445.41 1450.01
1468.42 1470.69 1475.40 1477.74 1478.45 1481.82
1485.82 1487.63 1493.31 1494.93 1495.85 1499.83
1501.54 1502.80 1503.07 1505.73 1507.43 1508.85
1509.44 1511.29 1515.91 1519.39 1521.88 1531.49
1540.21 1592.34 1605.38 1623.41 1645.22 1647.06
1652.43 1660.76 1673.39 3017.47 3022.36 3025.12
3032.16 3032.74 3037.07 3037.51 3047.94 3053.50
3053.98 3056.54 3082.10 3088.07 3091.97 3095.07
3103.95 3111.56 3118.21 3119.99 3121.65 3125.47
3132.61 3134.62 3134.94 3137.56 3138.21 3139.05
3152.12 3160.96 3168.72 3176.12 3176.95 3177.15
3181.82 3185.11 3186.46 3187.65 3200.65 3204.38
3213.93 3214.89 3215.21 3226.54 3232.74 3276.24

===============================

W3'

===============================

22.16 23.41 28.41 39.29 41.56 48.91
53.84 57.93 58.26 61.49 65.31 74.02
76.32 91.78 98.00 105.03 111.88 113.73
115.37 122.96 125.10 131.15 147.99 151.34
152.41 166.16 176.64 187.45 193.38 196.61
205.93 220.50 227.66 232.47 237.49 241.43
243.92 255.32 261.54 265.43 267.90 275.92
279.77 286.07 290.14 309.51 311.06 322.65
324.94 327.39 337.36 339.26 360.42 372.81
384.22 400.12 405.50 409.52 417.47 433.96
435.13 452.92 463.86 469.41 478.37 486.06
499.08 509.83 531.75 533.60 544.85 556.31
559.54 564.00 566.01 574.81 585.39 604.22
623.72 638.23 640.67 657.79 662.51 676.98
693.85 703.84 706.50 706.91 724.42 731.04
739.27 741.94 748.75 763.24 773.58 778.22
798.74 820.33 832.00 834.17 839.54 845.28
858.48 871.27 874.11 875.36 880.58 888.90
893.30 901.69 902.05 905.10 908.15 917.44
924.49 930.37 933.12 938.86 940.02 953.62
960.38 966.36 966.86 974.76 977.42 978.64
983.94 987.11 989.40 992.69 1024.79 1025.38
1060.56 1063.26 1064.59 1064.71 1066.43 1067.57
1082.44 1083.34 1093.64 1127.30 1155.84 1171.59
1179.90 1186.81 1188.85 1191.10 1207.62 1217.26
1221.19 1230.96 1244.29 1246.70 1247.29 1251.07
1279.30 1286.44 1296.40 1306.81 1311.67 1313.18
1318.69 1320.37 1326.71 1329.10 1333.33 1346.25
1350.80 1359.41 1367.73 1380.63 1407.73 1414.62
1426.30 1428.49 1429.44 1429.80 1441.49 1443.72
1444.97 1465.34 1472.04 1476.16 1478.35 1480.16
1488.75 1491.18 1492.68 1494.44 1497.15 1498.80
1499.91 1500.60 1503.53 1507.29 1509.14 1509.34
1512.03 1516.54 1518.16 1522.35 1525.14 1536.28
1540.00 1598.18 1603.88 1626.87 1647.20 1651.37
1653.05 1657.65 1677.43 2943.08 2997.99 3029.22
3031.80 3034.73 3035.31 3036.48 3039.63 3041.49
3042.62 3060.41 3085.36 3087.08 3093.38 3093.69
3101.79 3112.06 3114.96 3118.61 3118.66 3119.12
3120.23 3131.87 3134.28 3139.09 3144.75 3145.22
3146.13 3154.28 3163.05 3169.13 3174.06 3174.25
3175.13 3180.50 3183.11 3193.14 3193.89 3199.12
3205.22 3212.42 3212.72 3216.52 3231.72 3234.89

===============================

W3'-TS

===============================

-290.48 16.87 26.65 32.11 34.67 41.63
56.57 58.82 63.28 63.84 67.33 70.65
80.28 91.41 96.21 102.38 109.47 110.58
114.31 119.06 123.78 125.24 129.52 148.18
153.08 154.84 164.36 177.20 185.08 186.88
201.25 203.19 230.04 231.56 237.39 242.25
247.07 249.23 253.16 257.46 258.54 275.00
275.73 281.42 284.23 290.97 297.96 312.21
313.85 321.05 326.60 329.57 342.01 361.58
368.59 388.69 396.36 405.48 410.46 415.14
422.23 432.63 454.31 467.24 473.18 476.82
492.38 508.65 509.78 523.05 531.01 537.55
544.49 558.90 563.54 570.64 578.50 606.58
615.26 622.76 632.49 640.47 657.08 670.39
674.25 694.88 702.47 705.93 712.94 717.42
731.73 739.81 748.33 757.89 775.94 782.17
786.57 816.55 828.85 836.81 839.00 845.98
855.80 872.15 873.42 875.51 884.00 887.95
892.45 894.73 901.05 905.01 907.23 908.01
917.01 929.48 932.79 933.42 939.23 947.97
962.26 966.16 968.92 969.43 972.95 974.57
986.00 986.35 987.84 996.76 1025.27 1025.60
1060.74 1063.75 1064.19 1064.44 1067.67 1073.90
1083.36 1093.26 1116.45 1136.04 1169.90 1171.87
1178.31 1188.87 1193.07 1202.14 1221.22 1222.69
1233.96 1246.26 1247.00 1250.64 1255.35 1263.84
1286.61 1290.03 1297.39 1303.56 1310.32 1318.69
1322.07 1324.26 1328.13 1332.66 1345.46 1349.55
1351.11 1369.77 1378.55 1410.58 1413.71 1424.01
1425.92 1428.09 1430.14 1435.67 1438.92 1446.37
1458.98 1466.75 1473.28 1476.31 1478.53 1487.51
1488.98 1493.53 1495.71 1499.23 1500.55 1500.84
1500.87 1503.41 1506.64 1507.13 1508.27 1512.09
1513.07 1518.78 1520.11 1522.41 1526.14 1537.23
1542.40 1596.41 1606.91 1618.38 1645.15 1649.57
1654.95 1658.91 1676.62 2995.01 3025.51 3032.09
3033.43 3034.59 3036.63 3037.75 3038.28 3040.76
3046.37 3051.21 3082.83 3084.02 3090.41 3098.46
3104.15 3112.93 3115.99 3116.32 3118.99 3119.12
3119.15 3124.58 3128.34 3130.11 3133.20 3144.37
3148.79 3153.85 3160.44 3163.36 3164.41 3170.03
3175.36 3186.60 3190.18 3191.00 3200.75 3204.32

3215.26 3216.72 3216.97 3219.45 3223.65 3232.89

==============================

W4’

==============================

23.14 31.35 41.42 45.83 57.97 61.63
69.58 76.46 98.85 100.38 109.64 118.62
137.10 139.05 147.13 157.35 172.85 185.77
189.85 200.20 202.92 213.87 229.56 237.58
242.20 253.07 269.72 275.87 289.00 307.79
321.82 335.31 354.31 372.05 403.96 416.63
455.08 469.05 471.95 510.02 520.88 553.13
562.40 565.36 630.16 659.10 681.44 694.52
703.61 708.03 736.36 740.93 748.16 794.02
824.68 834.35 839.98 846.86 854.65 872.46
874.21 889.40 893.70 901.49 905.05 906.63
920.63 925.77 933.04 938.88 968.15 973.34
975.15 986.29 1025.11 1026.18 1042.83 1064.45
1064.54 1079.87 1093.00 1167.50 1184.65 1231.31
1242.99 1244.36 1297.32 1310.76 1319.67 1325.66
1326.60 1329.12 1331.99 1346.13 1346.43 1349.07
1427.16 1428.82 1432.76 1442.04 1466.52 1470.13
1475.29 1477.42 1477.82 1479.44 1484.03 1488.15
1491.77 1495.02 1499.01 1501.01 1506.42 1509.47
1514.19 1517.61 1595.95 1604.56 1646.20 1657.58
2004.57 2044.75 2284.73 3029.76 3034.36 3037.13
3040.80 3050.27 3053.81 3087.15 3094.10 3114.93
3120.27 3120.77 3121.08 3134.47 3135.94 3137.50
3156.20 3160.59 3161.23 3163.11 3167.23 3173.16
3187.86 3197.64 3209.42

==============================

W5’

==============================

18.60 26.31 40.91 48.55 54.01 58.25
69.49 78.54 94.92 99.58 104.04 128.40
132.47 145.24 149.34 156.57 163.23 167.61
175.61 189.22 195.08 199.16 199.84 211.33
216.42 238.03 240.83 259.84 263.68 278.76
292.34 325.62 347.27 351.56 372.89 410.28
417.10 449.19 466.57 467.83 472.86 487.20
526.13 532.70 549.15 563.70 581.84 628.07
678.68 681.13 706.81 711.52 733.39 737.16
738.08 749.74 830.88 834.76 847.38 848.35
849.37 871.82 890.50 893.27 894.86 903.08
904.11 923.74 935.55 938.56 968.37 976.36
980.53 982.11 1022.67 1025.38 1042.42 1064.04
1064.44 1081.14 1090.26 1181.33 1184.87 1188.08
1236.13 1244.13 1244.94 1296.83 1317.06 1318.91
1328.36 1329.30 1331.74 1334.90 1345.19 1345.80
1427.92 1428.73 1438.62 1439.41 1445.83 1452.14
1465.68 1467.92 1469.29 1469.70 1478.72 1479.83
1481.21 1483.80 1497.23 1498.57 1508.51 1510.50
1511.20 1521.00 1591.24 1600.71 1654.16 1660.30
2982.05 3028.96 3031.35 3047.49 3049.48 3050.84
3051.40 3054.00 3080.79 3083.21 3088.47 3114.20
3116.24 3135.51 3136.98 3138.19 3140.52 3148.07
3153.33 3156.04 3156.56 3161.56 3163.07 3166.88
3168.88 3201.03 3203.75

==============================

X1

==============================

17.83 19.26 26.33 32.19 41.10 45.19
53.30 56.84 58.14 64.34 67.79 68.22
72.21 82.05 93.47 97.78 109.77 115.08
121.41 130.64 135.77 145.07 154.59 158.20
163.13 166.68 169.43 177.17 179.15 200.56
203.33 210.17 215.70 221.57 226.31 237.61
241.41 245.82 248.44 252.14 256.14 264.32
270.53 275.84 279.21 288.55 294.76 303.53
315.09 321.04 326.46 332.86 340.95 355.29
370.22 375.85 384.32 389.42 406.90 415.67
425.06 431.18 446.26 447.94 452.92 467.11
469.51 478.35 488.86 496.45 526.88 528.92
531.40 537.63 545.65 552.10 553.34 568.31
599.92 627.10 632.18 639.36 651.45 665.28
671.96 680.62 684.67 700.89 708.34 711.04
726.25 729.41 729.94 732.61 749.79 775.53
785.22 803.37 816.56 829.76 833.35 847.14
848.37 849.20 852.81 866.53 882.55 883.96
888.32 888.90 894.21 898.84 899.88 906.21
918.30 930.22 933.30 935.79 936.96 946.16
966.58 972.38 975.53 977.61 980.88 981.27
982.50 1002.82 1003.89 1005.20 1021.79 1024.49
1049.68 1057.12 1062.47 1062.56 1064.38 1066.30
1080.12 1088.56 1134.76 1145.60 1177.25 1182.72
1184.92 1187.67 1188.25 1188.92 1210.68 1223.88
1233.96 1242.67 1246.58 1252.80 1257.34 1277.38
1282.43 1295.04 1311.98 1318.30 1320.33 1323.84
1324.35 1325.37 1337.01 1337.50 1339.66 1340.78
1362.11 1380.87 1388.75 1400.63 1407.80 1426.35
1427.47 1436.45 1437.56 1444.86 1458.17 1464.46
1468.98 1471.75 1473.09 1474.23 1479.83 1481.94
1485.67 1488.58 1490.48 1498.10 1498.27 1498.92
1500.44 1500.47 1502.87 1507.85 1508.83 1509.70
1513.50 1514.69 1517.33 1522.35 1526.96 1532.75
1587.32 1597.03 1615.20 1635.44 1650.75 1658.22
1659.23 1669.46 1706.78 2817.67 2963.71 3015.68
3019.43 3023.71 3027.01 3031.33 3042.89 3048.77
3048.87 3050.58 3051.18 3076.73 3083.03 3083.17
3088.38 3096.70 3097.05 3103.90 3109.68 3111.52
3111.72 3125.70 3129.82 3137.99 3140.01 3150.13
3154.12 3154.24 3155.81 3155.95 3158.96 3161.42
3165.95 3167.50 3189.49 3190.89 3201.56 3205.99
3207.21 3210.09 3219.47 3219.84 3251.29 3262.94

==============================

^{3}X1-TS

==============================

-271.60 24.89 27.56 29.69 30.68 41.72
44.18 50.48 52.51 59.26 63.92 64.95
75.82 79.73 85.92 98.02 104.05 106.82
116.29 125.75 128.82 138.74 140.52 150.20
155.44 156.40 167.25 176.18 192.05 196.49
199.61 206.19 212.37 220.00 229.72 233.76

241.40 245.26 246.21 252.55 257.83 263.42
266.58 273.57 281.76 288.95 294.29 300.97
310.92 319.54 320.85 331.83 348.16 349.77
366.40 374.39 394.49 397.14 401.80 407.86
412.18 420.80 443.69 448.18 459.85 466.43
469.82 471.86 482.80 486.71 529.38 531.67
539.56 550.83 564.20 588.56 596.12 608.47
609.98 623.73 625.65 647.13 667.57 669.94
677.75 697.27 702.61 708.10 712.55 713.48
725.12 730.82 734.15 736.56 747.93 760.28
766.12 769.80 821.00 831.33 834.77 845.31
847.66 849.11 850.42 860.22 867.35 869.53
871.10 877.58 893.59 894.87 902.89 906.17
913.03 918.94 928.09 931.58 933.34 935.24
936.68 938.19 953.46 968.17 969.39 974.14
977.52 977.84 979.39 982.56 1022.57 1024.18
1036.55 1044.46 1053.19 1060.42 1064.18 1064.85
1078.76 1087.74 1123.32 1129.74 1147.90 1173.80
1177.61 1178.50 1183.74 1189.36 1195.97 1214.57
1220.72 1224.17 1239.60 1242.50 1244.70 1269.90
1293.64 1296.14 1296.91 1304.48 1312.29 1314.66
1322.37 1327.25 1328.18 1336.36 1342.98 1344.04
1350.31 1374.09 1376.76 1406.73 1410.50 1413.20
1426.62 1428.41 1434.92 1439.79 1442.89 1458.94
1465.63 1471.22 1471.90 1474.40 1476.61 1477.20
1484.01 1484.95 1486.18 1486.48 1491.81 1495.17
1496.45 1498.57 1498.72 1501.95 1503.54 1508.42
1508.75 1509.82 1518.04 1519.88 1522.33 1530.01
1535.92 1588.82 1590.94 1599.83 1609.37 1619.94
1650.68 1653.82 1659.14 3024.86 3026.75 3027.02
3030.00 3030.89 3036.80 3043.80 3046.05 3048.00
3048.35 3053.64 3081.52 3087.69 3090.32 3096.77
3105.85 3111.09 3113.18 3116.33 3129.89 3130.66
3131.58 3132.49 3136.82 3137.89 3148.45 3149.70
3153.06 3155.27 3155.59 3157.78 3161.20 3164.14
3164.62 3166.37 3180.71 3184.14 3195.45 3196.92
3198.72 3208.05 3210.45 3211.63 3214.66 3243.81

==============================

^{3}X2

==============================

20.64 23.50 27.48 35.51 39.84 43.85
47.11 53.99 58.02 63.25 68.05 72.19
75.88 86.09 94.95 101.05 107.68 113.21
117.29 120.12 129.24 140.03 146.29 148.83
155.57 164.49 165.41 187.81 197.23 201.14
205.93 211.86 216.83 227.36 230.91 241.25
247.93 249.64 254.48 261.65 262.68 267.92
279.17 284.47 289.95 298.44 301.78 307.10
314.34 325.39 333.29 344.90 352.00 368.70
377.75 384.25 397.07 402.07 408.51 414.59
415.98 441.69 450.95 455.96 467.66 475.43
478.47 482.98 500.00 529.25 533.62 538.65
544.49 552.41 563.91 590.84 600.04 621.53
628.81 635.74 674.97 676.54 678.19 705.38
710.39 715.19 722.92 723.32 727.64 729.75
738.72 739.49 748.40 765.17 766.39 832.83
837.83 846.44 846.96 849.72 856.39 860.22
867.28 867.57 876.09 885.59 890.09 894.13
896.76 903.56 906.33 913.92 922.14 931.84
932.62 934.48 936.40 937.53 939.33 945.93
966.59 970.71 974.83 976.69 979.25 980.64
980.98 1013.73 1022.69 1023.63 1025.53 1049.60
1058.81 1064.50 1064.74 1066.05 1080.49 1088.66
1103.67 1126.96 1136.45 1136.49 1181.56 1183.24
1186.20 1189.03 1199.92 1228.41 1230.82 1238.77
1244.20 1245.47 1246.50 1250.33 1263.23 1294.83
1300.72 1305.93 1312.32 1319.04 1321.15 1328.64
1329.74 1332.61 1343.65 1345.91 1346.98 1353.24
1375.60 1378.61 1404.09 1409.04 1410.75 1418.06
1427.49 1428.83 1435.33 1442.14 1443.92 1466.47
1469.01 1469.50 1470.33 1478.72 1479.37 1481.16
1482.86 1483.86 1494.97 1495.49 1497.20 1498.88
1499.22 1500.13 1503.38 1508.11 1508.27 1510.18
1510.41 1518.35 1520.52 1524.04 1526.59 1531.57
1536.54 1587.55 1597.18 1597.41 1615.11 1624.20
1653.76 1656.98 1659.21 2926.59 2992.36 3020.24
3022.08 3027.62 3029.90 3030.28 3043.74 3046.55
3046.96 3049.64 3053.38 3083.89 3084.12 3085.04
3085.04 3091.86 3094.02 3106.75 3109.37 3111.34
3113.51 3114.15 3132.35 3133.30 3134.24 3134.80
3152.02 3152.65 3154.50 3156.49 3162.52 3165.36
3165.67 3166.95 3183.58 3187.06 3198.78 3200.49
3201.78 3205.16 3212.76 3213.34 3242.86 3248.83

==============================

Y1

==============================

19.73 22.37 29.01 30.53 39.08 40.01
42.07 47.56 57.29 61.04 63.77 76.31
85.33 92.26 97.57 107.43 115.66 122.90
128.38 135.42 139.32 144.62 151.38 156.04
161.82 171.64 176.22 187.86 194.85 205.79
211.72 220.97 223.08 225.65 237.48 242.36
244.99 248.89 254.24 258.19 263.32 271.80
281.76 291.33 303.11 305.12 318.91 319.97
323.73 331.91 356.64 357.94 360.65 372.56
381.09 404.62 420.62 436.95 439.52 442.07
454.00 460.35 461.88 468.40 484.35 492.51
505.93 510.70 531.12 538.27 551.46 557.28
561.58 567.13 609.79 616.09 636.94 641.23
657.53 659.87 666.14 677.73 690.72 696.00
708.75 730.11 735.42 743.57 746.22 747.66
769.91 787.49 795.80 807.53 819.67 834.16
838.93 843.30 847.81 873.49 879.58 888.28
892.22 892.82 893.56 901.58 902.92 922.25
926.73 932.25 935.97 937.84 958.88 960.41
969.84 973.11 976.87 979.57 981.43 992.57
994.01 1004.21 1019.57 1022.34 1022.66 1031.09
1046.52 1062.08 1063.56 1063.83 1063.94 1078.92
1088.89 1090.58 1098.52 1136.82 1153.56 1166.51
1181.18 1186.89 1189.13 1193.84 1204.53 1220.52
1228.68 1243.14 1245.28 1288.08 1293.12 1301.59
1305.60 1308.57 1311.23 1322.83 1324.51 1327.36

1328.63 1334.04 1336.70 1342.01 1344.53 1348.42
1349.71 1402.50 1405.89 1424.87 1427.43 1429.78
1435.99 1447.26 1453.30 1460.47 1469.76 1470.87
1474.10 1474.65 1478.77 1479.64 1479.95 1490.12
1491.98 1492.60 1496.95 1498.38 1498.90 1499.29
1502.17 1506.45 1508.22 1511.29 1511.63 1513.67
1514.25 1514.89 1521.99 1530.91 1583.95 1596.53
1605.49 1621.99 1645.69 1649.18 1652.26 1659.47
2809.26 2992.04 3018.04 3020.93 3021.37 3023.14
3031.89 3034.22 3037.84 3040.51 3051.71 3058.68
3075.02 3082.06 3084.02 3088.49 3091.48 3098.85
3106.27 3108.96 3110.00 3112.67 3113.58 3114.47
3119.72 3131.97 3137.08 3138.07 3142.30 3149.62
3152.73 3153.46 3159.11 3160.88 3175.19 3193.93
3195.71 3201.54 3213.12 3213.26 3217.75 3224.24
3236.01 3252.91 3257.68

==============================

^{3}Y1-TS

==============================

-266.80 17.47 28.95 37.59 39.83 42.24
50.43 55.46 59.67 62.59 73.98 77.61
78.35 88.03 95.33 100.04 106.48 112.28
117.68 123.90 127.59 132.98 148.26 154.97
167.48 174.34 180.97 187.21 193.75 207.55
211.30 213.89 219.78 224.25 241.67 243.16
244.71 247.24 247.86 252.33 260.28 275.07
278.46 295.08 299.40 306.90 314.51 323.49
329.07 342.82 360.33 362.35 366.07 374.18
401.94 405.91 415.20 421.07 424.27 431.65
437.53 445.67 456.92 461.62 471.64 472.13
480.40 511.54 531.09 542.55 548.62 555.54
565.48 619.31 625.39 632.54 656.67 658.42
665.80 675.15 678.21 692.59 699.40 706.53
711.43 728.47 732.99 736.18 749.02 752.60
772.14 777.35 778.85 781.69 810.27 834.13
842.32 844.31 848.03 851.22 860.12 875.20
887.46 892.61 894.37 901.01 902.10 917.01
922.83 932.44 933.42 935.96 952.36 952.84
961.18 963.76 967.78 968.09 973.87 977.68
978.82 983.09 985.60 1023.08 1023.70 1023.76
1033.67 1046.45 1051.46 1063.29 1064.47 1065.13
1071.60 1082.29 1090.88 1130.15 1157.05 1163.60
1166.64 1174.31 1188.15 1188.89 1198.66 1209.43
1225.07 1242.83 1244.86 1263.42 1274.16 1293.35
1305.95 1310.47 1312.03 1318.36 1321.57 1326.17
1327.30 1333.49 1335.76 1342.88 1344.94 1345.74
1378.04 1403.88 1411.99 1424.55 1428.06 1430.08
1439.64 1446.05 1455.27 1463.51 1469.55 1470.28
1475.09 1475.95 1478.30 1479.25 1483.86 1490.21
1491.87 1493.66 1495.24 1498.76 1500.48 1500.77
1501.54 1504.10 1505.63 1508.58 1511.94 1512.73
1517.19 1517.62 1521.36 1536.10 1564.41 1572.81
1593.37 1600.82 1614.15 1630.27 1648.87 1659.65
2986.22 3022.99 3024.81 3026.90 3027.25 3033.98
3035.74 3037.45 3045.15 3050.42 3068.20 3078.66
3085.25 3090.59 3095.77 3098.41 3111.49 3114.04
3114.78 3116.48 3117.40 3117.87 3122.32 3126.88
3132.77 3140.34 3141.27 3142.91 3147.17 3149.31
3152.23 3156.96 3158.53 3166.27 3179.79 3186.90
3189.73 3199.78 3204.64 3208.08 3208.67 3212.29
3218.50 3226.91 3248.56

==============================

^{3}Y2

==============================

19.98 21.79 32.79 35.04 41.15 42.93
47.21 54.45 57.60 62.18 67.29 70.72
77.95 90.49 99.90 110.73 115.61 122.44
129.63 136.60 136.87 147.32 151.55 163.13
173.20 182.05 191.82 193.71 199.24 201.07
212.81 218.86 228.40 234.84 243.45 245.62
254.49 267.21 271.39 273.19 278.41 285.30
290.41 295.02 310.69 319.74 334.89 335.30
339.44 363.98 369.81 380.42 386.93 390.17
418.93 421.49 423.11 425.55 441.09 445.66
449.63 459.21 472.11 472.40 477.25 511.29
522.33 531.84 547.09 559.70 585.06 624.03
628.07 656.33 656.49 670.29 675.28 696.54
699.92 706.50 708.20 728.79 728.94 739.51
741.47 746.07 749.40 769.67 771.98 818.66
831.95 838.12 839.44 842.85 844.37 845.13
850.42 873.91 874.15 884.61 888.73 890.04
894.63 899.96 908.14 918.33 928.36 933.07
934.34 943.71 952.78 959.87 963.22 967.33
968.01 971.20 972.39 973.82 979.37 979.99
1012.68 1022.65 1023.29 1024.17 1046.88 1051.04
1057.23 1063.35 1064.54 1080.64 1089.04 1107.50
1126.61 1132.32 1158.54 1161.39 1187.49 1191.55
1199.94 1208.75 1227.61 1230.11 1239.29 1240.98
1243.02 1248.57 1261.97 1267.20 1293.67 1309.78
1311.08 1314.48 1325.29 1329.65 1331.59 1333.16
1335.34 1336.07 1337.33 1342.34 1345.83 1381.06
1407.98 1411.95 1426.35 1427.33 1435.96 1442.86
1443.19 1455.43 1462.27 1467.27 1469.99 1475.53
1478.37 1479.84 1482.27 1486.92 1490.34 1492.83
1496.15 1496.80 1500.34 1503.05 1507.68 1509.52
1511.18 1515.41 1516.51 1518.02 1520.17 1522.64
1525.87 1527.06 1534.33 1536.65 1555.29 1565.39
1586.81 1599.81 1607.54 1624.22 1643.49 1662.22
1971.62 1989.52 2107.74 2890.81 2992.47 3020.65
3024.81 3026.64 3033.40 3036.44 3039.08 3047.11
3048.56 3055.33 3077.29 3082.34 3083.41 3089.12
3098.17 3104.27 3110.06 3117.60 3118.73 3130.49
3130.60 3130.80 3135.97 3142.79 3143.60 3147.08
3147.45 3151.69 3158.45 3160.41 3183.53 3186.52
3188.78 3189.49 3195.88 3206.94 3212.20 3217.33
3218.74 3221.51 3230.04

==============================

^{3}Y2-TS

==============================

-394.02 20.59 29.45 33.87 35.56 42.31
47.24 51.07 54.00 56.21 58.71 70.46
77.29 90.54 97.57 102.47 106.69 112.92

114.69 124.71 128.54 135.00 141.89 151.15
158.12 168.30 174.55 181.88 201.87 208.02
214.45 223.07 232.60 236.24 241.34 246.40
250.86 256.27 261.61 267.58 272.55 286.31
294.37 300.55 304.97 320.13 324.62 326.68
334.87 354.06 364.45 375.38 383.65 389.15
392.84 406.08 421.07 422.59 428.40 430.56
444.77 456.11 464.70 467.99 480.85 491.11
508.23 521.49 536.75 545.39 548.69 555.94
568.39 623.14 631.32 657.06 658.86 673.18
674.05 693.07 699.20 705.76 706.95 720.26
722.21 733.20 740.16 748.64 753.35 774.98
780.87 803.08 818.56 831.98 842.88 844.88
847.47 853.42 858.31 874.87 877.35 887.96
893.51 900.42 900.83 906.30 921.33 927.48
932.69 934.86 948.80 952.92 960.43 963.50
964.31 970.80 971.38 971.71 977.47 980.65
982.18 985.28 1022.50 1022.95 1024.01 1047.95
1049.34 1056.34 1060.70 1063.08 1063.70 1067.97
1075.52 1082.58 1090.91 1116.59 1128.83 1157.46
1166.54 1186.24 1187.45 1196.44 1219.94 1226.27
1243.23 1243.68 1246.94 1269.12 1277.05 1292.69
1304.44 1306.20 1310.81 1313.34 1319.79 1325.58
1326.96 1333.57 1335.22 1338.45 1341.82 1345.38
1374.16 1410.15 1412.23 1424.10 1424.86 1427.44
1429.20 1442.22 1442.82 1452.22 1464.98 1468.32
1472.53 1475.35 1477.93 1478.08 1482.48 1489.57
1493.08 1494.28 1495.14 1498.10 1498.54 1498.63
1498.81 1500.38 1502.25 1508.15 1510.40 1513.29
1517.90 1519.52 1523.70 1534.56 1561.23 1567.10
1583.04 1596.34 1601.99 1610.59 1625.86 1649.32
1658.47 3022.84 3025.60 3029.12 3034.06 3036.02
3037.21 3037.33 3045.67 3046.41 3081.33 3085.90
3090.43 3095.90 3101.50 3108.82 3112.51 3114.38
3116.07 3116.67 3120.72 3122.80 3130.11 3139.94
3142.81 3145.37 3146.07 3152.17 3160.58 3168.54
3169.26 3173.22 3177.86 3184.15 3186.75 3189.10
3200.57 3205.23 3206.22 3207.33 3212.27 3217.16
3220.46 3233.80 3270.08

================================

^{3}Y3

================================

15.71 23.91 30.37 41.99 45.04 46.32
47.12 50.56 62.84 64.44 90.36 94.57
100.57 107.18 112.11 117.74 127.62 140.31
147.18 149.27 163.65 183.50 193.00 201.46
204.76 219.99 226.18 237.26 241.31 242.18
247.08 256.02 261.60 272.32 298.93 301.38
322.05 338.04 354.71 359.72 378.25 395.48
405.15 415.72 417.32 427.32 434.99 454.97
464.97 465.68 479.59 507.37 533.59 544.52
555.94 563.51 593.14 625.03 631.49 657.51
658.67 677.31 678.86 694.83 700.25 701.16
706.54 728.25 733.17 741.11 746.88 751.43
773.00 778.48 819.44 831.90 840.98 843.04
848.59 851.23 854.44 877.94 890.95 895.62
901.76 903.83 912.96 920.74 932.58 936.62
952.36 958.98 962.88 965.16 967.23 973.67
977.49 979.16 984.36 1022.18 1024.68 1030.43
1046.98 1056.40 1063.65 1064.13 1064.46 1081.69
1089.99 1127.91 1159.29 1165.99 1183.88 1188.17
1198.80 1224.92 1242.56 1244.15 1265.29 1292.13
1303.58 1310.50 1312.61 1318.55 1325.28 1327.83
1330.64 1335.79 1339.88 1343.66 1346.61 1380.28
1424.92 1427.59 1428.61 1441.61 1453.24 1464.85
1467.02 1471.03 1473.02 1477.58 1478.30 1487.86
1489.74 1492.53 1497.78 1497.98 1499.93 1501.46
1507.88 1508.99 1511.96 1515.52 1522.60 1555.78
1567.36 1597.33 1600.15 1608.98 1625.27 1647.90
1658.12 3028.21 3031.59 3032.38 3037.72 3040.46
3050.80 3085.76 3088.23 3114.19 3115.89 3116.48
3117.05 3127.40 3134.55 3135.13 3140.04 3145.14
3152.35 3158.84 3162.95 3170.61 3174.04 3186.74
3189.26 3193.23 3202.64 3203.40 3205.78 3211.70
3216.55 3220.92 3230.64

================================

^{3}Y4

================================

20.72 23.88 33.58 35.58 39.48 45.06
49.81 53.86 54.79 57.98 67.59 72.17
78.93 88.33 95.16 105.44 117.98 119.48
124.18 129.66 135.07 136.56 143.82 150.14
160.94 166.93 176.60 176.92 189.87 192.11
212.75 218.58 223.96 228.38 234.18 238.67
242.99 247.84 249.05 268.48 274.03 285.18
287.10 299.58 312.17 325.13 331.32 348.20
361.19 367.43 369.49 376.83 405.21 417.53
419.68 427.01 429.80 437.56 444.34 446.12
453.28 465.57 469.35 476.38 483.39 512.63
537.42 541.29 553.28 555.30 570.61 620.10
622.68 626.24 653.68 656.63 657.46 661.21
662.59 671.09 679.24 691.39 699.69 700.62
711.22 715.58 728.60 731.69 735.31 738.39
748.20 751.70 757.60 768.21 772.27 776.23
790.48 812.67 835.05 839.66 841.81 844.27
844.98 856.57 857.57 863.84 873.46 888.12
891.98 898.13 903.30 906.84 922.77 933.04
934.74 953.78 956.66 960.60 964.26 965.87
966.65 970.97 976.48 977.83 978.80 979.67
981.33 983.39 988.41 1001.37 1020.99 1023.52
1023.72 1036.34 1047.12 1057.92 1059.01 1062.90
1063.67 1065.68 1075.07 1079.36 1081.52 1090.51
1126.80 1131.02 1157.15 1158.90 1170.89 1184.51
1186.70 1187.46 1195.59 1200.73 1226.16 1244.03
1245.41 1259.34 1276.69 1297.68 1305.35 1307.02
1311.26 1311.94 1321.95 1324.71 1325.50 1329.81
1330.17 1331.61 1335.19 1336.63 1341.33 1343.82
1367.24 1379.71 1424.78 1427.63 1429.79 1442.22
1444.15 1453.40 1463.06 1465.54 1468.27 1470.77
1476.01 1477.80 1479.22 1481.91 1486.47 1490.54
1497.18 1497.85 1499.36 1500.73 1502.82 1506.97
1509.95 1511.69 1513.78 1522.39 1550.81 1563.23

1567.95 1579.56 1593.04 1595.43 1603.02 1613.75
1625.87 1632.48 1643.45 1646.44 1656.61 3025.51
3026.13 3034.41 3036.27 3041.61 3051.21 3081.20
3084.34 3111.26 3113.43 3114.27 3115.07 3131.67
3133.73 3142.60 3145.69 3154.51 3157.69 3161.74
3162.84 3168.84 3183.29 3184.39 3186.25 3187.74
3189.59 3199.18 3203.41 3204.76 3205.54 3207.62
3211.40 3213.41 3217.17 3217.20 3219.82 3230.05
3248.87 3250.65 3280.37

==============================

^{3}Y4-TS

==============================

-830.59 12.53 30.09 32.42 36.50 39.24
42.39 47.10 52.91 54.52 61.29 65.21
69.33 81.25 85.44 97.18 103.84 109.07
112.76 115.97 121.63 131.91 136.47 140.65
148.57 156.13 158.40 164.35 172.16 181.88
185.83 199.43 207.41 211.31 217.29 219.57
222.73 239.27 247.14 252.71 258.14 262.43
267.82 275.94 301.04 317.09 321.98 322.82
350.93 359.24 360.35 366.10 374.72 404.58
410.91 414.58 418.62 422.09 431.89 440.37
442.68 448.47 465.53 469.34 483.62 511.34
514.83 536.99 540.01 549.62 553.44 572.55
622.08 627.47 629.93 639.77 656.15 656.81
664.59 666.85 679.39 692.70 694.17 696.31
698.10 709.32 727.46 729.03 732.88 737.17
745.53 749.57 764.63 768.19 769.46 777.78
794.90 812.70 816.90 832.32 834.65 839.95
840.09 843.19 853.89 873.52 891.95 892.90
896.52 902.00 902.60 904.83 907.23 922.94
932.35 934.06 953.17 962.44 966.35 968.70
972.32 977.63 977.82 979.38 981.75 982.83
987.63 993.33 1006.99 1013.45 1023.36 1024.28
1024.99 1043.22 1046.69 1057.12 1063.69 1063.83
1065.86 1075.33 1080.05 1090.06 1090.42 1102.69
1124.62 1136.17 1156.80 1170.89 1177.00 1184.14
1187.15 1189.85 1197.35 1202.43 1225.81 1243.13
1244.06 1255.33 1269.02 1294.41 1304.71 1310.08
1312.99 1315.36 1318.48 1323.69 1325.41 1327.16
1327.76 1328.70 1332.49 1336.98 1340.26 1342.68
1346.84 1372.32 1382.44 1425.53 1427.17 1430.32
1442.04 1453.37 1454.70 1466.39 1467.70 1470.06
1470.70 1475.94 1477.19 1478.19 1484.76 1490.11
1493.51 1498.63 1500.46 1500.97 1504.02 1506.78
1509.95 1511.09 1512.52 1516.41 1529.36 1547.98
1561.77 1573.78 1589.20 1591.33 1603.57 1616.08
1629.02 1635.71 1649.20 1650.90 1657.90 3024.95
3027.53 3033.77 3035.73 3041.72 3051.27 3079.80
3086.26 3109.64 3115.23 3115.56 3118.45 3128.90
3129.91 3132.03 3140.80 3143.74 3147.61 3150.87
3160.03 3161.39 3161.93 3167.46 3175.55 3179.35
3185.09 3188.17 3202.12 3202.91 3205.78 3207.02
3207.80 3209.25 3211.53 3213.87 3216.02 3223.92
3225.91 3230.52 3231.23

==============================

Y5

==============================

18.90 24.72 28.13 34.36 35.35 41.45
46.96 48.60 55.58 58.34 65.30 72.19
81.22 86.65 91.30 99.26 102.87 113.68
114.86 119.12 121.44 142.40 148.77 151.15
153.21 156.64 163.88 169.39 171.52 185.31
193.63 199.69 207.80 213.59 217.60 232.48
234.79 244.04 247.12 247.95 261.93 266.84
277.08 298.55 304.40 317.12 323.64 334.79
343.31 359.66 364.67 370.10 386.45 409.60
413.93 419.99 423.01 429.82 442.22 444.00
454.82 468.34 472.90 475.72 482.25 511.44
519.68 536.79 548.77 553.29 566.25 600.52
619.24 623.54 648.33 655.09 656.83 658.97
660.57 668.13 677.32 684.85 694.88 697.29
699.15 706.87 725.79 731.26 738.61 740.41
748.30 751.81 767.21 770.70 773.95 774.29
802.32 818.73 833.36 839.63 842.77 844.96
849.22 850.15 876.59 891.77 893.08 895.53
904.19 906.52 907.54 912.44 923.52 932.56
934.56 951.89 960.65 962.82 969.46 974.13
976.12 976.81 979.33 980.06 980.93 986.27
996.80 999.32 1008.77 1017.70 1023.50 1025.02
1031.73 1046.72 1055.75 1057.57 1063.38 1063.44
1063.90 1068.46 1081.96 1088.19 1092.05 1121.18
1133.51 1158.39 1174.72 1181.01 1184.24 1186.30
1196.06 1197.91 1211.09 1231.57 1243.30 1245.43
1257.13 1267.29 1296.11 1303.05 1304.90 1314.59
1314.79 1322.03 1325.63 1327.83 1328.91 1329.13
1330.12 1335.29 1336.95 1342.43 1348.74 1349.83
1382.80 1425.45 1427.26 1427.74 1430.03 1442.71
1446.96 1452.35 1459.99 1468.43 1472.15 1475.08
1477.03 1478.47 1485.30 1486.75 1487.28 1491.18
1496.15 1498.73 1501.85 1502.03 1505.74 1510.75
1513.84 1514.60 1519.29 1533.93 1554.98 1562.74
1573.04 1593.59 1603.56 1604.92 1605.16 1628.55
1649.64 1651.20 1658.58 1693.64 2951.17 2957.94
3025.53 3029.92 3032.49 3037.99 3042.10 3050.75
3080.28 3088.76 3111.16 3114.60 3116.31 3117.00
3128.53 3132.05 3133.44 3146.59 3148.28 3149.73
3156.16 3158.59 3161.21 3164.11 3166.38 3183.36
3184.85 3187.67 3190.80 3198.13 3200.53 3202.09
3206.98 3207.37 3211.48 3216.86 3221.56 3225.81
3229.20 3237.66 3240.98

==============================

N1

==============================

23.22 30.81 36.31 38.05 50.23 53.30
54.33 58.63 61.98 69.35 74.78 92.59
99.27 101.32 113.59 114.57 123.01 125.43
137.57 143.91 148.72 154.94 164.36 168.82
170.59 175.33 192.62 202.70 203.26 214.94
217.07 222.03 237.21 245.71 247.27 252.93
257.21 269.45 280.10 287.48 292.61 304.19
316.93 322.90 327.96 345.75 357.31 372.66

376.14	385.30	392.77	405.86	415.35	425.39
432.51	445.39	454.83	473.91	481.65	489.01
499.24	526.60	528.53	543.37	553.11	567.61
625.04	626.84	636.38	648.75	669.28	674.54
681.51	702.75	712.20	716.67	725.32	727.35
733.45	750.41	769.83	801.67	829.55	833.52
848.56	849.07	851.23	864.68	886.76	887.98
894.09	897.02	898.59	907.35	918.19	929.91
931.40	934.78	936.18	966.90	972.39	972.42
975.54	978.57	986.48	998.69	1006.78	1016.23
1019.27	1022.05	1024.97	1049.16	1057.16	1062.61
1063.91	1065.52	1080.90	1087.94	1097.12	1098.31
1174.64	1180.44	1184.69	1189.42	1191.66	1223.37
1232.67	1241.67	1247.03	1255.57	1281.61	1292.68
1305.21	1316.72	1320.12	1321.50	1325.52	1326.31
1328.77	1335.85	1340.78	1342.74	1392.05	1401.18
1408.83	1426.41	1427.95	1435.47	1437.66	1443.49
1456.84	1465.94	1468.82	1470.20	1472.68	1473.58
1480.05	1481.73	1482.80	1487.04	1488.00	1490.63
1497.75	1499.06	1499.79	1500.44	1508.31	1509.17
1509.85	1514.01	1517.73	1523.03	1527.37	1533.39
1584.87	1595.03	1630.60	1648.84	1653.26	1659.89
2824.16	2949.46	3016.37	3020.54	3026.21	3026.63
3031.85	3043.93	3045.59	3050.29	3050.54	3052.53
3079.76	3082.92	3083.75	3089.28	3094.82	3098.11
3106.82	3110.64	3111.95	3112.56	3126.86	3130.60
3130.88	3137.98	3150.61	3151.59	3153.00	3153.77
3154.54	3158.79	3161.64	3164.80	3171.51	3186.56
3202.11	3202.71	3209.71	3209.71	3223.67	3248.70

==============================

^{3}N1-TS

==============================

-435.87	22.27	27.20	32.13	42.38	43.45
44.86	53.32	61.16	62.55	68.74	75.07
83.65	94.86	108.65	118.02	124.05	132.52
136.17	144.73	147.63	150.32	156.64	159.56
161.68	169.20	179.96	194.18	197.93	201.21
214.92	225.39	228.04	235.84	244.95	246.84
249.47	261.35	272.80	275.60	286.15	289.14
307.87	310.10	321.43	328.31	349.14	355.70
369.42	390.88	396.11	402.94	405.78	413.30
437.60	444.08	460.31	464.39	471.77	475.08
484.74	528.41	550.63	565.22	577.71	614.06
625.08	626.86	663.61	668.28	671.88	690.09
698.63	699.88	704.86	714.55	721.72	727.47
748.58	752.91	773.65	819.93	831.07	840.35
844.97	845.96	850.55	863.72	871.25	876.36
886.82	888.63	900.95	904.77	906.95	916.28
918.46	926.94	931.07	938.12	954.61	967.48
971.25	973.55	975.84	976.88	977.04	989.85
990.16	1021.53	1023.21	1040.17	1043.97	1053.63
1063.81	1064.24	1076.71	1077.06	1085.54	1098.76
1160.53	1175.78	1177.69	1181.27	1186.02	1215.87
1223.95	1239.03	1242.42	1243.61	1272.60	1293.40
1305.63	1307.15	1314.75	1316.09	1324.28	1324.75
1325.95	1334.79	1340.61	1341.55	1396.59	1402.65
1408.97	1425.93	1427.40	1434.62	1436.10	1440.78
1467.80	1468.90	1469.70	1470.75	1472.83	1475.21
1478.75	1482.02	1485.41	1485.79	1486.82	1492.23
1498.51	1498.53	1499.26	1501.45	1508.12	1509.36
1510.07	1513.36	1518.27	1519.41	1520.04	1533.54
1579.25	1585.47	1597.18	1625.75	1650.63	1655.73
2967.89	2994.40	3018.54	3024.11	3025.60	3027.30
3034.21	3043.02	3044.92	3048.42	3048.83	3079.38
3082.48	3083.56	3084.92	3089.98	3101.15	3111.57
3111.95	3115.76	3120.90	3127.31	3128.02	3128.70
3128.80	3130.96	3146.46	3151.92	3152.44	3154.85
3155.73	3155.81	3160.18	3160.91	3166.32	3189.35
3198.35	3198.49	3202.42	3211.16	3220.47	3231.92

==============================

P1

==============================

26.48	39.43	49.26	51.62	55.29	56.84
57.71	66.71	79.09	81.87	85.67	99.17
100.32	102.77	107.64	116.63	121.38	128.72
134.36	137.91	149.55	153.15	158.17	159.55
163.85	169.50	174.09	176.05	187.03	195.88
205.71	214.14	228.42	231.60	234.05	245.11
246.70	248.83	255.41	260.64	267.26	271.12
275.33	281.46	290.36	294.70	305.01	311.64
316.25	329.86	335.60	347.08	353.54	373.66
376.36	389.16	405.15	411.57	423.14	446.89
459.75	469.67	478.44	489.57	504.17	527.34
530.24	539.27	552.16	567.18	626.43	638.65
650.29	652.37	672.73	681.28	698.30	706.77
708.41	714.60	721.00	725.69	732.33	749.00
799.81	811.87	831.01	832.52	845.42	847.14
848.68	857.85	860.23	865.74	884.23	885.86
890.30	896.89	904.09	918.92	927.49	927.57
933.54	935.84	966.79	968.62	969.49	971.57
973.38	976.37	983.24	988.00	991.41	1021.42
1025.08	1048.06	1062.53	1062.80	1063.07	1080.19
1087.41	1172.38	1182.73	1186.89	1189.70	1220.08
1234.12	1242.27	1246.26	1282.33	1294.30	1313.89
1317.56	1319.07	1322.02	1324.05	1327.01	1331.09
1333.22	1339.57	1341.73	1342.77	1355.57	1401.25
1408.57	1426.47	1427.77	1435.74	1437.60	1443.34
1444.05	1465.69	1466.43	1468.27	1470.78	1471.17
1473.14	1475.81	1478.55	1480.77	1483.01	1485.06
1485.69	1489.90	1491.85	1492.89	1497.68	1498.93
1499.52	1501.04	1502.48	1507.39	1509.30	1510.32
1513.99	1519.81	1522.00	1533.66	1586.40	1594.85
1651.38	1659.14	2827.25	2958.96	3021.47	3025.37
3027.07	3027.57	3035.84	3036.32	3044.32	3044.83
3046.57	3048.03	3052.18	3052.29	3057.09	3081.23
3083.88	3085.83	3086.90	3092.44	3107.16	3112.61
3112.83	3116.41	3120.04	3124.07	3126.98	3129.85
3132.17	3134.93	3135.13	3137.44	3142.25	3143.05
3152.38	3157.92	3160.32	3160.51	3162.43	3162.84
3163.01	3167.98	3179.21	3197.49	3201.94	3206.77

==============================

^{3}P1-TS

==============================

-365.25 22.03 33.94 38.67 51.52 55.34
57.89 58.29 60.89 69.32 75.58 84.00
92.38 95.78 102.09 109.70 110.67 123.86
129.40 133.21 135.49 151.10 152.77 155.71
162.42 168.69 176.49 190.43 192.34 194.11
197.79 204.88 208.69 223.84 230.83 231.40
241.70 248.94 253.81 255.03 256.59 258.99
263.32 275.33 282.94 287.91 295.77 309.20
317.36 319.02 325.20 328.88 350.10 363.71
370.61 385.66 400.41 410.09 414.66 443.48
462.92 469.01 470.49 474.16 486.67 529.69
548.97 553.70 565.32 622.97 627.17 652.29
667.86 670.64 688.06 697.93 703.29 711.71
714.39 718.89 720.28 728.44 749.17 781.90
807.06 822.22 833.38 840.81 842.20 845.80
856.82 861.54 863.87 876.40 883.96 887.59
889.33 899.13 901.40 917.35 924.65 929.91
931.50 937.18 960.24 965.20 969.61 970.85
972.73 975.35 976.24 978.14 987.67 1020.89
1023.42 1040.55 1046.83 1062.25 1063.73 1079.71
1087.70 1160.37 1181.16 1184.76 1185.88 1218.19
1232.01 1241.22 1244.60 1270.70 1293.23 1308.32
1315.03 1317.68 1321.52 1323.33 1325.69 1331.07
1331.58 1340.70 1341.40 1341.75 1352.75 1401.12
1408.67 1425.75 1427.33 1435.02 1435.38 1441.90
1461.81 1466.42 1468.08 1470.97 1471.71 1472.67
1474.05 1476.07 1476.67 1480.98 1482.19 1483.79
1484.48 1487.86 1490.04 1493.13 1496.46 1498.21
1499.26 1501.17 1504.54 1507.86 1509.36 1510.39
1513.34 1521.60 1523.22 1537.01 1584.50 1594.03
1649.36 1657.95 2963.95 3002.97 3019.71 3025.40
3026.15 3026.38 3035.18 3037.42 3039.23 3042.02
3047.99 3048.71 3049.78 3050.73 3078.93 3079.92
3086.50 3091.52 3100.61 3103.02 3110.64 3111.19
3111.98 3122.49 3123.97 3126.72 3127.16 3128.44
3129.46 3131.88 3138.64 3139.86 3140.83 3141.20
3148.06 3153.35 3154.98 3156.20 3157.63 3158.92
3159.95 3163.75 3164.19 3168.29 3197.00 3201.31

==============================

X3

==============================

15.58 23.11 34.65 41.36 51.65 52.36
56.44 57.64 59.56 63.15 77.33 79.31
86.24 90.26 96.49 105.76 112.12 122.59
129.64 141.49 149.08 150.64 154.21 160.55
169.36 174.12 186.82 191.55 198.63 205.90
218.85 222.21 226.90 235.34 244.86 251.71
258.51 265.73 268.18 274.22 280.63 285.12
290.83 293.95 299.77 307.86 322.08 325.25
334.87 338.65 345.82 348.84 359.43 377.07
383.49 394.52 400.74 413.85 415.69 441.34
449.51 457.74 464.24 466.80 472.11 475.63
495.75 501.47 509.09 532.72 537.66 551.44
556.17 565.65 567.76 588.77 601.14 629.30
632.37 653.97 668.17 673.40 682.24 705.65
709.96 723.62 728.79 732.21 733.21 735.18
736.76 748.98 769.92 771.71 778.32 828.62
832.19 835.11 847.76 851.68 854.17 869.29
876.64 878.17 881.17 883.49 891.85 897.74
899.51 903.49 907.62 913.50 923.62 925.65
934.64 935.87 943.65 943.82 945.59 946.78
964.87 971.05 972.04 977.48 982.00 983.13
984.44 1008.10 1023.28 1025.43 1032.01 1048.90
1060.18 1063.91 1064.13 1064.36 1082.81 1092.82
1102.96 1104.91 1117.49 1135.64 1162.30 1179.71
1185.78 1186.25 1189.60 1224.53 1231.42 1232.48
1235.02 1243.79 1245.37 1248.12 1263.54 1279.48
1296.48 1298.53 1313.86 1319.03 1321.11 1322.07
1326.64 1329.81 1332.21 1337.56 1341.63 1342.93
1348.67 1359.02 1368.91 1410.65 1414.74 1423.48
1428.04 1428.55 1437.18 1444.99 1445.45 1468.46
1469.62 1472.62 1472.98 1478.05 1482.52 1487.25
1487.84 1491.31 1496.49 1498.24 1498.67 1498.75
1500.31 1505.14 1508.30 1508.61 1509.25 1509.87
1513.33 1518.40 1520.95 1524.63 1530.24 1530.40
1540.96 1597.20 1606.00 1620.89 1640.08 1644.78
1652.66 1657.70 1671.82 3014.22 3022.40 3023.39
3025.48 3030.41 3032.29 3033.74 3044.97 3051.14
3053.49 3057.78 3081.39 3083.73 3086.07 3087.29
3089.30 3093.80 3107.24 3117.27 3118.89 3119.30
3124.09 3131.34 3133.71 3134.67 3147.61 3152.36
3155.46 3156.09 3164.68 3165.96 3166.65 3168.21
3168.83 3172.87 3179.46 3179.93 3194.52 3194.93
3202.67 3203.96 3207.47 3207.82 3219.57 3228.87

==============================

X3-TS

==============================

-1293.95 23.24 26.90 29.14 41.02 49.49
54.86 56.38 63.41 67.14 70.05 75.11
79.75 86.87 90.38 100.13 112.68 120.67
125.71 137.72 149.96 151.29 152.72 159.21
163.02 166.99 171.40 191.49 196.45 204.63
206.84 221.65 226.03 230.00 236.24 246.97
254.30 256.84 265.21 278.15 281.80 286.52
292.05 302.30 311.47 317.52 319.89 323.42
328.90 345.38 348.51 349.99 374.81 390.21
396.03 402.22 412.65 415.77 436.31 445.83
449.02 460.32 464.29 469.58 472.54 489.65
492.81 500.03 524.27 540.56 548.36 554.61
561.69 569.67 574.93 586.18 598.64 627.26
632.36 657.81 673.10 678.87 681.48 705.41
710.19 727.82 729.13 729.93 731.39 732.24
740.59 746.94 772.65 774.04 777.21 807.66
822.33 832.66 838.62 848.32 851.00 851.69
871.12 877.43 880.66 885.05 887.51 890.52
893.20 893.70 903.07 905.35 905.51 925.49
927.47 934.30 937.46 939.02 948.15 948.88
954.78 964.53 967.52 978.62 979.70 979.86
982.20 984.43 1021.80 1024.72 1033.26 1035.22
1059.40 1062.86 1063.21 1064.02 1064.92 1082.60
1092.48 1097.29 1107.33 1120.01 1159.61 1175.42

1184.86 1185.02 1187.48 1189.98 1226.16 1231.65
1232.97 1234.59 1243.18 1244.74 1246.20 1268.17
1276.72 1280.11 1288.07 1296.65 1311.62 1313.45
1319.15 1323.10 1324.67 1326.67 1330.08 1336.40
1341.25 1345.24 1358.88 1368.56 1376.97 1409.27
1416.09 1427.34 1427.92 1438.45 1444.00 1444.40
1469.75 1470.56 1472.27 1472.29 1480.24 1480.74
1485.21 1486.99 1489.23 1491.26 1496.02 1496.27
1498.89 1500.23 1500.93 1507.60 1508.41 1509.98
1511.59 1512.19 1518.03 1520.56 1524.20 1531.51
1538.25 1594.75 1604.17 1624.95 1646.54 1648.21
1651.51 1656.83 1673.09 1848.42 3021.40 3027.41
3030.03 3030.79 3042.05 3047.98 3048.99 3050.67
3057.95 3068.65 3085.11 3085.31 3090.19 3097.27
3102.09 3106.70 3115.87 3116.03 3119.32 3125.26
3125.39 3131.59 3133.50 3141.47 3152.53 3155.95
3158.23 3163.00 3163.70 3165.01 3168.01 3170.62
3174.06 3180.76 3182.08 3194.55 3194.69 3197.29
3199.34 3203.57 3206.63 3209.34 3211.76 3227.86

==============================

X4

==============================

20.08 22.16 26.30 29.50 32.82 41.45
48.33 49.63 54.87 58.74 62.46 70.54
84.85 87.37 92.41 106.30 110.88 113.72
117.69 123.48 138.20 143.39 147.53 151.61
157.36 166.35 175.77 182.48 196.71 200.27
211.68 218.30 228.08 233.98 238.31 242.91
246.63 253.64 256.02 259.71 276.95 279.80
287.95 293.47 301.02 306.45 316.35 323.56
325.76 331.27 345.89 358.43 373.72 379.43
384.80 400.58 411.69 412.37 438.38 439.74
446.64 451.36 457.82 465.64 471.80 490.75
498.12 520.90 535.59 542.28 545.70 551.28
557.98 564.75 583.11 626.43 629.11 633.16
660.04 663.79 673.95 676.14 702.18 706.91
709.77 720.70 723.60 729.27 730.89 735.76
744.58 748.65 774.36 777.09 823.35 827.04
832.91 845.62 846.42 849.58 852.88 856.51
866.80 871.78 880.60 884.42 892.71 894.55
896.05 898.98 901.28 902.55 907.88 923.70
925.10 931.82 933.20 935.26 945.57 956.44
962.23 963.83 968.68 969.64 975.64 981.84
983.44 987.11 1022.46 1024.58 1033.94 1057.20
1062.08 1063.79 1064.24 1067.64 1080.57 1089.82
1101.42 1107.14 1122.68 1158.80 1173.86 1186.51
1187.43 1189.59 1215.86 1216.54 1222.40 1225.53
1233.64 1242.96 1243.86 1247.56 1249.56 1272.37
1274.62 1278.00 1296.12 1305.19 1313.26 1319.44
1326.98 1329.60 1330.32 1332.67 1333.49 1336.02
1344.85 1346.87 1359.20 1375.96 1377.97 1407.86
1413.03 1427.63 1428.42 1436.74 1441.86 1443.54
1465.17 1467.66 1470.51 1471.28 1478.25 1479.04
1481.92 1482.83 1487.37 1491.65 1495.37 1497.78
1498.50 1499.61 1501.97 1503.63 1508.20 1509.58
1510.75 1517.76 1518.22 1520.32 1525.18 1533.71
1534.78 1591.44 1600.52 1628.59 1650.71 1651.36
1656.20 1656.81 1678.23 2910.03 3022.51 3027.74
3029.01 3031.82 3036.61 3046.25 3046.43 3049.13
3053.48 3073.70 3084.12 3084.20 3088.45 3089.25
3092.97 3098.50 3109.49 3112.36 3115.52 3117.63
3128.13 3132.32 3134.27 3134.59 3147.30 3153.76
3154.31 3155.52 3157.30 3163.85 3164.06 3168.84
3170.16 3175.26 3181.71 3182.46 3194.67 3199.15
3199.19 3201.05 3204.90 3211.51 3213.00 3246.47

==============================

X5

==============================

19.44 26.59 29.16 33.20 39.92 42.94
50.21 51.76 53.08 55.72 58.95 64.84
71.87 74.23 78.67 79.49 86.05 92.07
97.68 103.27 106.91 113.04 123.08 131.63
134.39 140.79 149.32 153.84 157.87 161.85
164.19 169.27 176.01 179.54 182.40 188.25
193.31 211.49 215.67 221.38 227.06 236.32
240.88 241.48 246.03 251.43 258.41 259.05
261.41 267.30 274.99 290.21 292.59 293.65
298.63 304.84 311.09 320.13 331.00 332.41
335.20 337.01 343.64 345.83 357.95 365.04
378.75 383.65 387.77 394.85 406.80 414.66
427.21 434.74 442.01 445.54 448.20 450.45
458.79 471.04 475.55 479.12 484.48 492.56
524.58 526.45 530.02 543.10 545.94 553.06
554.23 557.12 557.98 566.44 583.90 598.82
624.76 625.20 631.25 634.39 658.95 663.35
672.97 674.75 681.64 690.30 700.64 706.11
708.20 713.23 716.03 719.85 724.29 728.32
734.58 738.37 742.79 750.08 772.84 775.64
782.69 783.52 808.33 819.08 822.46 830.76
846.42 848.80 853.92 857.83 860.65 862.63
864.04 868.73 879.34 880.79 884.37 884.97
887.61 892.44 896.00 898.25 899.95 901.15
901.39 902.51 908.11 923.75 931.32 932.81
935.52 944.39 946.53 960.76 965.12 967.13
970.97 972.68 974.93 979.76 981.47 986.10
987.41 1001.41 1003.05 1005.15 1022.17 1024.98
1038.26 1056.89 1058.73 1062.00 1062.98 1064.23
1064.51 1067.16 1082.11 1089.02 1104.68 1117.83
1120.69 1137.16 1146.86 1164.86 1187.03 1187.65
1189.11 1189.51 1190.55 1192.63 1214.26 1216.66
1216.84 1224.21 1227.09 1234.09 1244.22 1248.01
1248.81 1251.66 1253.24 1263.55 1272.71 1279.08
1283.49 1284.45 1292.12 1308.53 1314.02 1316.56
1320.58 1323.87 1326.05 1328.90 1332.13 1337.09
1341.46 1343.98 1349.97 1360.88 1378.50 1379.97
1390.37 1391.08 1395.93 1404.90 1407.58 1425.17
1426.99 1434.88 1437.88 1444.73 1469.18 1470.82
1472.60 1474.35 1481.45 1483.56 1485.90 1490.84
1491.96 1492.94 1496.41 1496.76 1497.23 1500.51
1500.94 1502.71 1503.41 1507.03 1507.93 1509.60
1509.86 1516.97 1518.57 1519.97 1523.45 1524.76
1527.83 1536.13 1541.26 1580.22 1589.33 1614.51

1615.19 1630.34 1648.36 1652.40 1653.76 1657.79
1662.22 1662.81 1679.72 1683.71 2953.05 3017.48
3023.04 3023.57 3028.58 3042.34 3050.54 3051.44
3052.31 3056.58 3075.66 3077.86 3083.65 3097.18
3102.72 3105.00 3105.89 3109.05 3109.87 3112.90
3129.00 3131.38 3133.22 3134.30 3140.34 3149.57
3153.52 3157.49 3157.53 3167.72 3169.97 3171.57
3175.41 3179.95 3180.64 3185.70 3185.95 3190.51
3191.38 3196.33 3201.61 3205.57 3205.60 3210.13
3210.78 3212.38 3213.00 3219.29 3220.01 3229.53
3257.72 3268.69 3287.68

===============================

^{3}X6

===============================

12.07 25.37 27.26 31.68 34.67 36.01
42.25 44.39 47.76 56.04 68.14 69.05
70.03 72.44 75.22 82.63 93.43 101.87
107.15 112.42 118.71 129.74 131.47 132.95
143.21 150.28 151.23 164.85 169.23 191.08
194.41 196.75 199.32 208.21 220.39 229.64
235.67 237.40 243.02 249.32 256.40 261.52
265.52 266.83 275.31 282.40 285.94 294.98
315.62 322.68 331.11 335.76 352.68 354.29
367.47 375.11 390.58 398.43 407.04 413.33
416.17 419.29 448.96 449.70 454.04 467.03
468.10 471.08 476.78 484.18 529.80 531.37
534.66 539.57 541.21 553.37 566.18 581.00
591.67 595.51 603.03 621.78 625.04 626.97
628.19 661.40 670.62 674.43 676.44 677.86
697.19 701.67 709.03 712.76 714.62 727.03
729.33 729.52 738.44 739.61 747.77 763.28
764.05 765.68 772.77 801.74 831.80 835.29
845.67 847.34 850.84 853.72 854.31 858.62
862.73 866.98 869.11 874.76 877.98 886.12
890.68 894.38 896.21 901.08 905.69 909.47
920.60 928.74 932.85 935.12 941.10 943.50
954.67 960.12 964.40 974.04 976.18 977.90
979.20 979.65 981.55 982.92 1023.24 1025.78
1053.55 1055.92 1061.57 1062.05 1064.46 1064.54
1080.31 1088.26 1093.13 1122.96 1124.40 1133.03
1151.38 1179.88 1182.19 1184.00 1188.08 1189.54
1190.38 1197.88 1212.34 1223.44 1224.87 1231.14
1242.23 1243.10 1245.67 1246.93 1252.88 1292.94
1295.53 1298.28 1305.14 1310.02 1315.09 1320.36
1321.49 1328.74 1330.09 1339.36 1343.06 1345.05
1346.52 1348.24 1350.56 1373.24 1374.14 1375.13
1375.93 1415.96 1425.53 1428.11 1436.10 1444.62
1469.27 1470.55 1472.93 1473.73 1476.16 1478.22
1484.21 1484.48 1488.71 1491.89 1496.85 1497.34
1497.48 1501.16 1502.03 1505.43 1507.82 1509.63
1520.12 1522.49 1530.82 1535.24 1586.73 1591.20
1599.87 1609.10 1619.15 1629.22 1650.84 1652.99
1653.83 1659.08 1660.51 1678.30 2960.05 3025.13
3026.54 3045.97 3051.91 3052.59 3054.07 3080.17
3083.81 3111.99 3113.00 3132.76 3133.49 3135.19
3143.19 3146.19 3155.93 3158.96 3161.04 3161.49
3162.83 3172.50 3179.13 3183.08 3184.62 3186.46
3188.32 3189.08 3197.89 3197.96 3198.05 3204.78
3205.41 3207.38 3208.03 3211.70 3212.84 3212.92
3216.37 3222.77 3240.33

===============================

X7

===============================

17.20 24.28 28.10 40.59 43.09 46.97
54.69 56.95 63.87 67.93 69.81 72.02
77.00 83.31 89.95 90.40 98.19 106.72
108.95 115.40 129.88 132.68 138.25 143.81
146.83 158.36 165.29 168.45 169.96 181.76
193.34 196.99 210.69 215.95 220.76 229.21
234.07 240.98 247.71 253.11 256.27 265.59
271.66 275.56 287.39 298.69 301.60 316.88
321.70 324.08 326.59 351.22 362.21 369.68
379.15 392.65 400.14 415.38 417.94 436.62
443.75 444.35 452.08 453.43 460.69 465.18
468.85 475.01 483.86 500.17 532.62 540.59
542.85 552.25 554.87 556.53 566.69 576.88
577.93 589.15 612.55 625.92 628.38 630.73
652.58 656.16 660.87 669.89 675.16 690.87
697.46 702.44 712.88 721.49 726.34 732.18
733.14 735.94 737.16 747.44 758.96 766.24
778.52 778.85 780.68 817.11 826.05 830.37
834.52 845.70 848.51 855.86 859.44 860.73
865.37 869.67 874.26 880.07 881.55 882.76
892.79 894.13 896.59 901.25 904.72 906.88
908.45 925.97 935.39 936.42 938.63 941.86
961.46 964.67 965.87 966.27 970.70 976.48
981.35 987.87 988.65 992.34 1024.39 1027.35
1057.54 1062.14 1062.23 1064.29 1064.60 1065.19
1074.12 1085.63 1106.66 1107.25 1118.83 1120.78
1158.59 1164.88 1179.96 1186.37 1186.79 1187.63
1189.11 1191.31 1213.60 1219.19 1223.86 1227.00
1239.95 1240.95 1243.13 1247.32 1267.61 1279.41
1287.08 1297.68 1298.93 1303.29 1306.40 1320.03
1321.13 1324.13 1327.73 1329.49 1330.34 1336.31
1336.63 1341.58 1347.80 1353.58 1358.26 1360.39
1369.04 1376.52 1423.15 1428.60 1439.95 1447.48
1461.65 1466.34 1470.57 1474.75 1479.96 1481.06
1484.75 1487.63 1490.48 1490.84 1499.88 1500.09
1500.33 1500.95 1506.11 1508.69 1509.32 1511.67
1514.95 1516.99 1531.39 1533.95 1592.70 1607.37
1619.64 1627.85 1641.56 1643.74 1649.70 1653.23
1655.06 1658.09 1672.02 1676.98 2952.19 3027.84
3028.96 3051.22 3051.72 3053.03 3053.91 3085.92
3087.39 3114.64 3116.88 3134.05 3137.37 3145.32
3146.16 3154.10 3154.28 3161.31 3164.54 3165.14
3166.59 3173.90 3180.01 3180.14 3183.93 3185.09
3186.69 3194.52 3195.46 3200.07 3203.42 3204.62
3205.90 3207.55 3207.93 3212.26 3212.68 3212.82
3215.10 3261.02 3280.48

References

(1) B. C. Bailey, H. Fan, E. W. Baum, J. C. Huffman, M.-H. Baik and D. J. Mindiola, *J. Am. Chem. Soc.* 2005, **127**, 16016.

(2) J. A. Flores, V. N. Cavaliere, D. Buck, B. Pinter, G. Chen, M. G. Crestani, M.-H. Baik and D. J. Mindiola, *Chem. Sci.* 2011, **2**, 1457.

(3) *SAINT*; Bruker AXS Inc.: Madison, WI, USA. 2009.

(4) G. M. Sheldrick, *SADABS*; University of Gottingen: Germany, 2007.

(5) (a) G. M. Sheldrick, *Acta Crystallogr.* 2008, **A64**, 112; (b) G. M. Sheldrick, *Acta Crystallogr.* 2015, **C71**, 3.

(6) $R_1 = \Sigma||F_o| - |F_c|| / \Sigma |F_o|$, $wR_2 = [\Sigma w(F_o^2 - F_c^2)^2/\Sigma w(F_o^2)^2]^{½}$, $GOF = [\Sigma w(F_o^2 - F_c^2)^2/(n - p)]^{½}$;where n = the number of reflections and p = the number of parameters refined.

(7) R. G. Parr and W. Yang, Density Functional Theory of Atoms and Molecules. Oxford University Press: New York, 1989.

(8) A. D. Bochevarov, E. Harder, T. F. Hughes, J. R. Greenwood, D. A. Braden, D. M. Philipp, D. Rinaldo, M. D. Halls, J. Zhang and R. Friesner, *Int. J. Quantum Chem.* 2013, **113***,* 2110.

(9) J. C. Slater, Quantum Theory of Molecules and Solids, Vol. 4: The Self-Consistent Field for Molecules and Solids; McGraw-Hill: New York, 1974.

(10) S. H. Vosko, L. Wilk, M. Nusair, *Can. J. Phys.* 1980, **58***,* 1200.

(11) A. D. Becke, *Phys. Rev. A* 1988, **38***,* 3098.

(12) A. D. Becke, *J. Chem. Phys.* 1993, **98***,* 5648.

(13) C. Lee, W. Yang and R. G. Parr, *Phys. Rev. B* 1988, **37***,* 785.

(14) S. Grimme, J. Antony, S. Ehrlich and H. Krieg, *J. Chem. Phys.* 2010**, 132**, 154104.

(15) P. J. Hay and W. R. Wadt, *J. Chem. Phys.* 1985, **82***,* 270.

(16) P. J. Hay and W. R. Wadt, *J. Chem. Phys.* 1985, **82**, 299.

(17) W. R. Wadt, P. J. Hay, *J. Chem. Phys.* 1985, **82**, 284.

(18) T. H. Dunning, Jr. *J. Chem. Phys.* 1989, **90**, 1007.

(19) B. Marten, K. Kim, C. Cortis, R. A. Friesner, R. B. Murphy, M. N. Ringnalda, D. Sitkoff and B. Honig, *J. Phys. Chem.* 1996, **100**, 11775.

(20) M. Friedrichs, R. H. Zhou, S. R. Edinger and R. A. Friesner, *J. Phys. Chem. B* 1999, **103**, 3057.

(21) S. R. Edinger, C. Cortis, P. S. Shenkin and R. A. Friesner, *J. Phys. Chem. B* 1997, **101**, 1190.

(22) A. A. Rashin and B. Honig, *J. Phys. Chem.* 1985, **89**, 5588.

(23) E. V. Lenthe and E. J. Baerends, E. J. *J. Comput. Chem.* 2003, **24**, 1142.
